# Supplementary material for: TBK1-Zyxin signaling controls tumor-associated macrophage recruitment to mitigate antitumor immunity
Source: EMBO J. 2024 Sep 20;43(21):4984–5017. doi: 10.1038/s44318-024-00244-9 (PMC11535546; doi:10.1038/s44318-024-00244-9)
Supplement: Supplementary file 1 — Appendix [file 44318_2024_244_MOESM1_ESM.pdf]

# Appendix Tables

Appendix Table S1. List of Recombinant DNA ........................................................................... 1

Appendix Table S2. Antibodies Used in Study ........................................................................... 2

Appendix Table S3. Oligos Used in Study ................................................................................. 3

# Appendix Table S1. List of Recombinant DNA

| DNA | SOURCE | IDENTIFIER |
|---|---|---|
| pX330 | Addgene | Cat#42230 |
| pGEX-6P-1 | Lab stored | N/A |
| pCMV-STING-Flag | Lab stored | N/A |
| pCMV-STING R281Q-Flag | Lab stored | N/A |
| pCMV-STING-HA | Lab stored | N/A |
| pCMV-TBK1-HA | Lab stored | N/A |
| pRK5-TBK1-Myc | Lab stored | N/A |
| pRK5-TBK1 K38A-Myc | Lab stored | N/A |
| pRK5-AKT1-Flag | Lab stored | N/A |
| pRK5-HA-TBK1 | Lab stored | N/A |
| pRK5-HA-TBK1 K38A | Lab stored | N/A |
| pRK5-TBK1 (1-299)-Myc | Lab stored | N/A |
| pRK5-TBK1 (1-382)-Myc | Lab stored | N/A |
| pRK5-TBK1 (299-729)-Myc | Lab stored | N/A |
| pRK5-Myc-IRF3 2SA | Lab stored | N/A |
| pRK5-Zyxin-Flag | This paper | N/A |
| pRK5-Zyxin-Myc | This paper | N/A |
| pCMV-Zyxin-Flag | This paper | N/A |
| pRK5-Zyxin S142D-Flag | This paper | N/A |
| pRK5-Zyxin S143D-Flag | This paper | N/A |
| pRK5-Zyxin S142/143D-Flag | This paper | N/A |
| pRK5-Zyxin S150D-Flag | This paper | N/A |
| pRK5-Zyxin S313D-Flag | This paper | N/A |
| pRK5-Zyxin S142/143A-Flag | This paper | N/A |
| pRK5-VASP-Myc | This paper | N/A |
| pRK5-Zyxin (1-220)-Flag | This paper | N/A |
| pRK5-Zyxin (1-380)-Flag | This paper | N/A |
| pRK5-Zyxin (380-572)-Flag | This paper | N/A |

## Appendix Table S2. Antibodies Used in Study

| **Antibody** | **Company** | **Catalog** | **Species** | **Clone** | | **Dilution** | | | |
|---|---|---|---|---|---|---|---|---|---|
| | | | | | **WB** | **IP** | **IF** | **IHC** | **F** |
| Rhodamine phalloidin | Invitrogen | R415 | | | | | 1:1000 | | |
| anti-CD11b | Cell Signaling Technology | 46512S | Rat | Monoclonal | | | 1:100 | | |
| anti-pSTING (S365) | Cell Signaling Technology | 72971S | Rabbit | Monoclonal | 1:1000 | | | | |
| anti-pSTING (S365) | Cell Signaling Technology | 62912S | Rabbit | Monoclonal | 1:1000 | | 1:100 | | |
| anti-STING | Cell Signaling Technology | 50494S | Rabbit | Monoclonal | 1:1000 | | 1:200 | | |
| anti-STING | Abcam | ab181125 | Rabbit | Monoclonal | | | 1:100 | | |
| anti-Zyxin | Abcam | ab109316 | Rabbit | Monoclonal | 1:10000 | 1:200 | 1:200 | 1:200 | |
| Alexa Fluor® 488 Anti-Zyxin | Abcam | ab237072 | Rabbit | Monoclonal | | | 1:200 | | |
| anti-pZyxin (S142/143) | Cell Signaling Technology | 8467S | Rabbit | Monoclonal | 1:1000 | | 1:100 | 1:100 | 1:50 |
| anti-VASP | Cell Signaling Technology | 3132 | Rabbit | Monoclonal | | | 1:200 | | |
| anti-pTBK1 (S172) | Cell Signaling Technology | 5483S | Rabbit | Monoclonal | 1:3000 | | 1:200 | | |
| anti-TBK1 | Cell Signaling Technology | 3504S | Rabbit | Monoclonal | 1:3000 | | | | |
| anti-TBK1 | Cell Signaling Technology | 38066S | Rabbit | Monoclonal | 1:3000 | | 1:100 | | |
| anti-TBK1 | Abcam | ab40676 | Rabbit | Monoclonal | 1:3000 | | | | |
| anti-pIRF3 (S396) | Cell Signaling Technology | 4947S | Rabbit | Monoclonal | 1:3000 | | | | |
| anti-pIRF3 (S396) | Cell Signaling Technology | 29047S | Rabbit | Monoclonal | 1:3000 | | | | |
| anti-IRF3 | Cell Signaling Technology | 4302S | Rabbit | Monoclonal | 1:2000 | | | | |
| anti-IRF3 | Abcam | ab76493 | Rabbit | Monoclonal | 1:2000 | | | | |
| anti-CD4 | eBioscience | 14976680 | Rat | Monoclonal | | | | 1:100 | |
| anti-CD8 | eBioscience | 14080880 | Rat | Monoclonal | | | | 1:100 | |
| anti-F4/80 | Bio-Rad | MCA497RT | Rat | Monoclonal | | | | 1:100 | |
| anti-Flag (M2) | Sigma | F3165 | Mouse | Monoclonal (M2 clone) | 1:5000 | 1:200 | 1:500 | | |
| anti-Flag M2 agarose | Sigma | A2220 | Mouse | Monoclonal (M2 clone) | IP (~20ul/2mgs proteins) | | | | |
| anti-HA | Cell Signaling Technology | 3724S | Rabbit | Monoclonal | 1:5000 | 1:200 | 1:200 | | |
| anti-HA | Sigma | H9658 | Mouse | Monoclonal | 1:3000 | 1:200 | 1:200 | | |
| anti-Myc | Cell Signaling Technology | 2276 | Mouse | Monoclonal | 1:3000 | 1:200 | 1:100 | | |
| anti-β-Actin | Sigma | A5441 | Mouse | Monoclonal | 1:20000 | | | | |
| | | | | | | | | | |
| **Alexa-labeled secondary antibodies** | | | | | | | | | |
| | Jackson | 111-095-003 | | | | | 1:500 | | |
| | Jackson | 115-095-003 | | | | | 1:500 | | |
| | Jackson | 111-025-003 | | | | | 1:500 | | |
| | Jackson | 115-025-003 | | | | | 1:500 | | |
| | | | | | | | | | |
| **Antibody for FACS** | | | | | | | | | |
| FITC anti-mouse CD45 | Biolegend | 147710 | Rat | Monoclonal | | | | | 1:200 |
| BV421 anti-mouse F4/80 | Biolegend | 123131 | Rat | Monoclonal | | | | | 1:200 |
| PE anti-mouse/human CD11b | Biolegend | 101208 | Rat | Monoclonal | | | | | 1:200 |
| APC anti-mouse CD11c | Biolegend | 117310 | Armenian Hamster | Monoclonal | | | 1:100 | | 1:200 |
| PE anti-mouse CD45 | Biolegend | 103106 | Rat | Monoclonal | | | | | 1:200 |
| APC anti-mouse CD3 | Biolegend | 100236 | Rat | Monoclonal | | | | | 1:200 |
| Pacific Blue™ anti-mouse CD4 | Biolegend | 116008 | Rat | Monoclonal | | | | | 1:200 |
| PerCP/Cyanine5.5 anti-mouse CD8a | Biolegend | 100734 | Rat | Monoclonal | | | | | 1:100 |
| TruStain FcX™ (anti-mouse CD16/32) | Biolegend | 101319 | Rat | Monoclonal | | | | | |

# Appendix Table S3. Oligos Used in Study

| qPCR Primers | Sequence | Species | Company |
|---|---|---|---|
| Zyxin-Forward Primer | 5'-CCGATGATCGAGGAACCATTC-3' | mouse | biosune |
| Zyxin-Reverse Primer | 5'-CGTTCTTGGTCATGTCGTCCA-3' | mouse | biosune |

| Cas9 Primers | Sequence | Species | Company |
|---|---|---|---|
| Zyxin-Cas9-Forward Primer-SET1 | 5'-CACCGATCATTTCCCCCTGCGCCTC-3' | human | biosune |
| Zyxin-Cas9-Reverse Primer-SET1 | 5'-AAACGAGGCGCAGGGGGAAATGATC-3' | human | biosune |
| Zyxin-Cas9-Forward Primer-SET2 | 5'-CACCGTCTGCCCCAGGTTCCGGCTC-3' | human | biosune |
| Zyxin-Cas9-Reverse Primer-SET2 | 5'-AAACGAGCCGGAACCTGGGGCAGAC-3' | human | biosune |
| Zyxin-Cas9-Forward Primer-SET1 | 5'-CACCGCCGGATGCTCCTTCTTCTGT-3' | mouse | biosune |
| Zyxin-Cas9-Reverse Primer-SET1 | 5'-AAACACAGAAGAAGGAGCATCCGGC-3' | mouse | biosune |
| Zyxin-Cas9-Forward Primer-SET2 | 5'-CACCGTCTGCTAATACACAGCCCCG-3' | mouse | biosune |
| Zyxin-Cas9-Reverse Primer-SET2 | 5'-AAACCGGGGCTGTGTATTAGCAGAC-3' | mouse | biosune |
