# Supplementary figures and images for: TBK1-Zyxin signaling controls tumor-associated macrophage recruitment to mitigate antitumor immunity (part 1 of 2)
Source: EMBO J. 2024 Sep 20;43(21):4984–5017. doi: 10.1038/s44318-024-00244-9 (PMC11535546; doi:10.1038/s44318-024-00244-9)

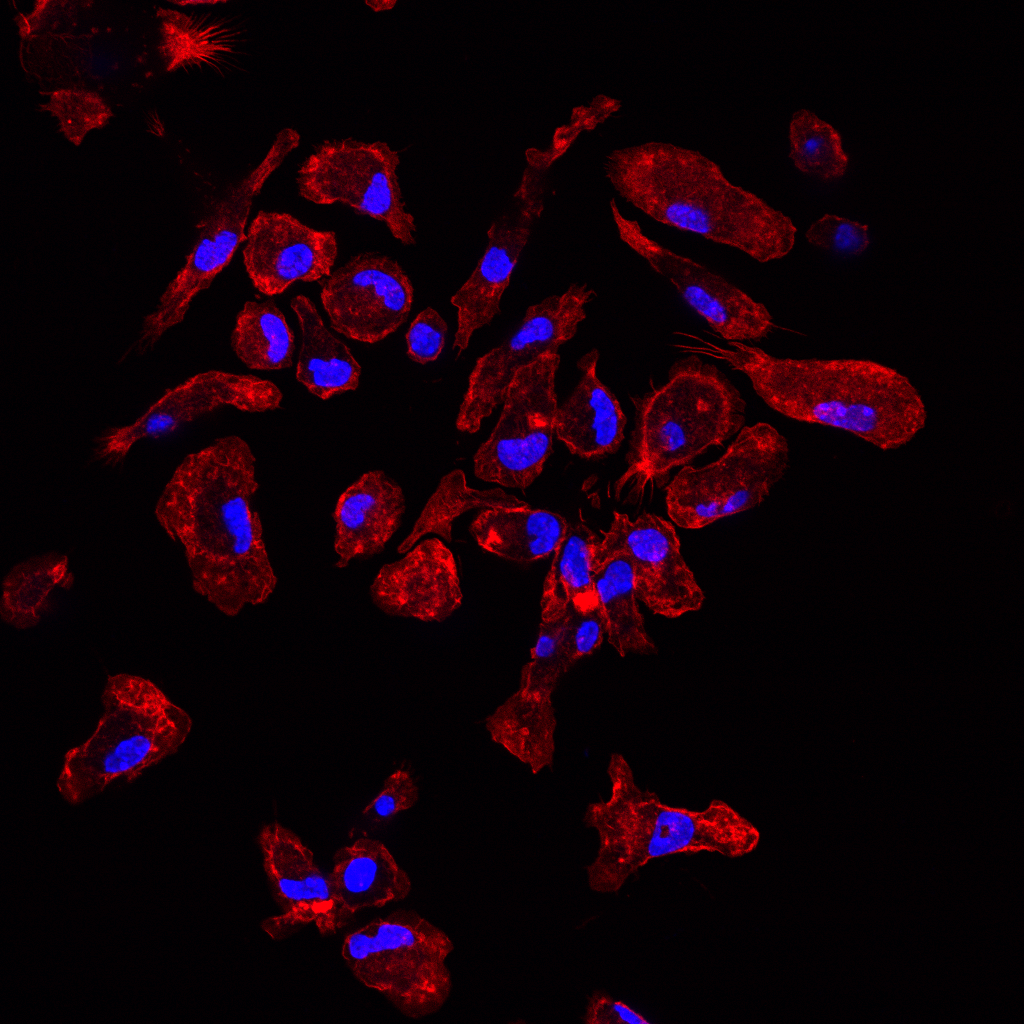

Supplement: Supplementary file 5 — Source data Fig. 1 [file 44318_2024_244_MOESM5_ESM.zip › Figure 1/1A/Control.tif]

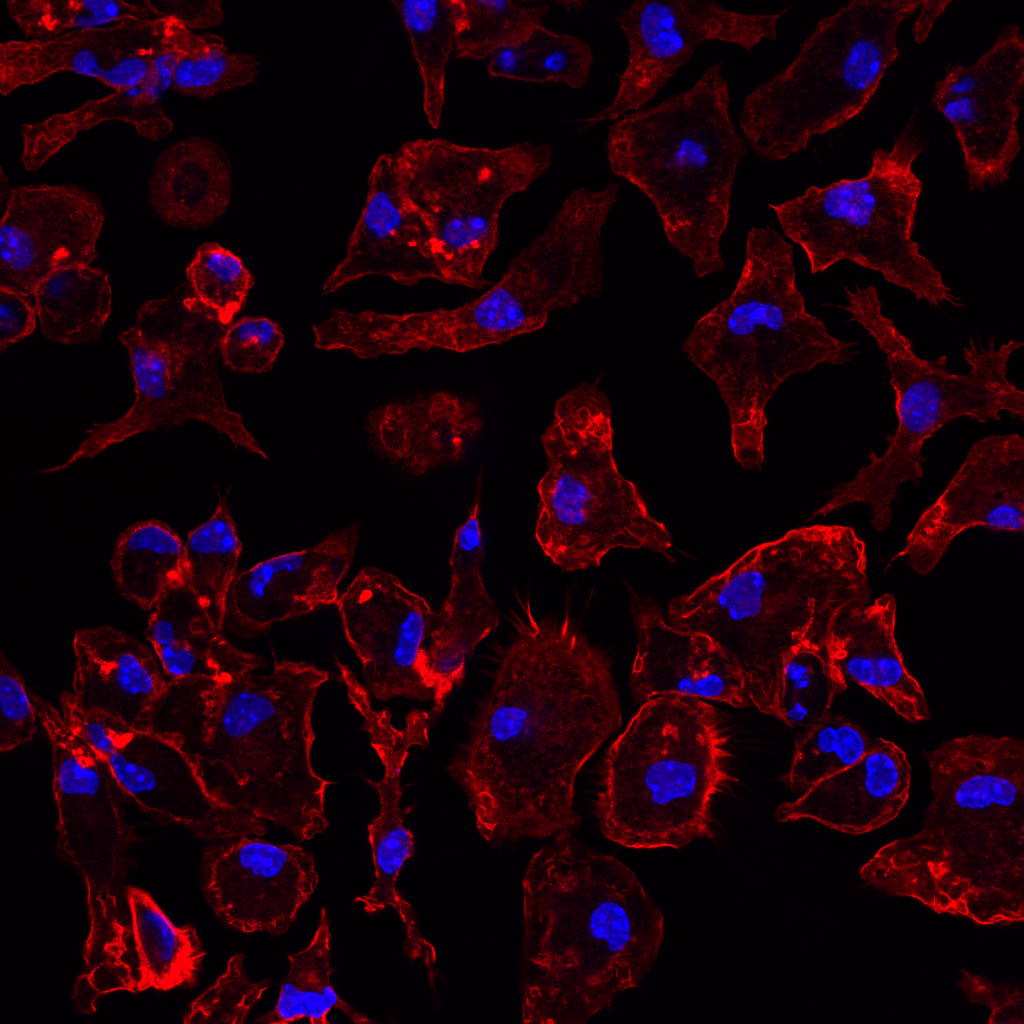

Supplement: Supplementary file 5 — Source data Fig. 1 [file 44318_2024_244_MOESM5_ESM.zip › Figure 1/1A/DMXAA.tif]

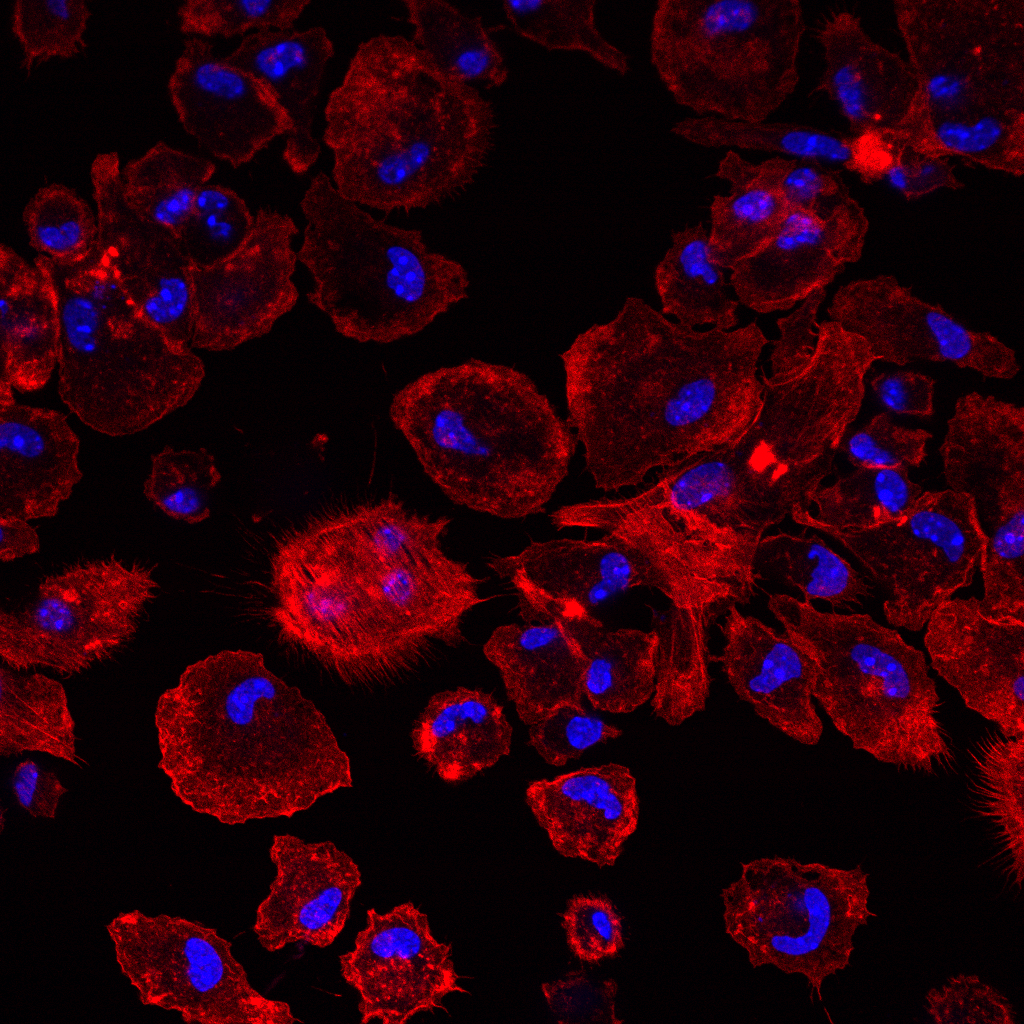

Supplement: Supplementary file 5 — Source data Fig. 1 [file 44318_2024_244_MOESM5_ESM.zip › Figure 1/1A/HSV-1.tif]

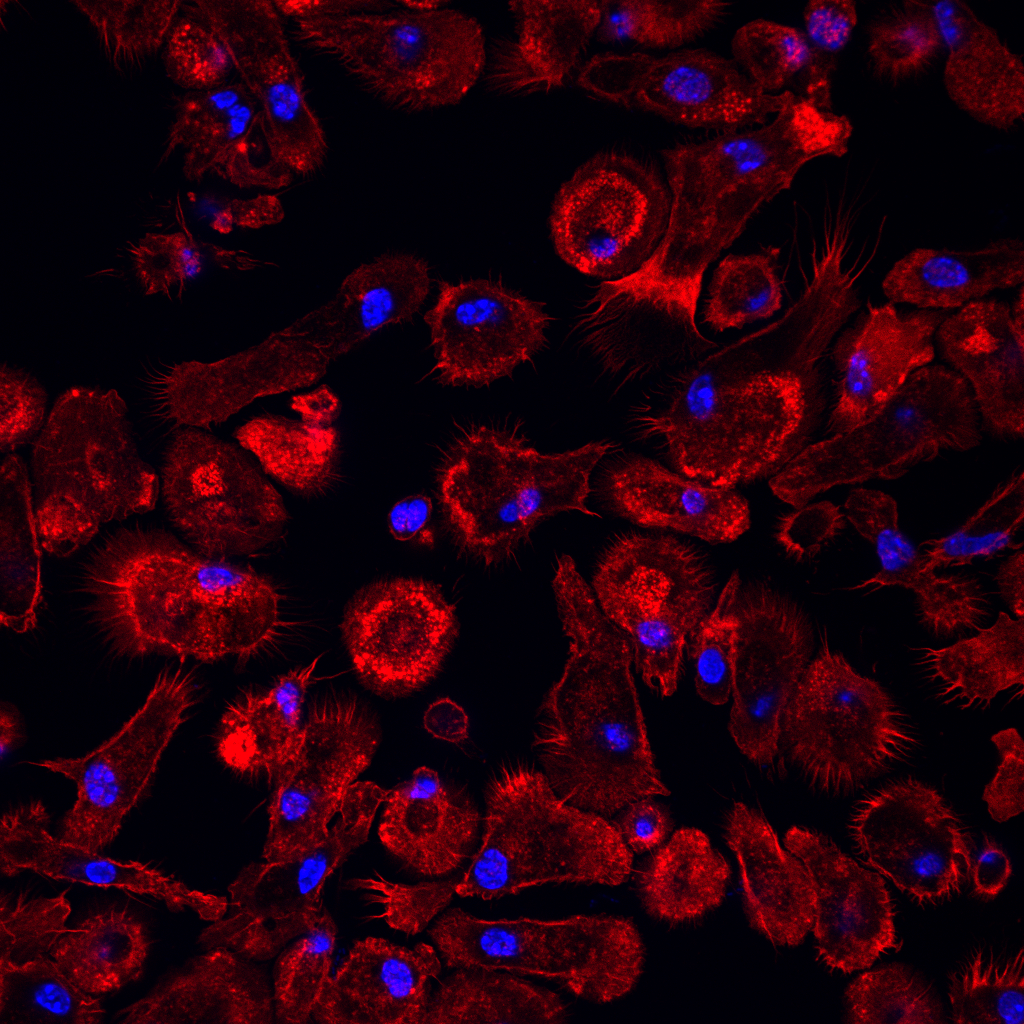

Supplement: Supplementary file 5 — Source data Fig. 1 [file 44318_2024_244_MOESM5_ESM.zip › Figure 1/1A/poly (I C).tif]

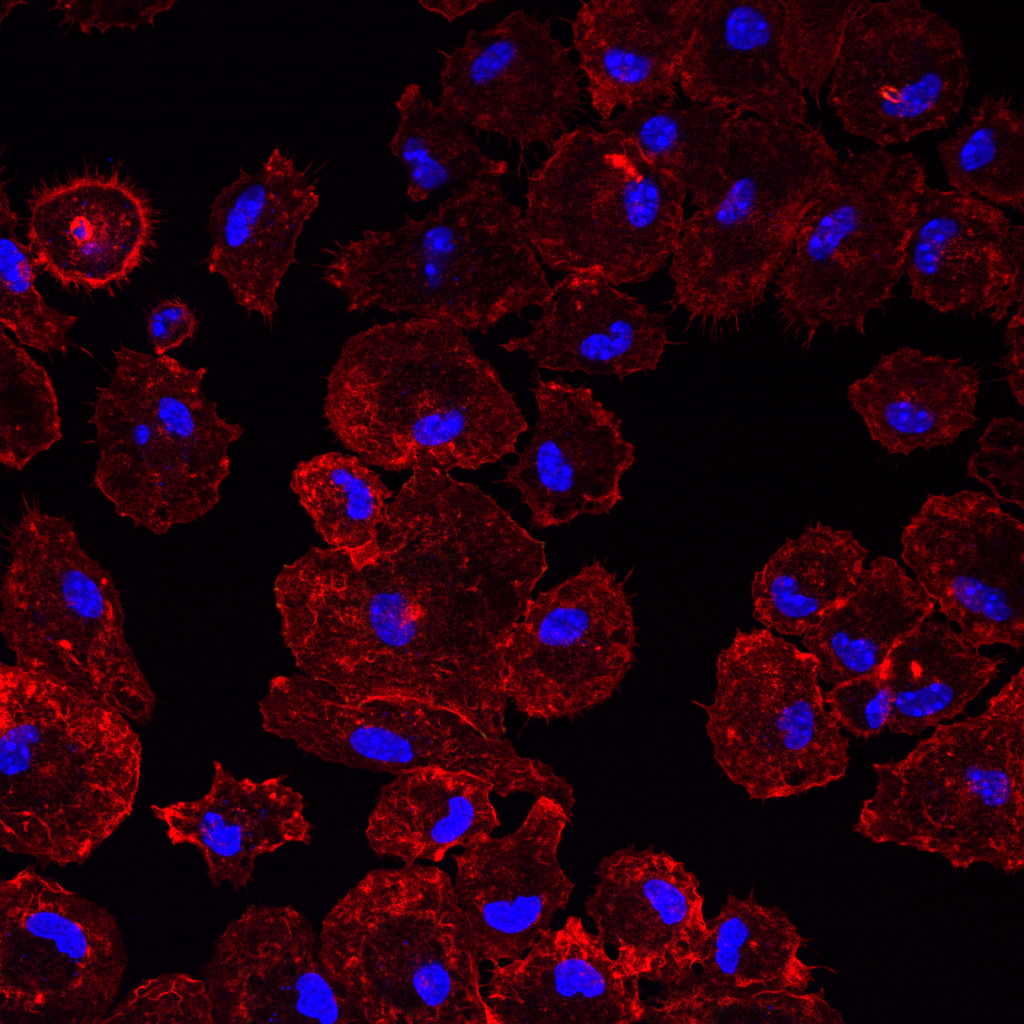

Supplement: Supplementary file 5 — Source data Fig. 1 [file 44318_2024_244_MOESM5_ESM.zip › Figure 1/1A/SeV.tif]

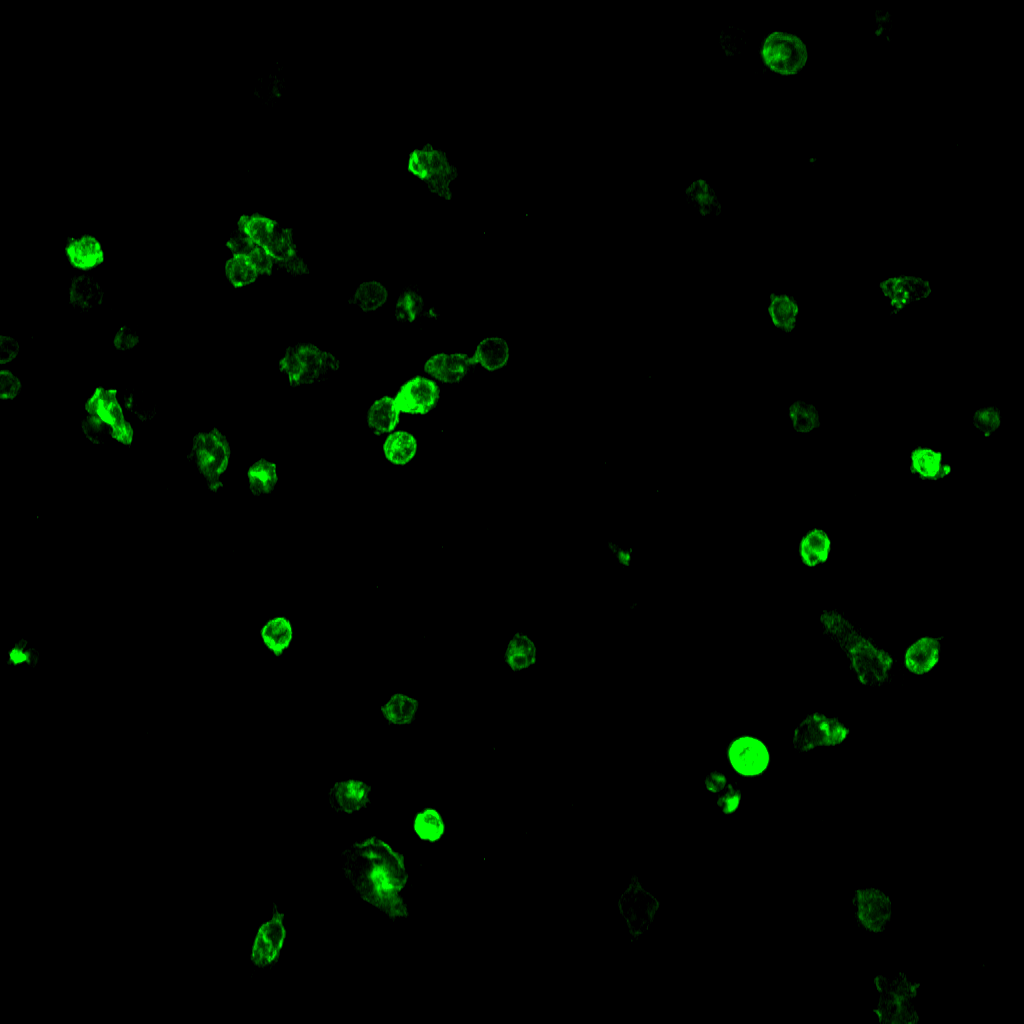

Supplement: Supplementary file 5 — Source data Fig. 1 [file 44318_2024_244_MOESM5_ESM.zip › Figure 1/1C/Ctrl PM adhesion in glass slides CD11b.tif]

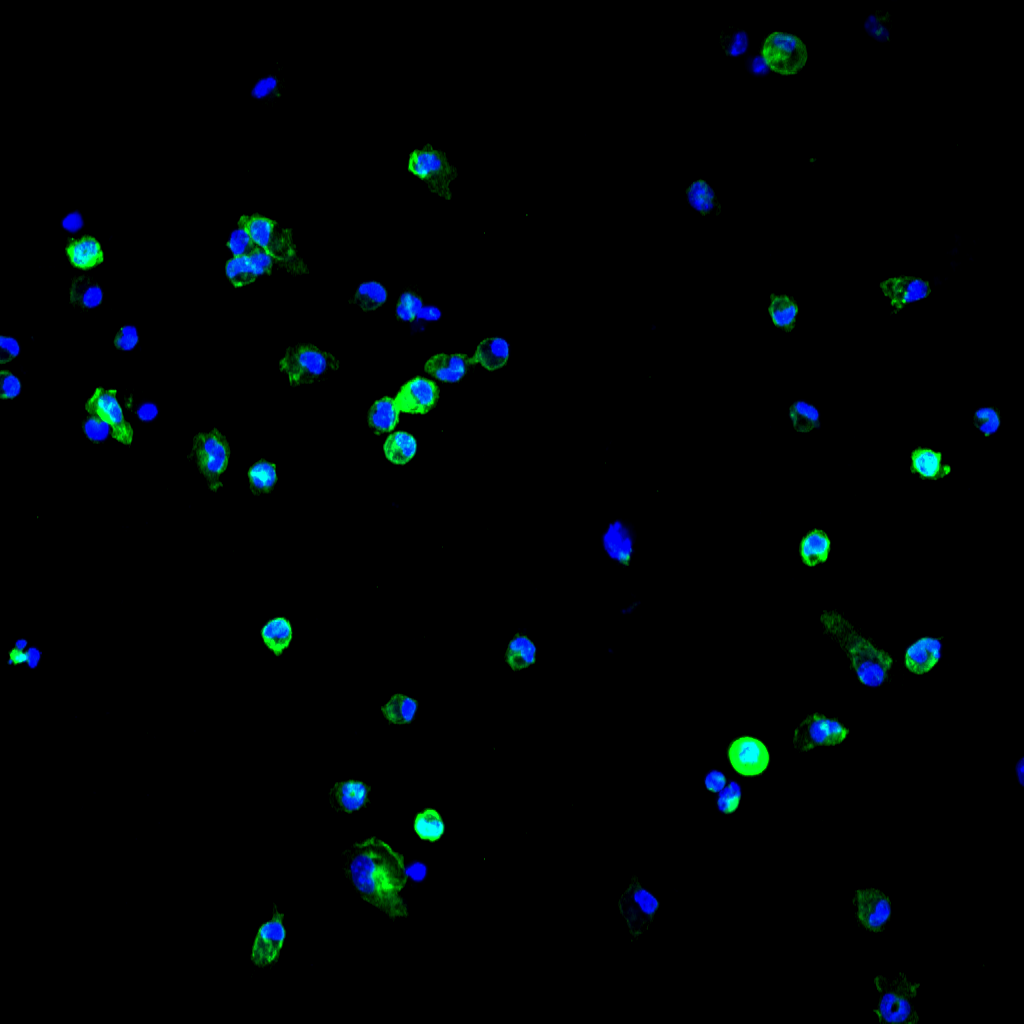

Supplement: Supplementary file 5 — Source data Fig. 1 [file 44318_2024_244_MOESM5_ESM.zip › Figure 1/1C/Ctrl PM adhesion in glass slides.tif]

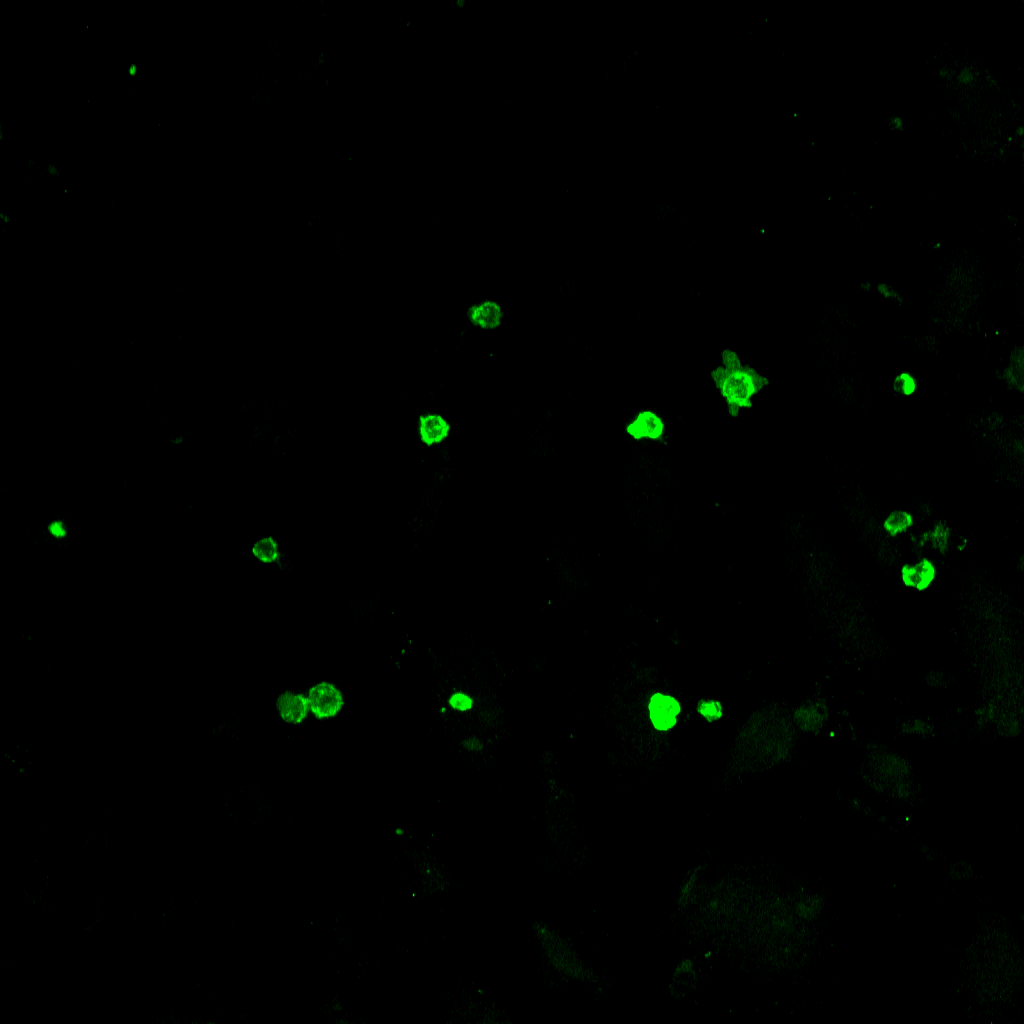

Supplement: Supplementary file 5 — Source data Fig. 1 [file 44318_2024_244_MOESM5_ESM.zip › Figure 1/1C/Ctrl PM adhesion in HUVECs CD11b.tif]

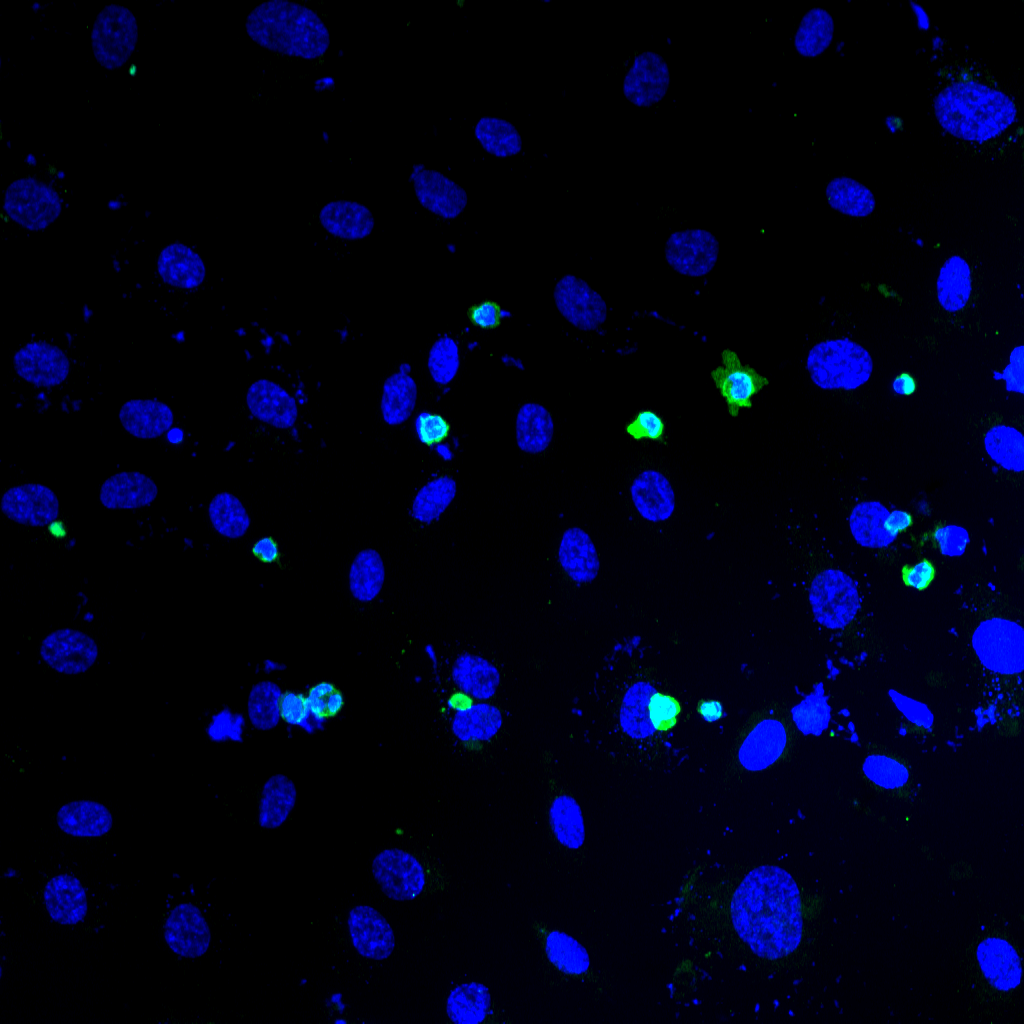

Supplement: Supplementary file 5 — Source data Fig. 1 [file 44318_2024_244_MOESM5_ESM.zip › Figure 1/1C/Ctrl PM adhesion in HUVECs.tif]

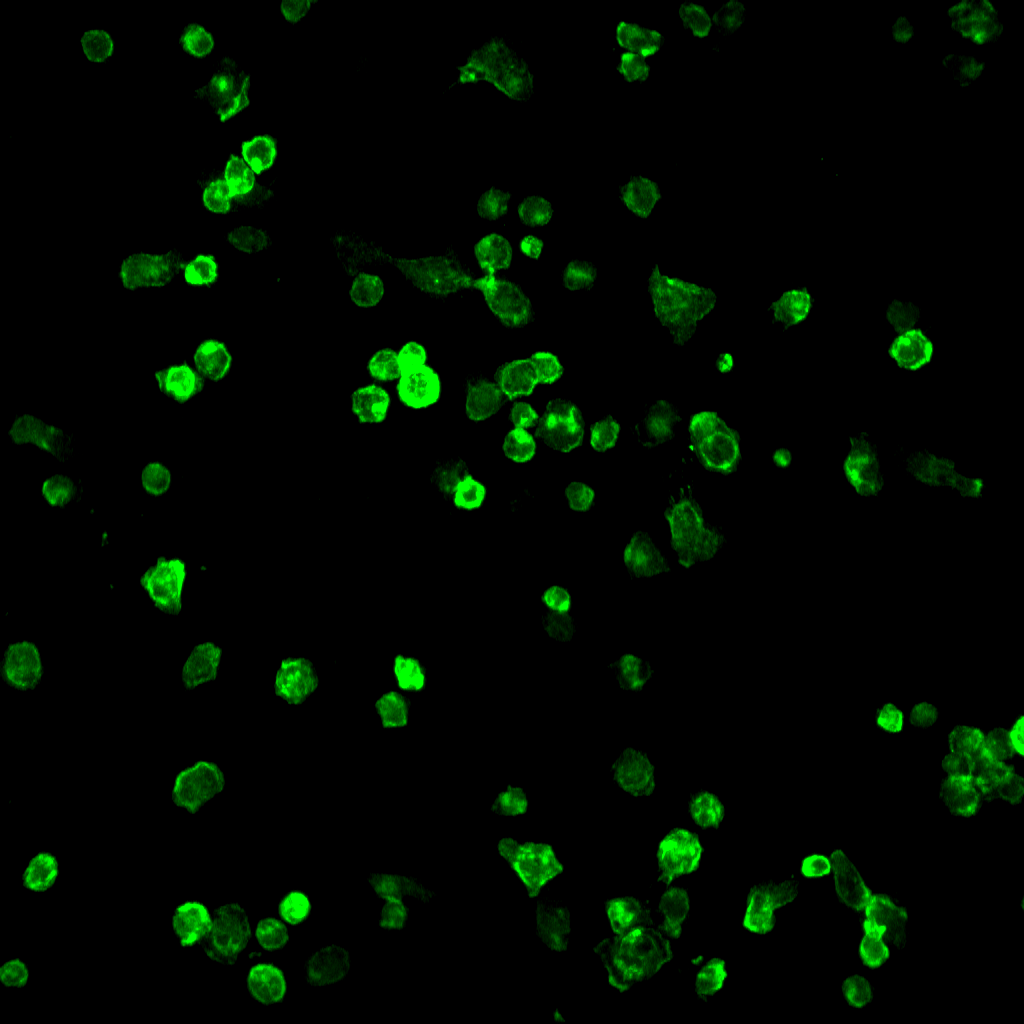

Supplement: Supplementary file 5 — Source data Fig. 1 [file 44318_2024_244_MOESM5_ESM.zip › Figure 1/1C/DMXAA PM adhesion in glass slides CD11b.tif]

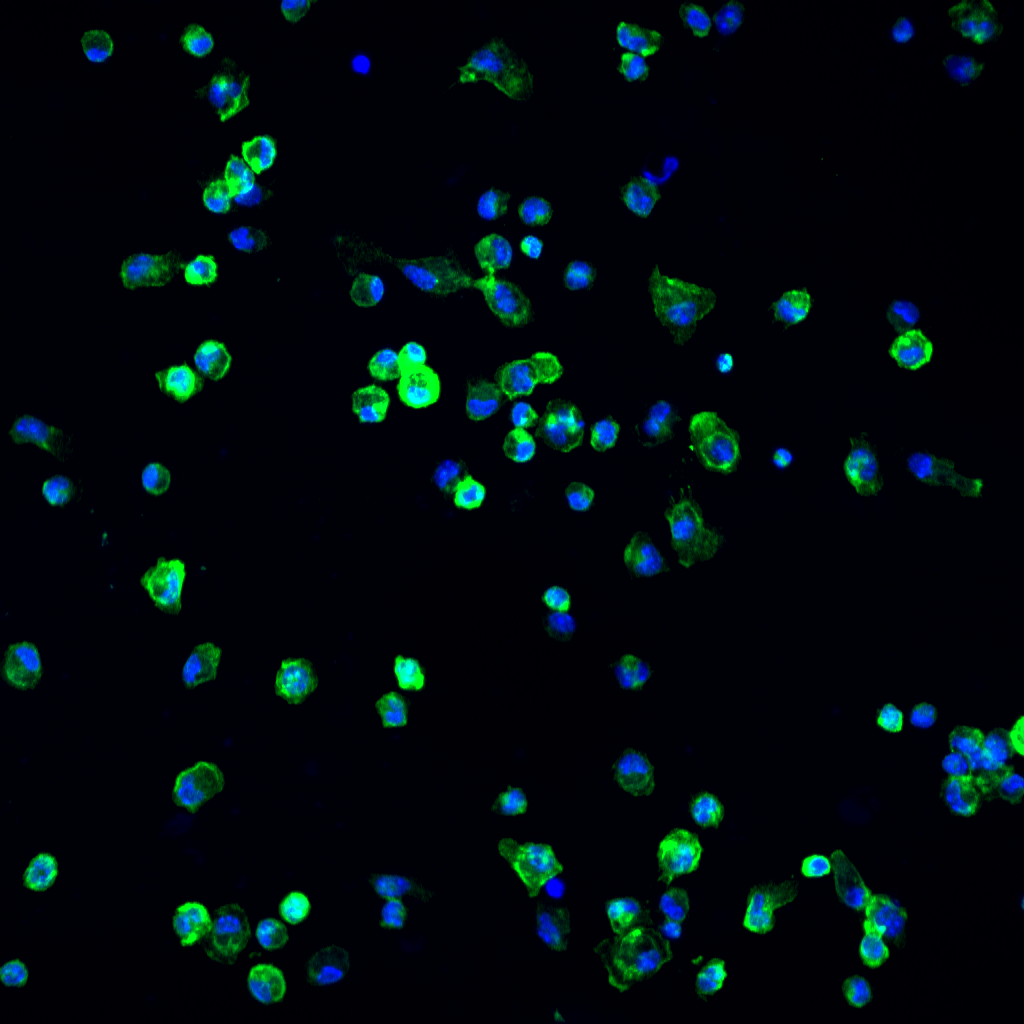

Supplement: Supplementary file 5 — Source data Fig. 1 [file 44318_2024_244_MOESM5_ESM.zip › Figure 1/1C/DMXAA PM adhesion in glass slides.tif]

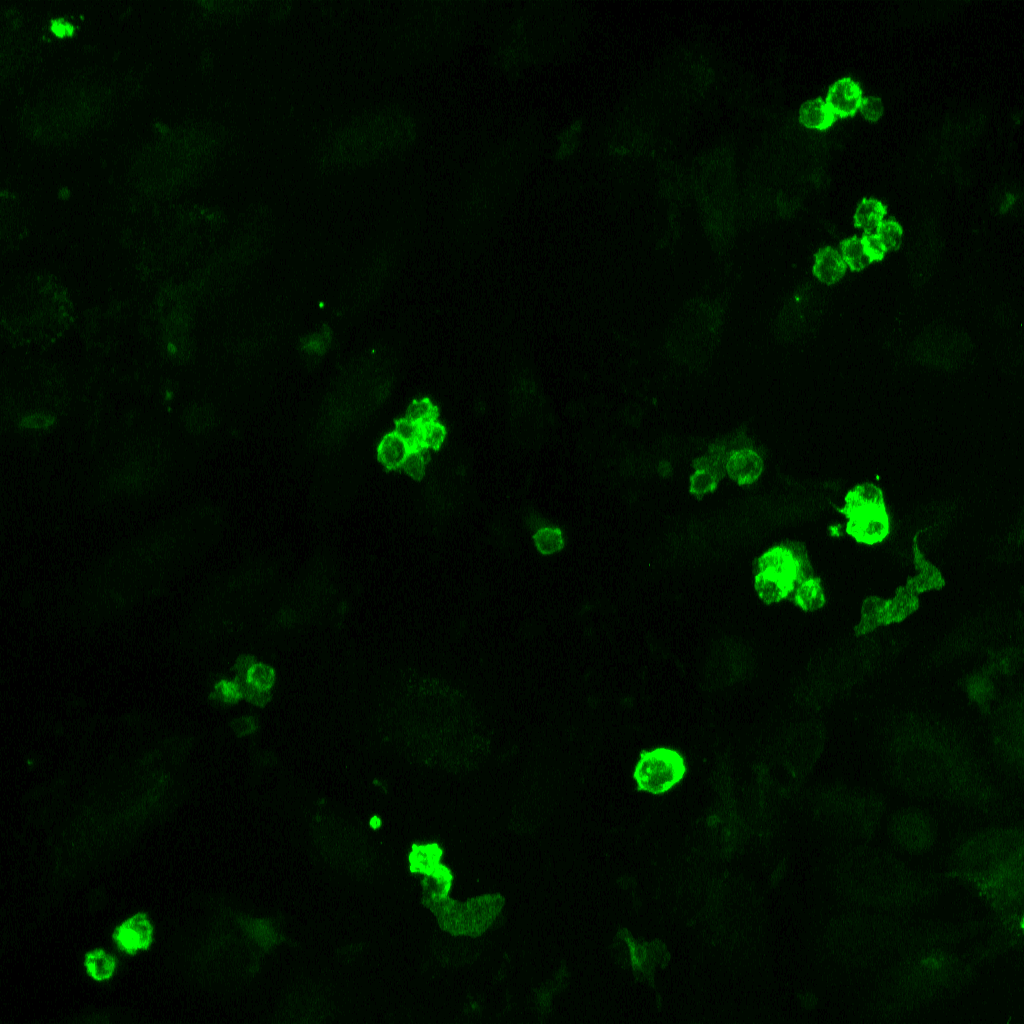

Supplement: Supplementary file 5 — Source data Fig. 1 [file 44318_2024_244_MOESM5_ESM.zip › Figure 1/1C/DMXAA PM adhesion in HUVECs CD11b.tif]

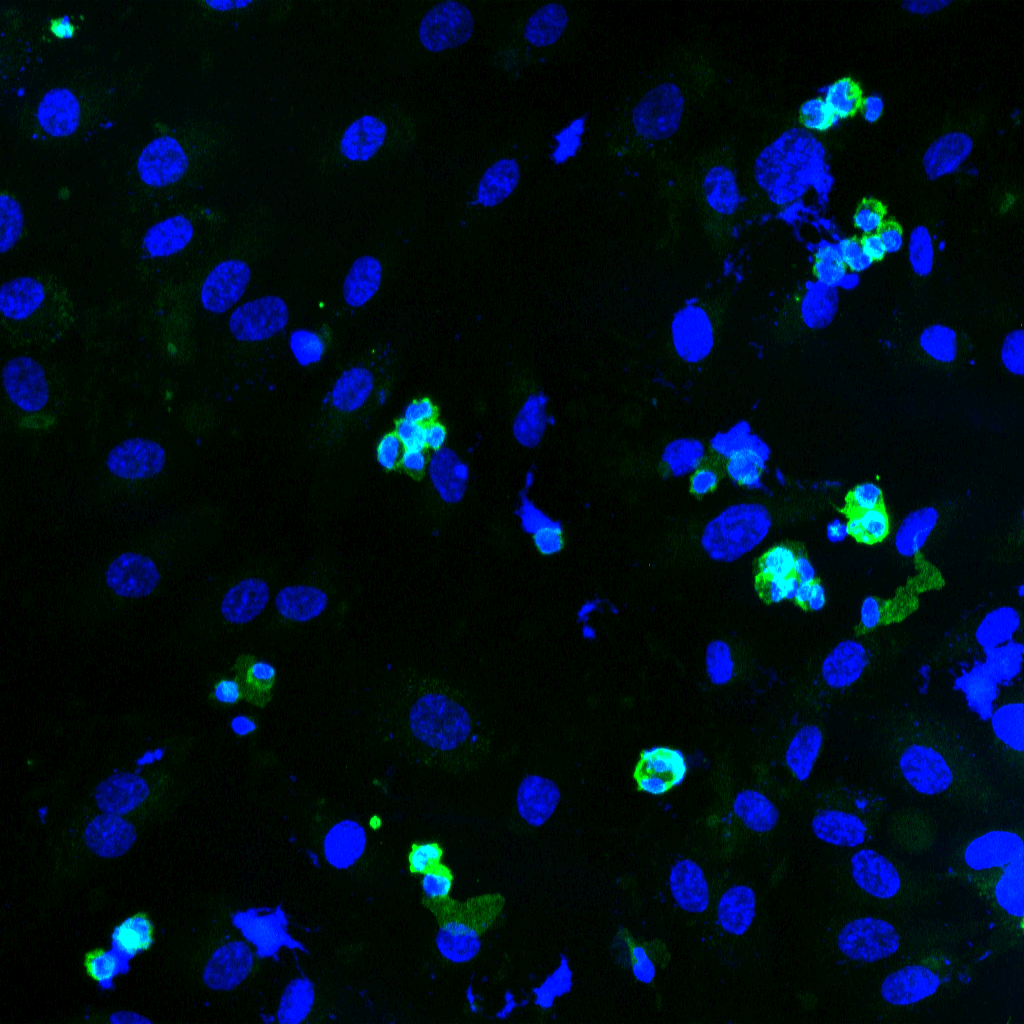

Supplement: Supplementary file 5 — Source data Fig. 1 [file 44318_2024_244_MOESM5_ESM.zip › Figure 1/1C/DMXAA PM adhesion in HUVECs.tif]

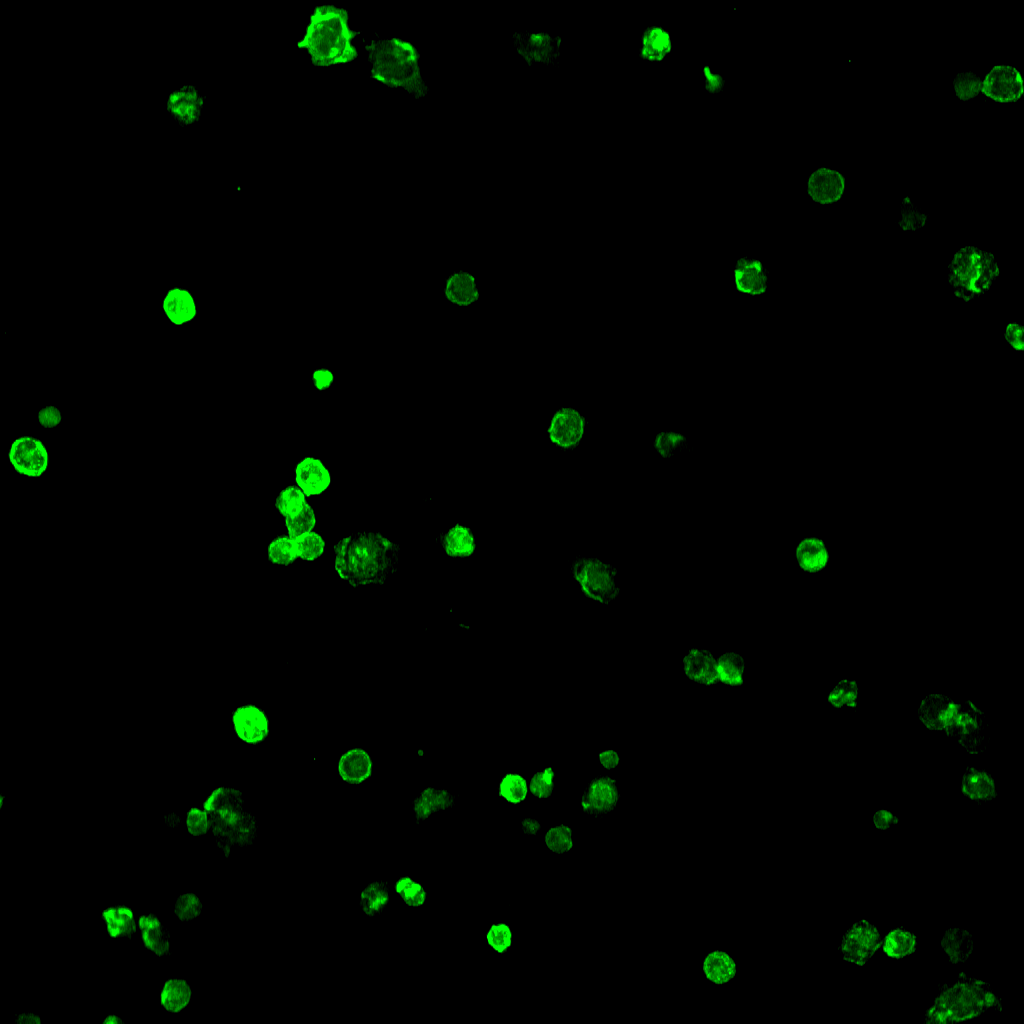

Supplement: Supplementary file 5 — Source data Fig. 1 [file 44318_2024_244_MOESM5_ESM.zip › Figure 1/1C/DMXAA+GSK8612 PM adhesion in glass slides CD11b.tif]

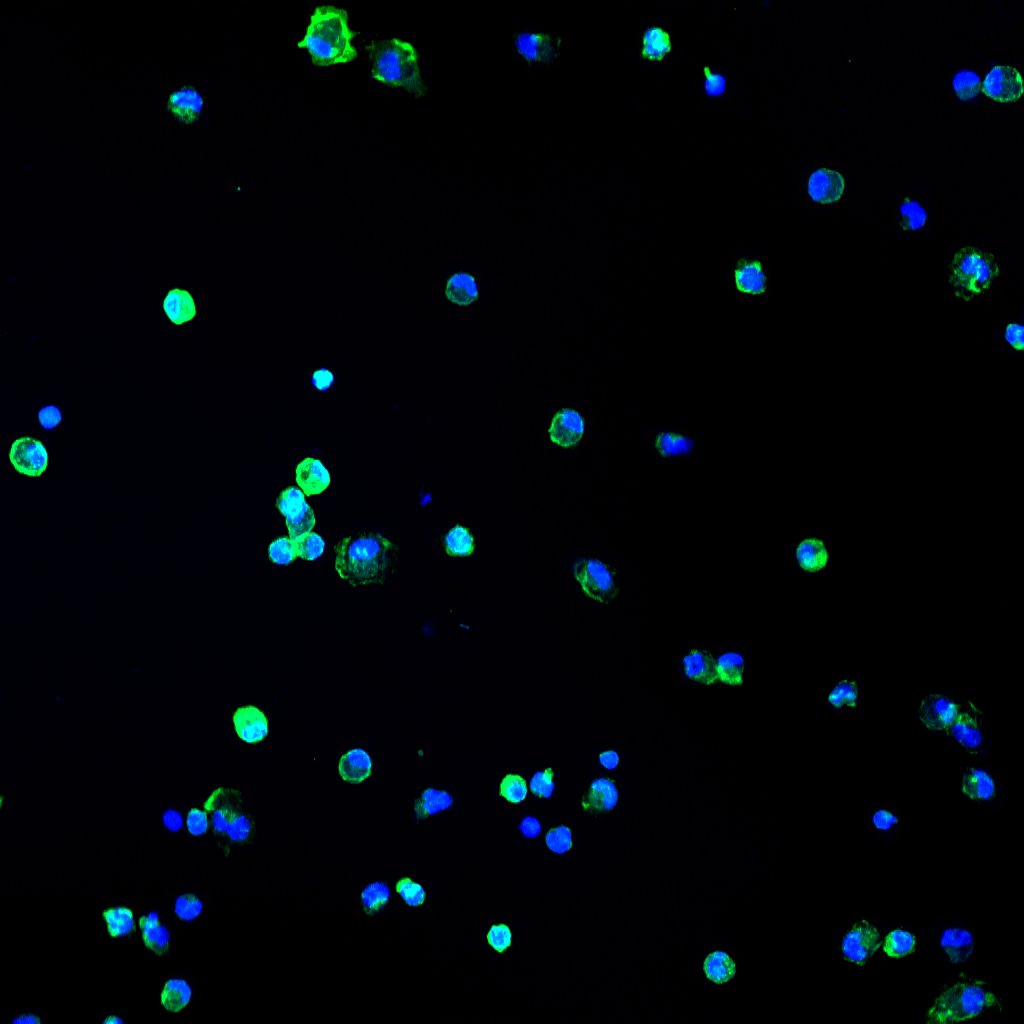

Supplement: Supplementary file 5 — Source data Fig. 1 [file 44318_2024_244_MOESM5_ESM.zip › Figure 1/1C/DMXAA+GSK8612 PM adhesion in glass slides.tif]

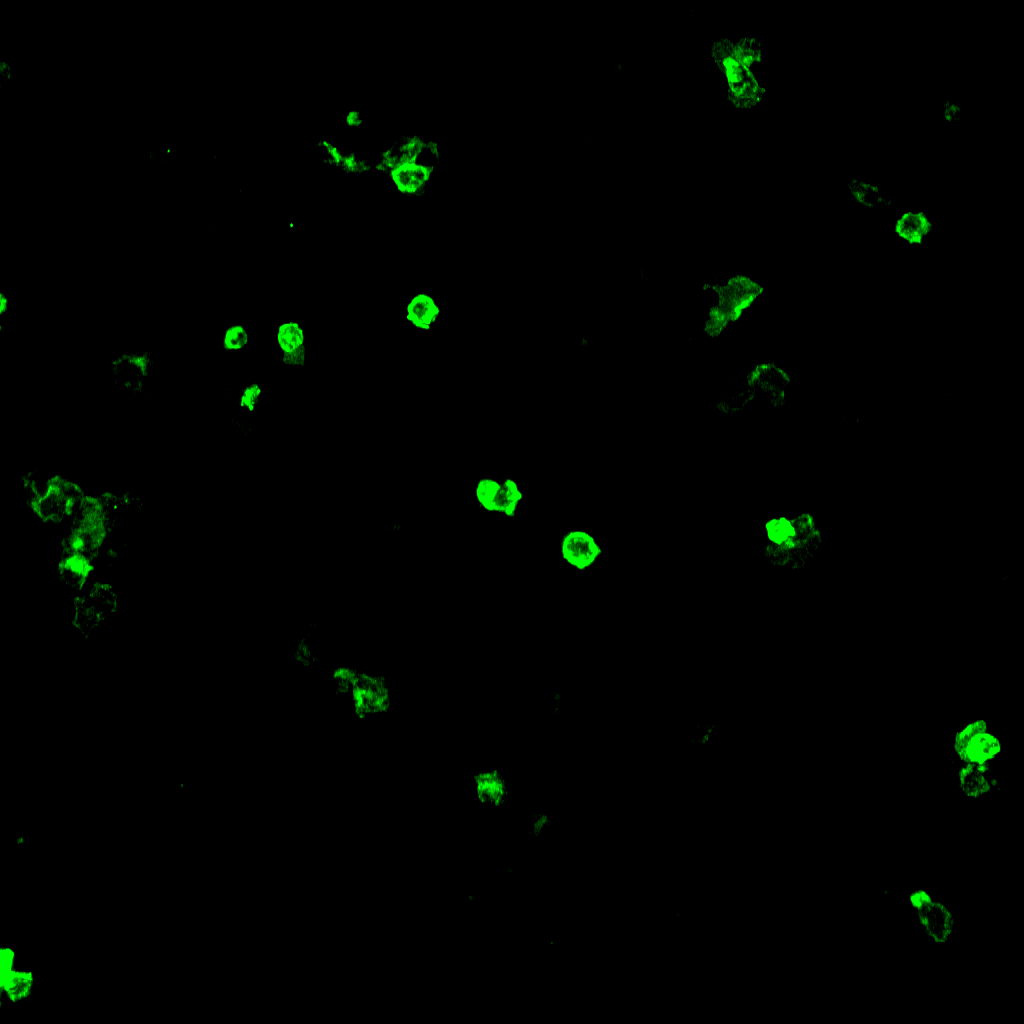

Supplement: Supplementary file 5 — Source data Fig. 1 [file 44318_2024_244_MOESM5_ESM.zip › Figure 1/1C/DMXAA+GSK8612 PM adhesion in HUVECs CD11b.tif]

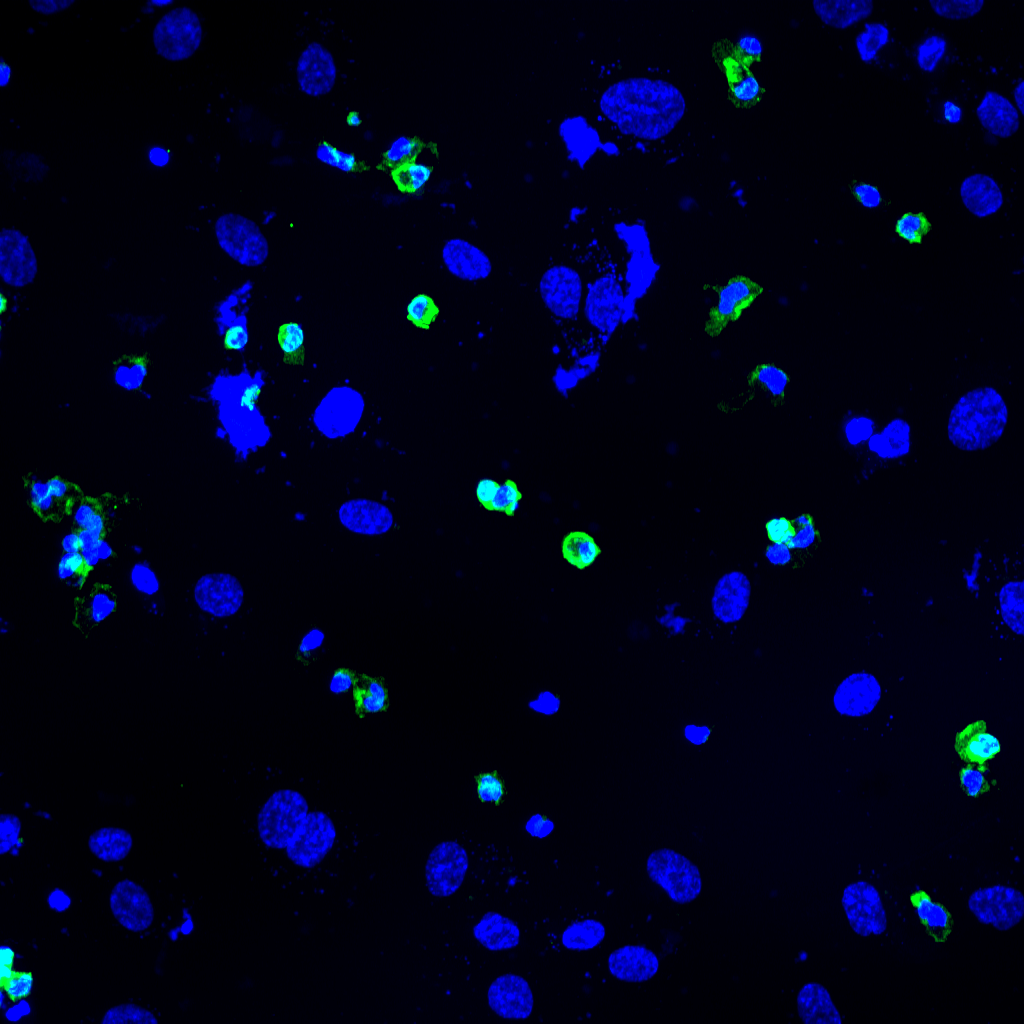

Supplement: Supplementary file 5 — Source data Fig. 1 [file 44318_2024_244_MOESM5_ESM.zip › Figure 1/1C/DMXAA+GSK8612 PM adhesion in HUVECs.tif]

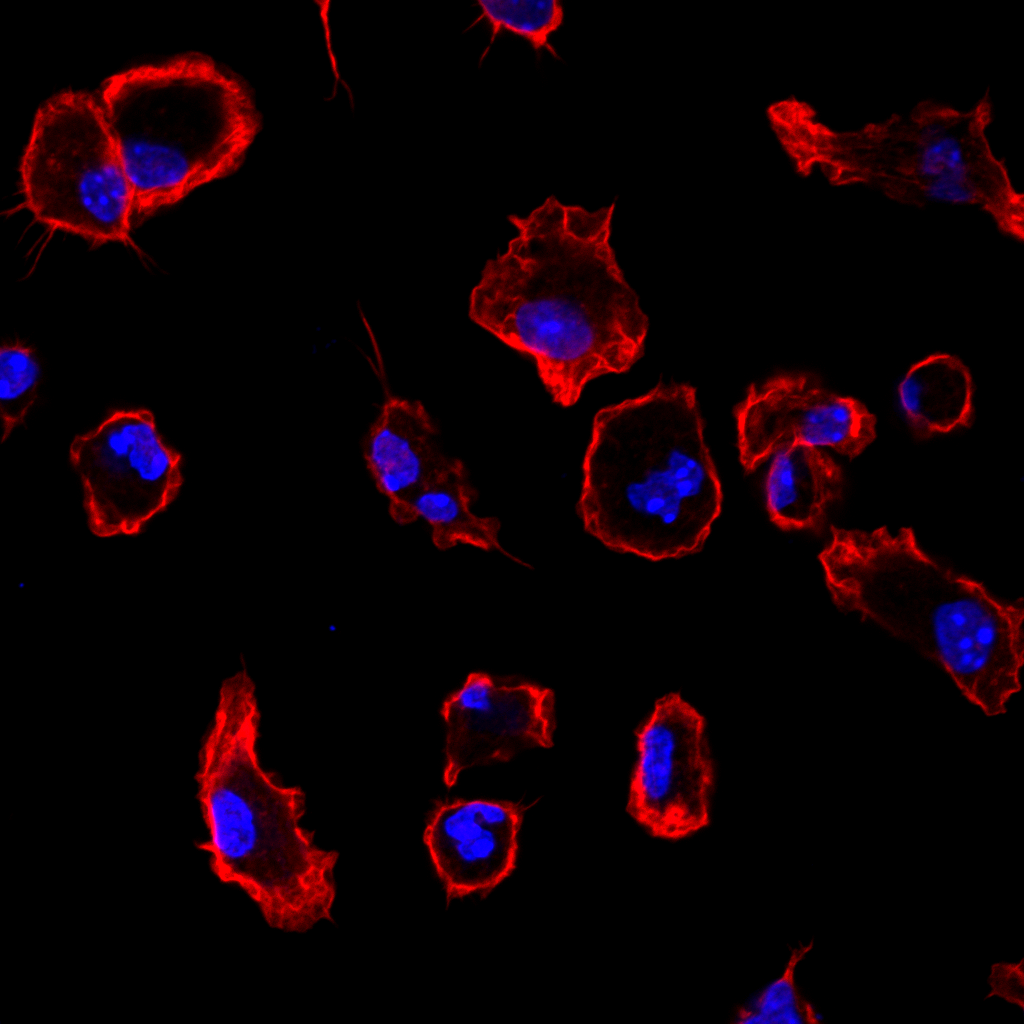

Supplement: Supplementary file 5 — Source data Fig. 1 [file 44318_2024_244_MOESM5_ESM.zip › Figure 1/1D/STING KO DMXAA.tif]

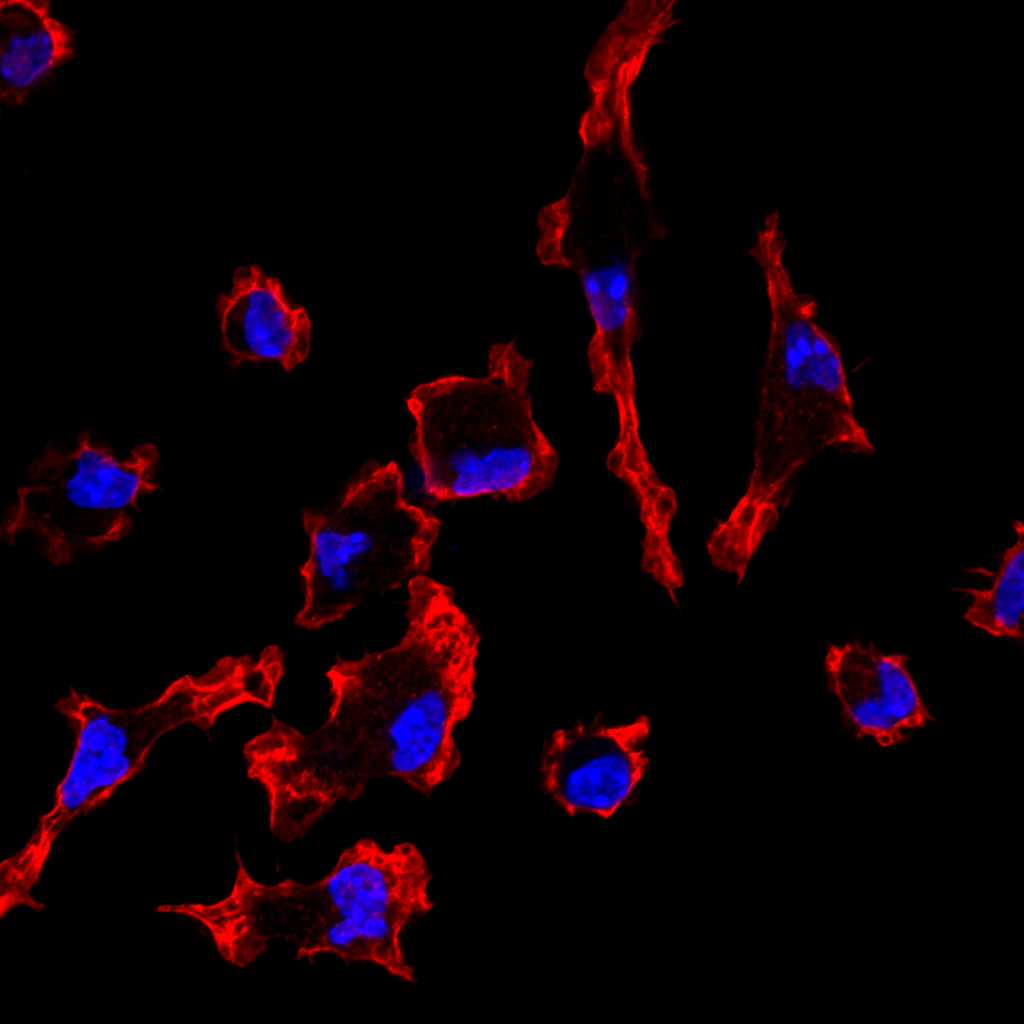

Supplement: Supplementary file 5 — Source data Fig. 1 [file 44318_2024_244_MOESM5_ESM.zip › Figure 1/1D/STING KO Vehicle.tif]

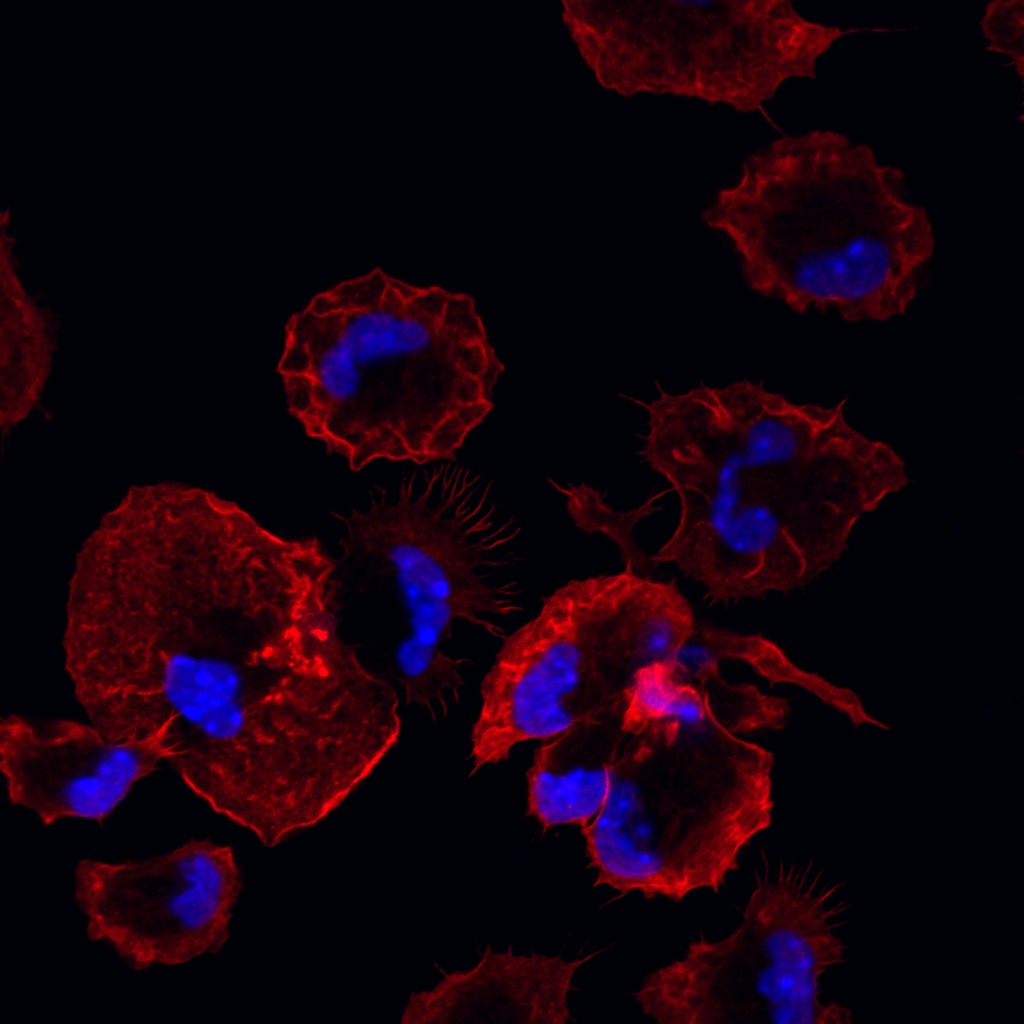

Supplement: Supplementary file 5 — Source data Fig. 1 [file 44318_2024_244_MOESM5_ESM.zip › Figure 1/1D/WT DMXAA.tif]

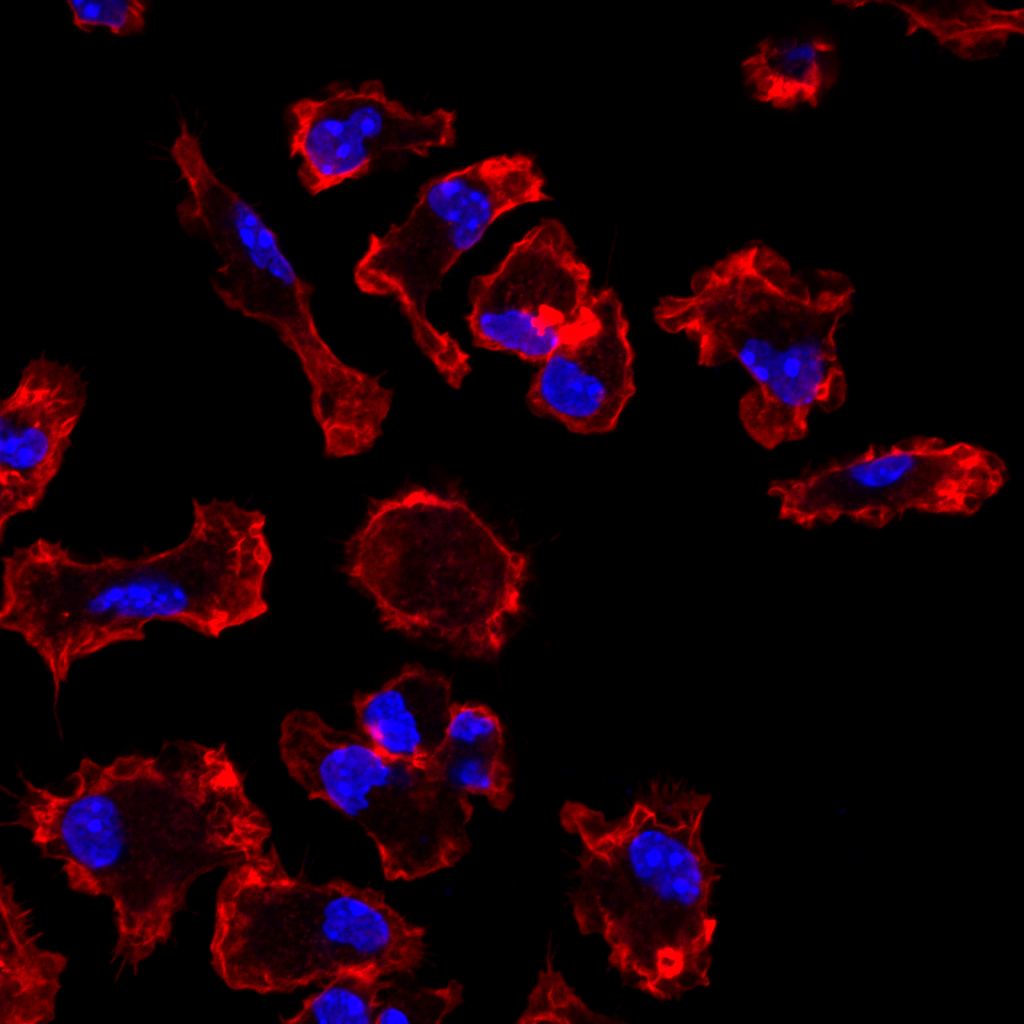

Supplement: Supplementary file 5 — Source data Fig. 1 [file 44318_2024_244_MOESM5_ESM.zip › Figure 1/1D/WT Vehicle.tif]

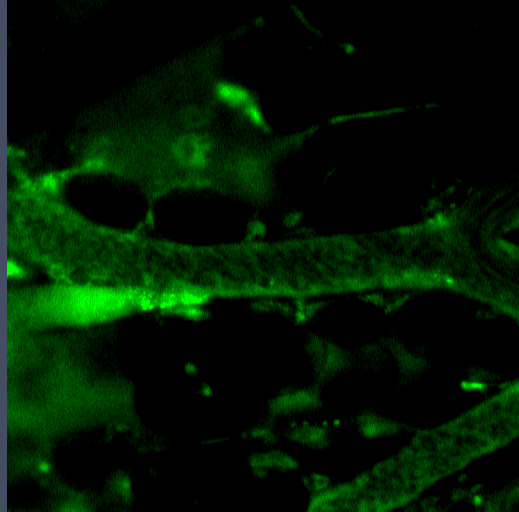

Supplement: Supplementary file 5 — Source data Fig. 1 [file 44318_2024_244_MOESM5_ESM.zip › Figure 1/1F/cGAMP Blood vessel.tif]

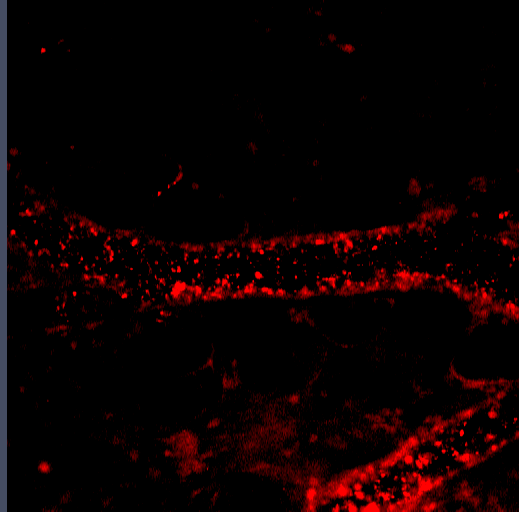

Supplement: Supplementary file 5 — Source data Fig. 1 [file 44318_2024_244_MOESM5_ESM.zip › Figure 1/1F/cGAMP Leukocyte.tif]

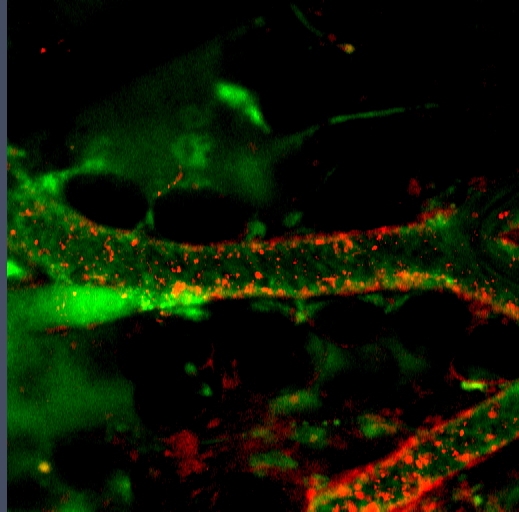

Supplement: Supplementary file 5 — Source data Fig. 1 [file 44318_2024_244_MOESM5_ESM.zip › Figure 1/1F/cGAMP Merge.tif]

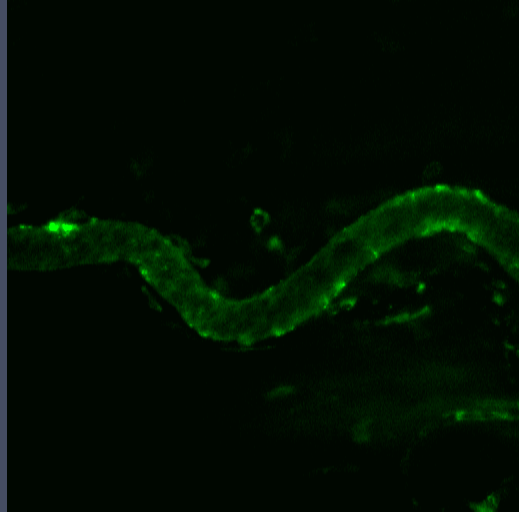

Supplement: Supplementary file 5 — Source data Fig. 1 [file 44318_2024_244_MOESM5_ESM.zip › Figure 1/1F/Vehicle Blood vessel.tif]

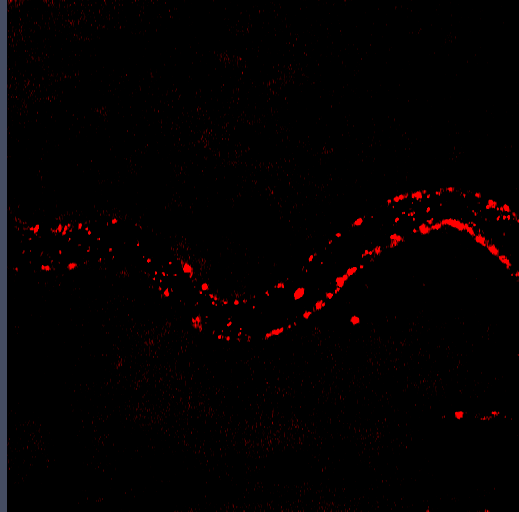

Supplement: Supplementary file 5 — Source data Fig. 1 [file 44318_2024_244_MOESM5_ESM.zip › Figure 1/1F/Vehicle Leukocyte.tif]

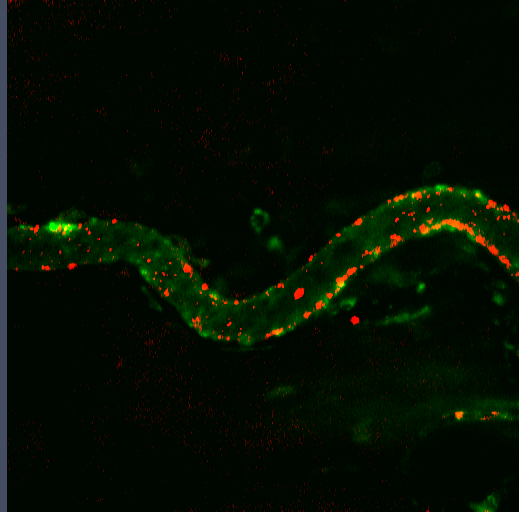

Supplement: Supplementary file 5 — Source data Fig. 1 [file 44318_2024_244_MOESM5_ESM.zip › Figure 1/1F/Vehicle Merge.tif]

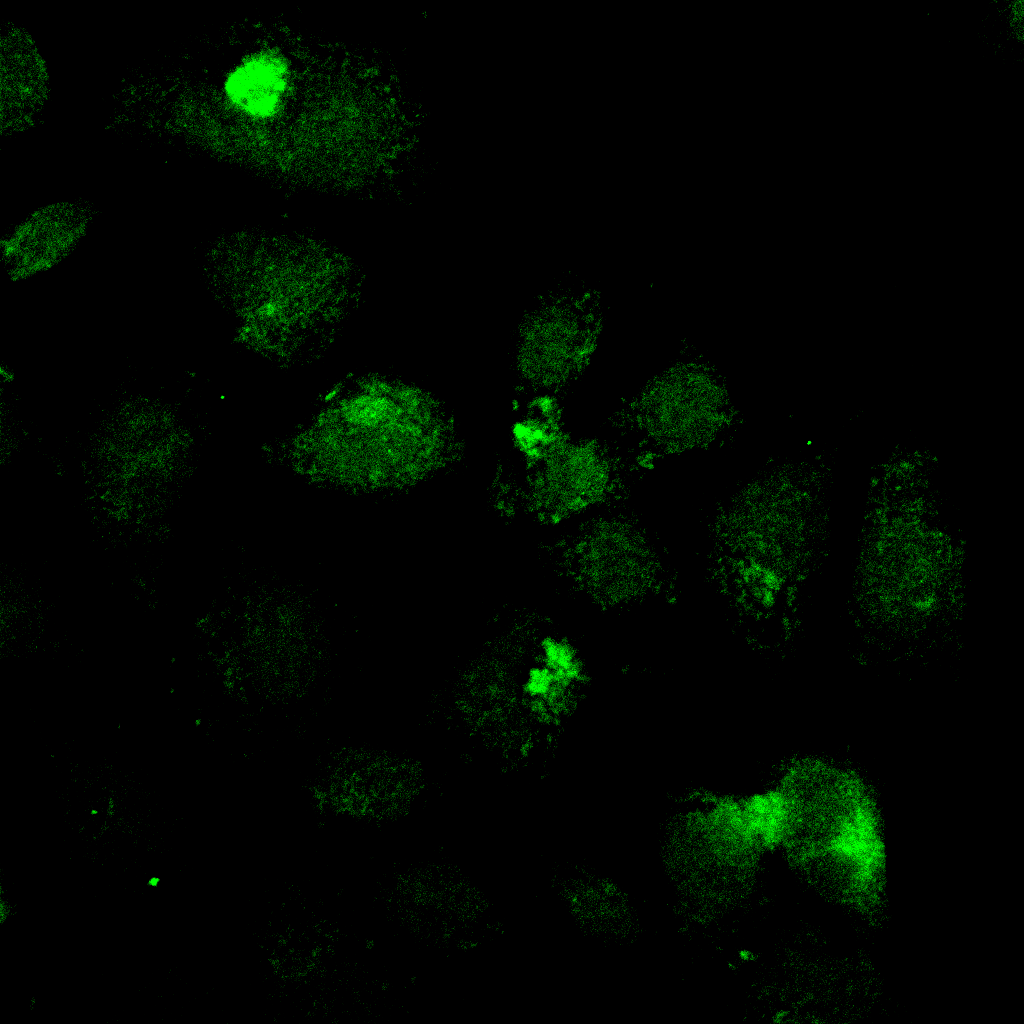

Supplement: Supplementary file 6 — Source data Fig. 2 [file 44318_2024_244_MOESM6_ESM.zip › Figure 2/2B/DLD1 Zyxin-Flag Tet-on cGAMP STING.tif]

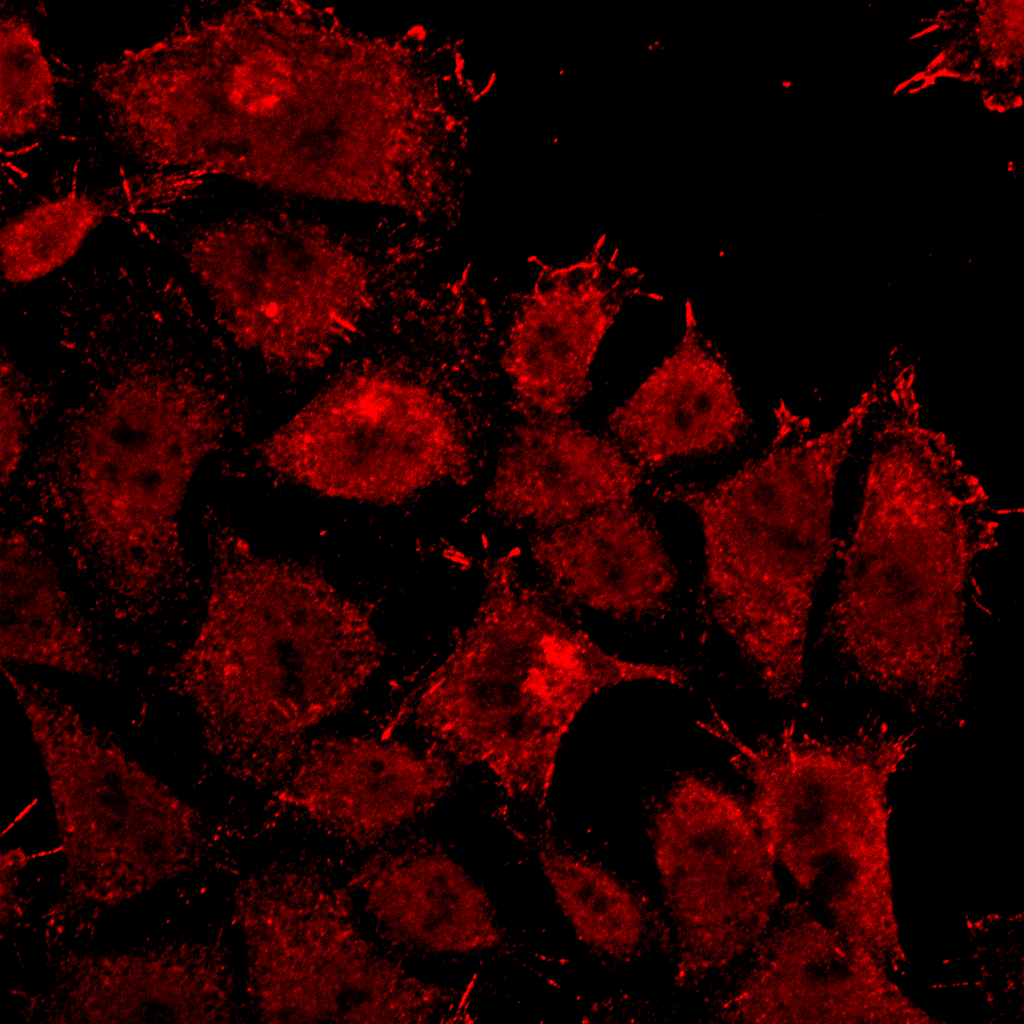

Supplement: Supplementary file 6 — Source data Fig. 2 [file 44318_2024_244_MOESM6_ESM.zip › Figure 2/2B/DLD1 Zyxin-Flag Tet-on cGAMP Zyxin-Flag.tif]

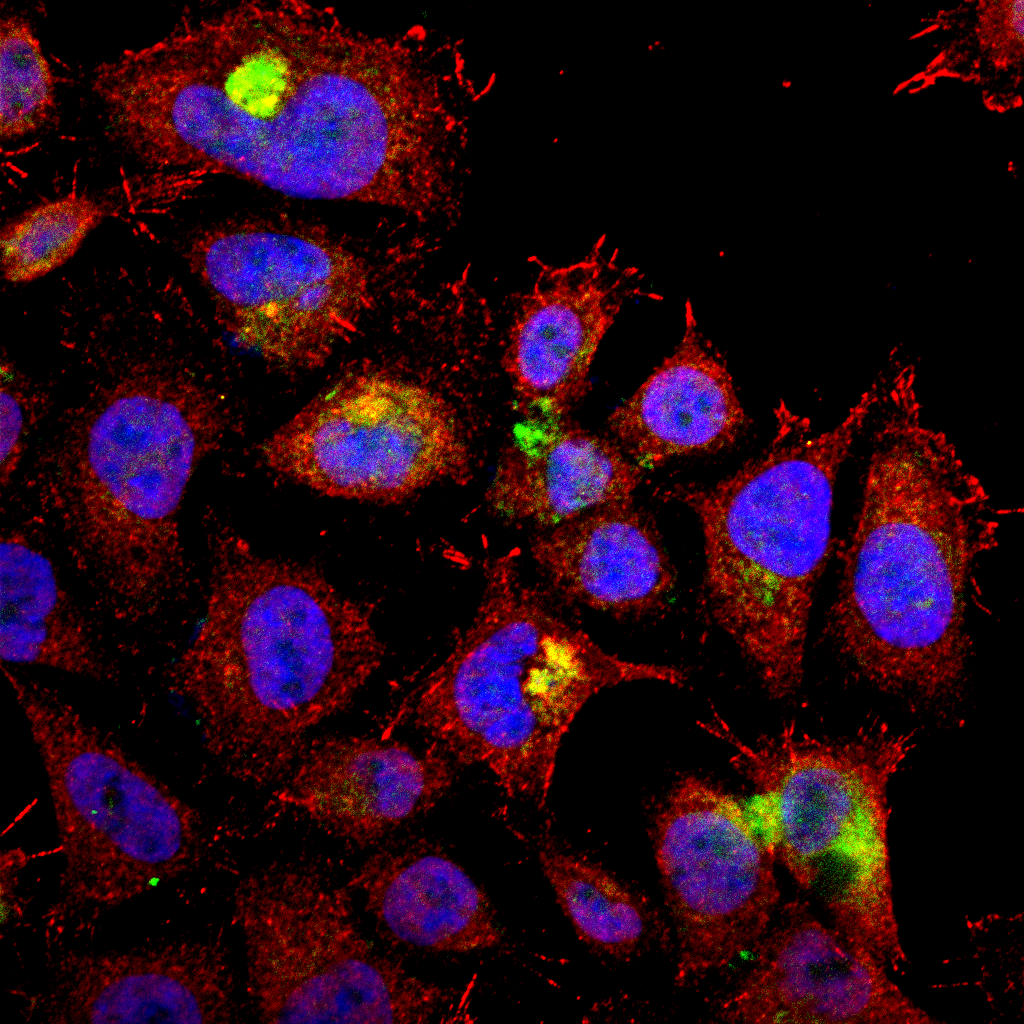

Supplement: Supplementary file 6 — Source data Fig. 2 [file 44318_2024_244_MOESM6_ESM.zip › Figure 2/2B/DLD1 Zyxin-Flag Tet-on cGAMP.tif]

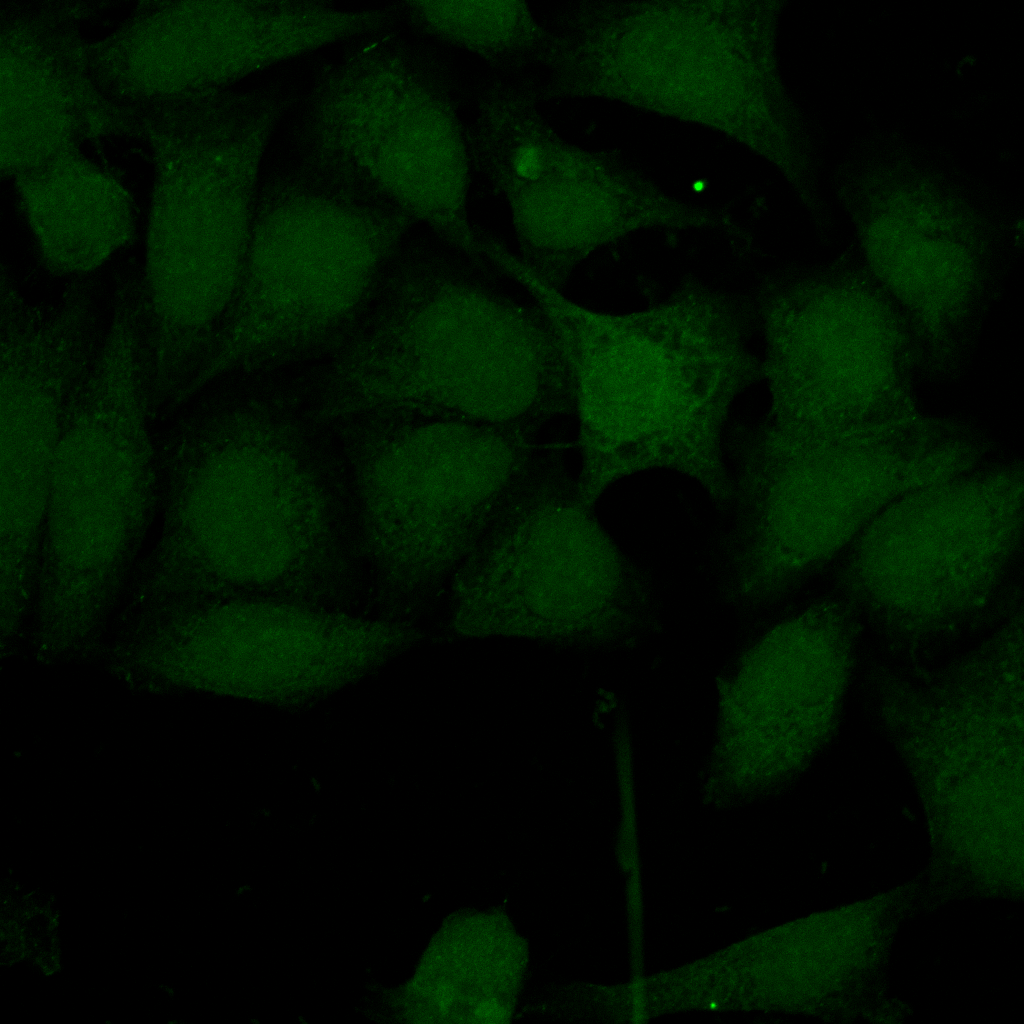

Supplement: Supplementary file 6 — Source data Fig. 2 [file 44318_2024_244_MOESM6_ESM.zip › Figure 2/2B/DLD1 Zyxin-Flag Tet-on Vehicle STING.tif]

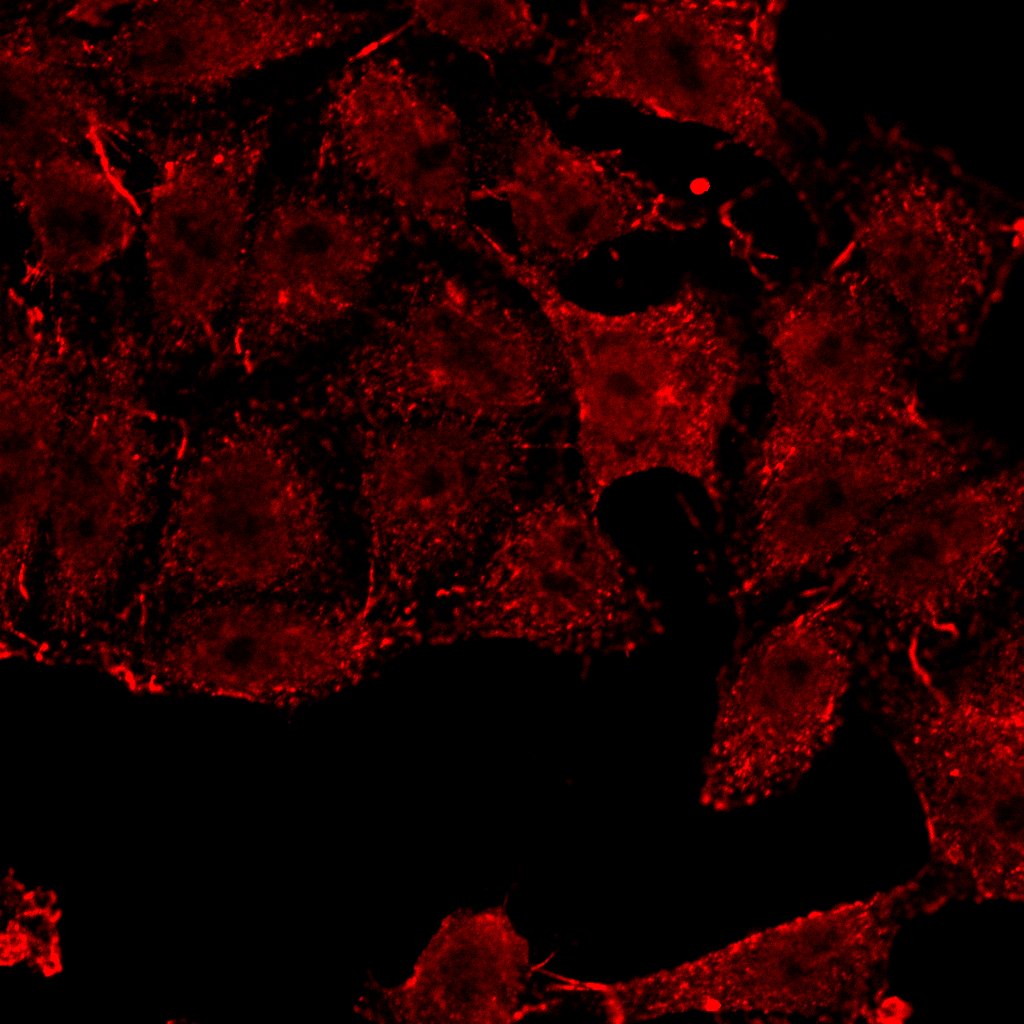

Supplement: Supplementary file 6 — Source data Fig. 2 [file 44318_2024_244_MOESM6_ESM.zip › Figure 2/2B/DLD1 Zyxin-Flag Tet-on Vehicle Zyxin-Flag.tif]

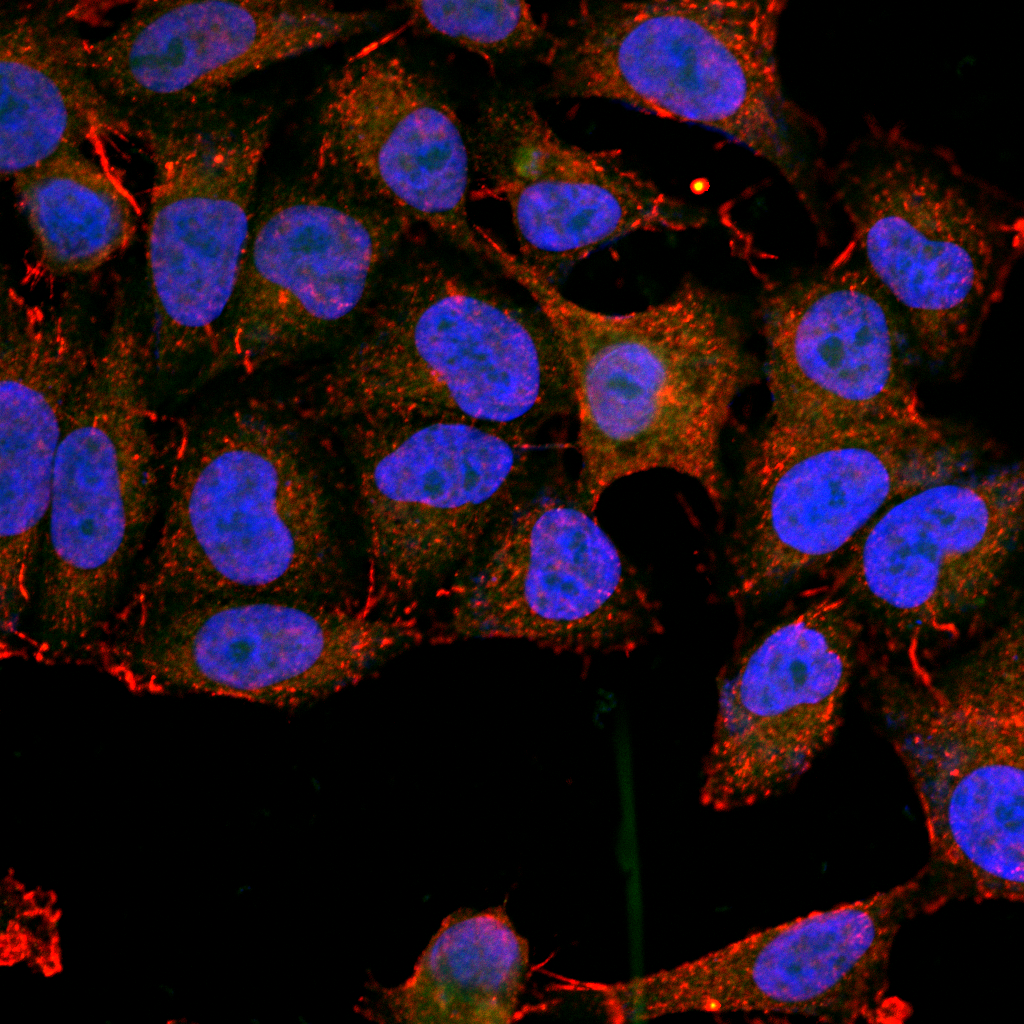

Supplement: Supplementary file 6 — Source data Fig. 2 [file 44318_2024_244_MOESM6_ESM.zip › Figure 2/2B/DLD1 Zyxin-Flag Tet-on Vehicle.tif]

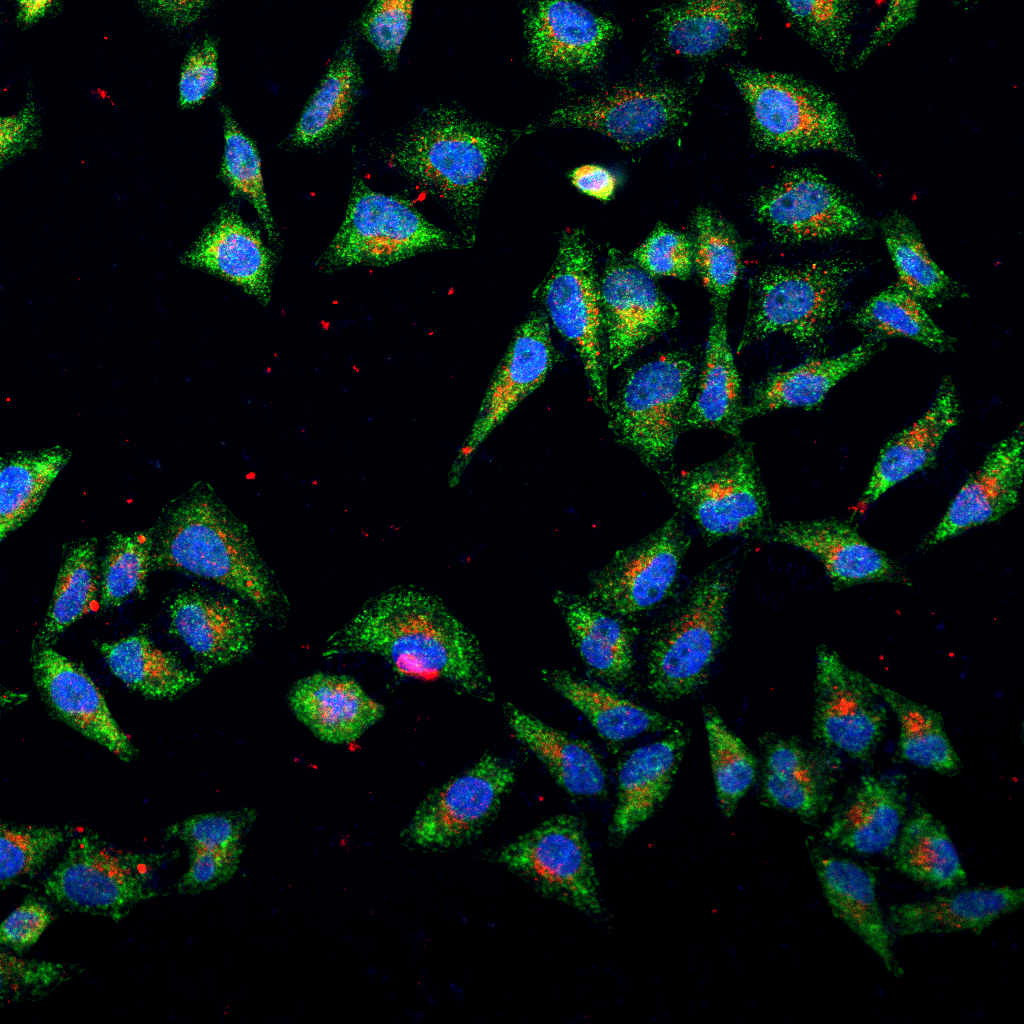

Supplement: Supplementary file 6 — Source data Fig. 2 [file 44318_2024_244_MOESM6_ESM.zip › Figure 2/2C/HeLa STING-HA Tet-on diAZBI 1 h Merge.tif]

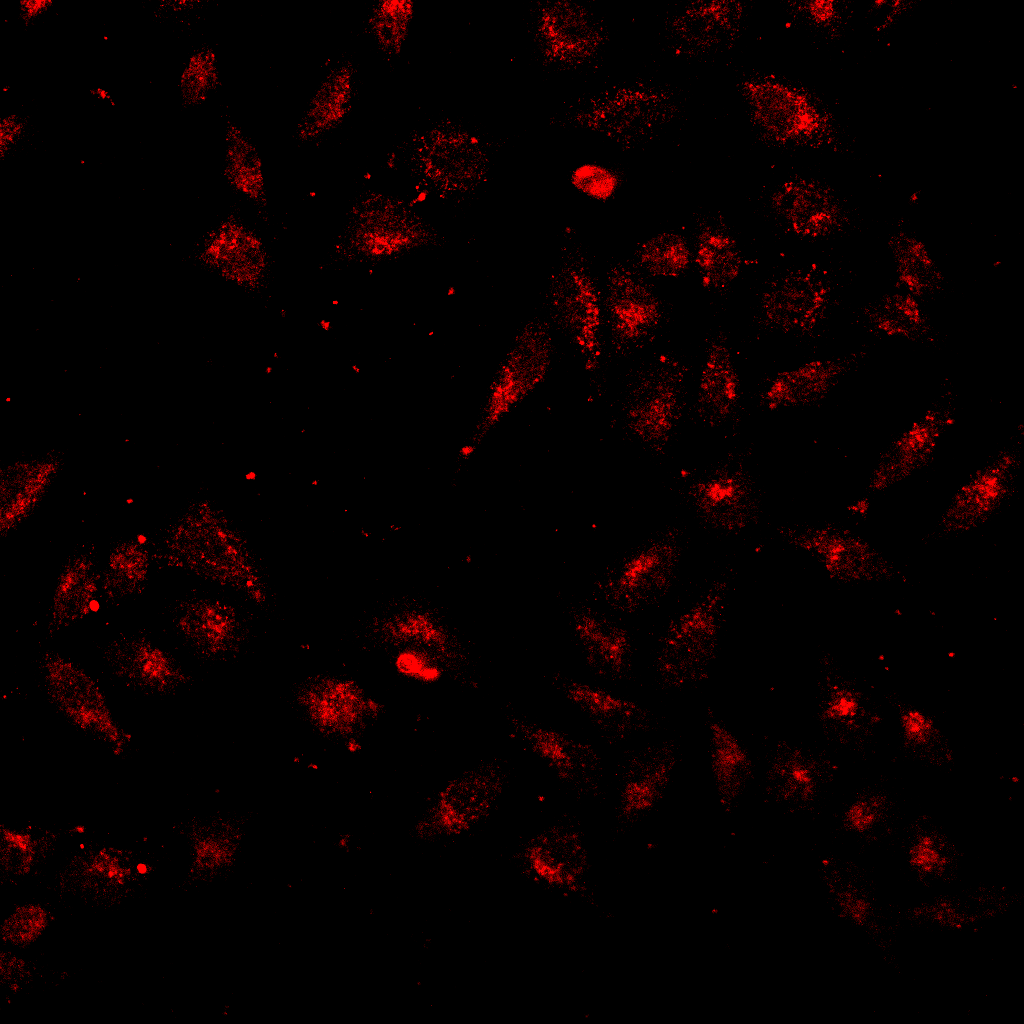

Supplement: Supplementary file 6 — Source data Fig. 2 [file 44318_2024_244_MOESM6_ESM.zip › Figure 2/2C/HeLa STING-HA Tet-on diAZBI 1 h STING.tif]

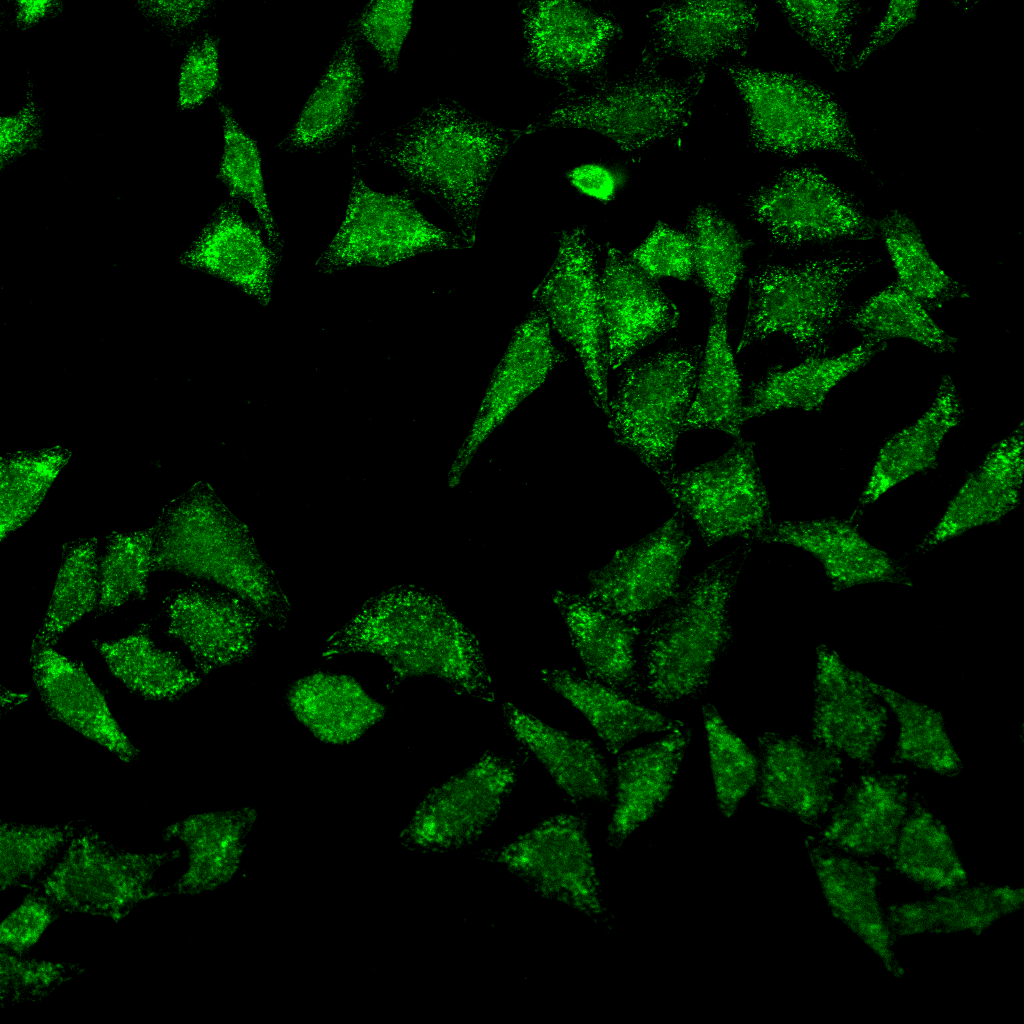

Supplement: Supplementary file 6 — Source data Fig. 2 [file 44318_2024_244_MOESM6_ESM.zip › Figure 2/2C/HeLa STING-HA Tet-on diAZBI 1 h Zyxin.tif]

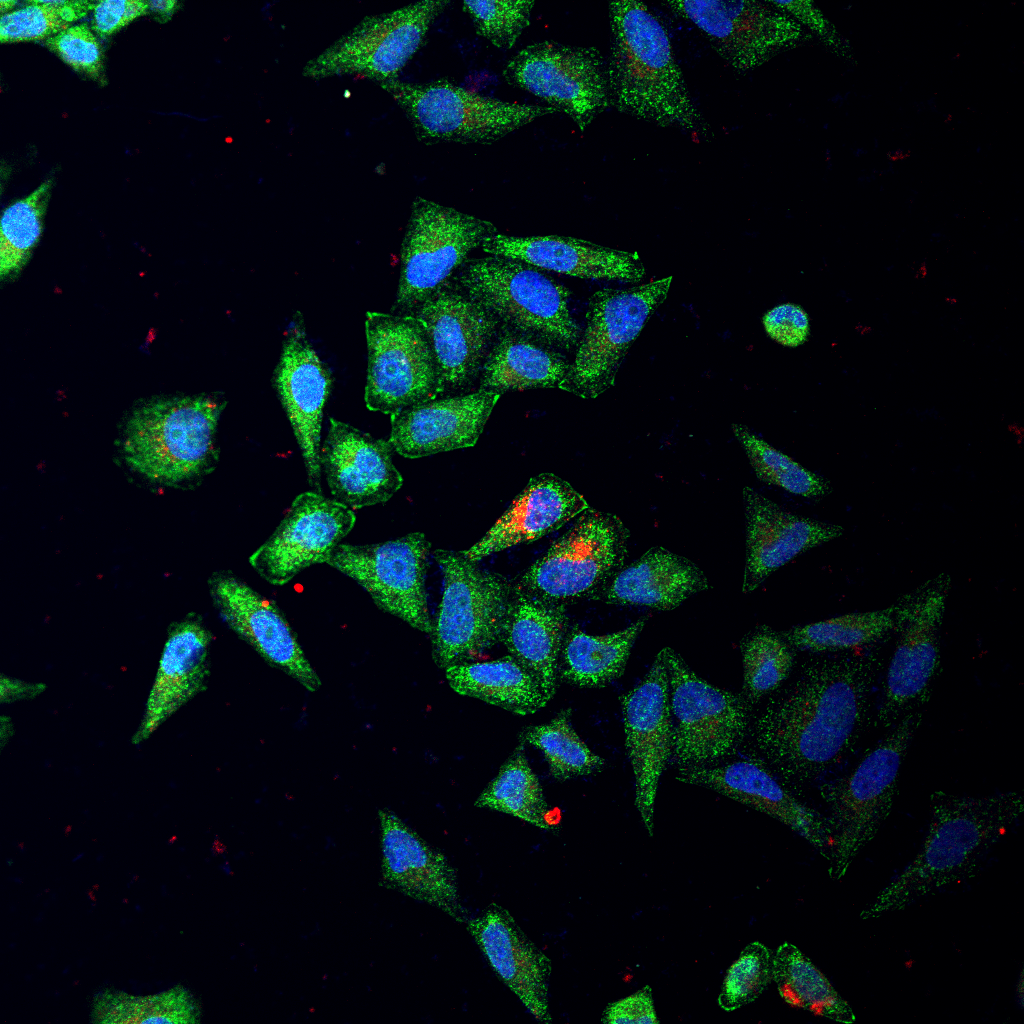

Supplement: Supplementary file 6 — Source data Fig. 2 [file 44318_2024_244_MOESM6_ESM.zip › Figure 2/2C/HeLa STING-HA Tet-on diAZBI 2 h Merge.tif]

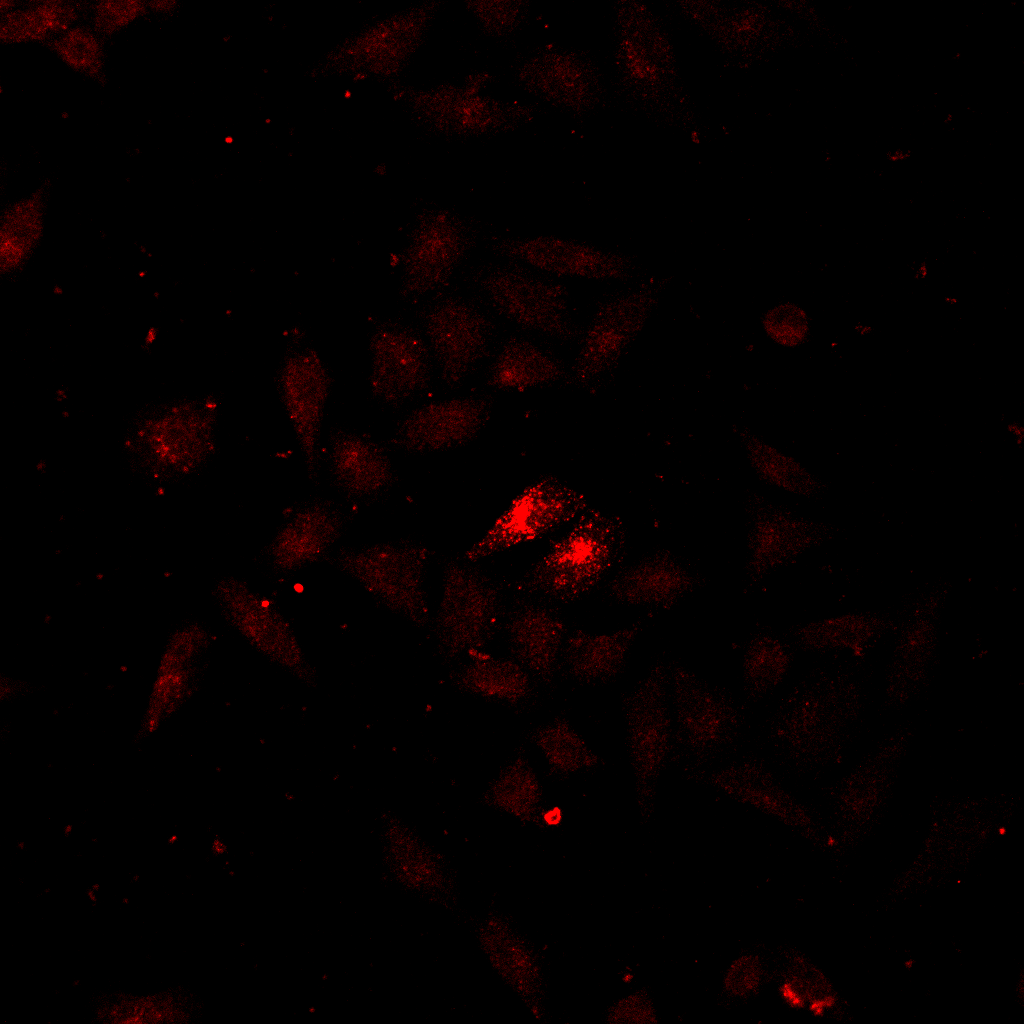

Supplement: Supplementary file 6 — Source data Fig. 2 [file 44318_2024_244_MOESM6_ESM.zip › Figure 2/2C/HeLa STING-HA Tet-on diAZBI 2 h STING.tif]

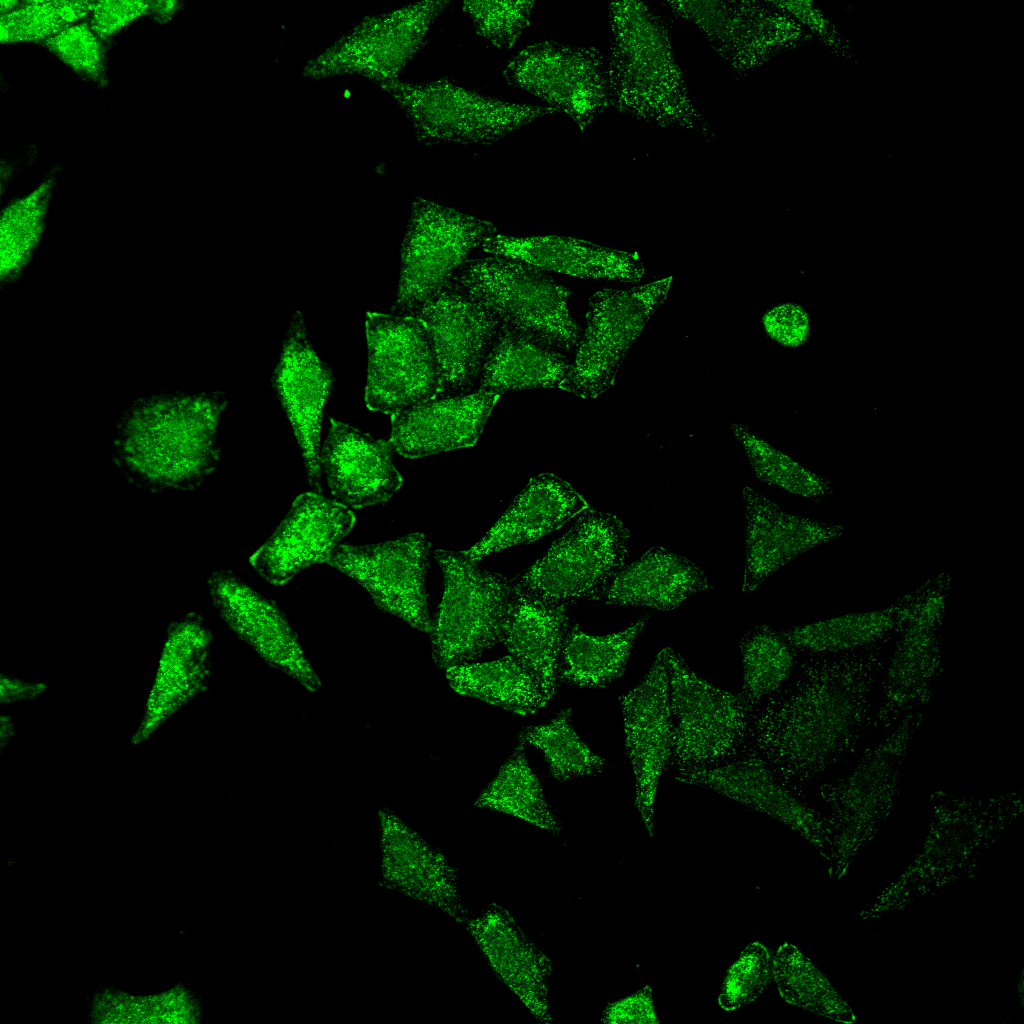

Supplement: Supplementary file 6 — Source data Fig. 2 [file 44318_2024_244_MOESM6_ESM.zip › Figure 2/2C/HeLa STING-HA Tet-on diAZBI 2 h Zyxin.tif]

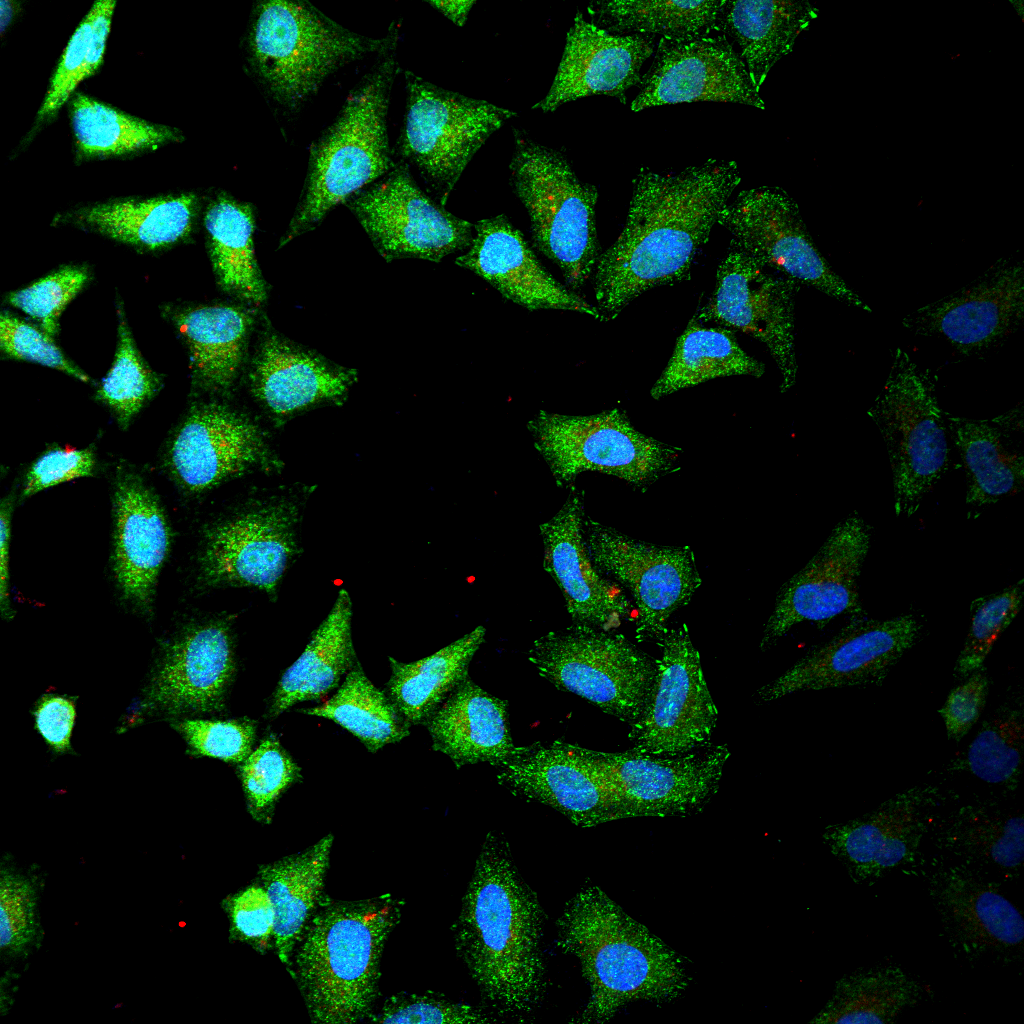

Supplement: Supplementary file 6 — Source data Fig. 2 [file 44318_2024_244_MOESM6_ESM.zip › Figure 2/2C/HeLa STING-HA Tet-on diAZBI 4 h Merge.tif]

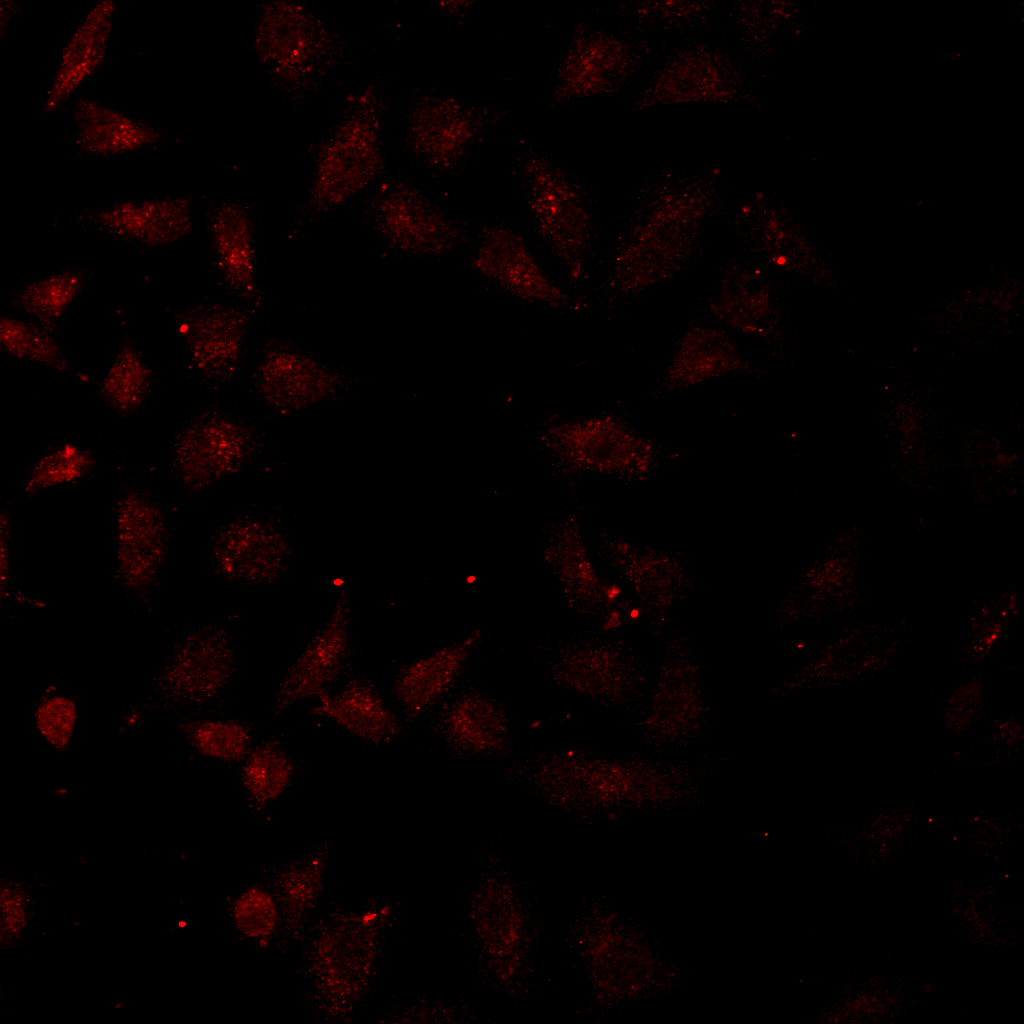

Supplement: Supplementary file 6 — Source data Fig. 2 [file 44318_2024_244_MOESM6_ESM.zip › Figure 2/2C/HeLa STING-HA Tet-on diAZBI 4 h STING.tif]

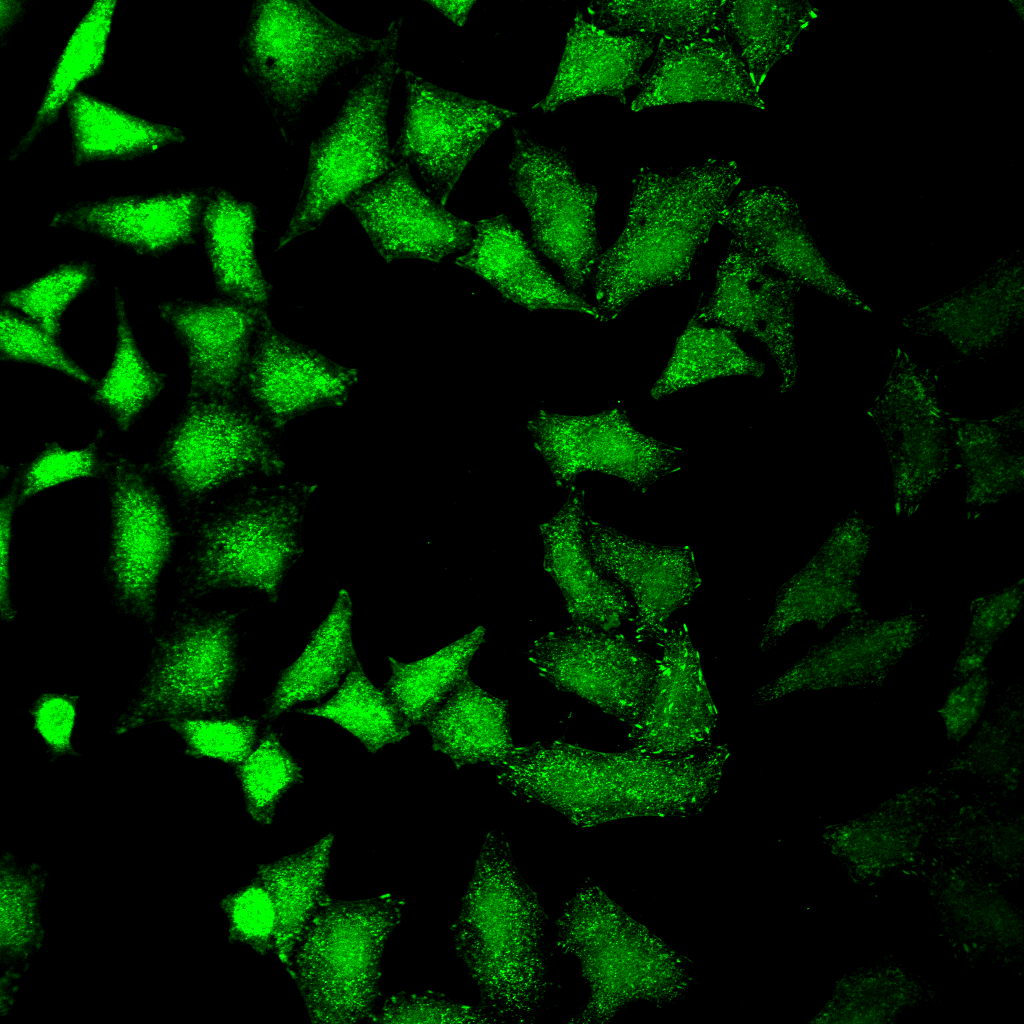

Supplement: Supplementary file 6 — Source data Fig. 2 [file 44318_2024_244_MOESM6_ESM.zip › Figure 2/2C/HeLa STING-HA Tet-on diAZBI 4 h Zyxin.tif]

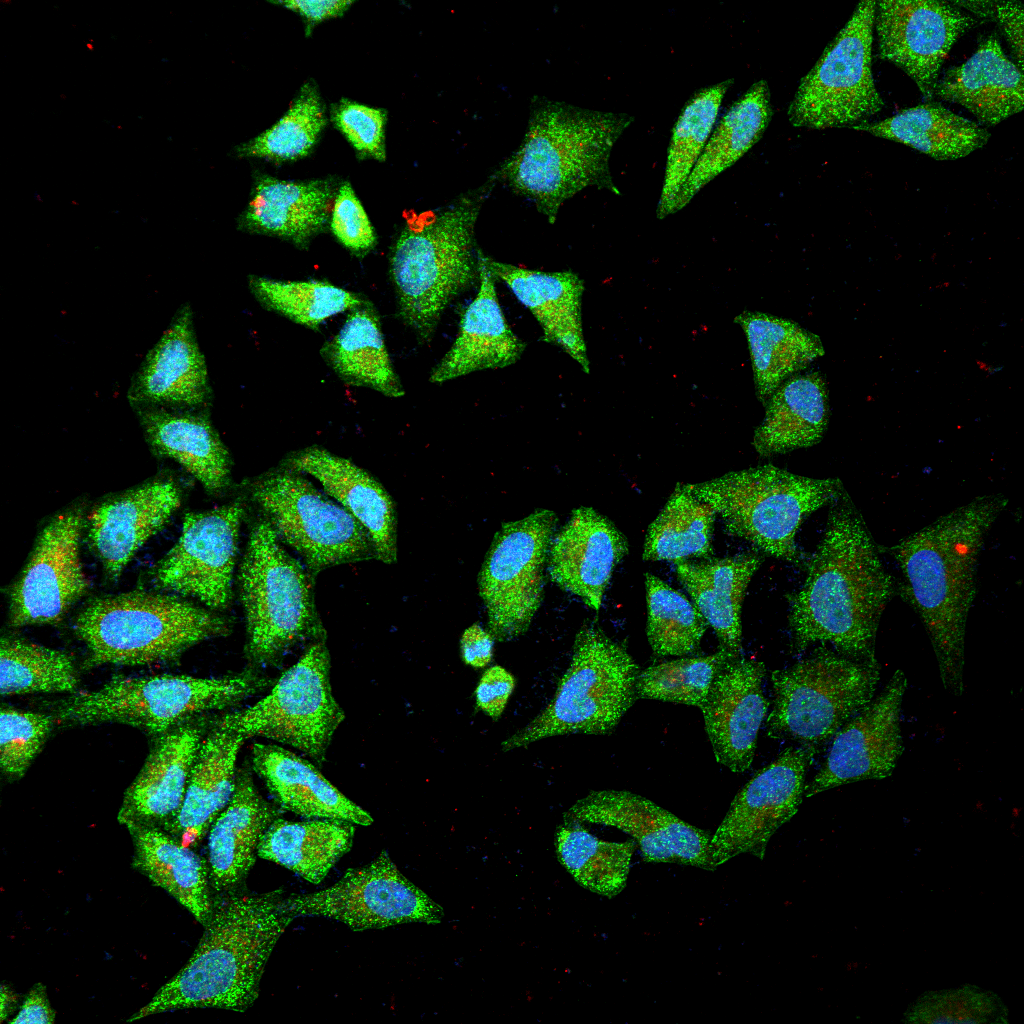

Supplement: Supplementary file 6 — Source data Fig. 2 [file 44318_2024_244_MOESM6_ESM.zip › Figure 2/2C/HeLa STING-HA Tet-on Vehicle Merge.tif]

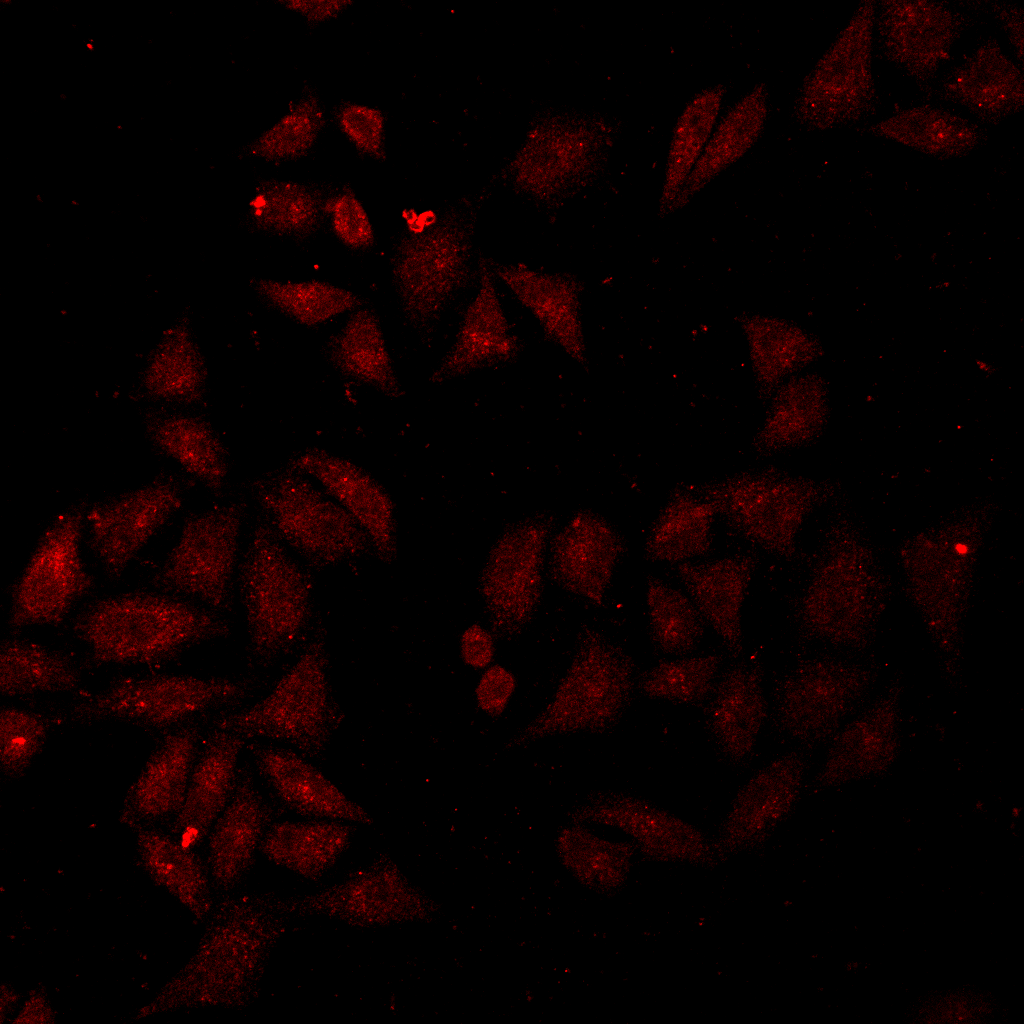

Supplement: Supplementary file 6 — Source data Fig. 2 [file 44318_2024_244_MOESM6_ESM.zip › Figure 2/2C/HeLa STING-HA Tet-on Vehicle STING.tif]

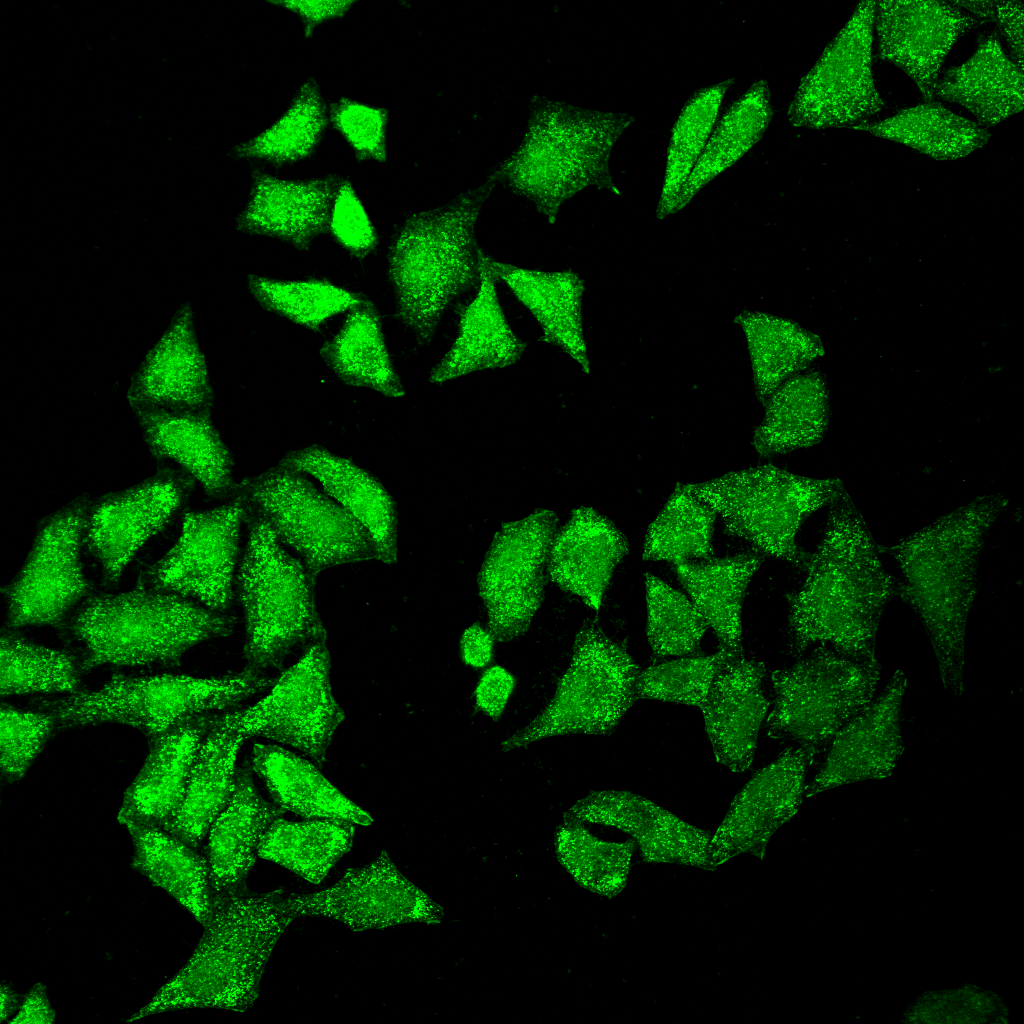

Supplement: Supplementary file 6 — Source data Fig. 2 [file 44318_2024_244_MOESM6_ESM.zip › Figure 2/2C/HeLa STING-HA Tet-on Vehicle Zyxin.tif]

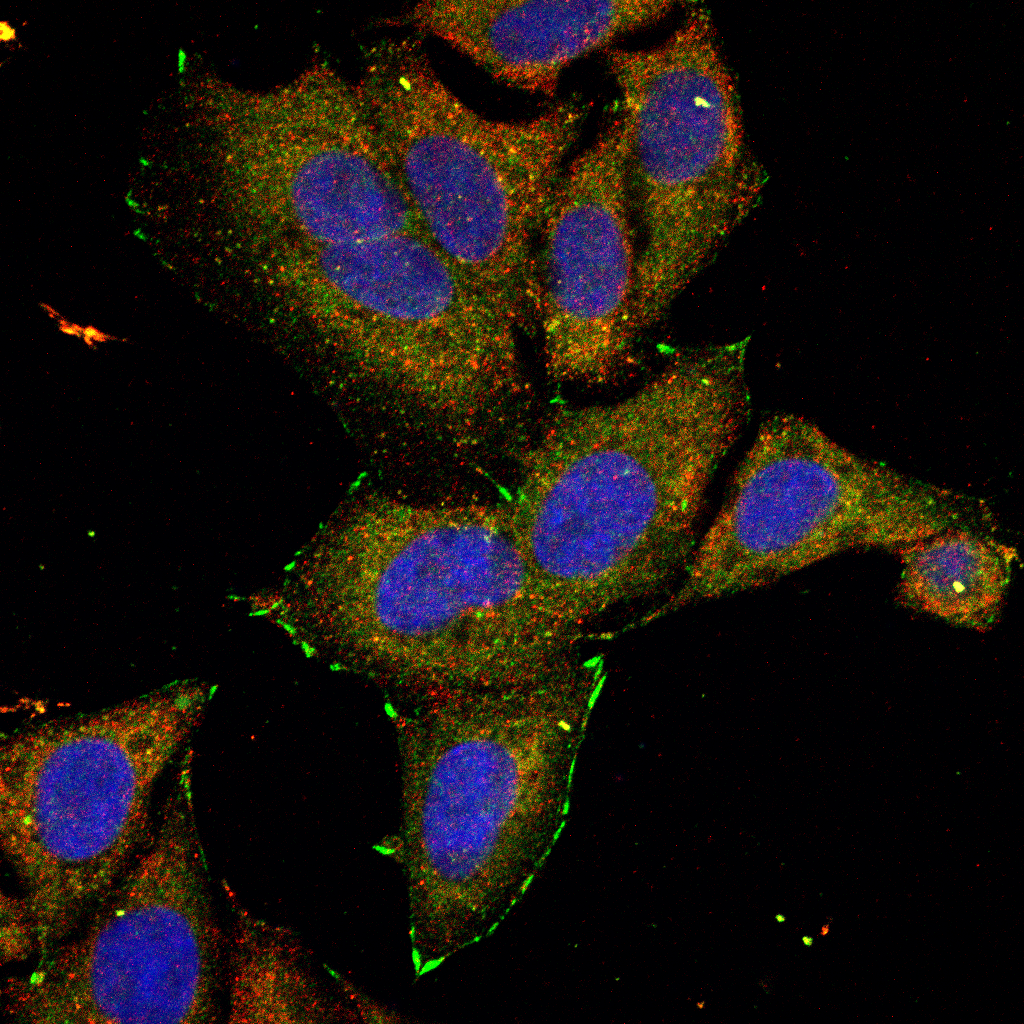

Supplement: Supplementary file 6 — Source data Fig. 2 [file 44318_2024_244_MOESM6_ESM.zip › Figure 2/2E/DLD1 TBK1-HA Tet-on poly (I C) Merge.tif]

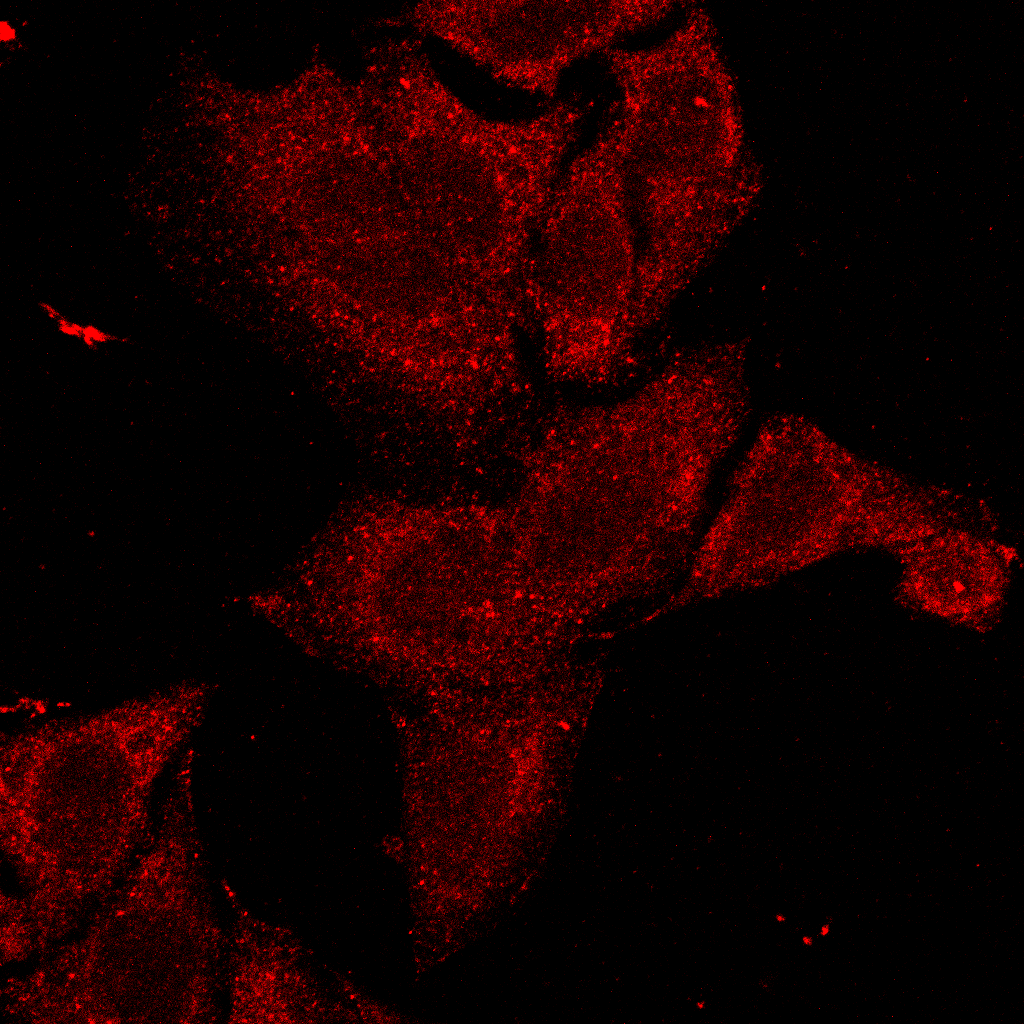

Supplement: Supplementary file 6 — Source data Fig. 2 [file 44318_2024_244_MOESM6_ESM.zip › Figure 2/2E/DLD1 TBK1-HA Tet-on poly (I C) TBK1-HA.tif]

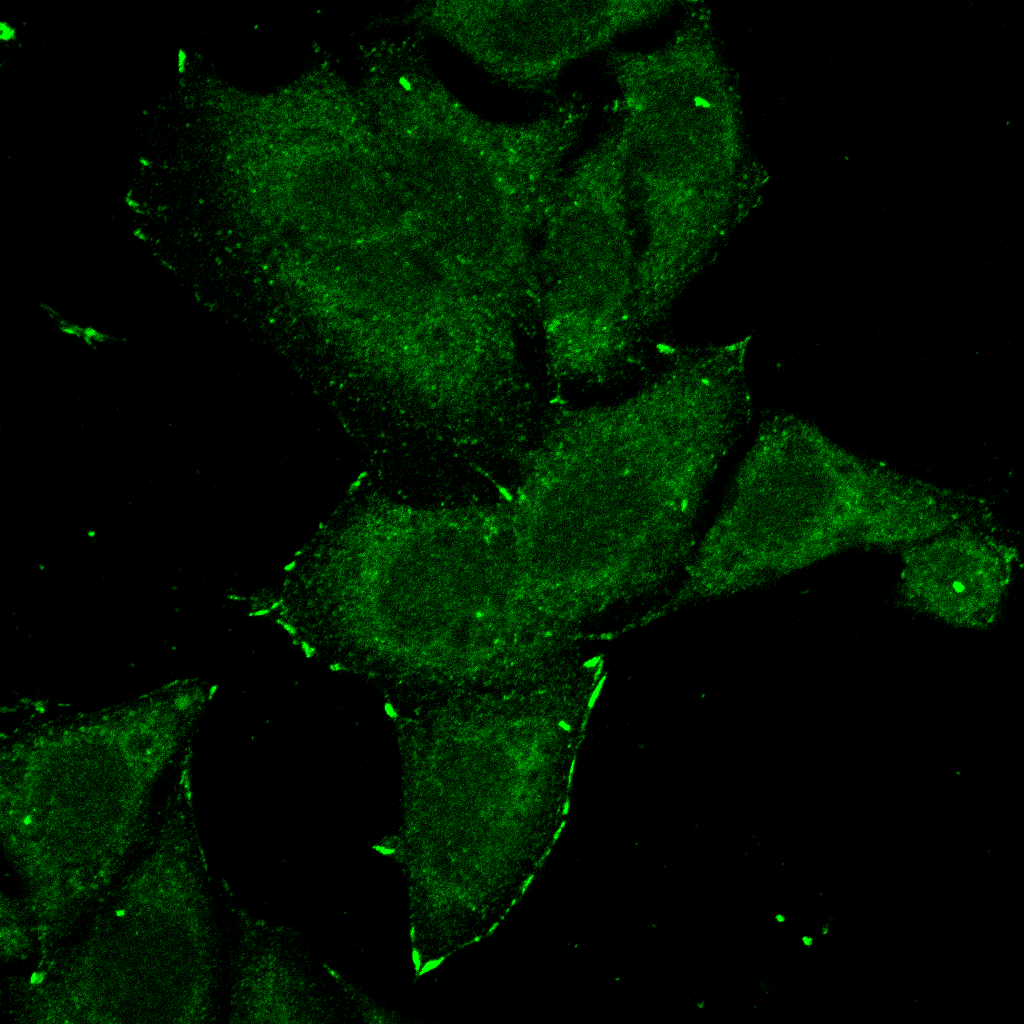

Supplement: Supplementary file 6 — Source data Fig. 2 [file 44318_2024_244_MOESM6_ESM.zip › Figure 2/2E/DLD1 TBK1-HA Tet-on poly (I C) Zyxin.tif]

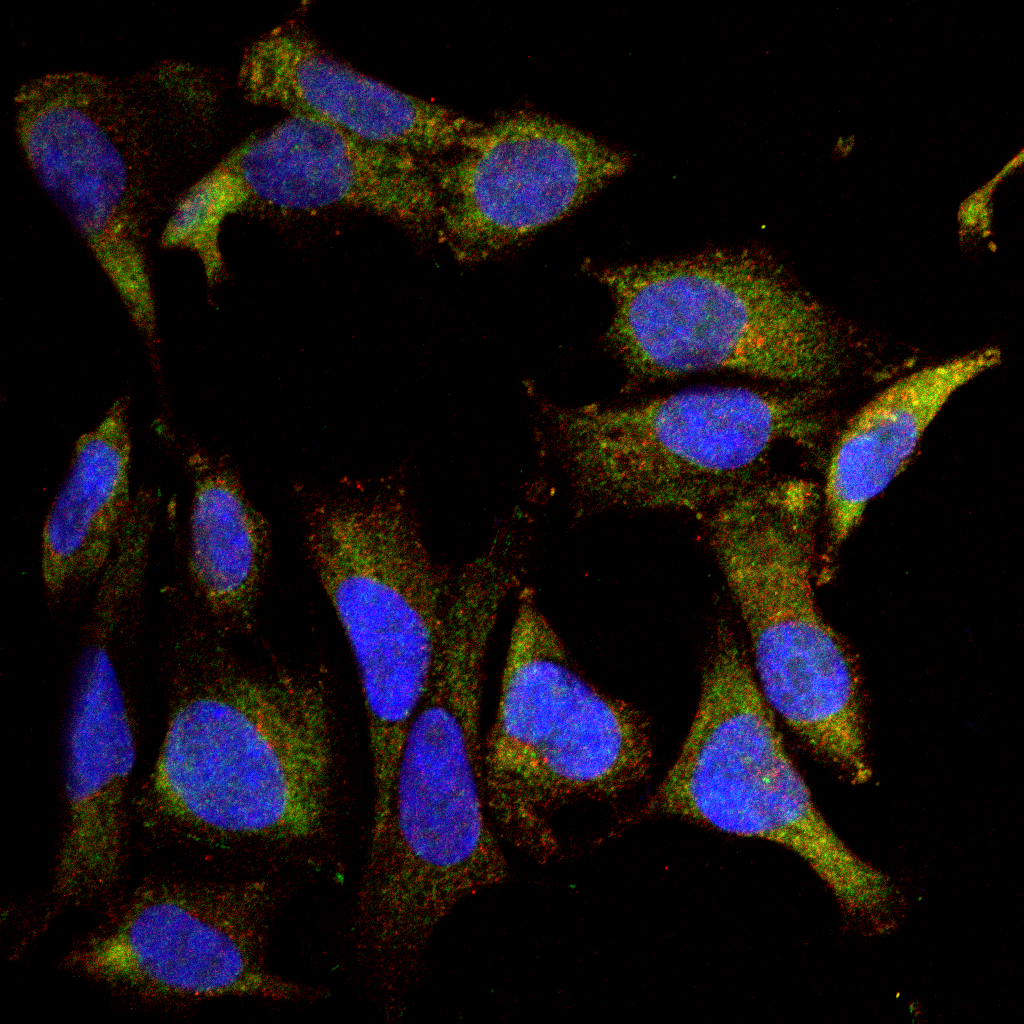

Supplement: Supplementary file 6 — Source data Fig. 2 [file 44318_2024_244_MOESM6_ESM.zip › Figure 2/2E/DLD1 TBK1-HA Tet-on Vehicle Merge.tif]

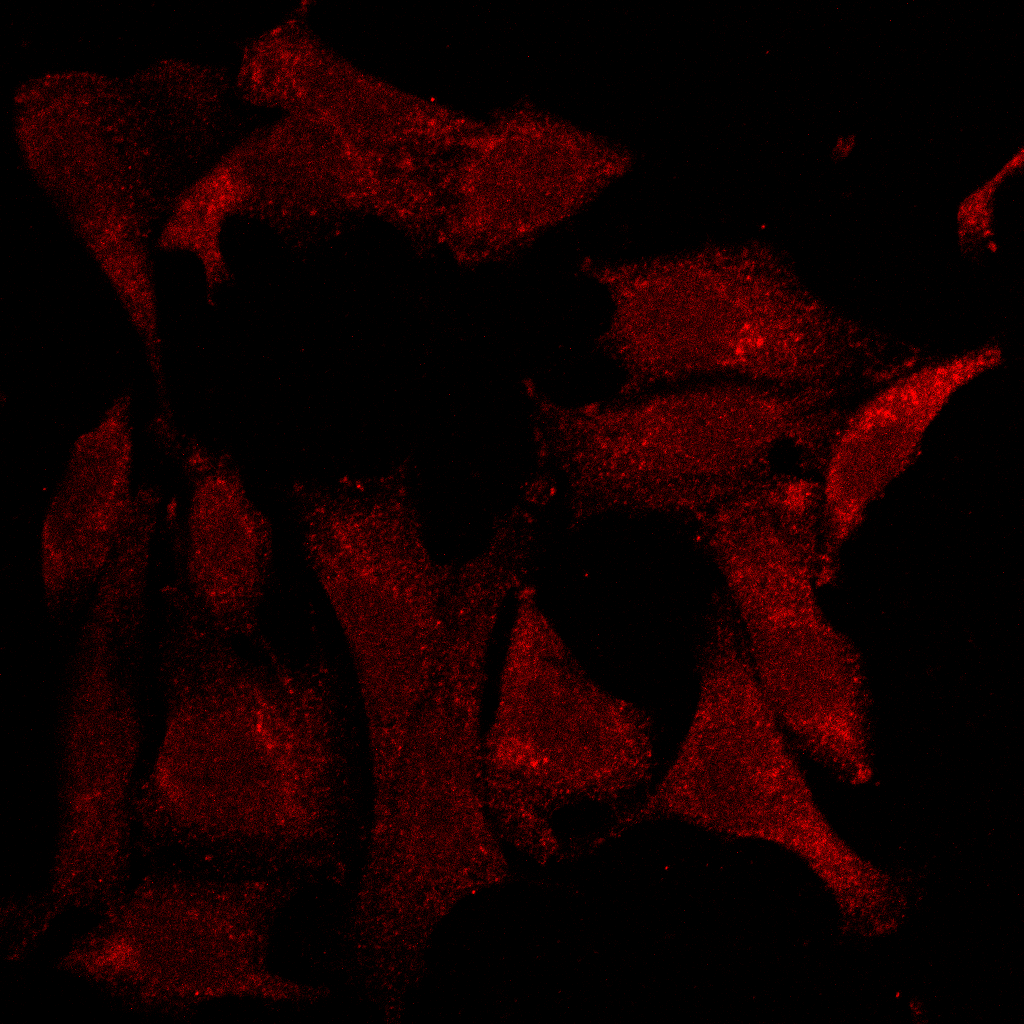

Supplement: Supplementary file 6 — Source data Fig. 2 [file 44318_2024_244_MOESM6_ESM.zip › Figure 2/2E/DLD1 TBK1-HA Tet-on Vehicle TBK1-HA.tif]

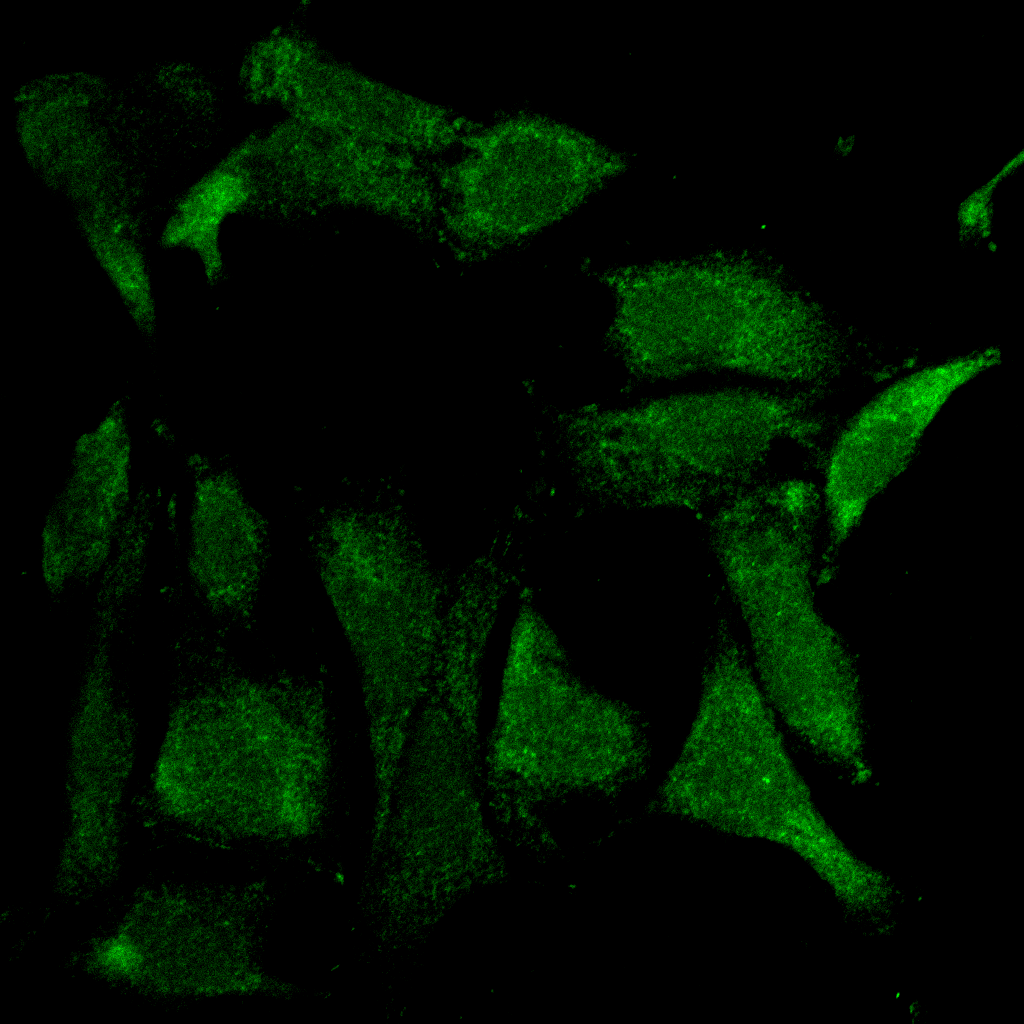

Supplement: Supplementary file 6 — Source data Fig. 2 [file 44318_2024_244_MOESM6_ESM.zip › Figure 2/2E/DLD1 TBK1-HA Tet-on Vehicle Zyxin.tif]

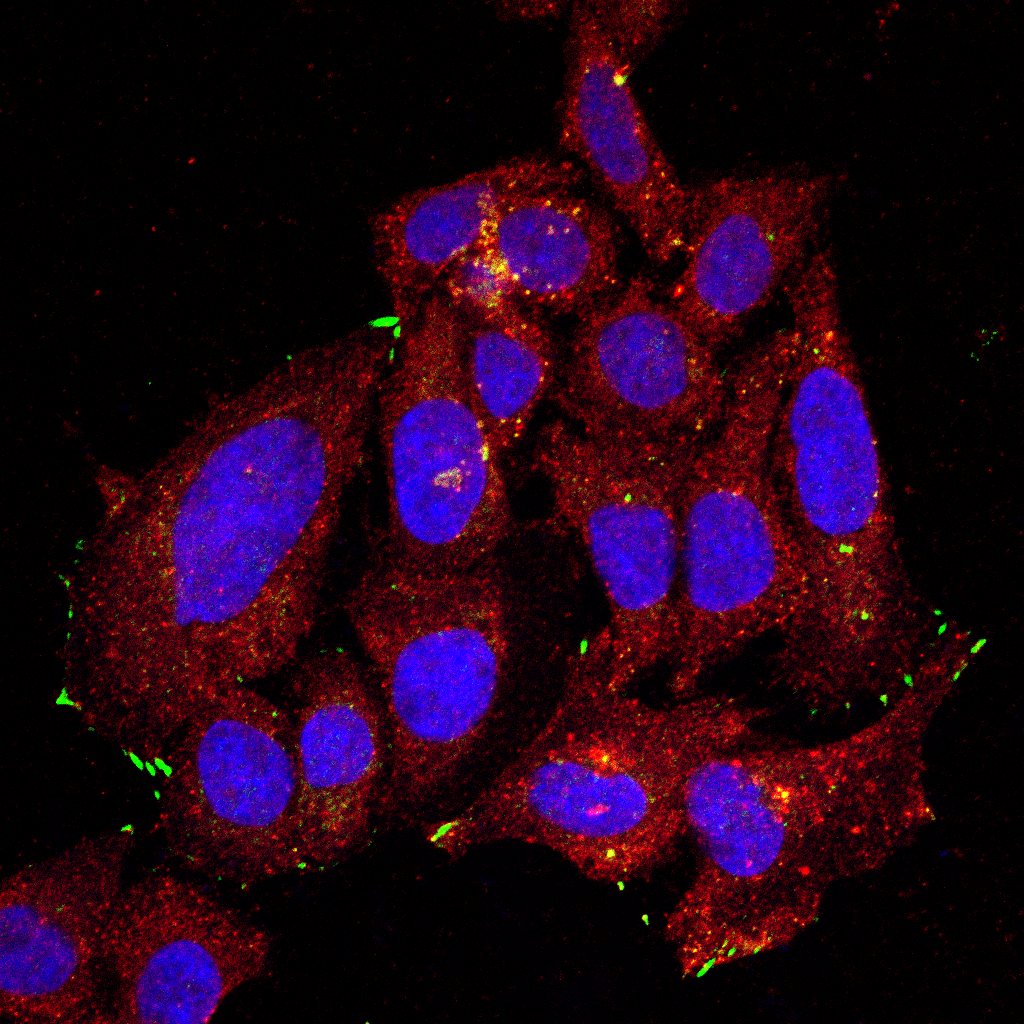

Supplement: Supplementary file 6 — Source data Fig. 2 [file 44318_2024_244_MOESM6_ESM.zip › Figure 2/2F/DLD1 TBK1-HA Tet-on poly (I C) Merge.tif]

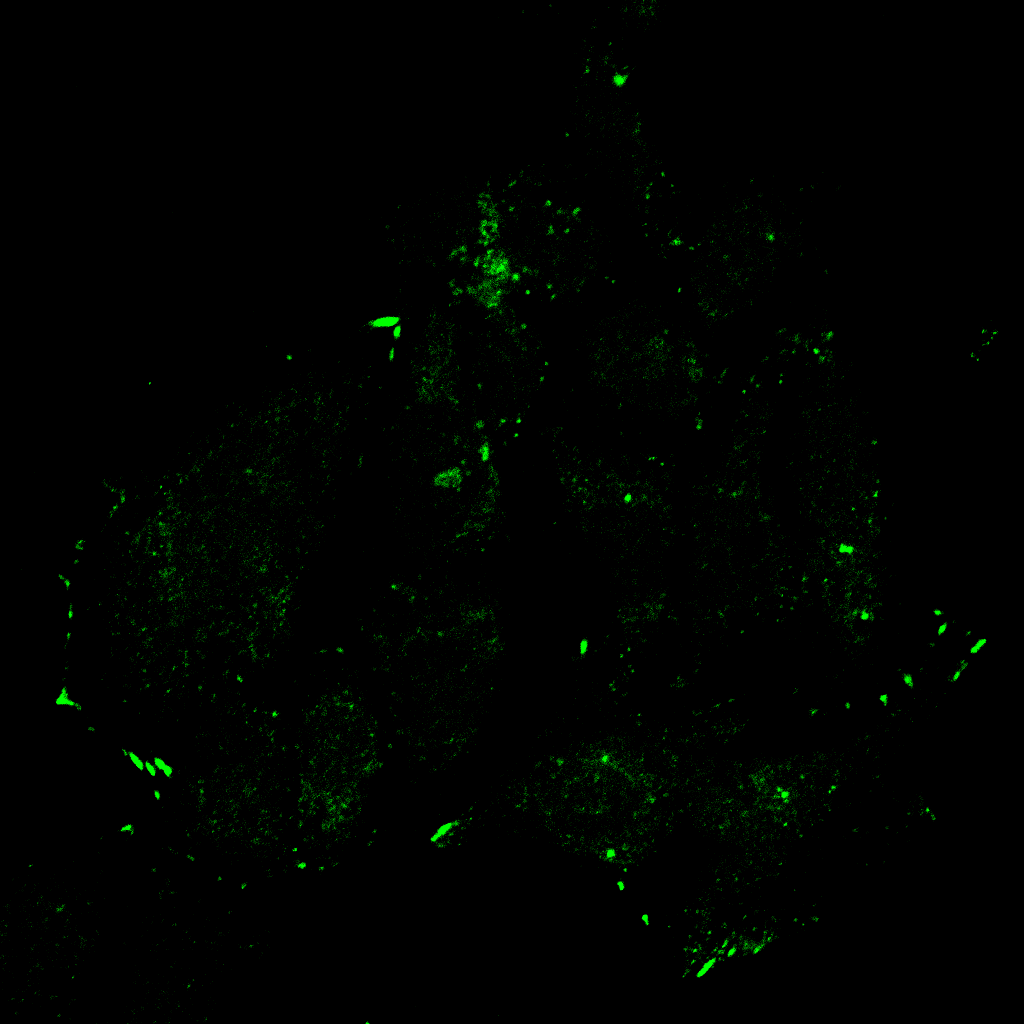

Supplement: Supplementary file 6 — Source data Fig. 2 [file 44318_2024_244_MOESM6_ESM.zip › Figure 2/2F/DLD1 TBK1-HA Tet-on poly (I C) pZyxin.tif]

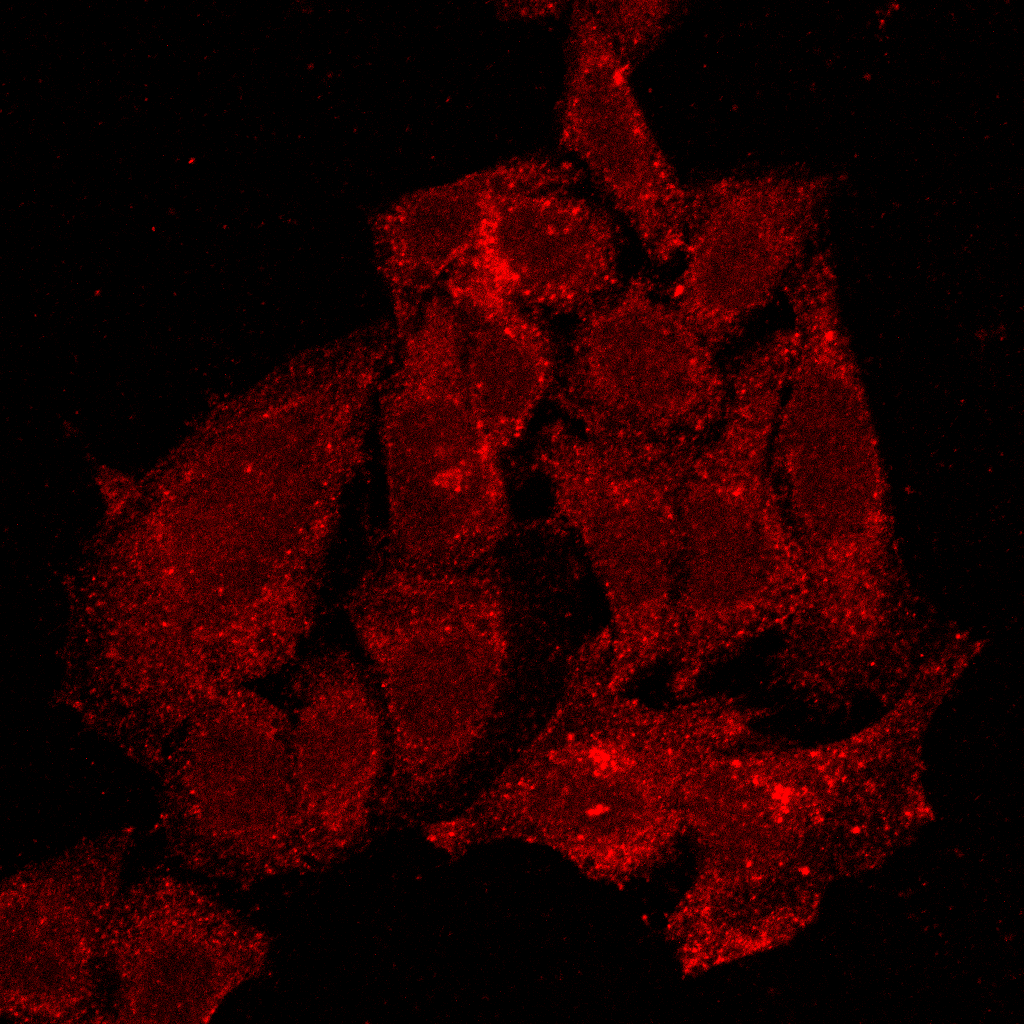

Supplement: Supplementary file 6 — Source data Fig. 2 [file 44318_2024_244_MOESM6_ESM.zip › Figure 2/2F/DLD1 TBK1-HA Tet-on poly (I C) TBK1-HA.tif]

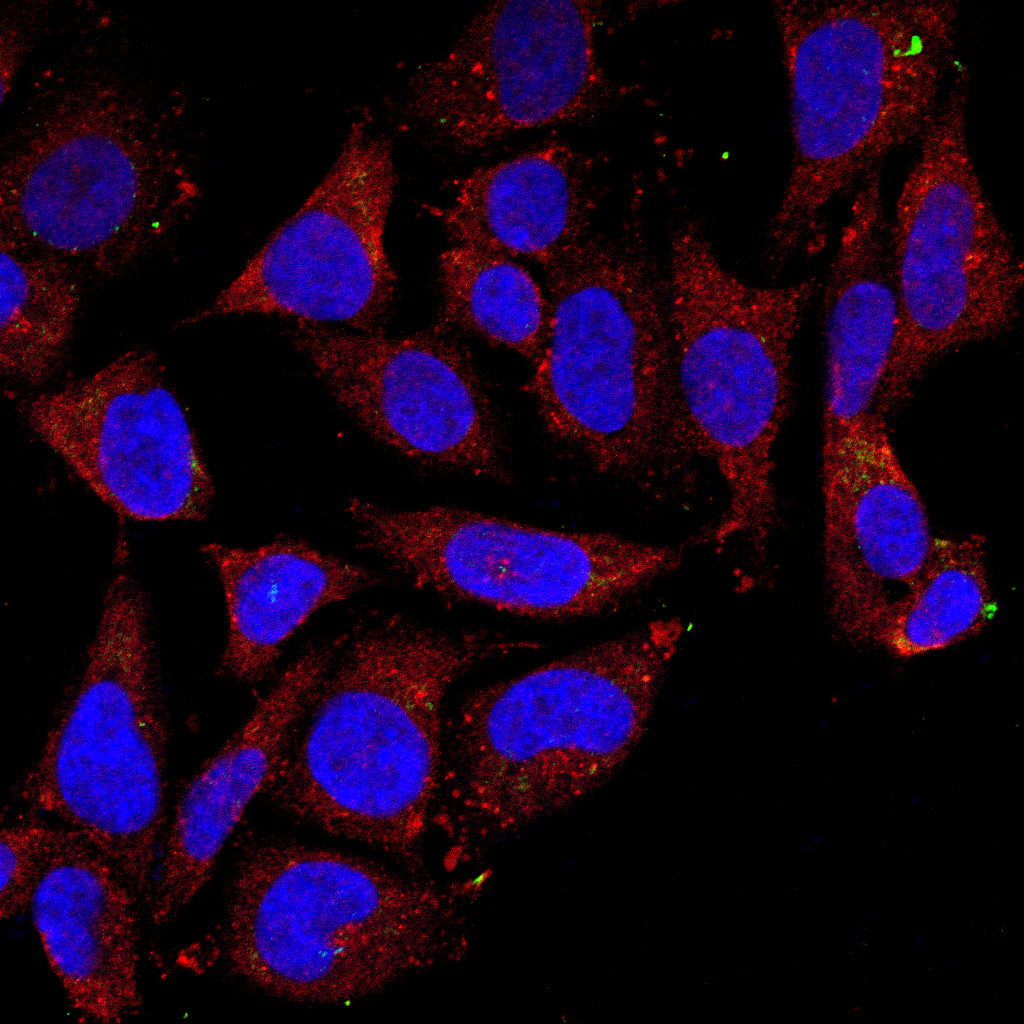

Supplement: Supplementary file 6 — Source data Fig. 2 [file 44318_2024_244_MOESM6_ESM.zip › Figure 2/2F/DLD1 TBK1-HA Tet-on Vehicle Merge.tif]

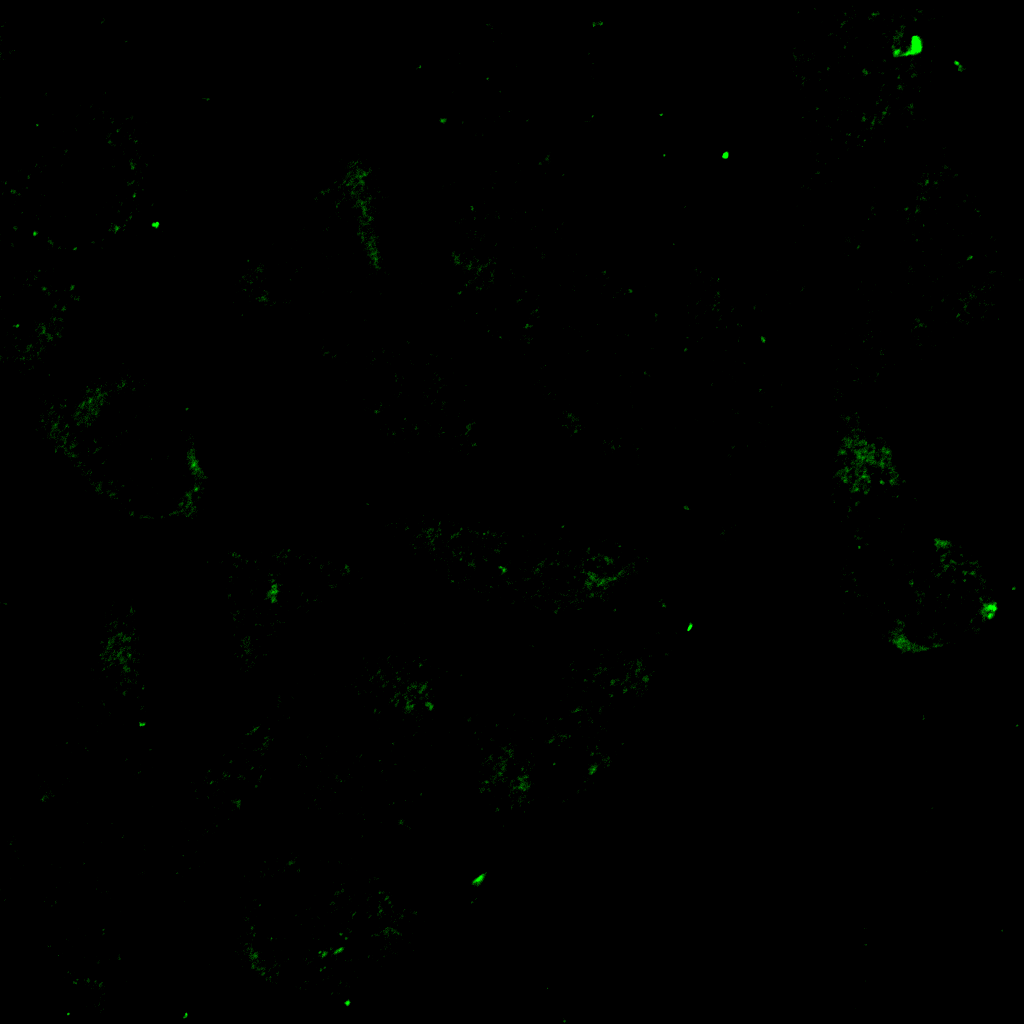

Supplement: Supplementary file 6 — Source data Fig. 2 [file 44318_2024_244_MOESM6_ESM.zip › Figure 2/2F/DLD1 TBK1-HA Tet-on Vehicle pZyxin.tif]

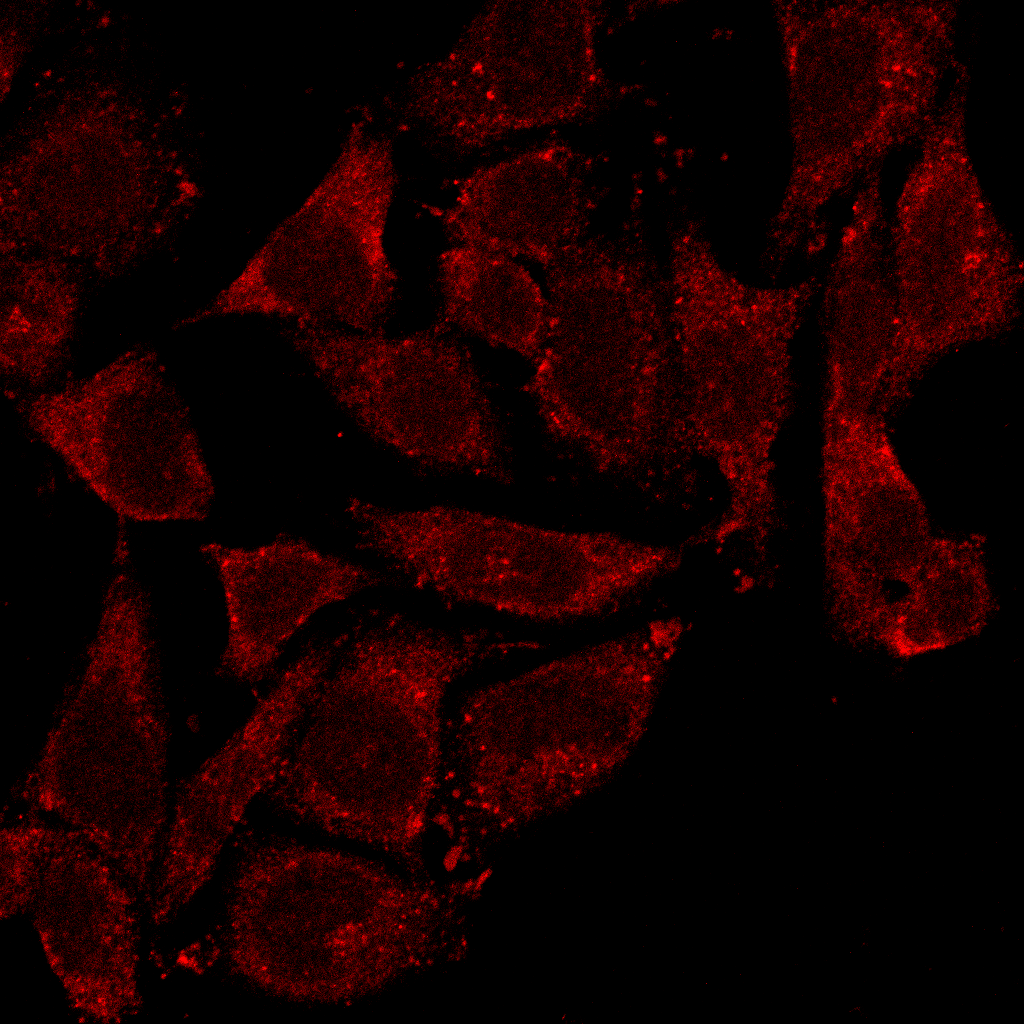

Supplement: Supplementary file 6 — Source data Fig. 2 [file 44318_2024_244_MOESM6_ESM.zip › Figure 2/2F/DLD1 TBK1-HA Tet-on Vehicle TBK1-HA.tif]

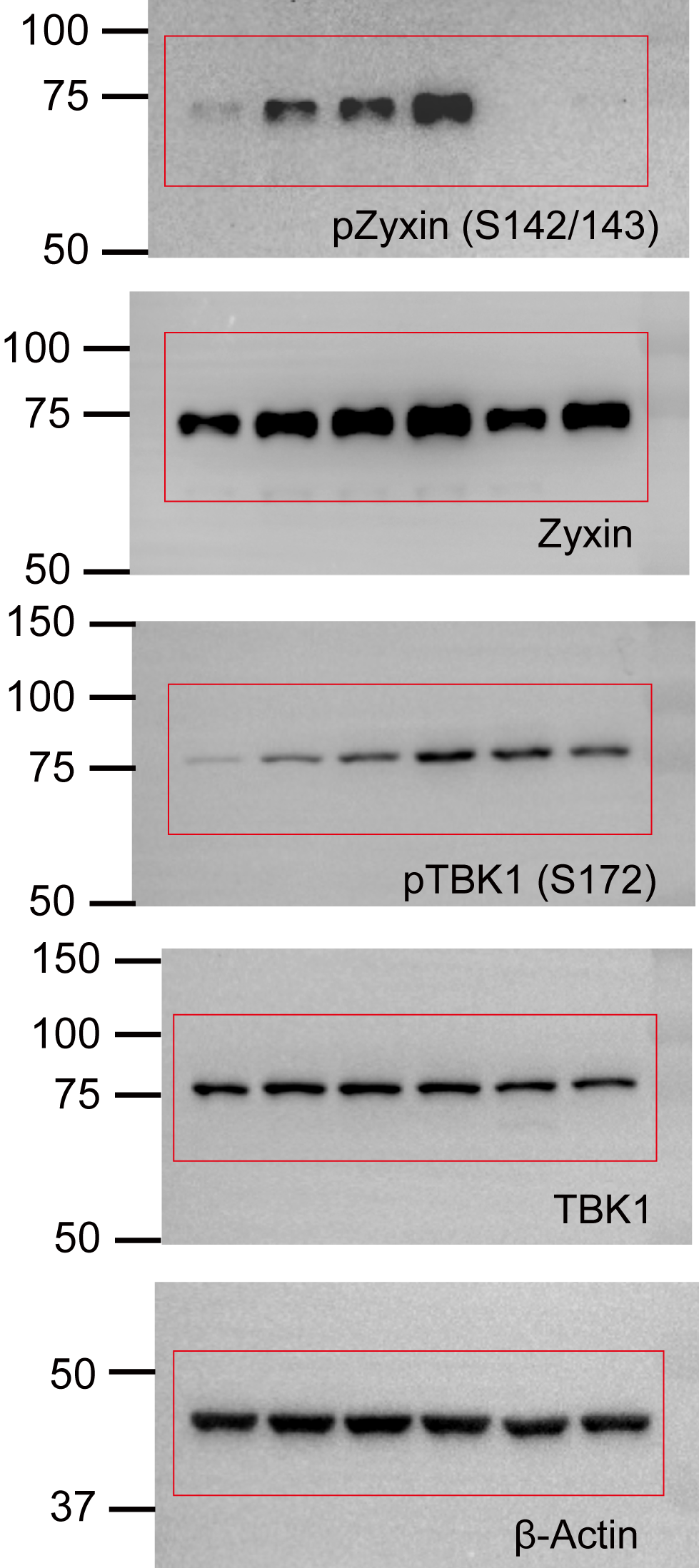

Supplement: Supplementary file 6 — Source data Fig. 2 [file 44318_2024_244_MOESM6_ESM.zip › Figure 2/2G/2J.tif]

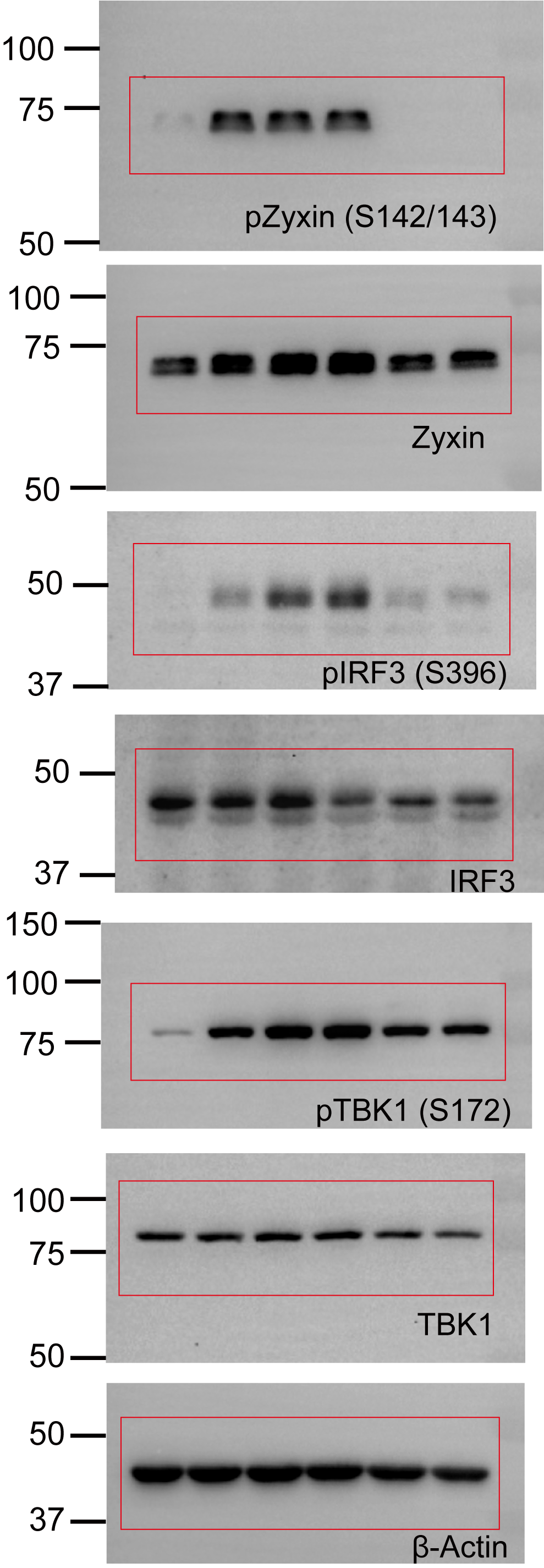

Supplement: Supplementary file 6 — Source data Fig. 2 [file 44318_2024_244_MOESM6_ESM.zip › Figure 2/2H/2K.tif]

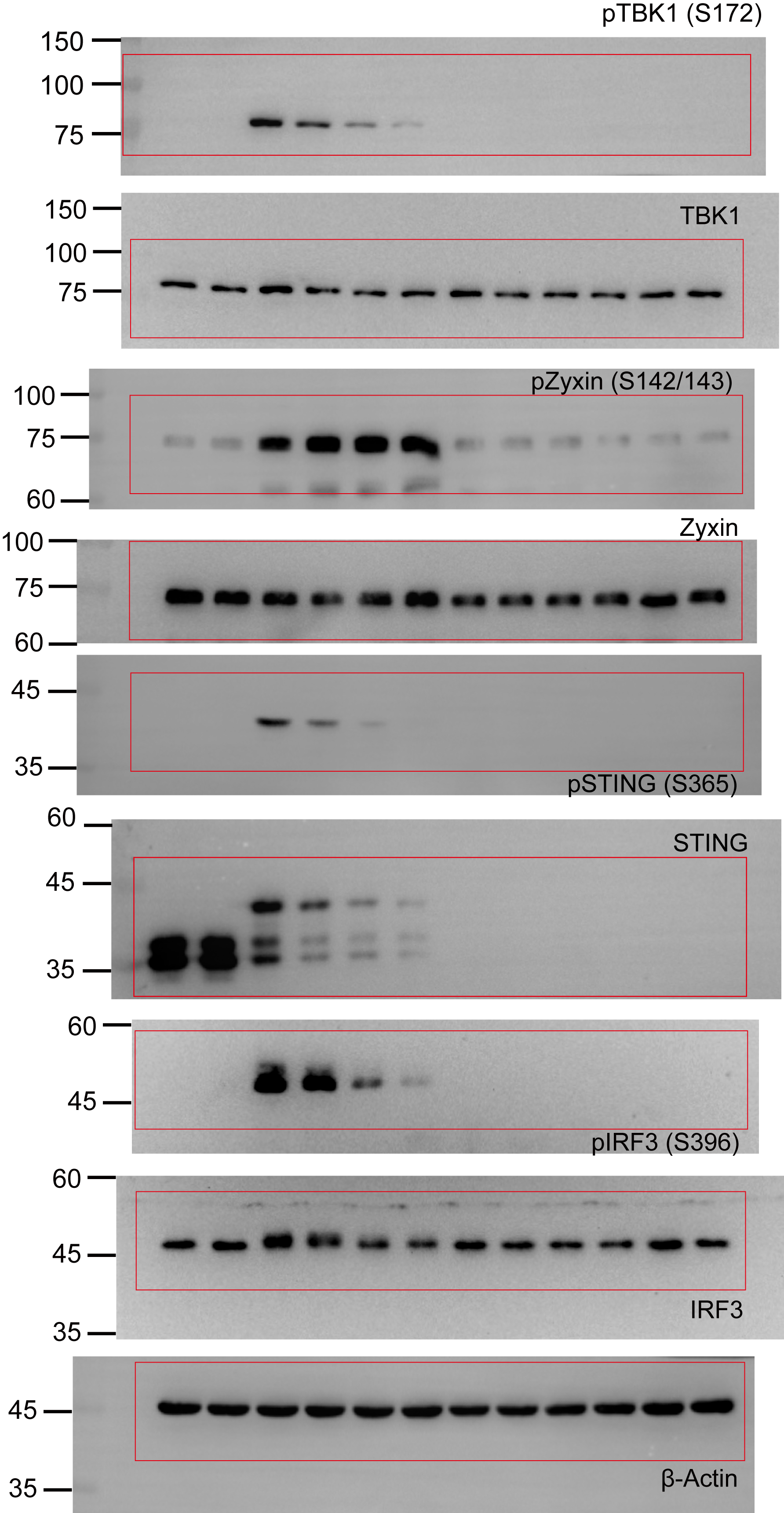

Supplement: Supplementary file 6 — Source data Fig. 2 [file 44318_2024_244_MOESM6_ESM.zip › Figure 2/2I/2L.tif]

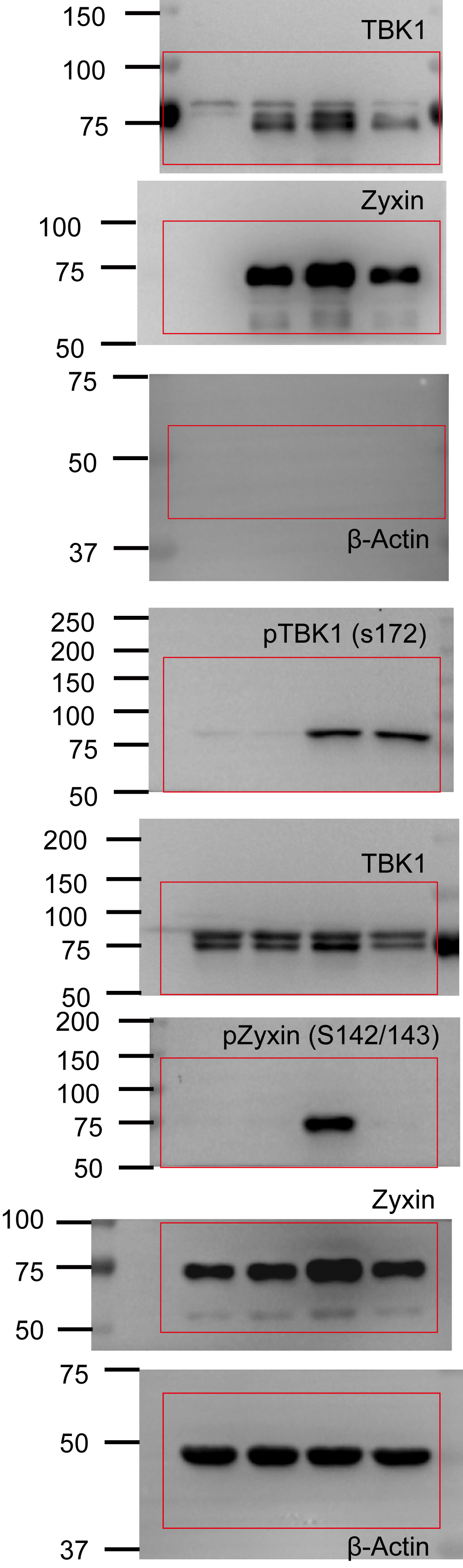

Supplement: Supplementary file 7 — Source data Fig. 3 [file 44318_2024_244_MOESM7_ESM.zip › Figure 3/3A/3A.tif]

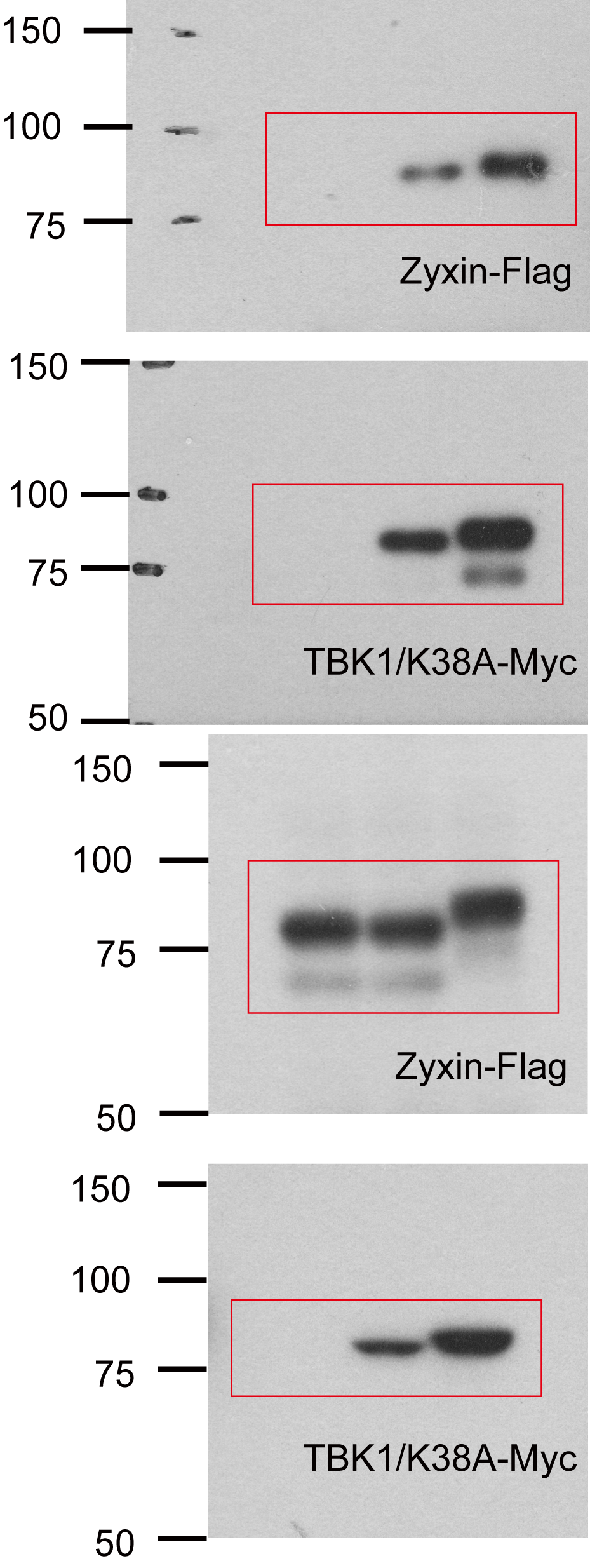

Supplement: Supplementary file 7 — Source data Fig. 3 [file 44318_2024_244_MOESM7_ESM.zip › Figure 3/3B/3B.tif]

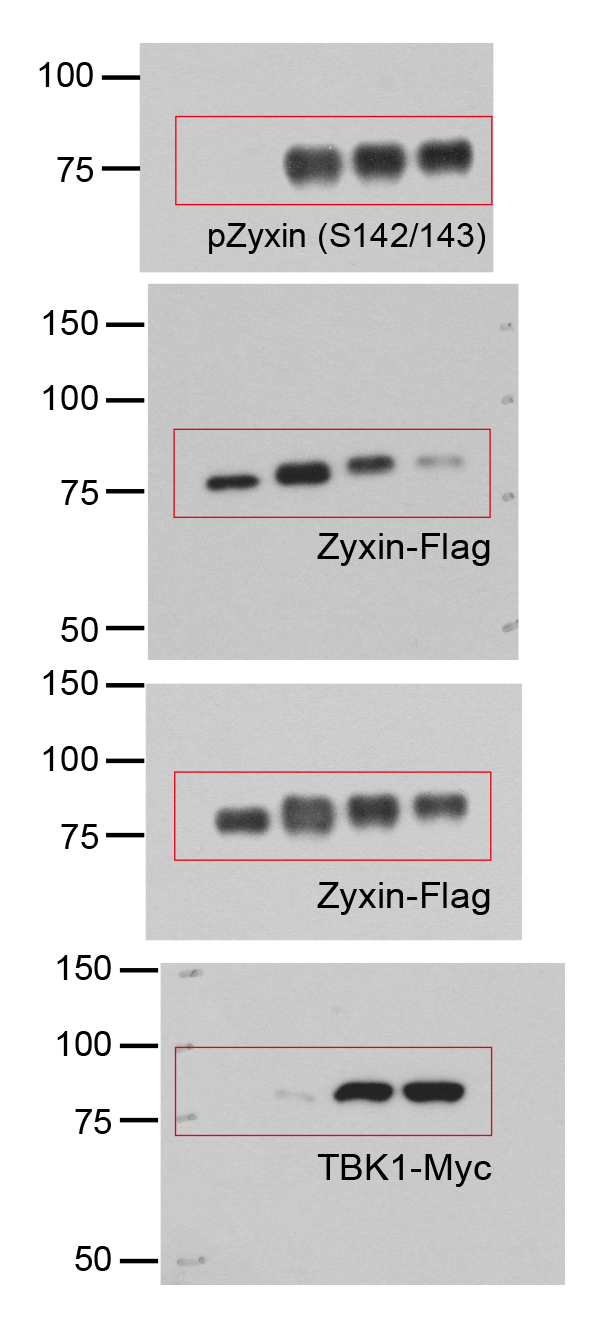

Supplement: Supplementary file 7 — Source data Fig. 3 [file 44318_2024_244_MOESM7_ESM.zip › Figure 3/3C/3C.tif]

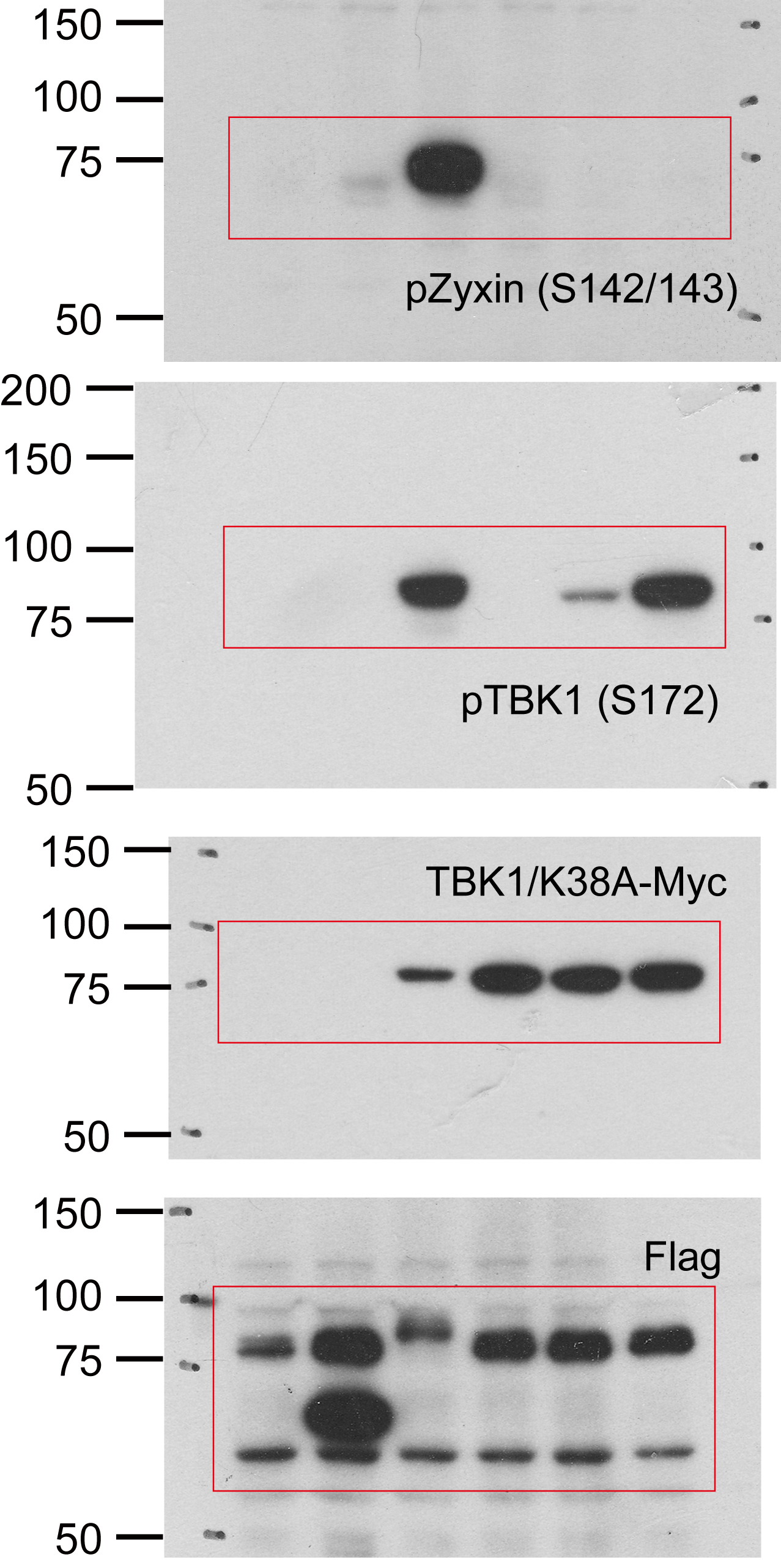

Supplement: Supplementary file 7 — Source data Fig. 3 [file 44318_2024_244_MOESM7_ESM.zip › Figure 3/3D/3D.tif]

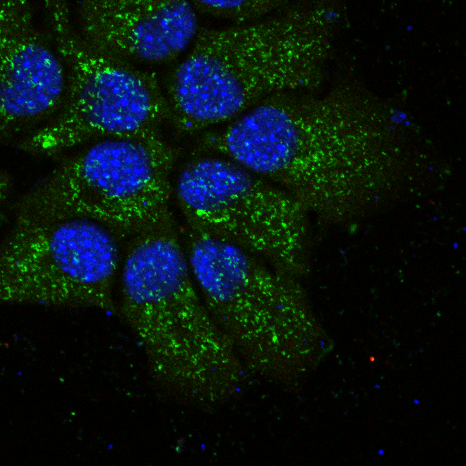

Supplement: Supplementary file 7 — Source data Fig. 3 [file 44318_2024_244_MOESM7_ESM.zip › Figure 3/3E/NMuMG Ctrl Merge.tif]

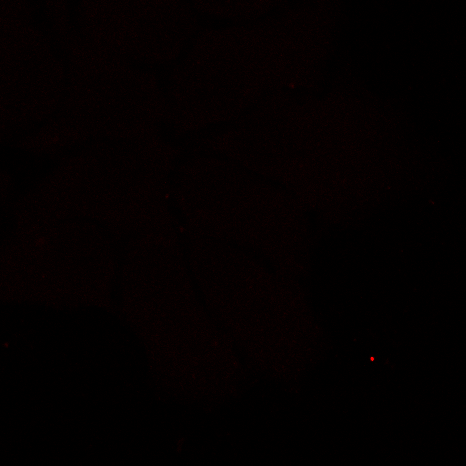

Supplement: Supplementary file 7 — Source data Fig. 3 [file 44318_2024_244_MOESM7_ESM.zip › Figure 3/3E/NMuMG Ctrl TBK1-Myc.tif]

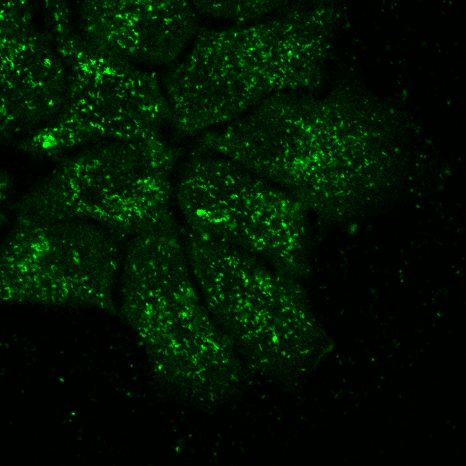

Supplement: Supplementary file 7 — Source data Fig. 3 [file 44318_2024_244_MOESM7_ESM.zip › Figure 3/3E/NMuMG Ctrl Zyxin.tif]

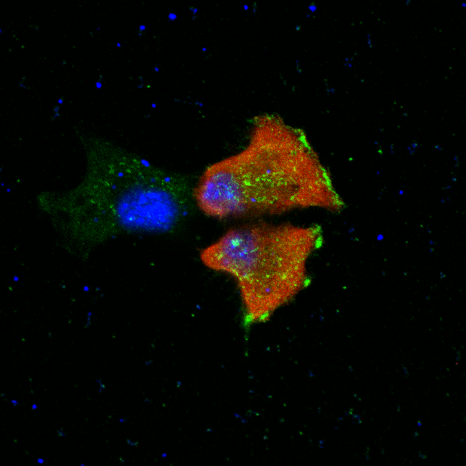

Supplement: Supplementary file 7 — Source data Fig. 3 [file 44318_2024_244_MOESM7_ESM.zip › Figure 3/3E/NMuMG TBK1 Merge.tif]

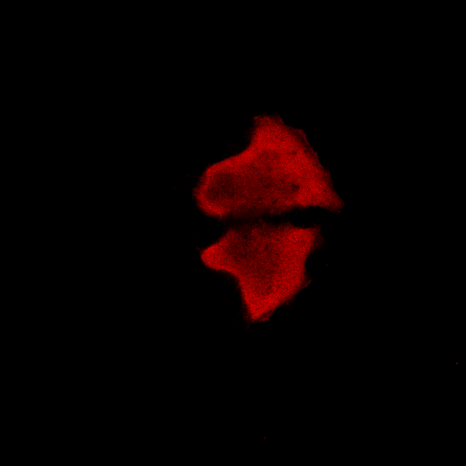

Supplement: Supplementary file 7 — Source data Fig. 3 [file 44318_2024_244_MOESM7_ESM.zip › Figure 3/3E/NMuMG TBK1 TBK1-Myc.tif]

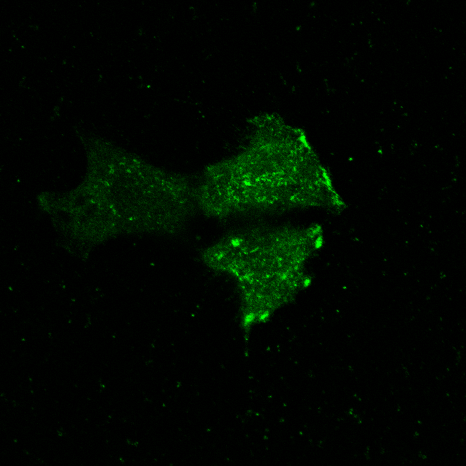

Supplement: Supplementary file 7 — Source data Fig. 3 [file 44318_2024_244_MOESM7_ESM.zip › Figure 3/3E/NMuMG TBK1 Zyxin.tif]

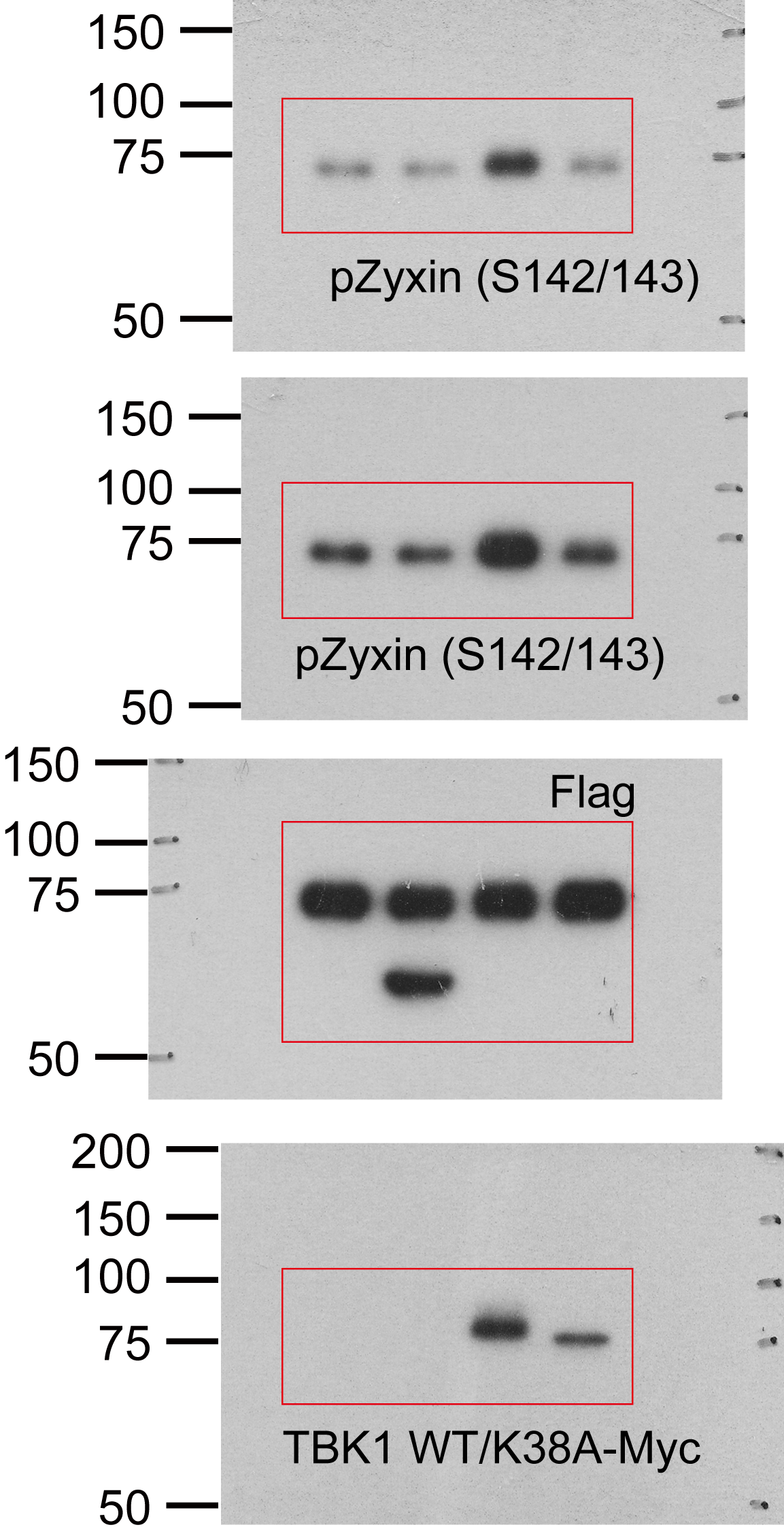

Supplement: Supplementary file 7 — Source data Fig. 3 [file 44318_2024_244_MOESM7_ESM.zip › Figure 3/3F/3F.tif]

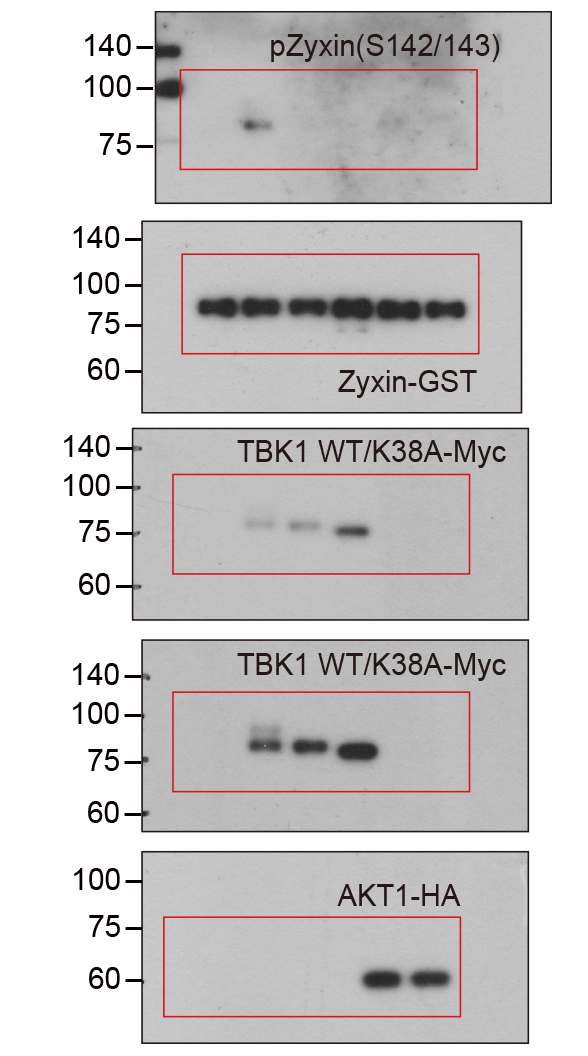

Supplement: Supplementary file 7 — Source data Fig. 3 [file 44318_2024_244_MOESM7_ESM.zip › Figure 3/3G/3G.tif]

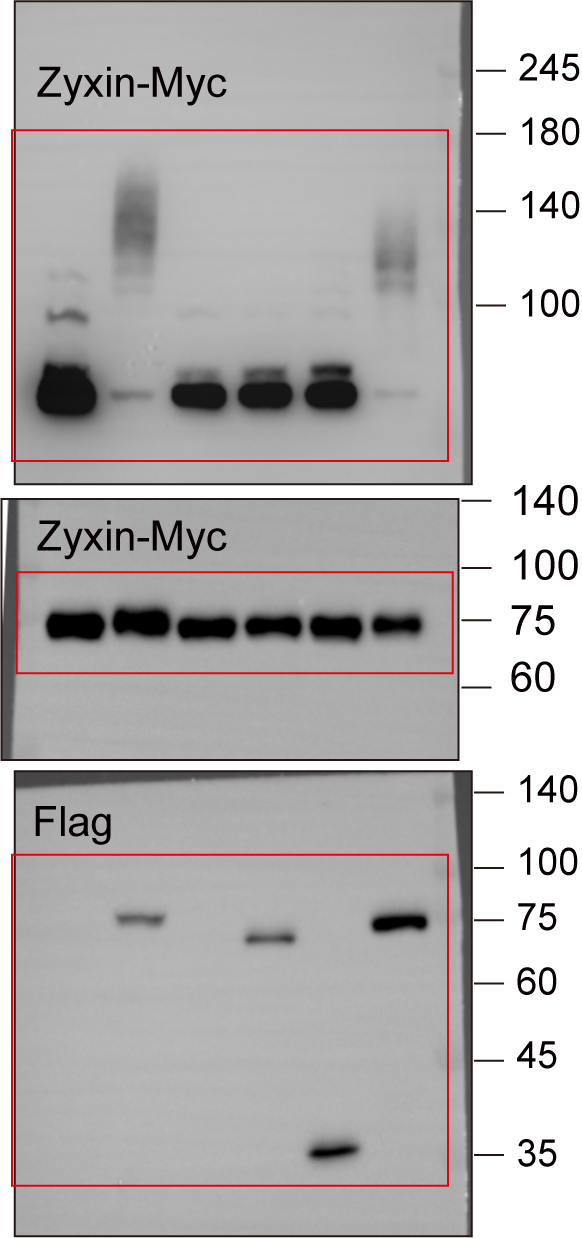

Supplement: Supplementary file 7 — Source data Fig. 3 [file 44318_2024_244_MOESM7_ESM.zip › Figure 3/3H/3H.tif]

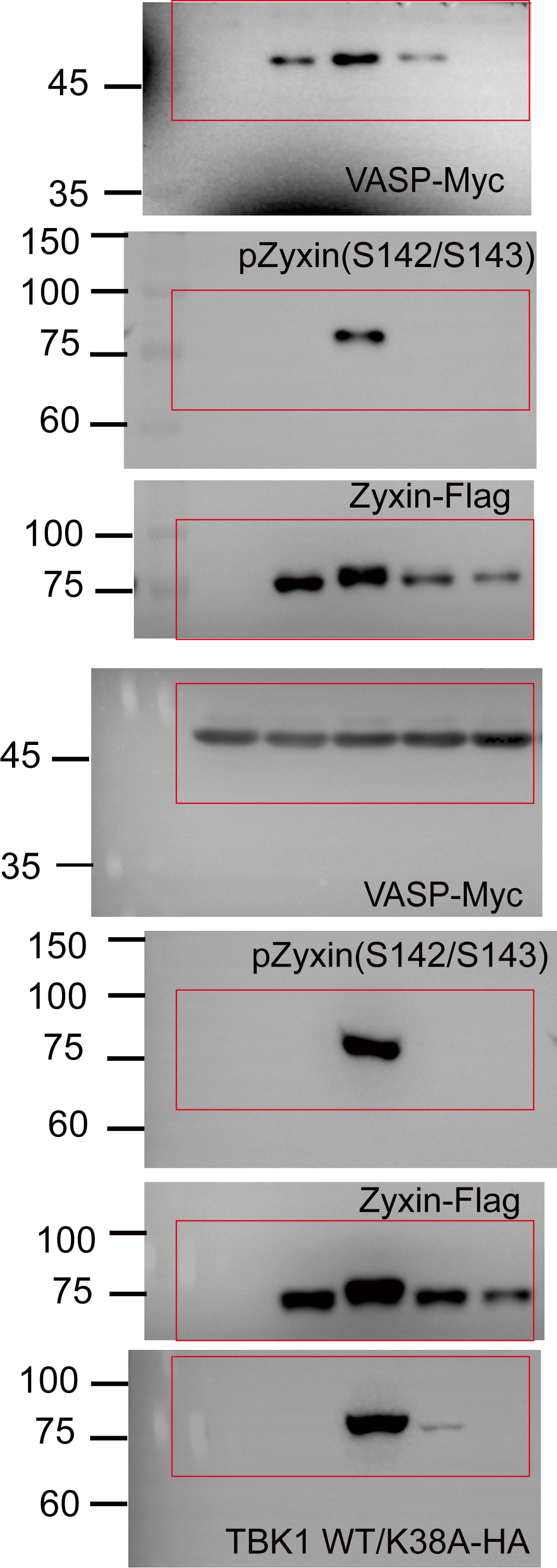

Supplement: Supplementary file 7 — Source data Fig. 3 [file 44318_2024_244_MOESM7_ESM.zip › Figure 3/3J/3J.tif]

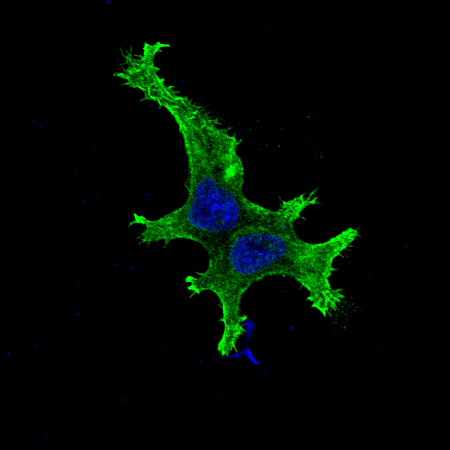

Supplement: Supplementary file 7 — Source data Fig. 3 [file 44318_2024_244_MOESM7_ESM.zip › Figure 3/3K/VASP Merge.tif]

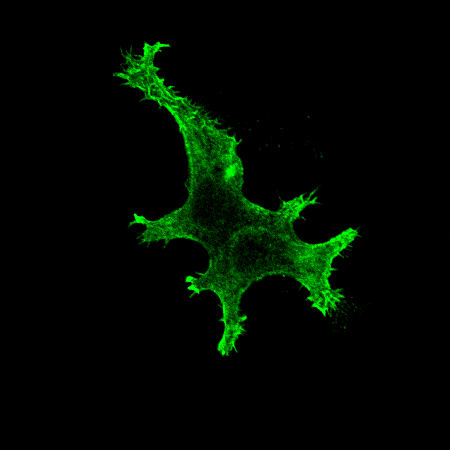

Supplement: Supplementary file 7 — Source data Fig. 3 [file 44318_2024_244_MOESM7_ESM.zip › Figure 3/3K/VASP VASP.tif]

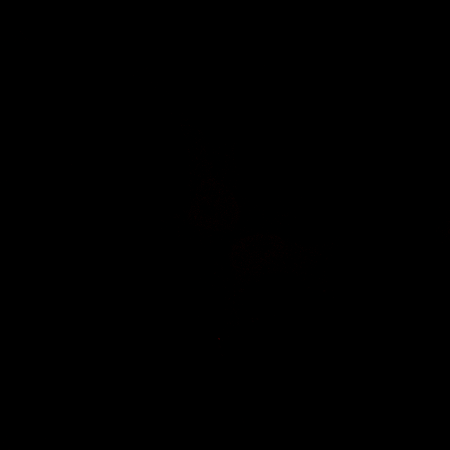

Supplement: Supplementary file 7 — Source data Fig. 3 [file 44318_2024_244_MOESM7_ESM.zip › Figure 3/3K/VASP Zyxin.tif]

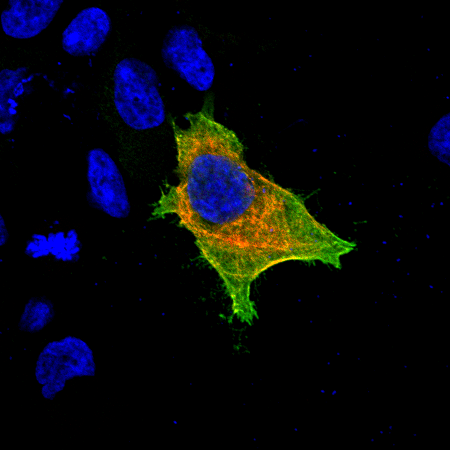

Supplement: Supplementary file 7 — Source data Fig. 3 [file 44318_2024_244_MOESM7_ESM.zip › Figure 3/3K/VASP+Zyxin Merge.tif]

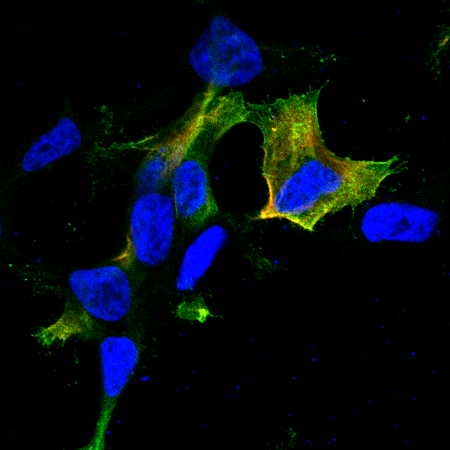

Supplement: Supplementary file 7 — Source data Fig. 3 [file 44318_2024_244_MOESM7_ESM.zip › Figure 3/3K/VASP+Zyxin S142 143A Merge.tif]

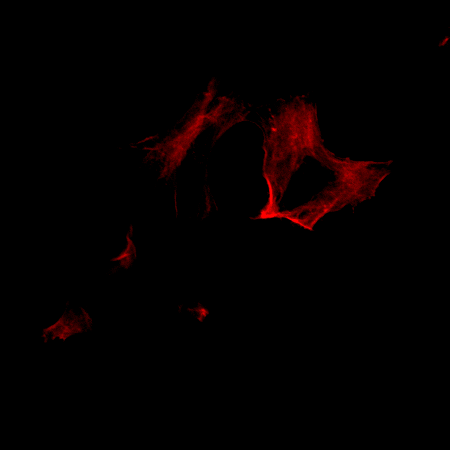

Supplement: Supplementary file 7 — Source data Fig. 3 [file 44318_2024_244_MOESM7_ESM.zip › Figure 3/3K/VASP+Zyxin S142 143A VASP.tif]

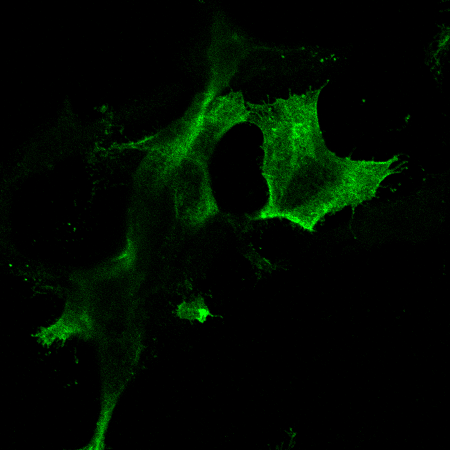

Supplement: Supplementary file 7 — Source data Fig. 3 [file 44318_2024_244_MOESM7_ESM.zip › Figure 3/3K/VASP+Zyxin S142 143A Zyxin S142 143A.tif]

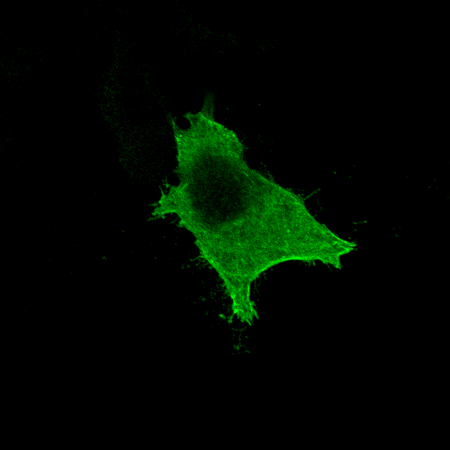

Supplement: Supplementary file 7 — Source data Fig. 3 [file 44318_2024_244_MOESM7_ESM.zip › Figure 3/3K/VASP+Zyxin VASP.tif]

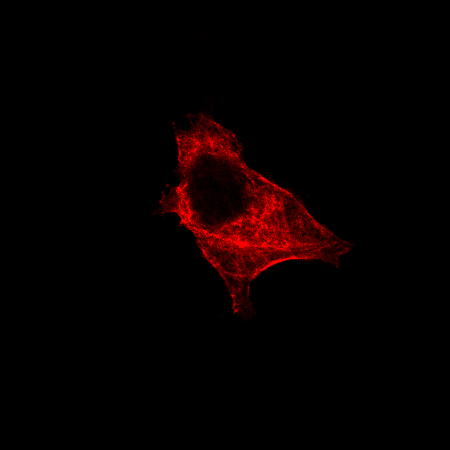

Supplement: Supplementary file 7 — Source data Fig. 3 [file 44318_2024_244_MOESM7_ESM.zip › Figure 3/3K/VASP+Zyxin Zyxin.tif]

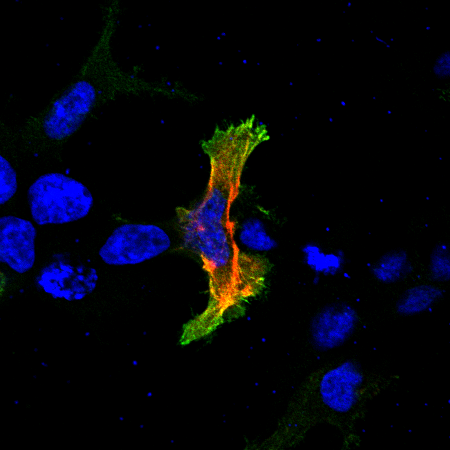

Supplement: Supplementary file 7 — Source data Fig. 3 [file 44318_2024_244_MOESM7_ESM.zip › Figure 3/3K/VASP+Zyxin+TBK1 K38A Merge.tif]

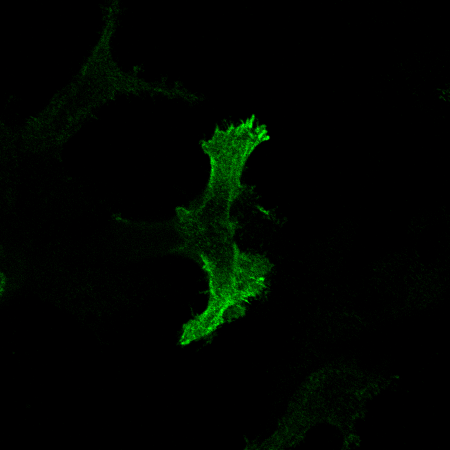

Supplement: Supplementary file 7 — Source data Fig. 3 [file 44318_2024_244_MOESM7_ESM.zip › Figure 3/3K/VASP+Zyxin+TBK1 K38A VASP.tif]

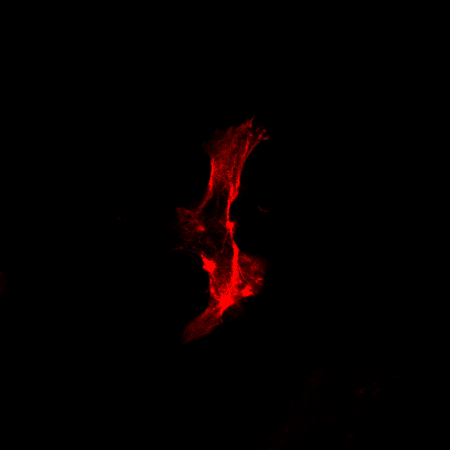

Supplement: Supplementary file 7 — Source data Fig. 3 [file 44318_2024_244_MOESM7_ESM.zip › Figure 3/3K/VASP+Zyxin+TBK1 K38A Zyxin.tif]

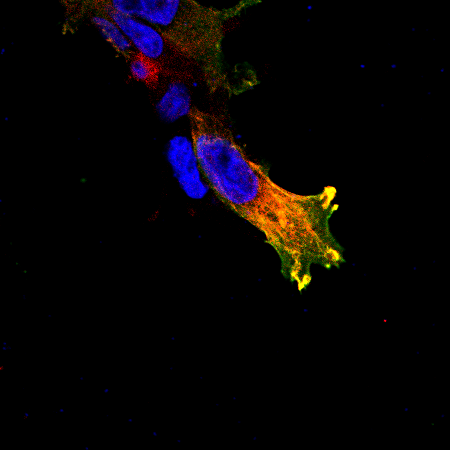

Supplement: Supplementary file 7 — Source data Fig. 3 [file 44318_2024_244_MOESM7_ESM.zip › Figure 3/3K/VASP+Zyxin+TBK1 Merge.tif]

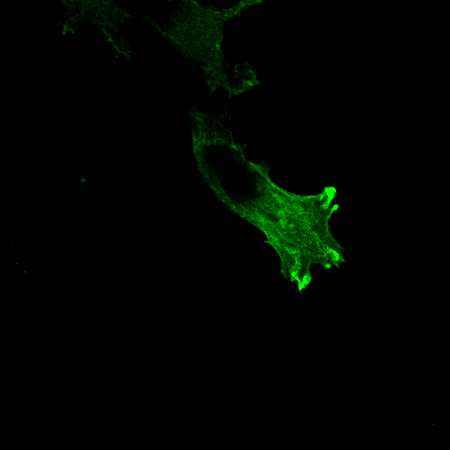

Supplement: Supplementary file 7 — Source data Fig. 3 [file 44318_2024_244_MOESM7_ESM.zip › Figure 3/3K/VASP+Zyxin+TBK1 VASP.tif]

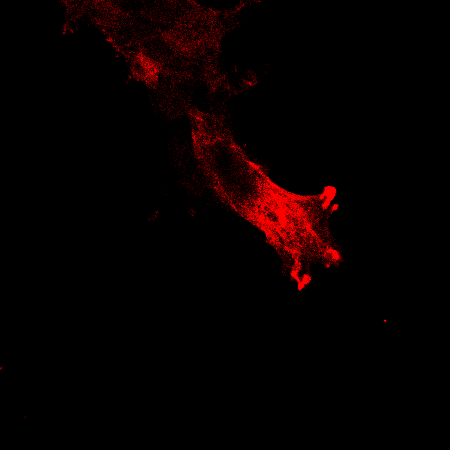

Supplement: Supplementary file 7 — Source data Fig. 3 [file 44318_2024_244_MOESM7_ESM.zip › Figure 3/3K/VASP+Zyxin+TBK1 Zyxin.tif]

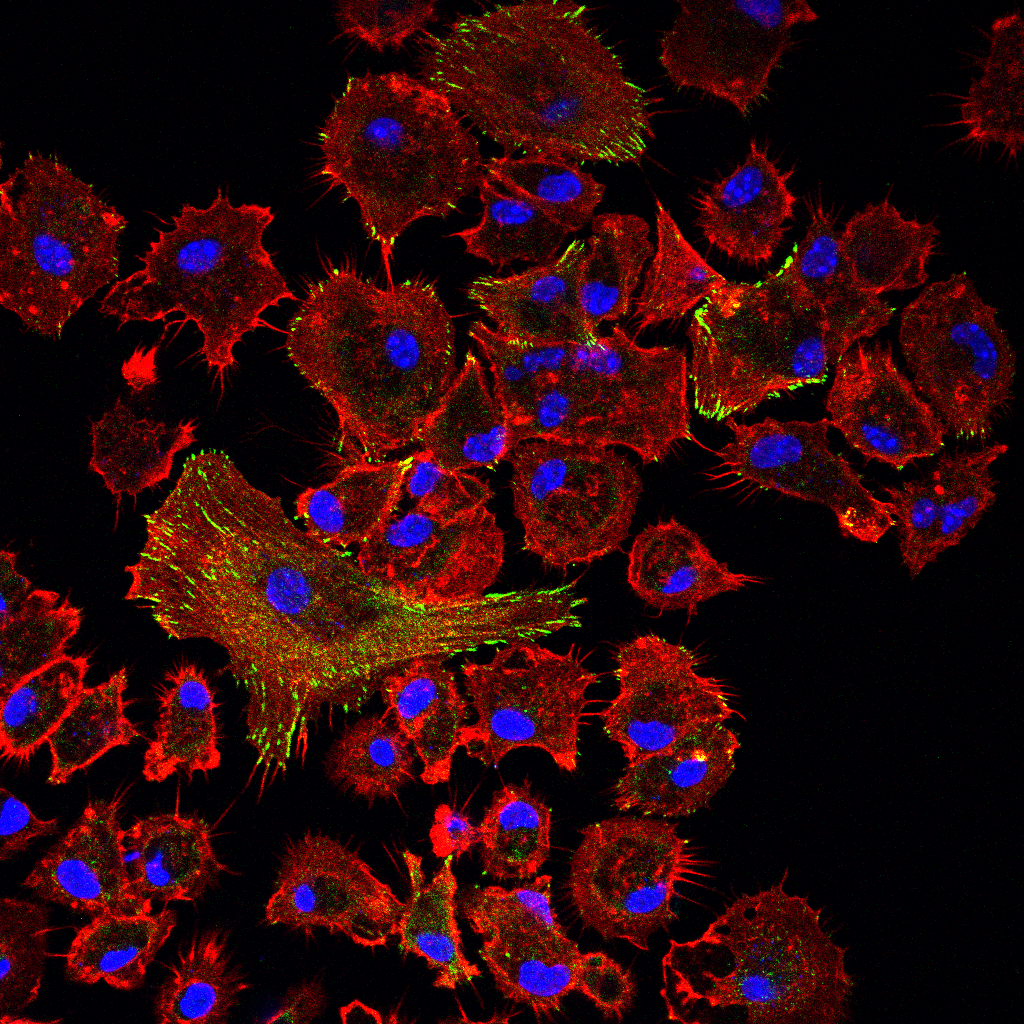

Supplement: Supplementary file 8 — Source data Fig. 4 [file 44318_2024_244_MOESM8_ESM.zip › Figure 4/4A/SeV pZyxin.tif]

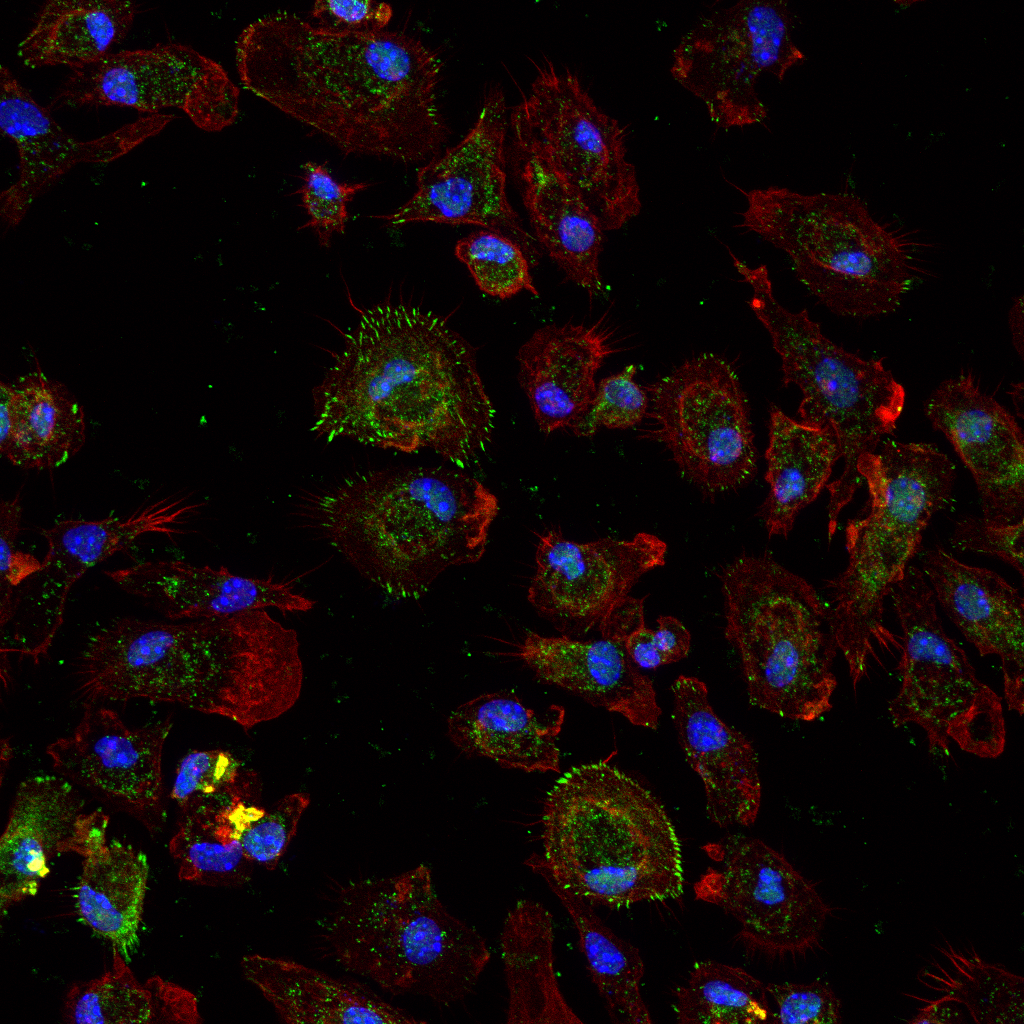

Supplement: Supplementary file 8 — Source data Fig. 4 [file 44318_2024_244_MOESM8_ESM.zip › Figure 4/4A/SeV Zyxin.tif]

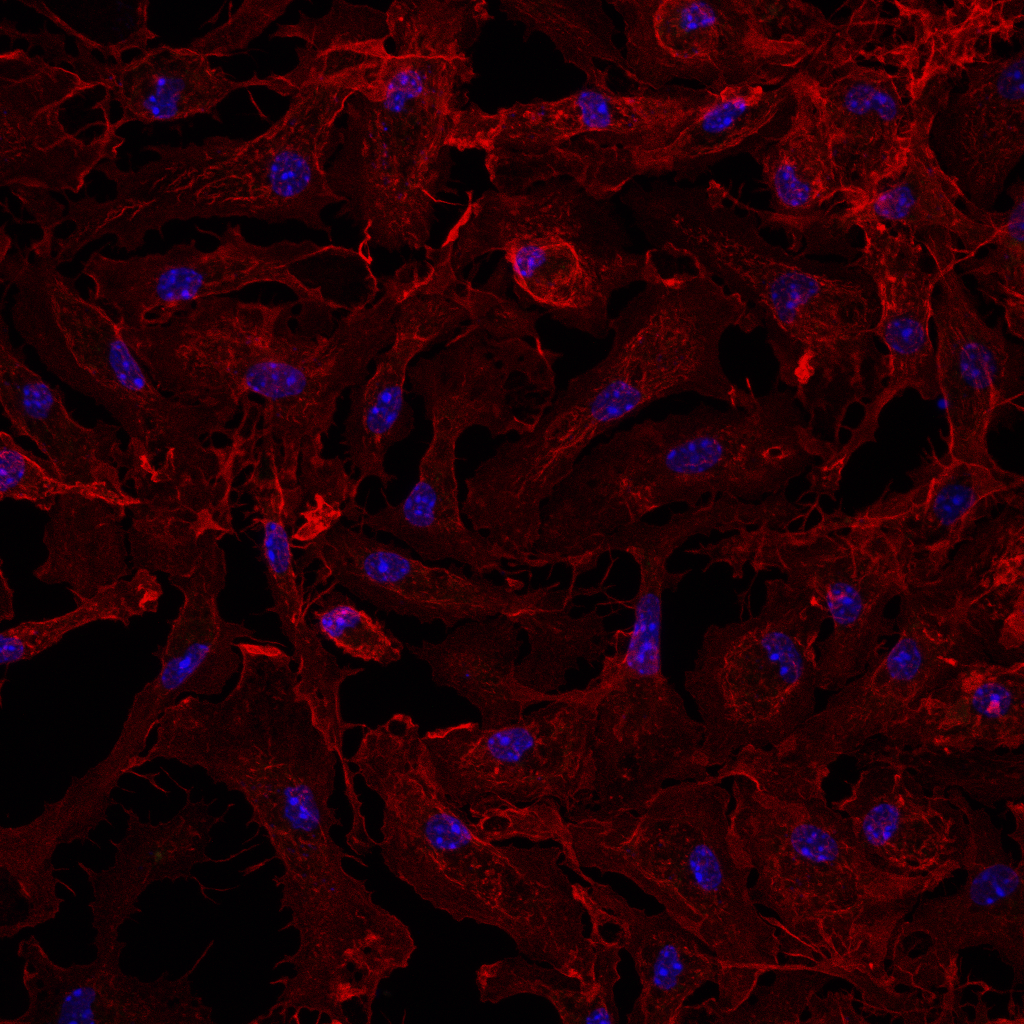

Supplement: Supplementary file 8 — Source data Fig. 4 [file 44318_2024_244_MOESM8_ESM.zip › Figure 4/4A/SeV+MRT67307 pZyxin.tif]

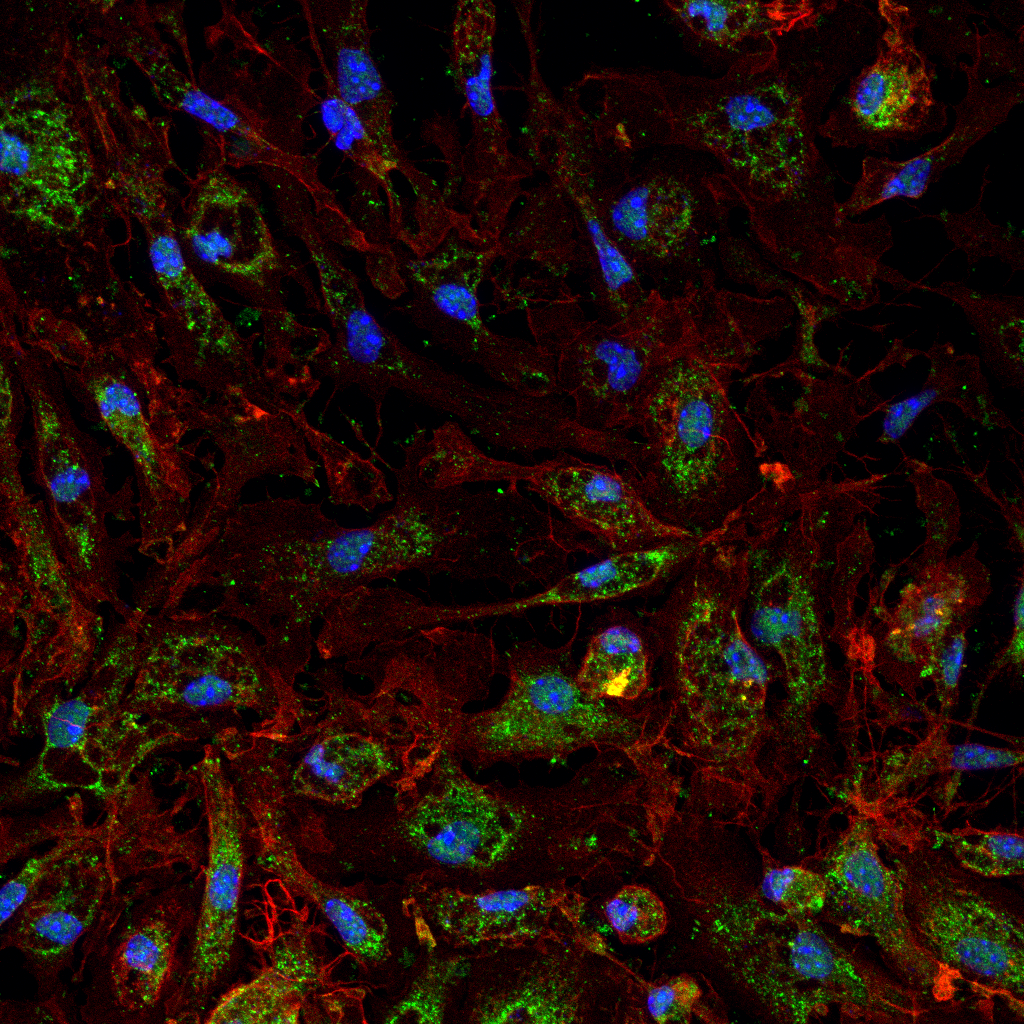

Supplement: Supplementary file 8 — Source data Fig. 4 [file 44318_2024_244_MOESM8_ESM.zip › Figure 4/4A/SeV+MRT67307 Zyxin.tif]

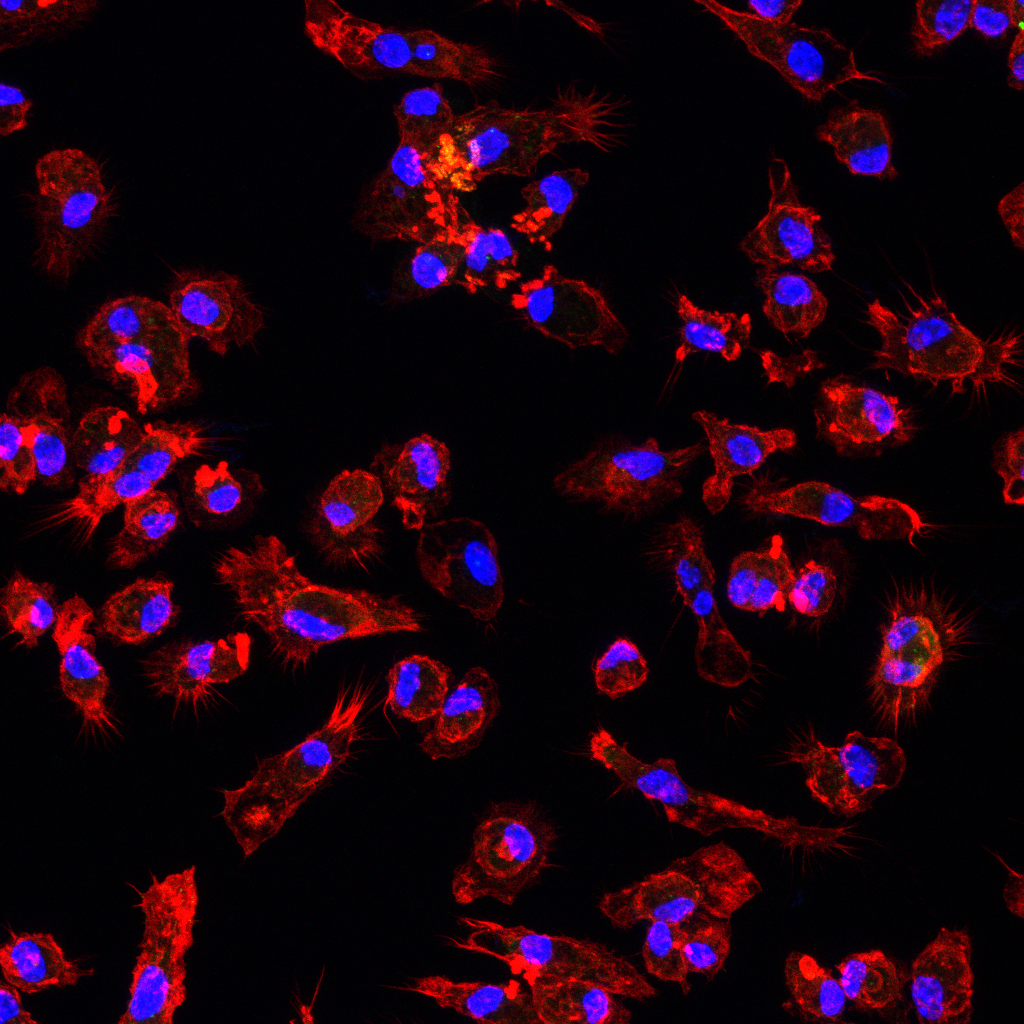

Supplement: Supplementary file 8 — Source data Fig. 4 [file 44318_2024_244_MOESM8_ESM.zip › Figure 4/4A/Vehicle pZyxin.tif]

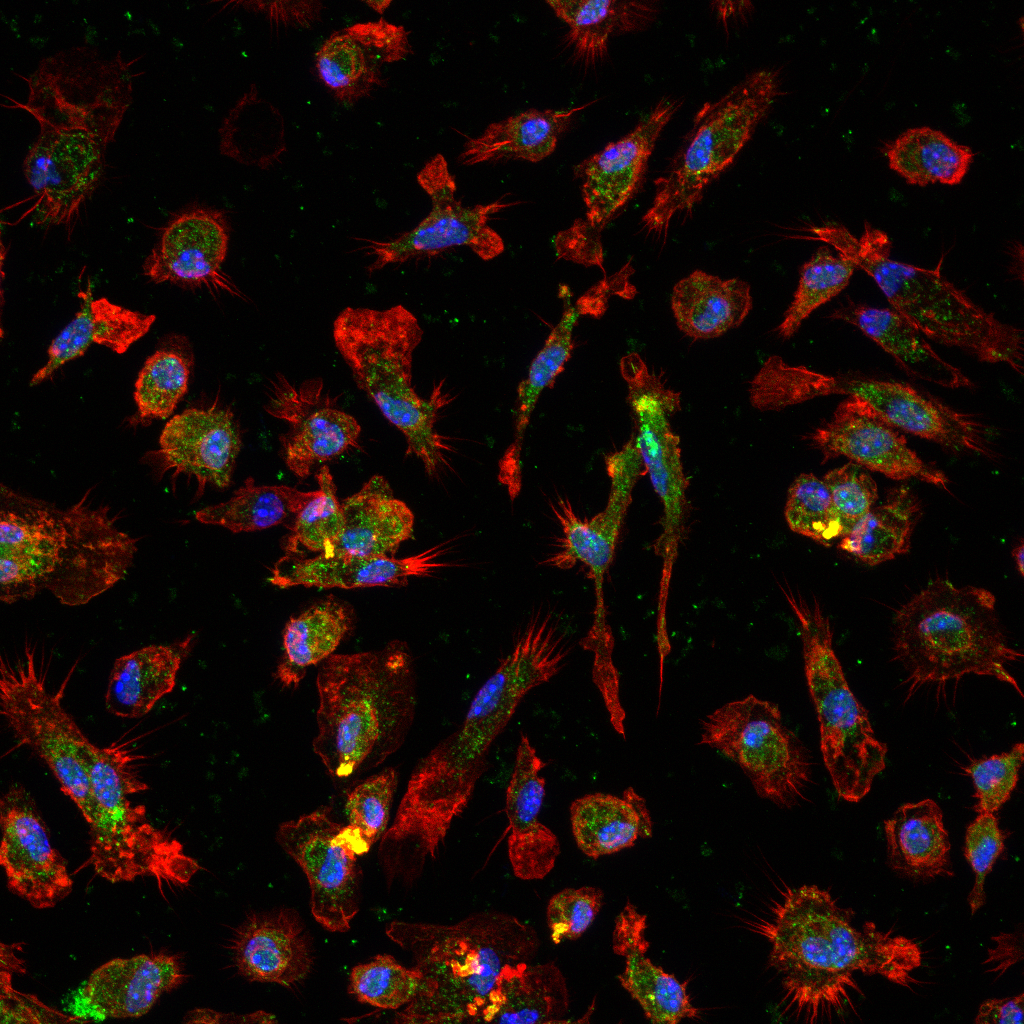

Supplement: Supplementary file 8 — Source data Fig. 4 [file 44318_2024_244_MOESM8_ESM.zip › Figure 4/4A/Vehicle Zyxin.tif]

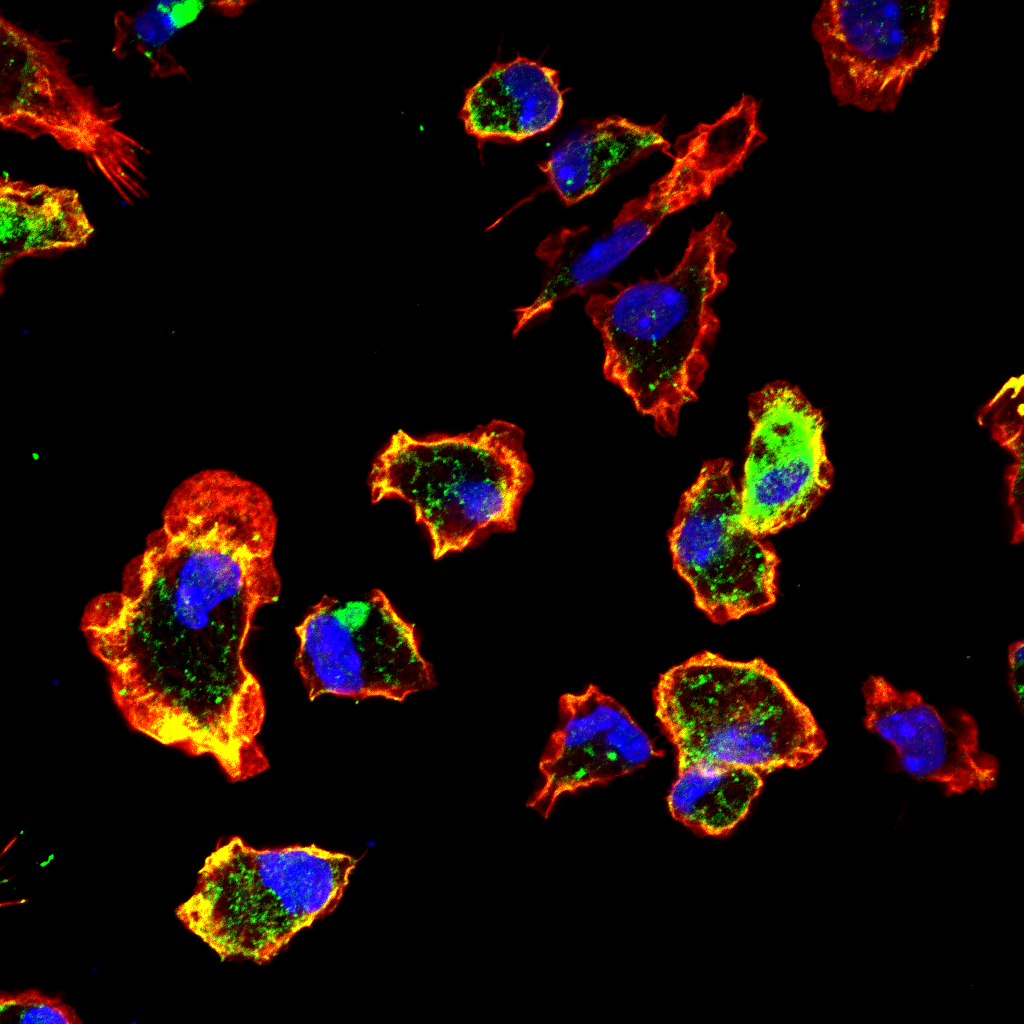

Supplement: Supplementary file 8 — Source data Fig. 4 [file 44318_2024_244_MOESM8_ESM.zip › Figure 4/4C/STING KO DMXAA Zyxin.tif]

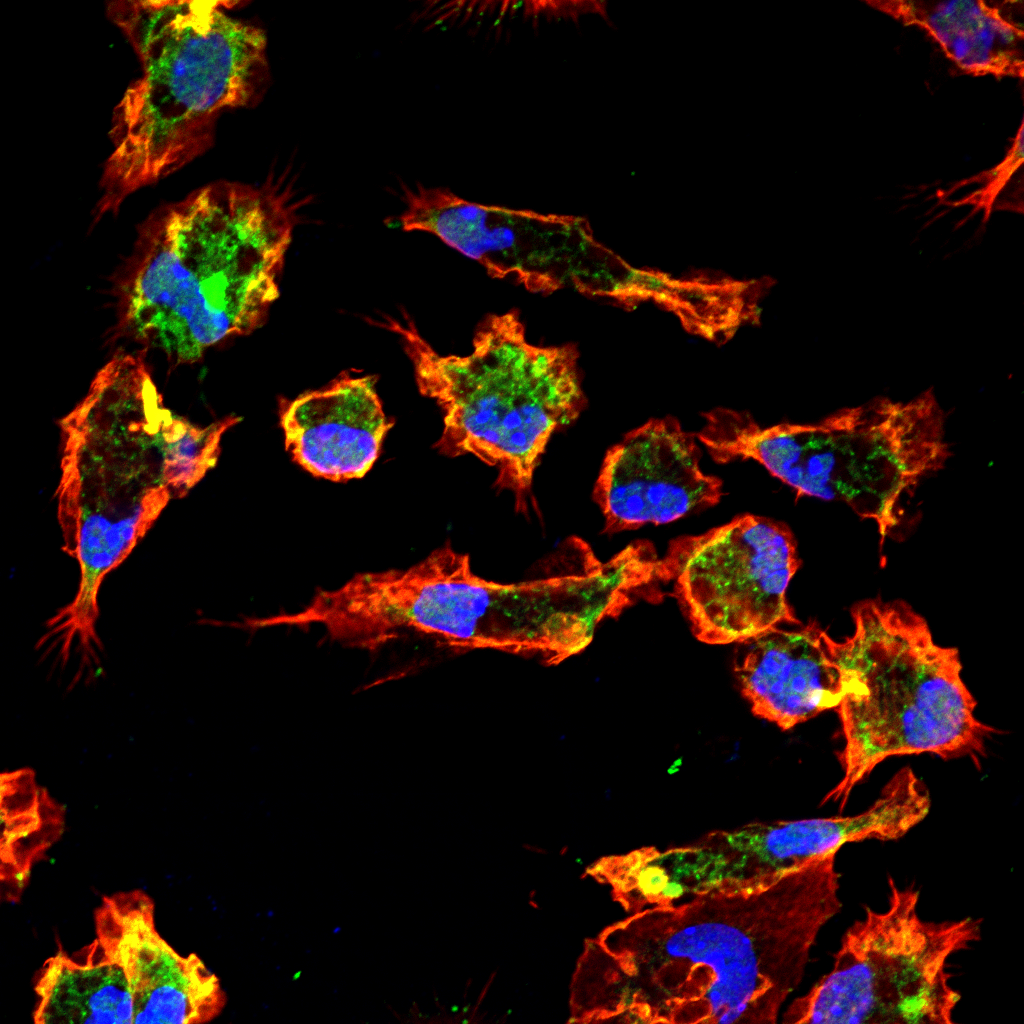

Supplement: Supplementary file 8 — Source data Fig. 4 [file 44318_2024_244_MOESM8_ESM.zip › Figure 4/4C/STING KO Vehicle Zyxin.tif]

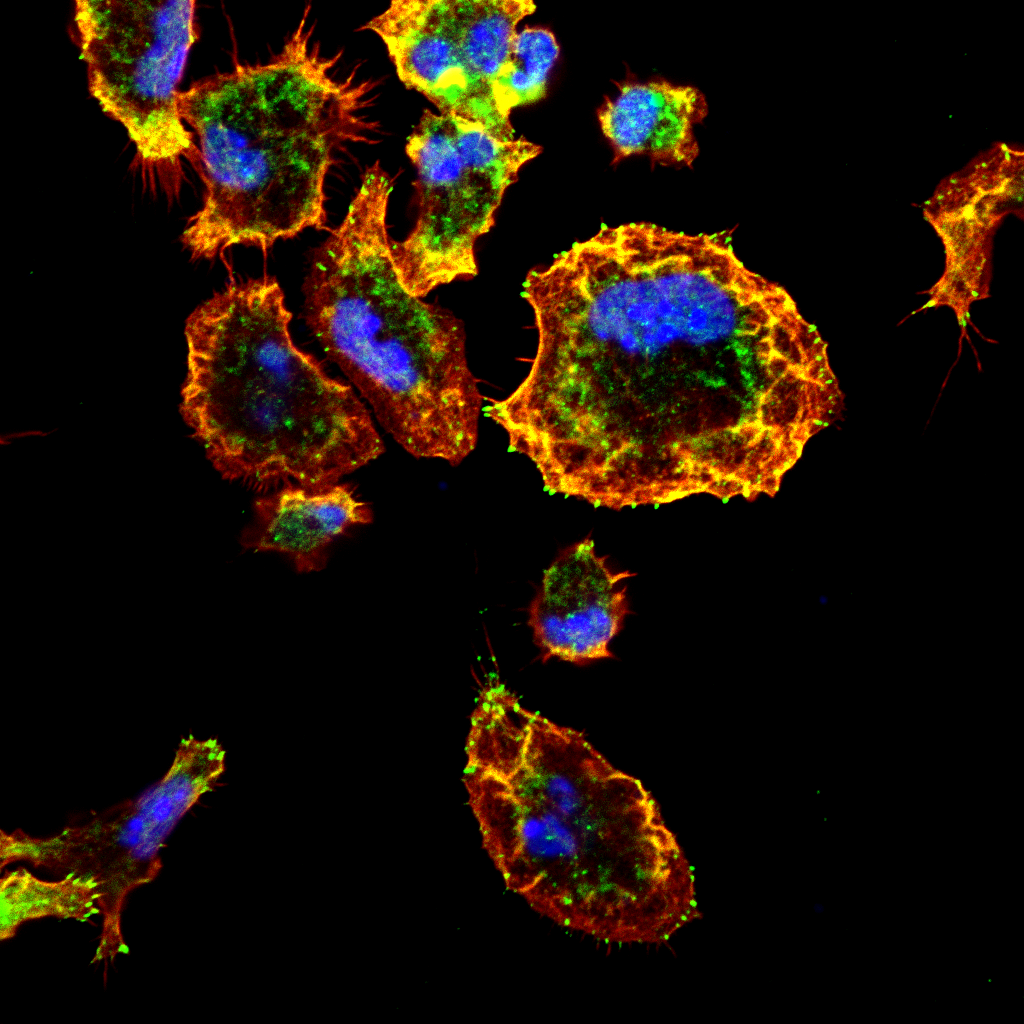

Supplement: Supplementary file 8 — Source data Fig. 4 [file 44318_2024_244_MOESM8_ESM.zip › Figure 4/4C/WT DMXAA Zyxin.tif]

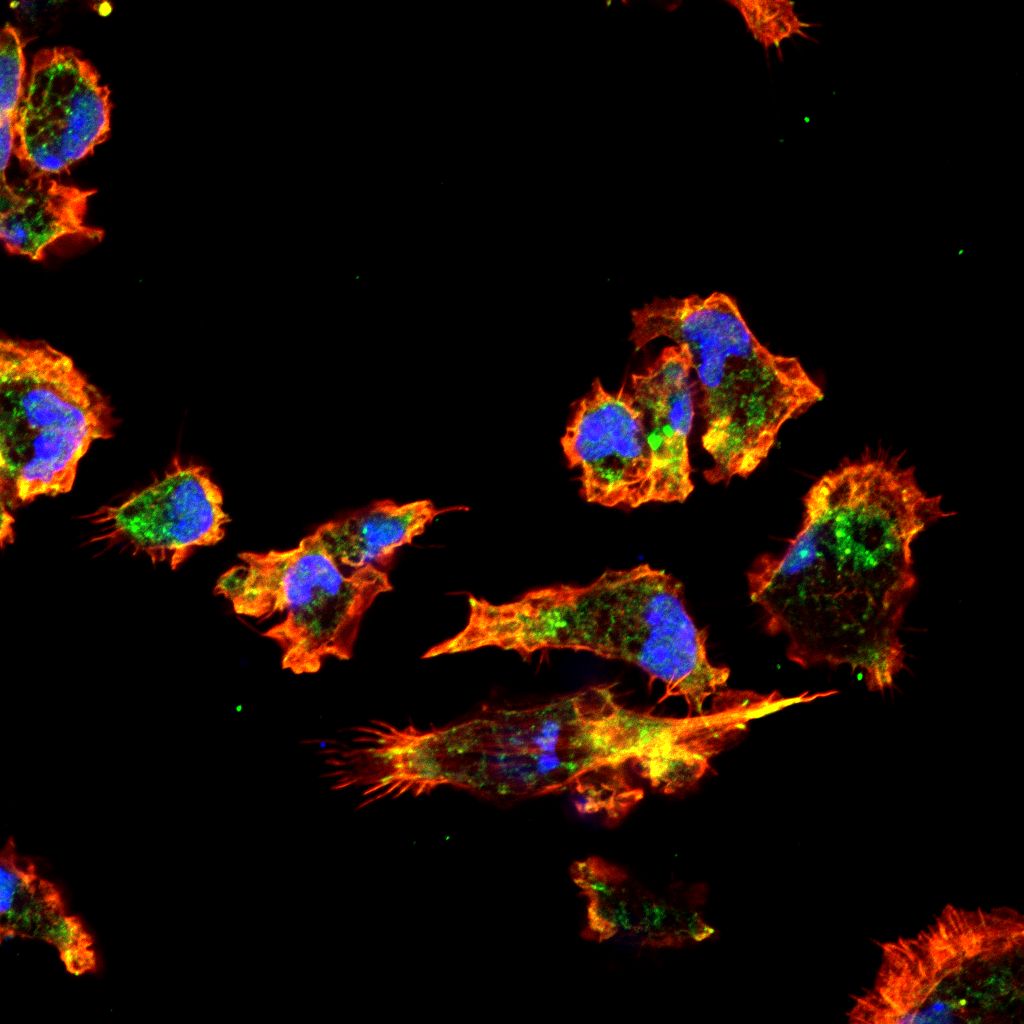

Supplement: Supplementary file 8 — Source data Fig. 4 [file 44318_2024_244_MOESM8_ESM.zip › Figure 4/4C/WT Vehicle Zyxin.tif]

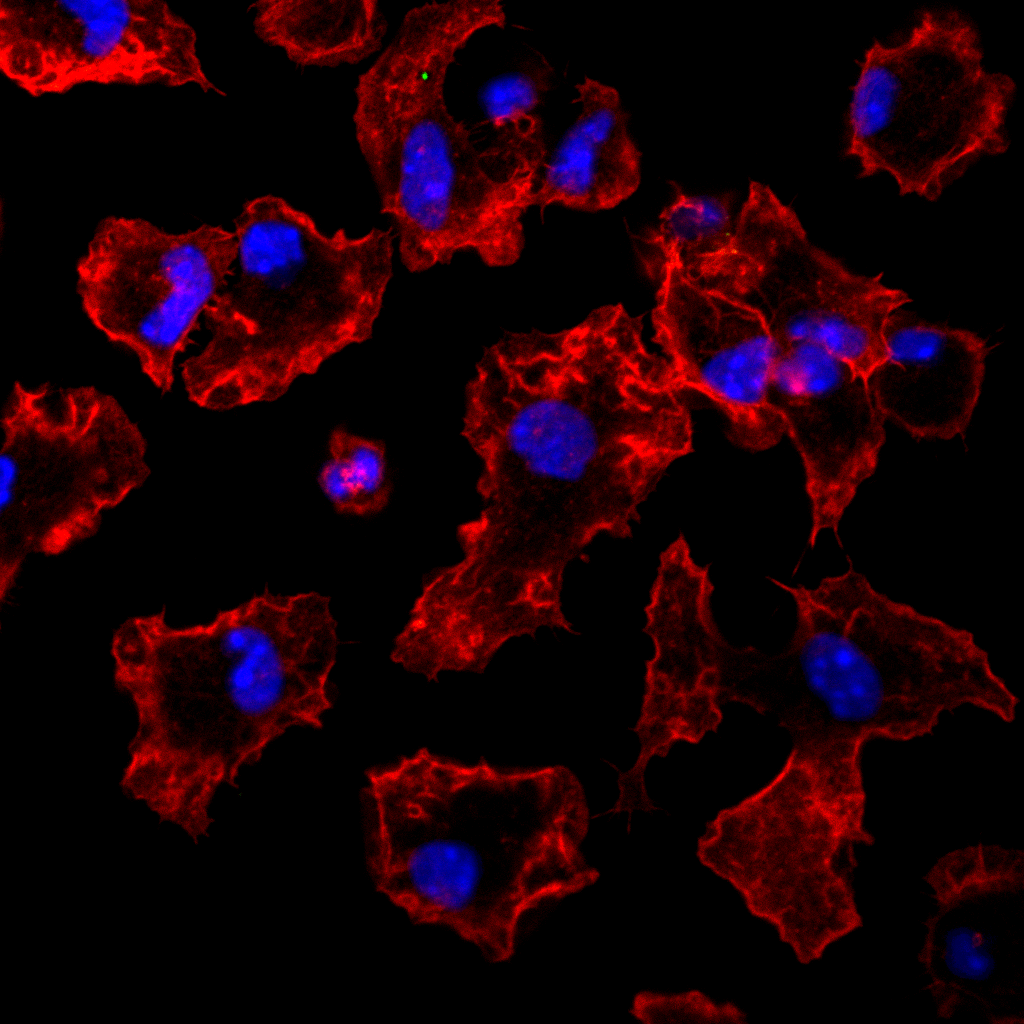

Supplement: Supplementary file 8 — Source data Fig. 4 [file 44318_2024_244_MOESM8_ESM.zip › Figure 4/4E/STING KO DMXAA pZyxin.tif]
