# Supplementary figures and images for: TBK1-Zyxin signaling controls tumor-associated macrophage recruitment to mitigate antitumor immunity (part 2 of 2)
Source: EMBO J. 2024 Sep 20;43(21):4984–5017. doi: 10.1038/s44318-024-00244-9 (PMC11535546; doi:10.1038/s44318-024-00244-9)

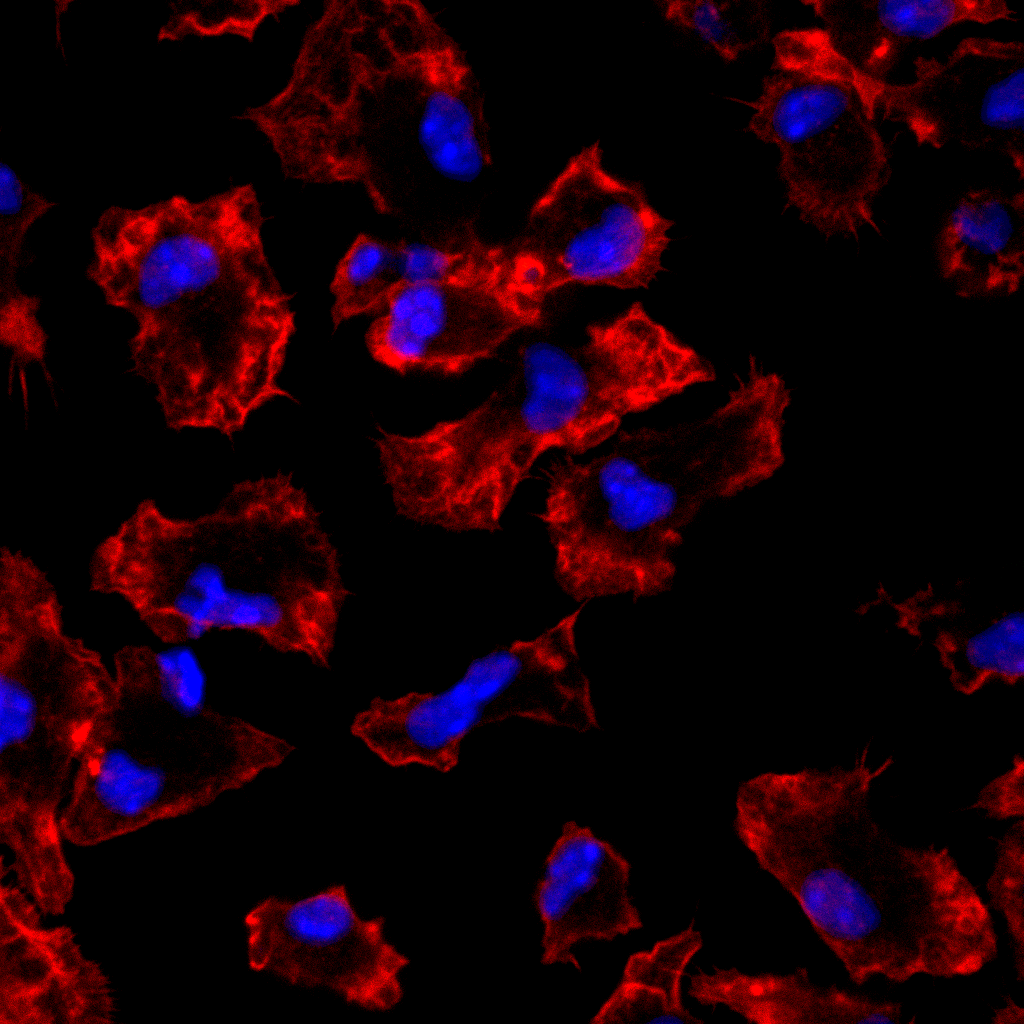

Supplement: Supplementary file 8 — Source data Fig. 4 [file 44318_2024_244_MOESM8_ESM.zip › Figure 4/4E/STING KO Vehicle pZyxin.tif]

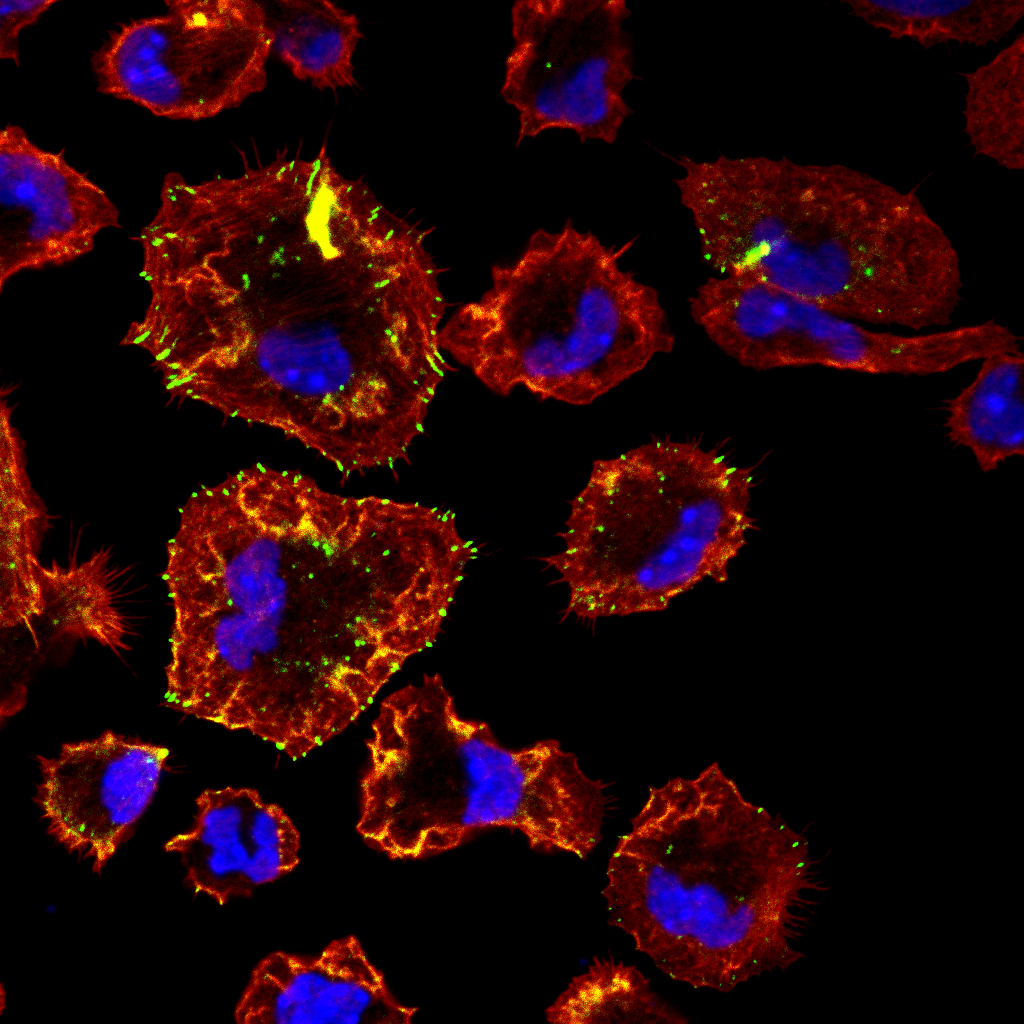

Supplement: Supplementary file 8 — Source data Fig. 4 [file 44318_2024_244_MOESM8_ESM.zip › Figure 4/4E/WT DMXAA pZyxin.tif]

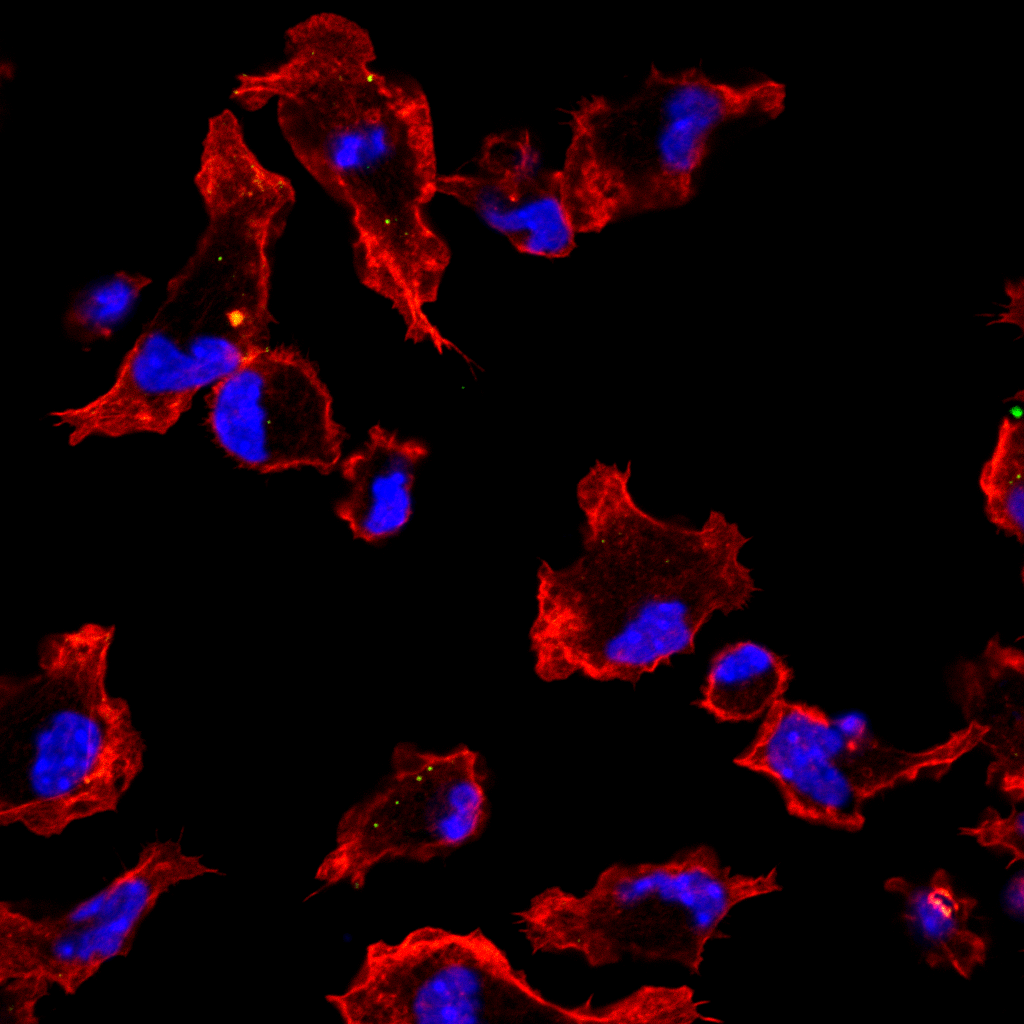

Supplement: Supplementary file 8 — Source data Fig. 4 [file 44318_2024_244_MOESM8_ESM.zip › Figure 4/4E/WT Vehicle pZyxin.tif]

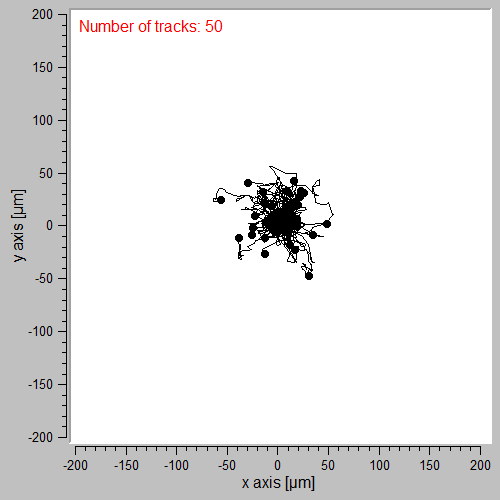

Supplement: Supplementary file 8 — Source data Fig. 4 [file 44318_2024_244_MOESM8_ESM.zip › Figure 4/4G/Statistics of WT PMs DMXAA in mVCAM Fc.bmp]

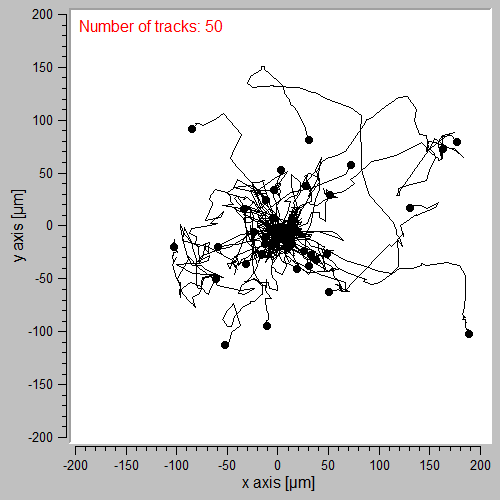

Supplement: Supplementary file 8 — Source data Fig. 4 [file 44318_2024_244_MOESM8_ESM.zip › Figure 4/4G/Statistics of WT PMs Vehicle in mVCAM Fc.bmp]

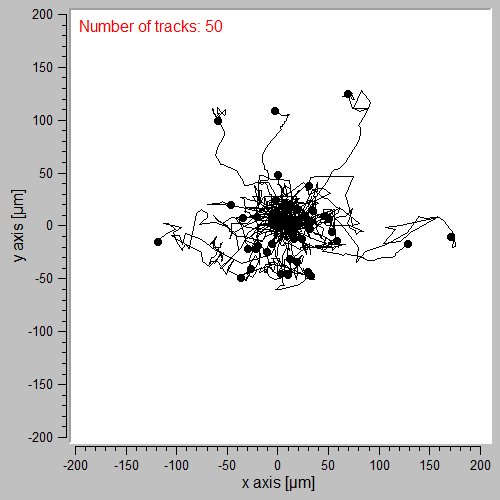

Supplement: Supplementary file 8 — Source data Fig. 4 [file 44318_2024_244_MOESM8_ESM.zip › Figure 4/4G/Statistics of Zyxin KO PMs DMXAA in mVCAM Fc.bmp]

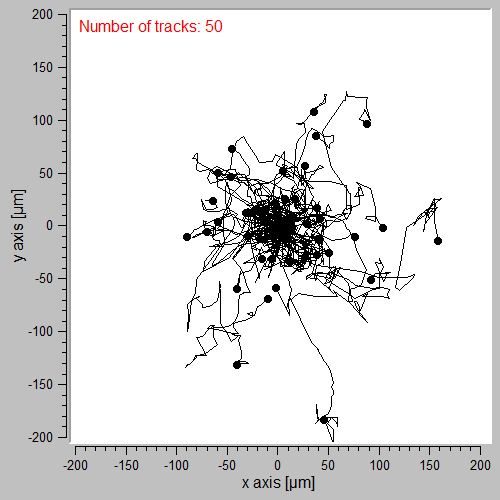

Supplement: Supplementary file 8 — Source data Fig. 4 [file 44318_2024_244_MOESM8_ESM.zip › Figure 4/4G/Statistics of Zyxin KO PMs Vehicle in mVCAM Fc.bmp]

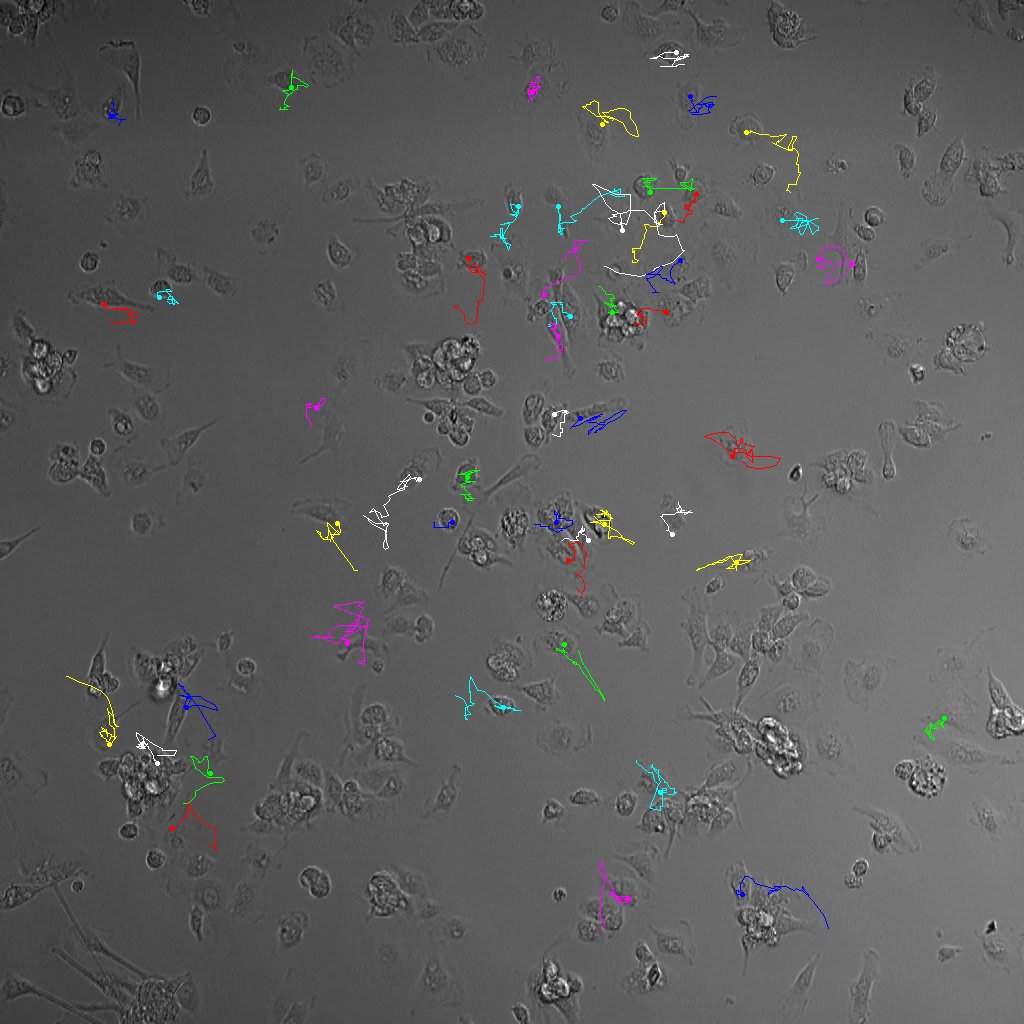

Supplement: Supplementary file 8 — Source data Fig. 4 [file 44318_2024_244_MOESM8_ESM.zip › Figure 4/4G/WT PMs DMXAA in mVCAM Fc.jpg]

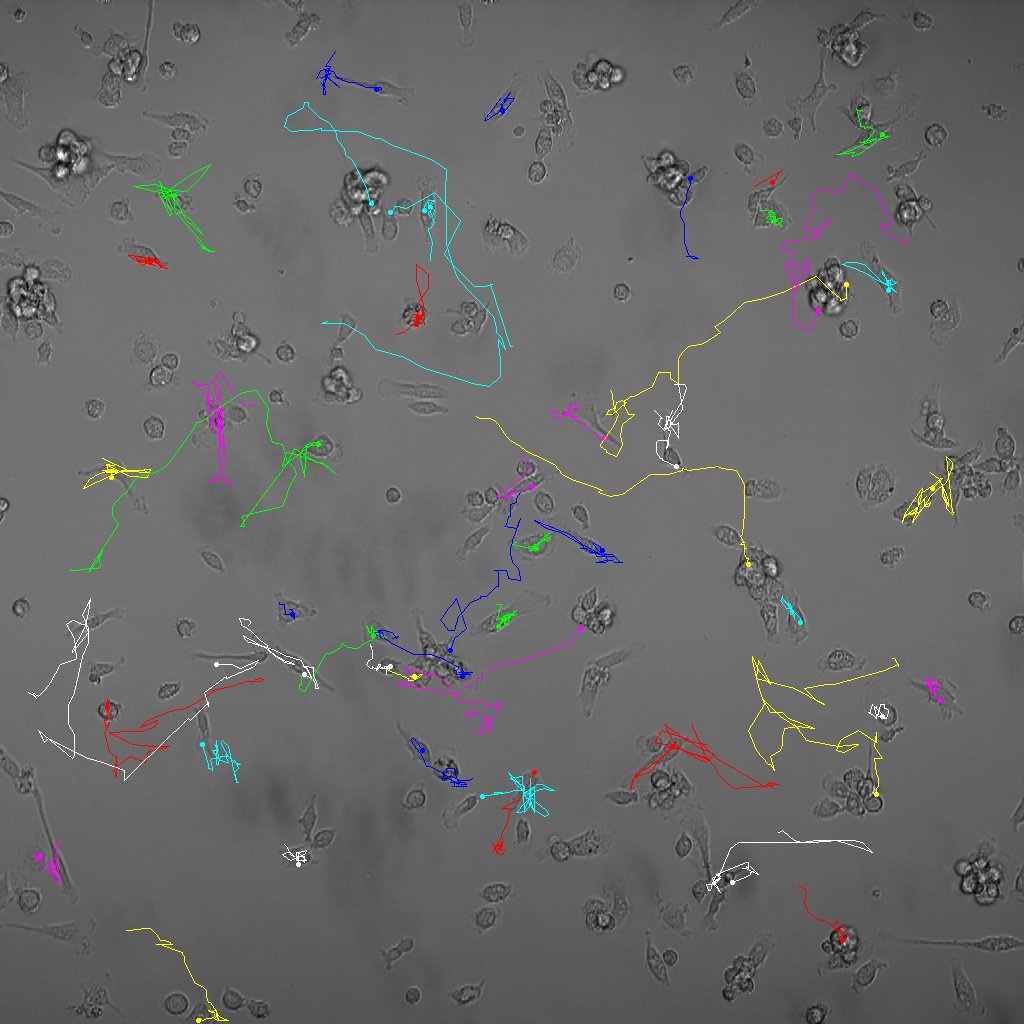

Supplement: Supplementary file 8 — Source data Fig. 4 [file 44318_2024_244_MOESM8_ESM.zip › Figure 4/4G/WT PMs Vehicle in mVCAM Fc.jpg]

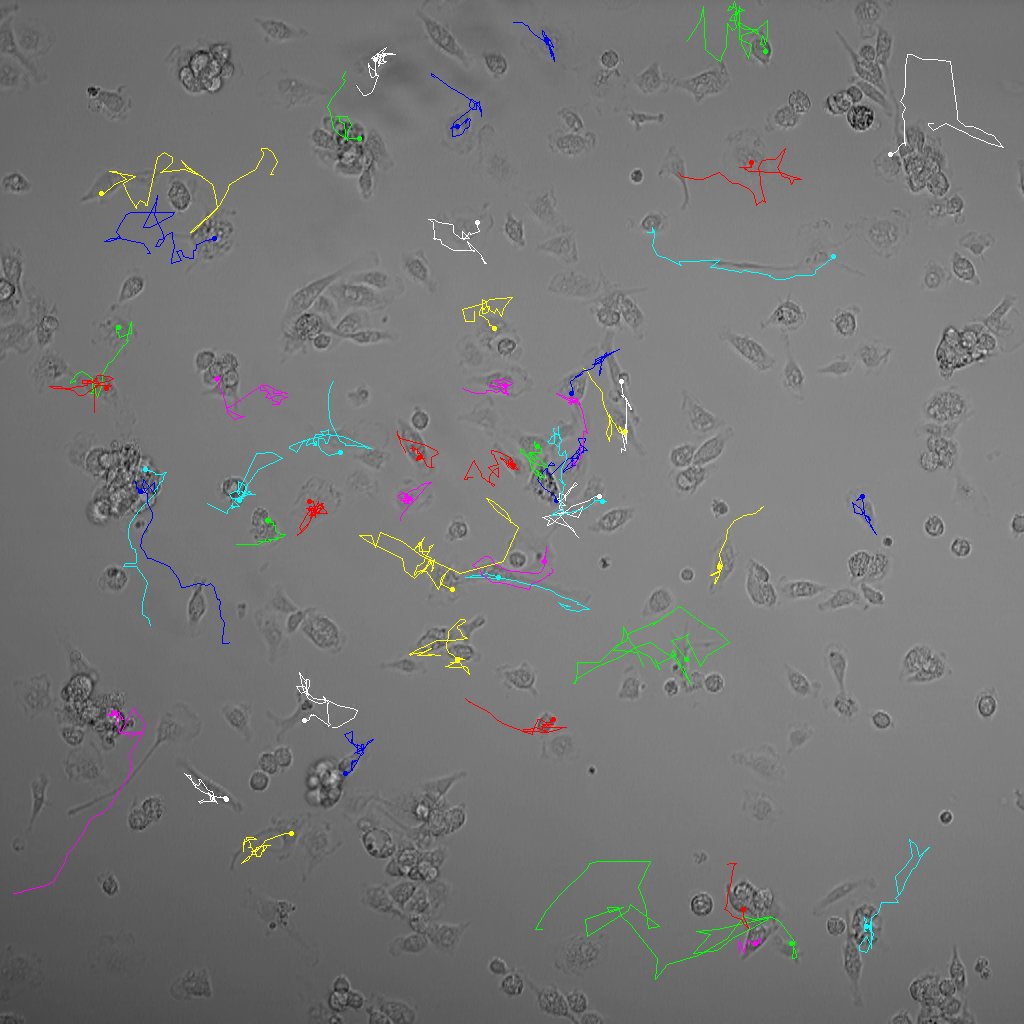

Supplement: Supplementary file 8 — Source data Fig. 4 [file 44318_2024_244_MOESM8_ESM.zip › Figure 4/4G/Zyxin KO PMs DMXAA in mVCAM Fc.jpg]

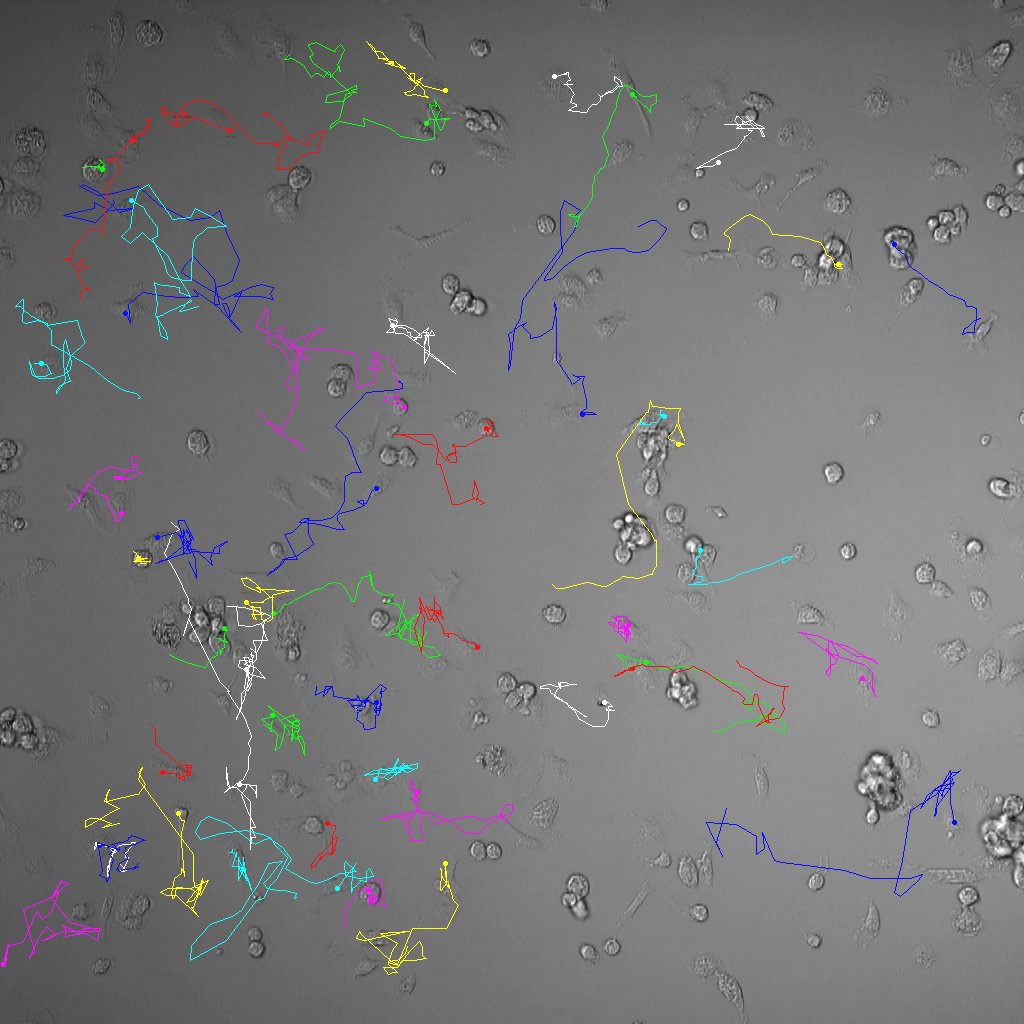

Supplement: Supplementary file 8 — Source data Fig. 4 [file 44318_2024_244_MOESM8_ESM.zip › Figure 4/4G/Zyxin KO PMs Vehicle in mVCAM Fc.jpg]

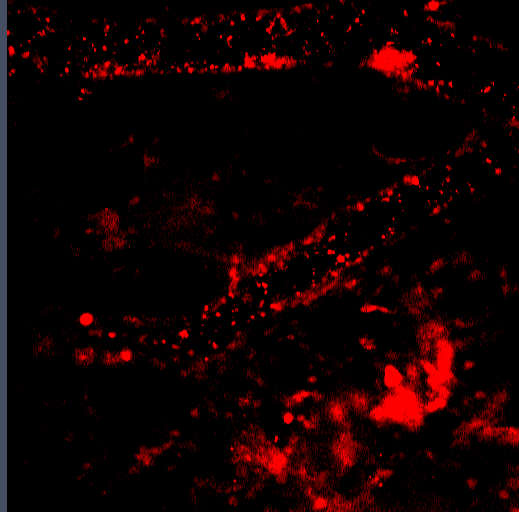

Supplement: Supplementary file 8 — Source data Fig. 4 [file 44318_2024_244_MOESM8_ESM.zip › Figure 4/4I/WT cGAMP Leukocyte.tif]

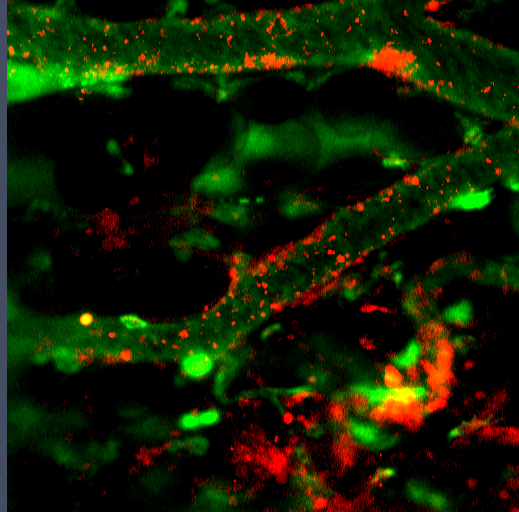

Supplement: Supplementary file 8 — Source data Fig. 4 [file 44318_2024_244_MOESM8_ESM.zip › Figure 4/4I/WT cGAMP Merge.tif]

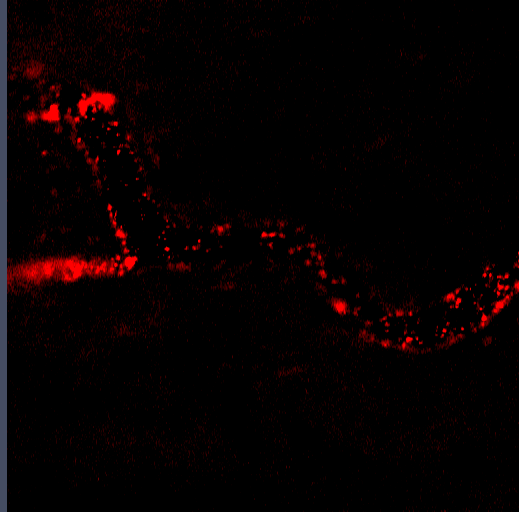

Supplement: Supplementary file 8 — Source data Fig. 4 [file 44318_2024_244_MOESM8_ESM.zip › Figure 4/4I/WT Vehicle Leukocyte.tif]

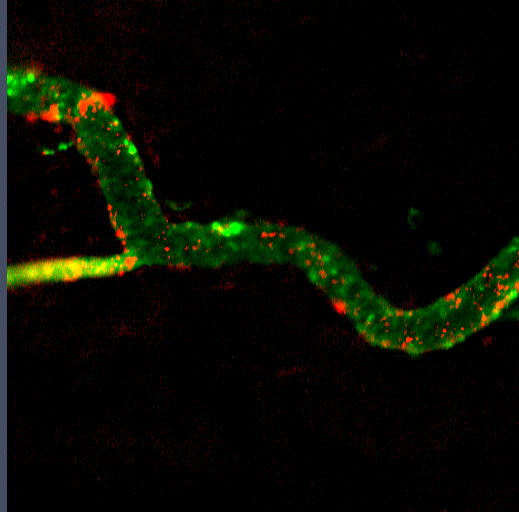

Supplement: Supplementary file 8 — Source data Fig. 4 [file 44318_2024_244_MOESM8_ESM.zip › Figure 4/4I/WT Vehicle Merge.tif]

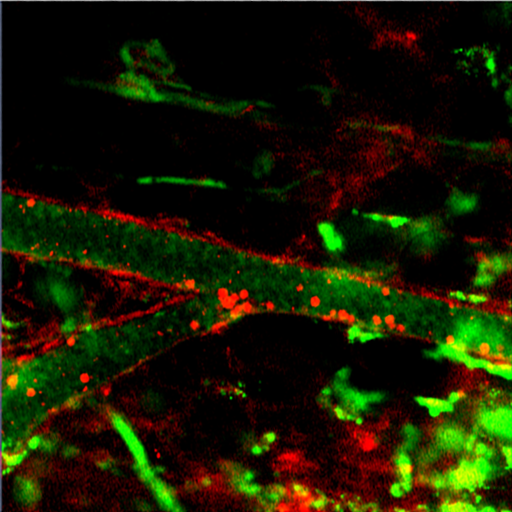

Supplement: Supplementary file 8 — Source data Fig. 4 [file 44318_2024_244_MOESM8_ESM.zip › Figure 4/4I/Zyxin KO cGAMP Merge.tif]

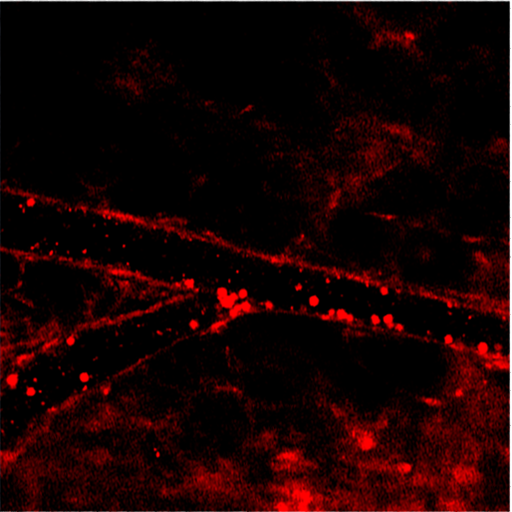

Supplement: Supplementary file 8 — Source data Fig. 4 [file 44318_2024_244_MOESM8_ESM.zip › Figure 4/4I/Zyxin KO cGAMP Leukocyte.tif]

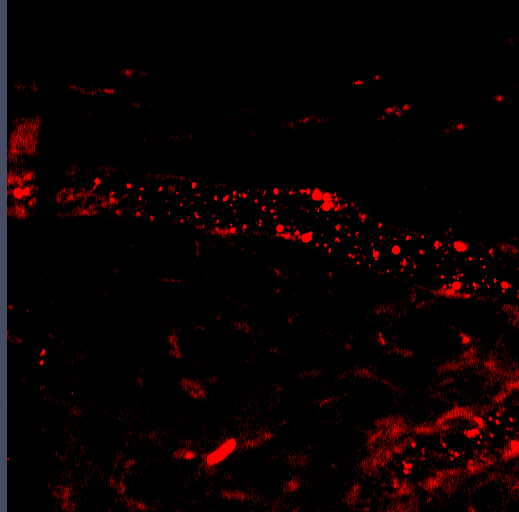

Supplement: Supplementary file 8 — Source data Fig. 4 [file 44318_2024_244_MOESM8_ESM.zip › Figure 4/4I/Zyxin KO Vehicle Leukocyte.tif]

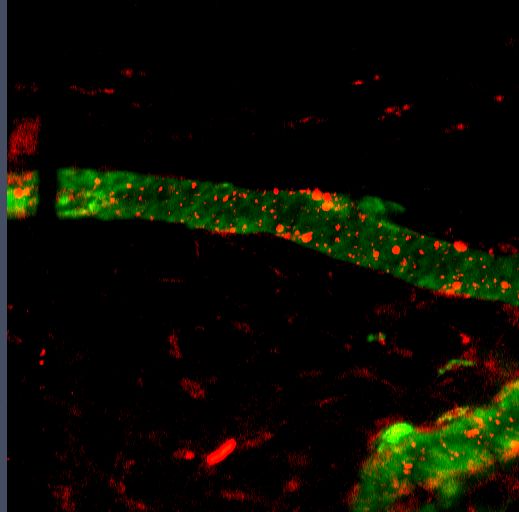

Supplement: Supplementary file 8 — Source data Fig. 4 [file 44318_2024_244_MOESM8_ESM.zip › Figure 4/4I/Zyxin KO Vehicle Merge.tif]

**Figure 5**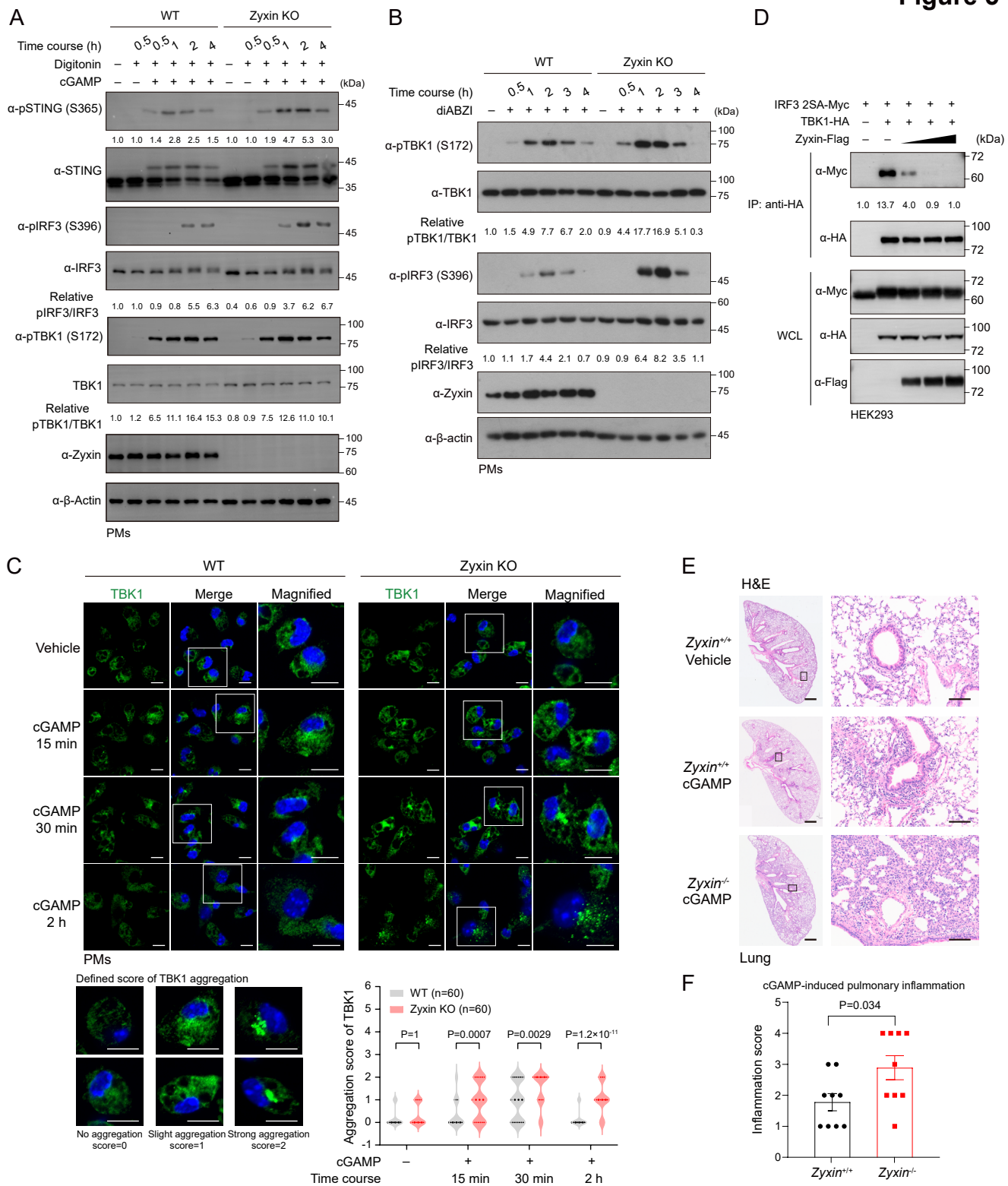

Supplement: Supplementary file 9 — Source data Fig. 5 [file 44318_2024_244_MOESM9_ESM.zip › Figure 5.pdf]

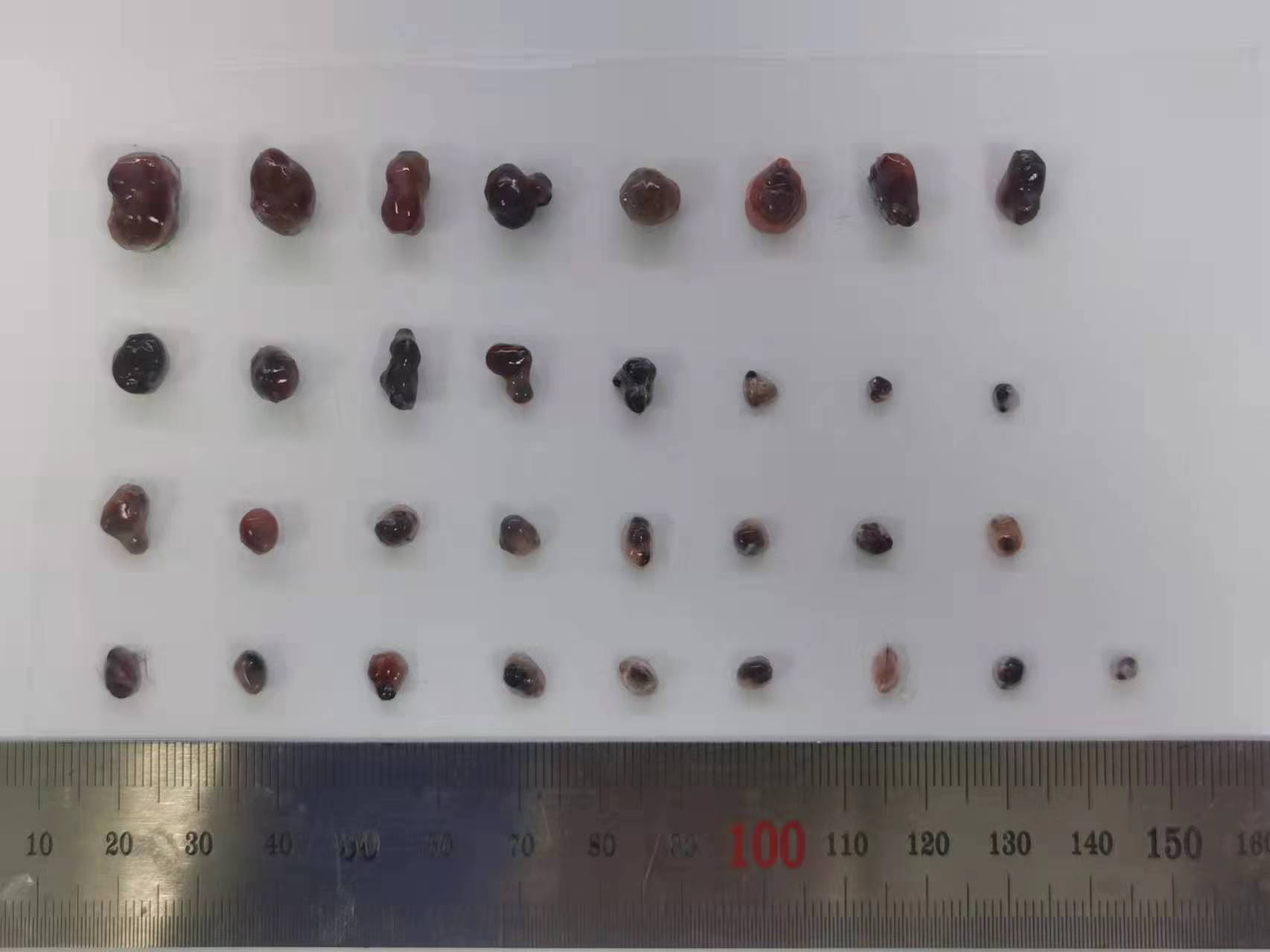

Supplement: Supplementary file 10 — Source data Fig. 6 [file 44318_2024_244_MOESM10_ESM.zip › Figure 6/6D/6D B16-F10 Inoculation.tif]

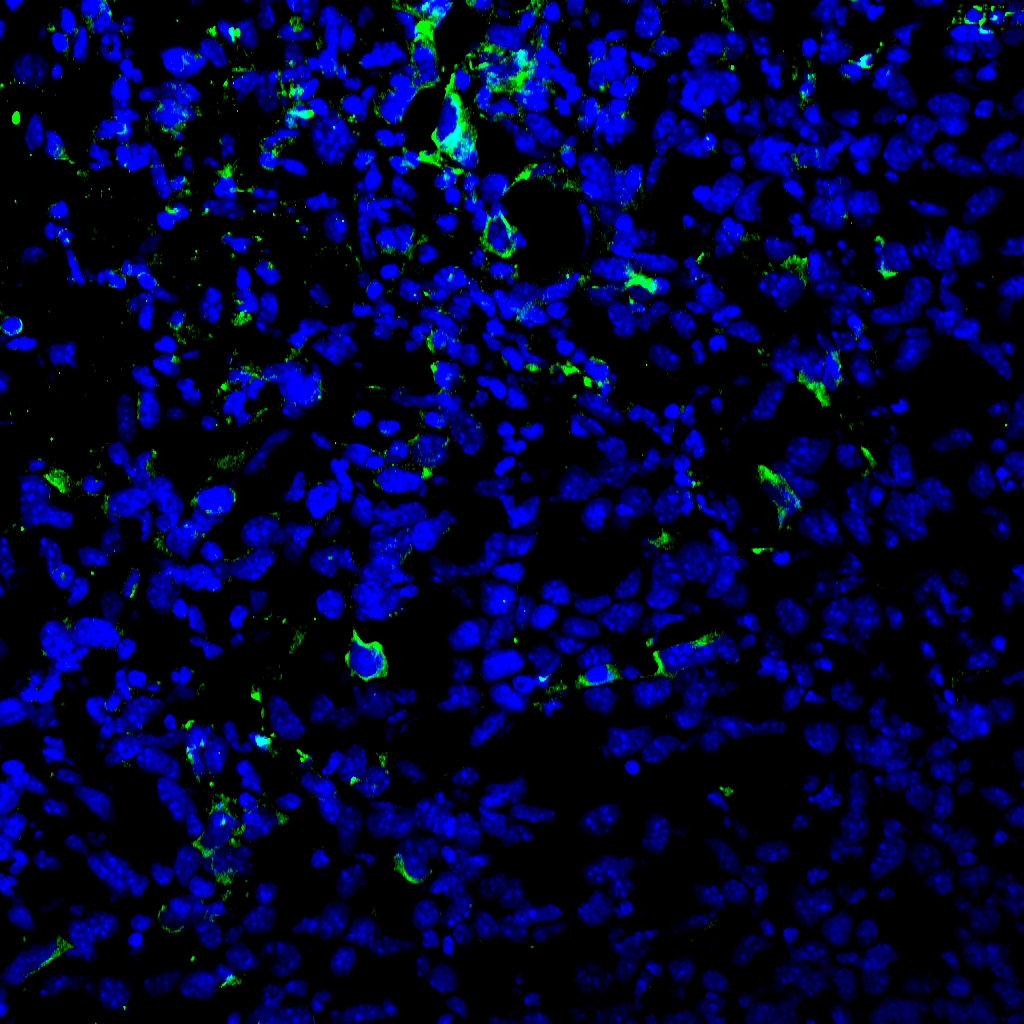

Supplement: Supplementary file 10 — Source data Fig. 6 [file 44318_2024_244_MOESM10_ESM.zip › Figure 6/6E/B16 WT cGAMP CD4 Merge.tif]

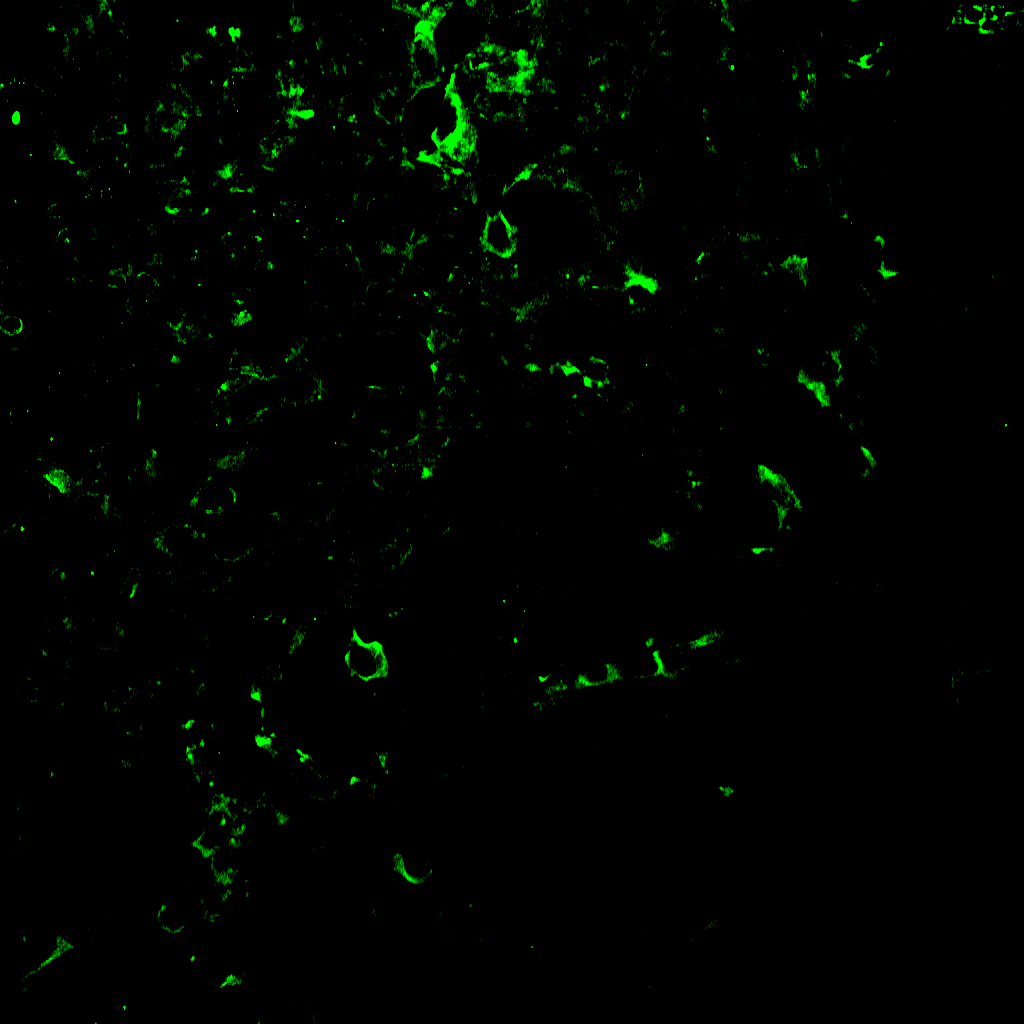

Supplement: Supplementary file 10 — Source data Fig. 6 [file 44318_2024_244_MOESM10_ESM.zip › Figure 6/6E/B16 WT cGAMP CD4.tif]

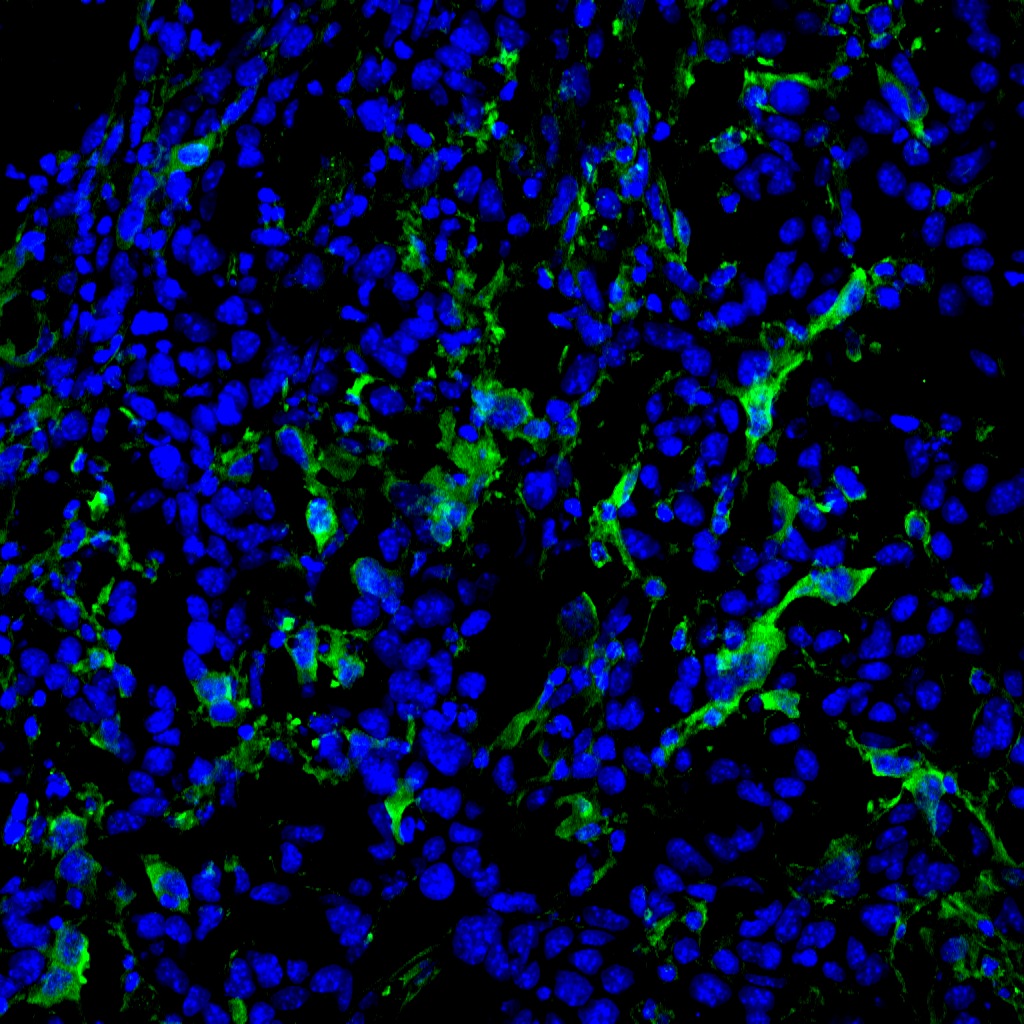

Supplement: Supplementary file 10 — Source data Fig. 6 [file 44318_2024_244_MOESM10_ESM.zip › Figure 6/6E/B16 WT cGAMP CD8 Merge.tif]

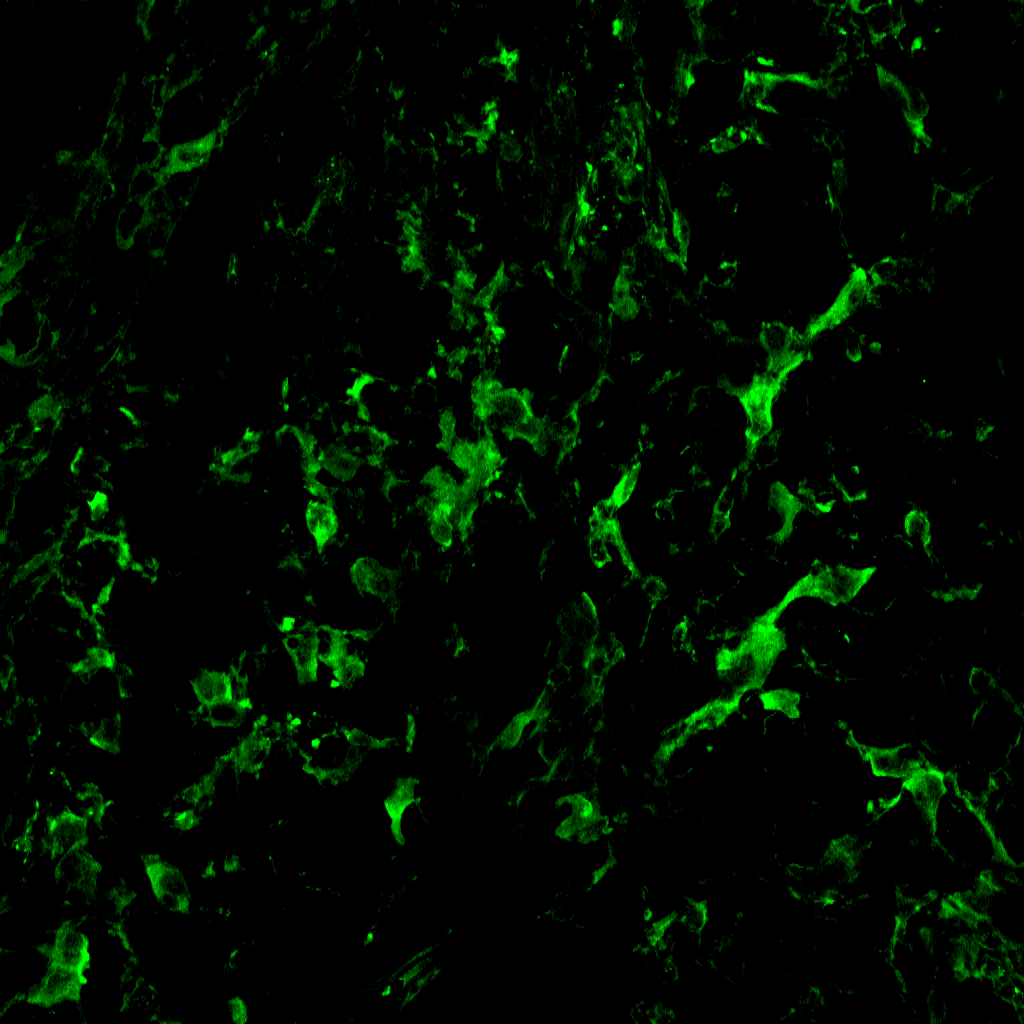

Supplement: Supplementary file 10 — Source data Fig. 6 [file 44318_2024_244_MOESM10_ESM.zip › Figure 6/6E/B16 WT cGAMP CD8.tif]

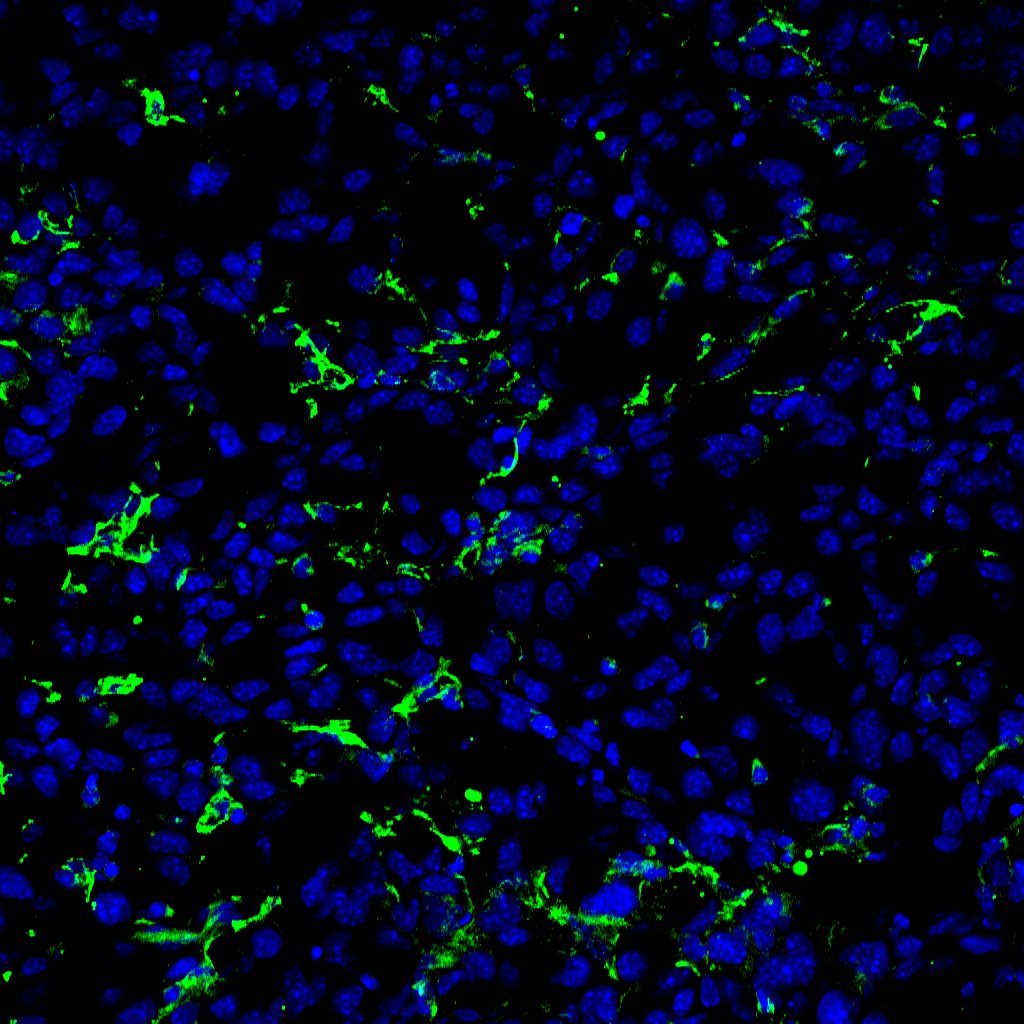

Supplement: Supplementary file 10 — Source data Fig. 6 [file 44318_2024_244_MOESM10_ESM.zip › Figure 6/6E/B16 WT cGAMP F4 80 Merge.tif]

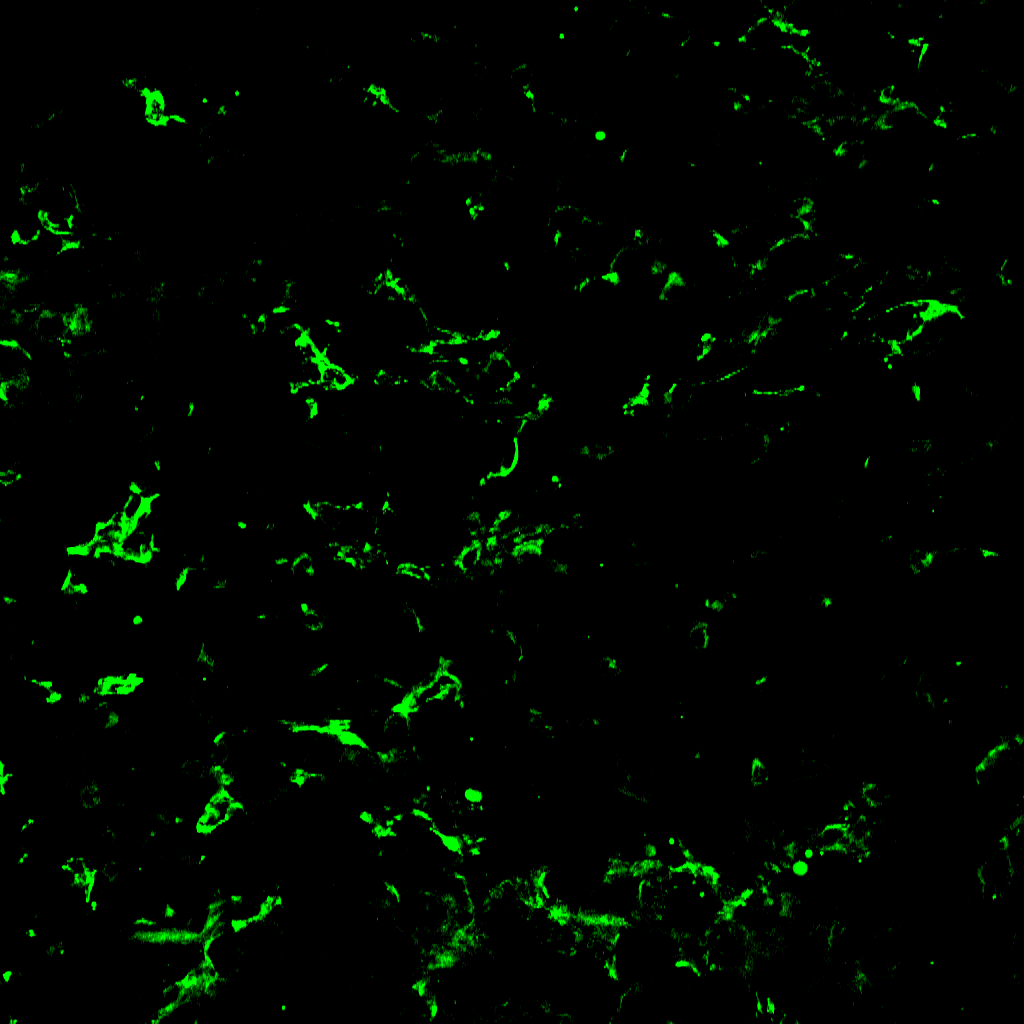

Supplement: Supplementary file 10 — Source data Fig. 6 [file 44318_2024_244_MOESM10_ESM.zip › Figure 6/6E/B16 WT cGAMP F4 80.tif]

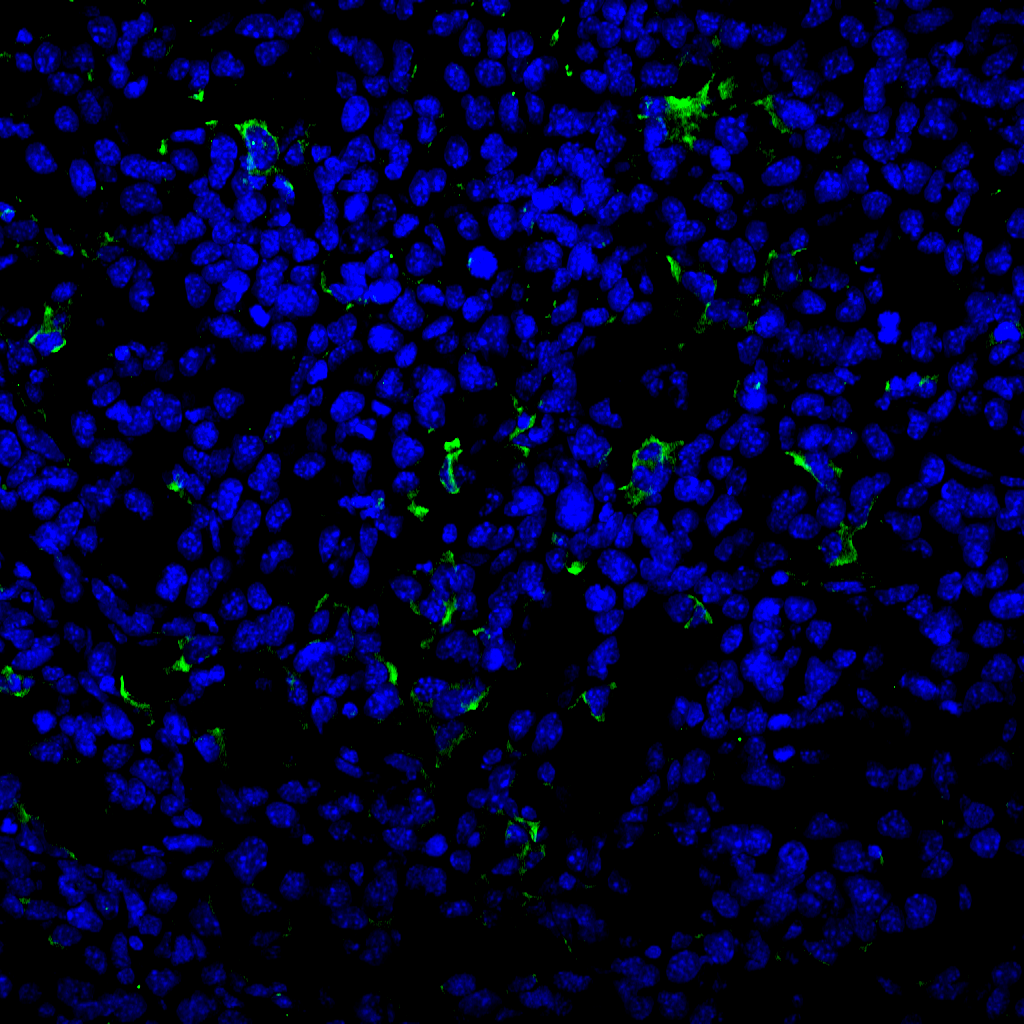

Supplement: Supplementary file 10 — Source data Fig. 6 [file 44318_2024_244_MOESM10_ESM.zip › Figure 6/6E/B16 WT ctrl CD4 Merge.tif]

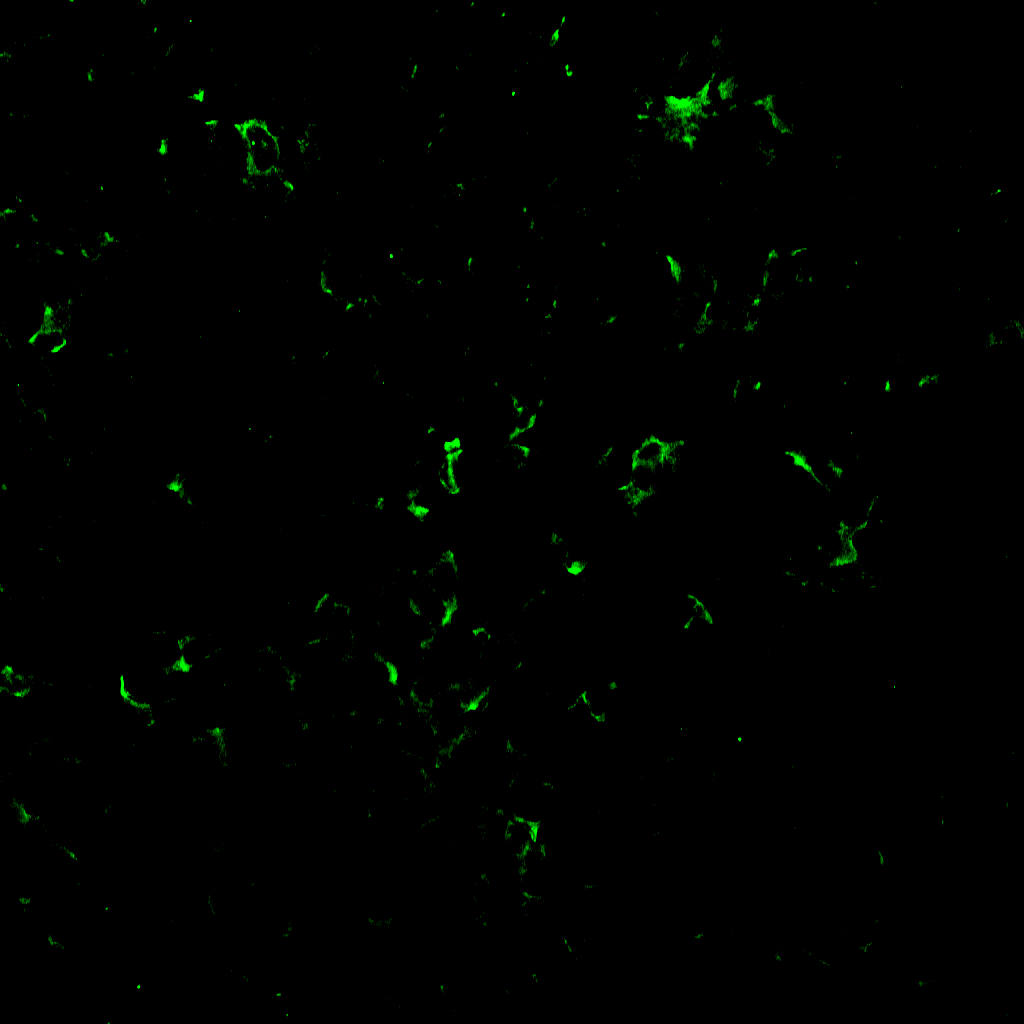

Supplement: Supplementary file 10 — Source data Fig. 6 [file 44318_2024_244_MOESM10_ESM.zip › Figure 6/6E/B16 WT Ctrl CD4.tif]

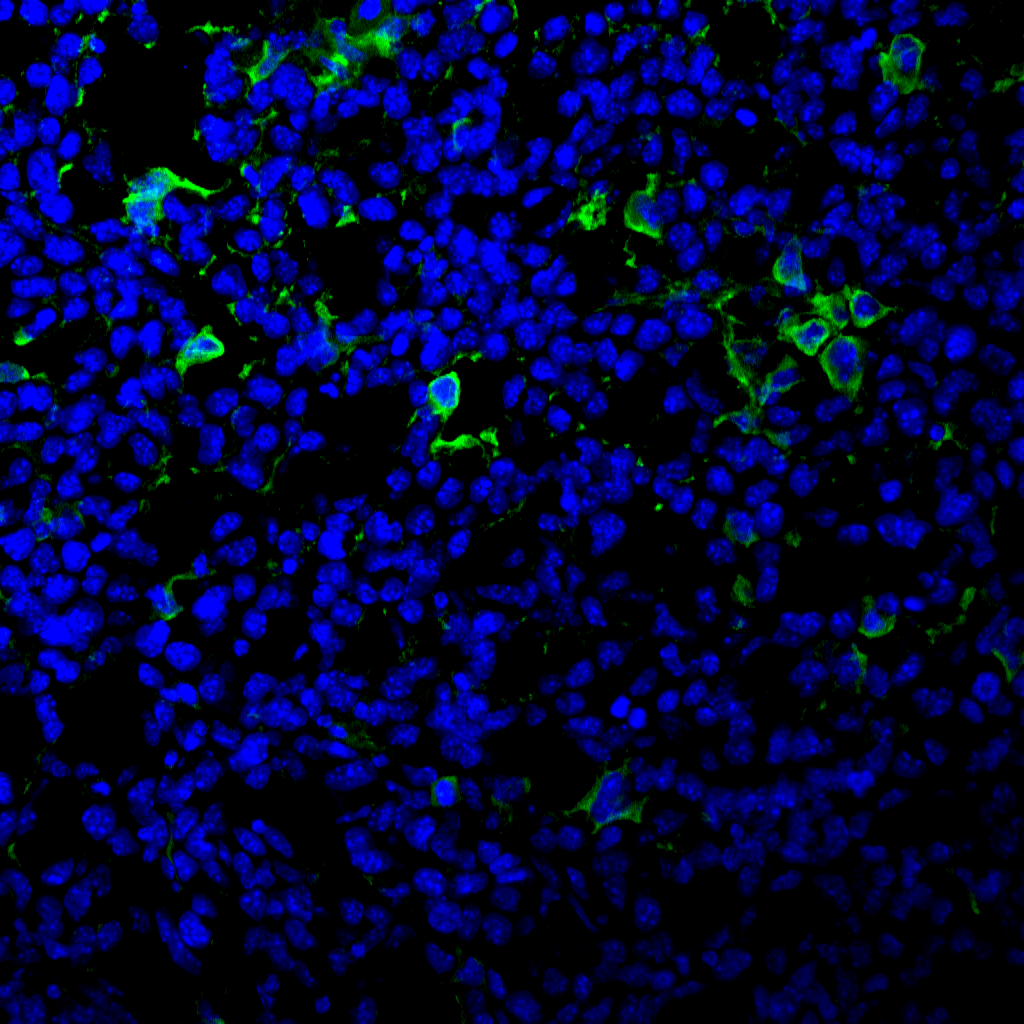

Supplement: Supplementary file 10 — Source data Fig. 6 [file 44318_2024_244_MOESM10_ESM.zip › Figure 6/6E/B16 WT Ctrl CD8 Merge.tif]

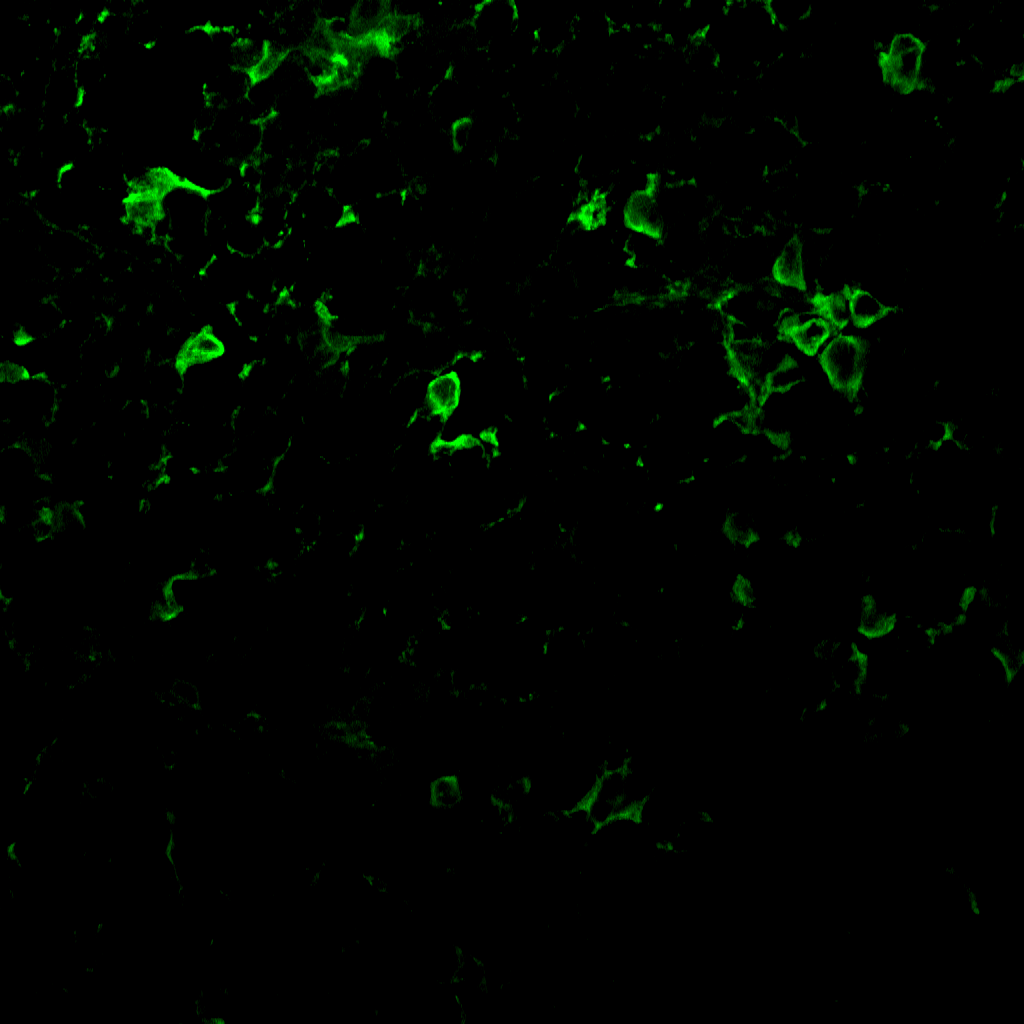

Supplement: Supplementary file 10 — Source data Fig. 6 [file 44318_2024_244_MOESM10_ESM.zip › Figure 6/6E/B16 WT Ctrl CD8.tif]

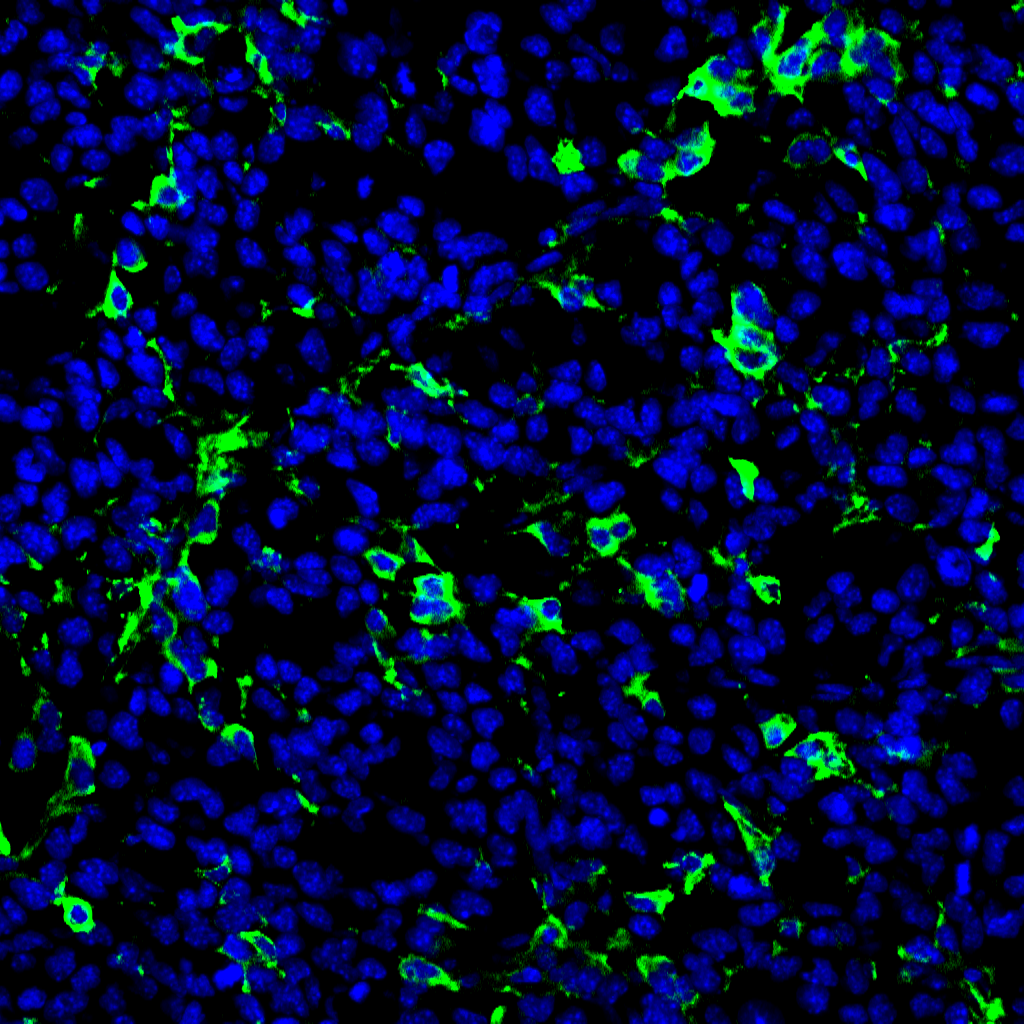

Supplement: Supplementary file 10 — Source data Fig. 6 [file 44318_2024_244_MOESM10_ESM.zip › Figure 6/6E/B16 WT Ctrl F4 80 Merge.tif]

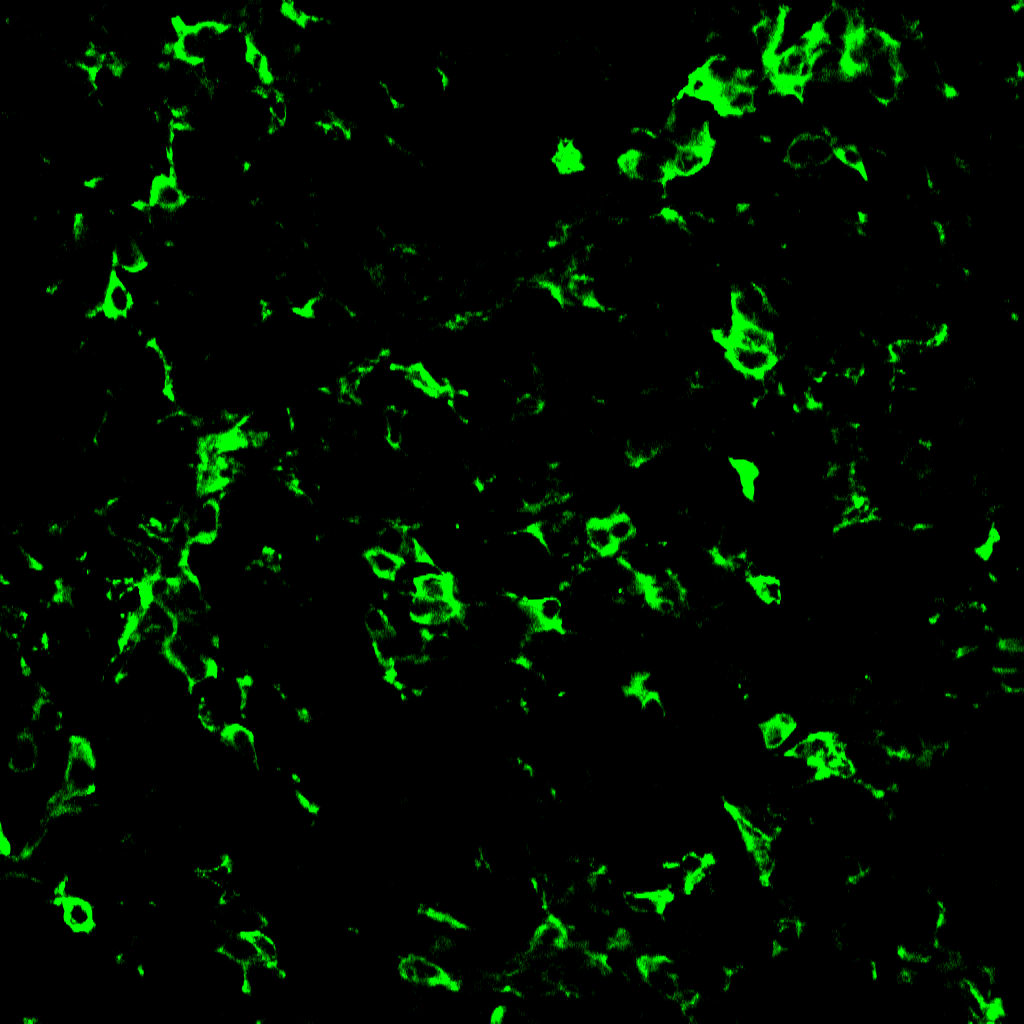

Supplement: Supplementary file 10 — Source data Fig. 6 [file 44318_2024_244_MOESM10_ESM.zip › Figure 6/6E/B16 WT Ctrl F4 80.tif]

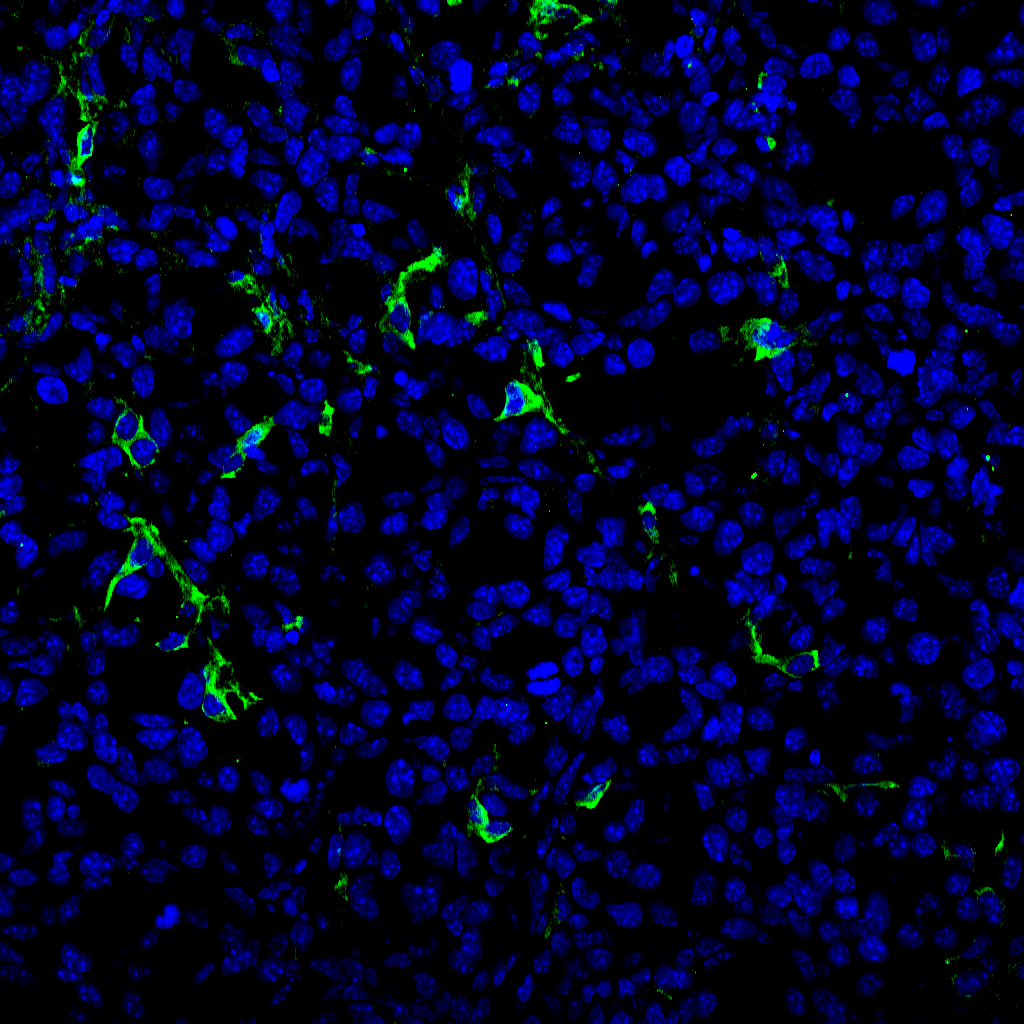

Supplement: Supplementary file 10 — Source data Fig. 6 [file 44318_2024_244_MOESM10_ESM.zip › Figure 6/6E/B16 Zyxin KO cGAMP CD4 Merge.tif]

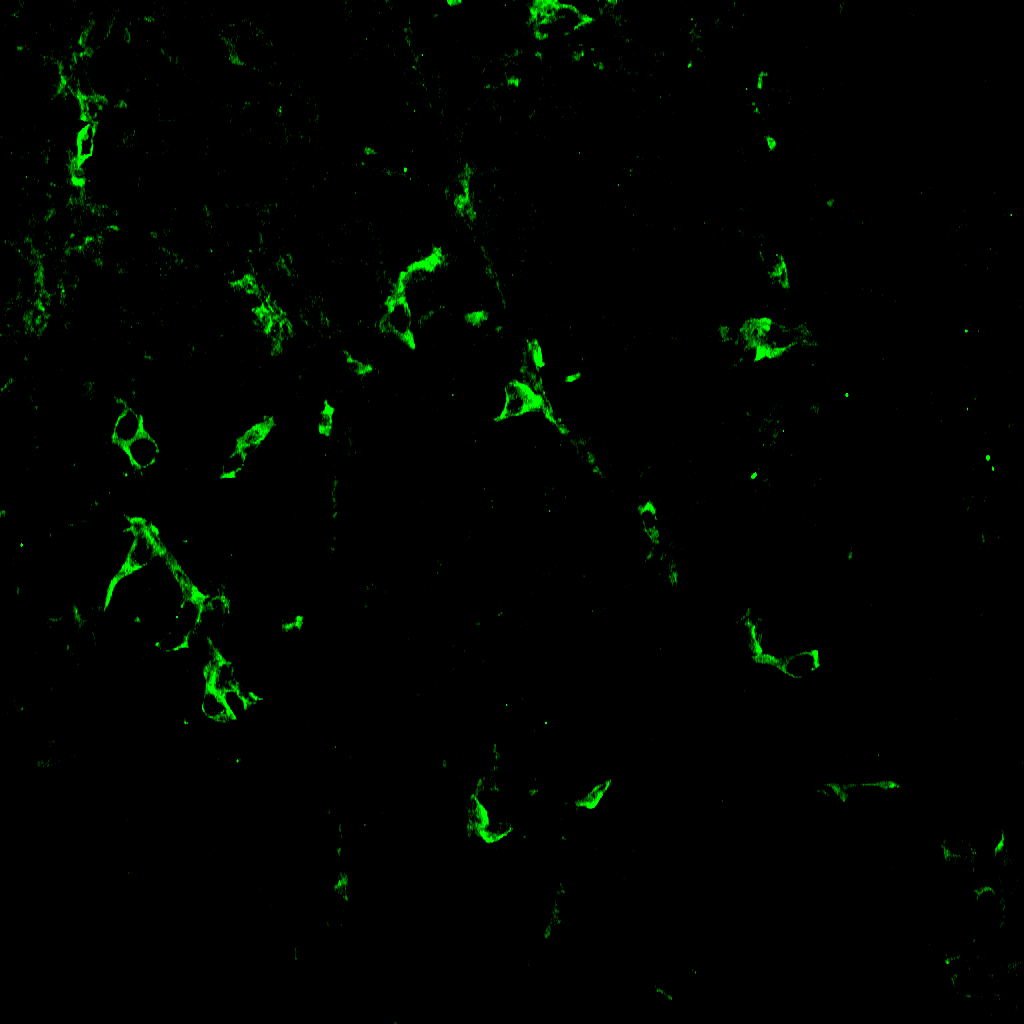

Supplement: Supplementary file 10 — Source data Fig. 6 [file 44318_2024_244_MOESM10_ESM.zip › Figure 6/6E/B16 Zyxin KO cGAMP CD4.tif]

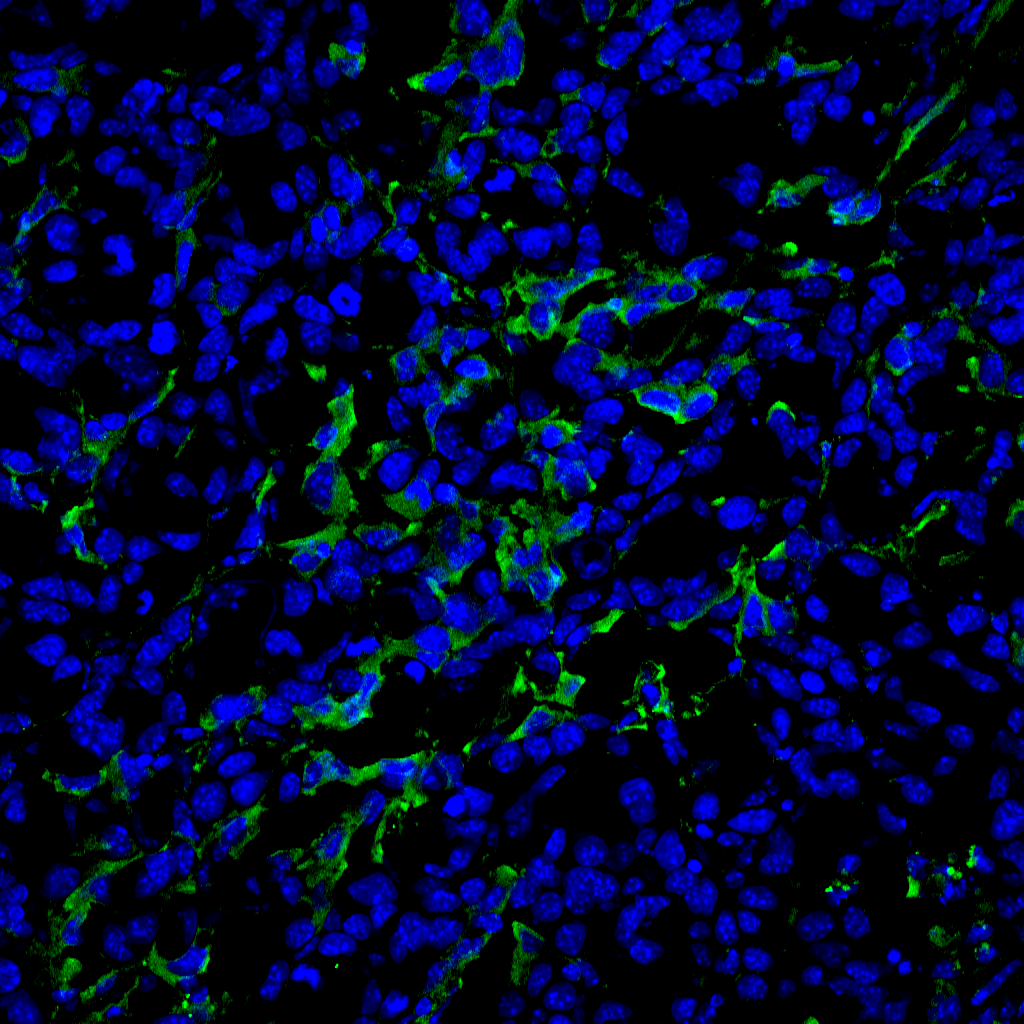

Supplement: Supplementary file 10 — Source data Fig. 6 [file 44318_2024_244_MOESM10_ESM.zip › Figure 6/6E/B16 Zyxin KO cGAMP CD8 Merge.tif]

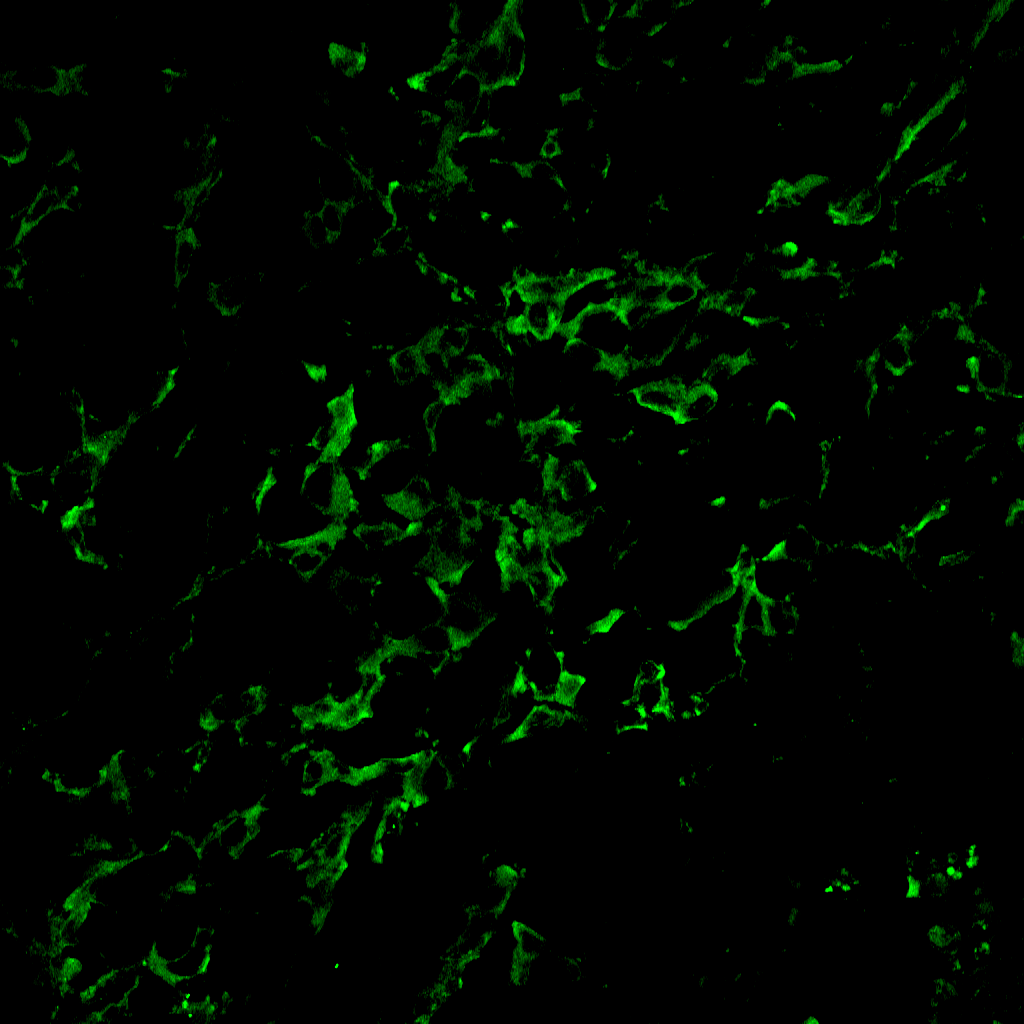

Supplement: Supplementary file 10 — Source data Fig. 6 [file 44318_2024_244_MOESM10_ESM.zip › Figure 6/6E/B16 Zyxin KO cGAMP CD8.tif]

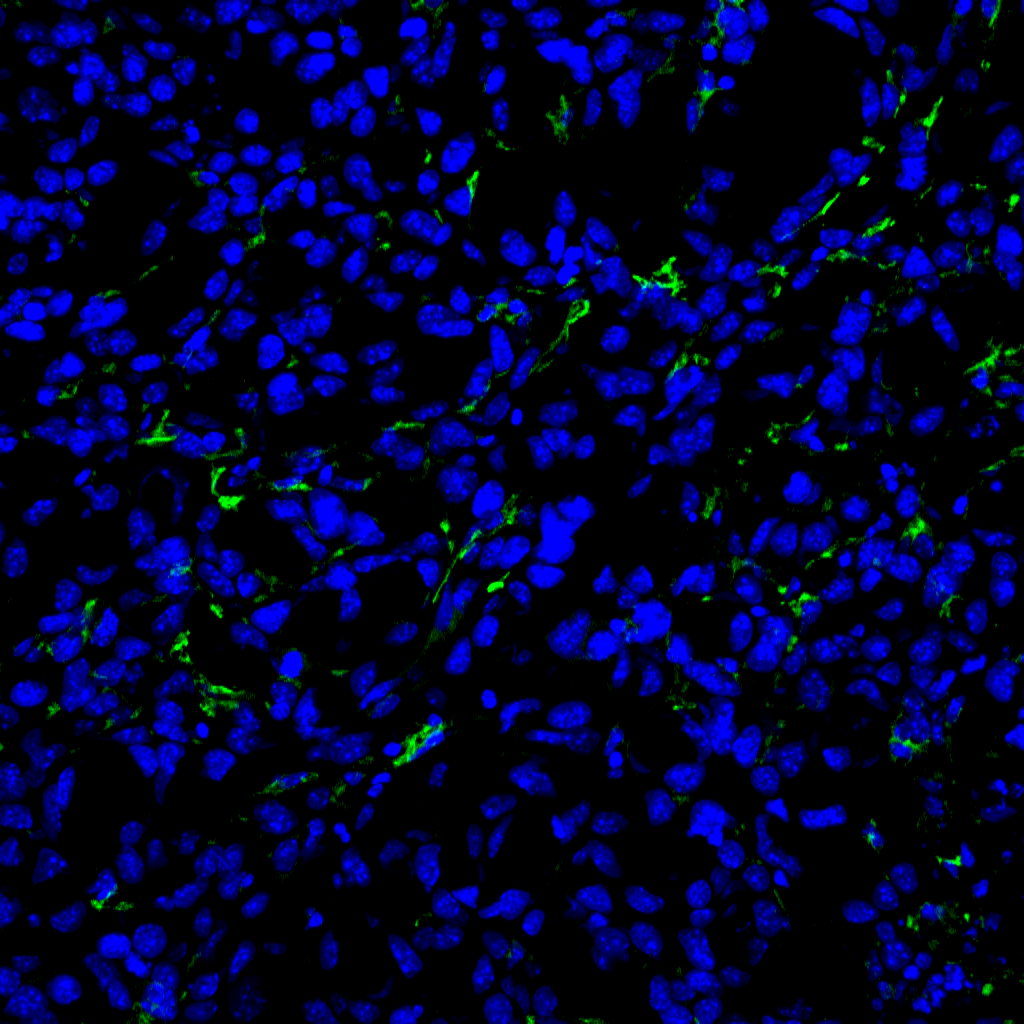

Supplement: Supplementary file 10 — Source data Fig. 6 [file 44318_2024_244_MOESM10_ESM.zip › Figure 6/6E/B16 Zyxin KO cGAMP F4 80 Merge.tif]

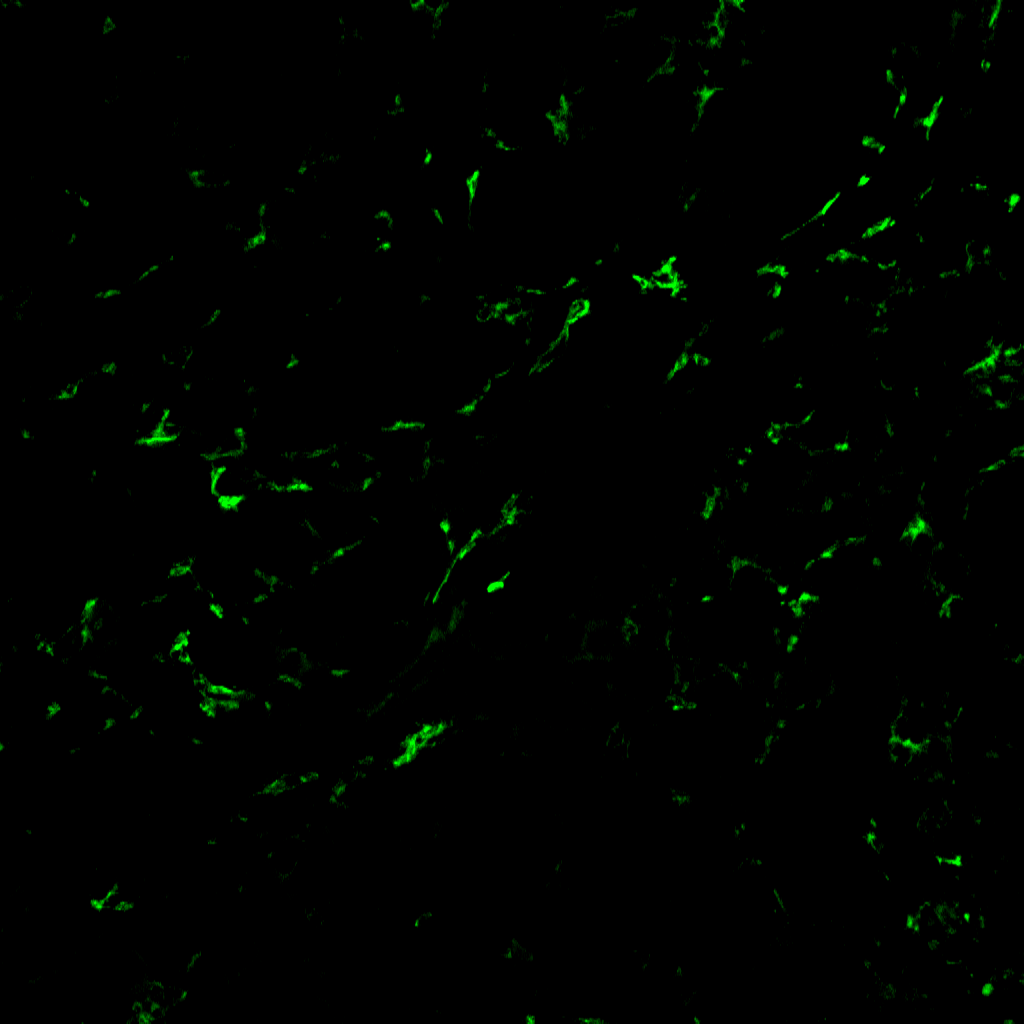

Supplement: Supplementary file 10 — Source data Fig. 6 [file 44318_2024_244_MOESM10_ESM.zip › Figure 6/6E/B16 Zyxin KO cGAMP F4 80.tif]

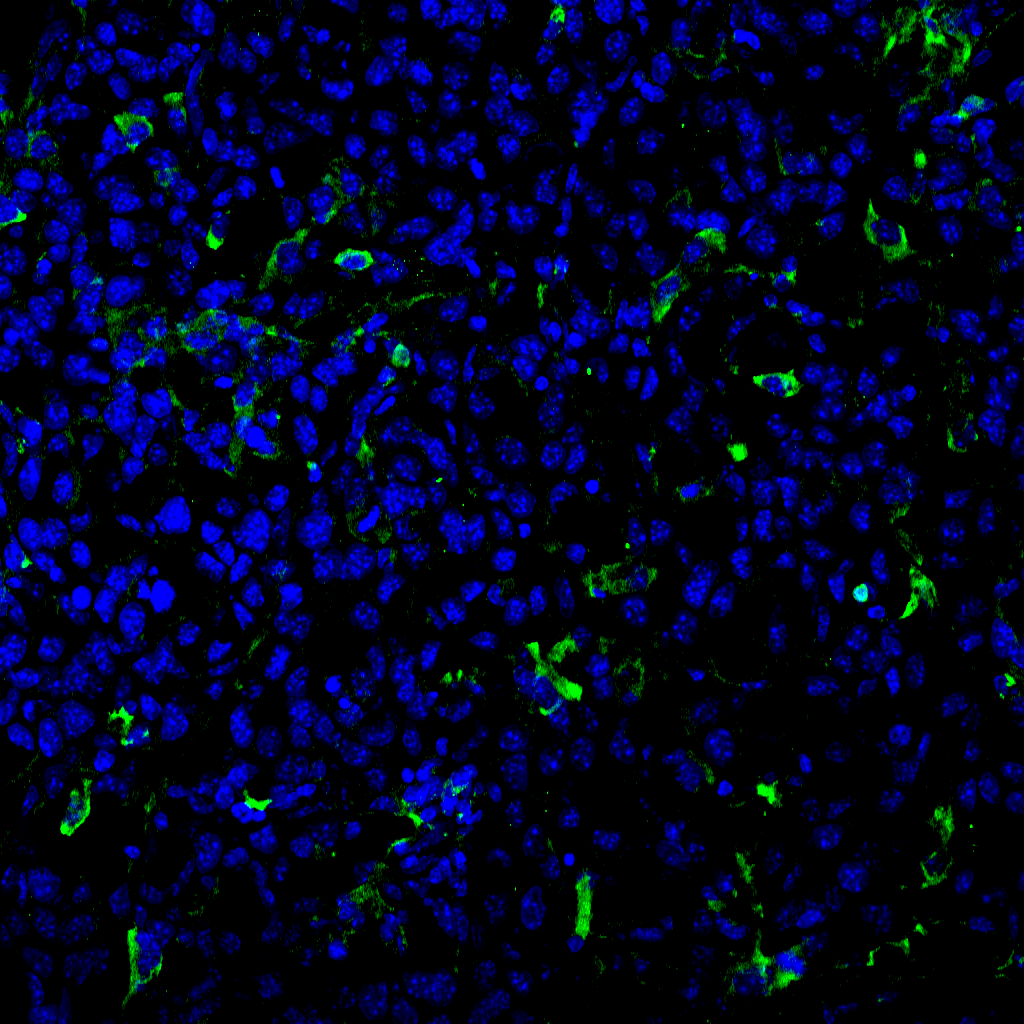

Supplement: Supplementary file 10 — Source data Fig. 6 [file 44318_2024_244_MOESM10_ESM.zip › Figure 6/6E/B16 Zyxin KO Ctrl CD4 Merge.tif]

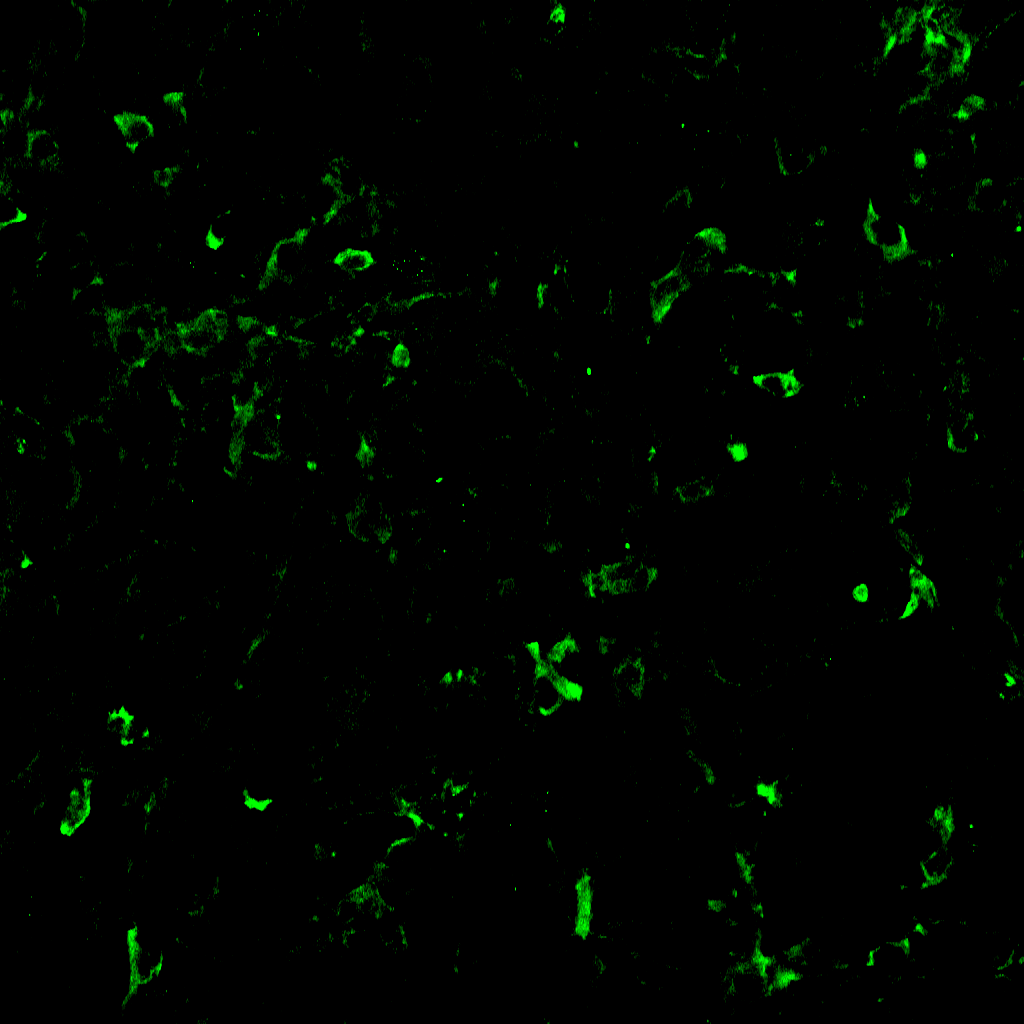

Supplement: Supplementary file 10 — Source data Fig. 6 [file 44318_2024_244_MOESM10_ESM.zip › Figure 6/6E/B16 Zyxin KO Ctrl CD4.tif]

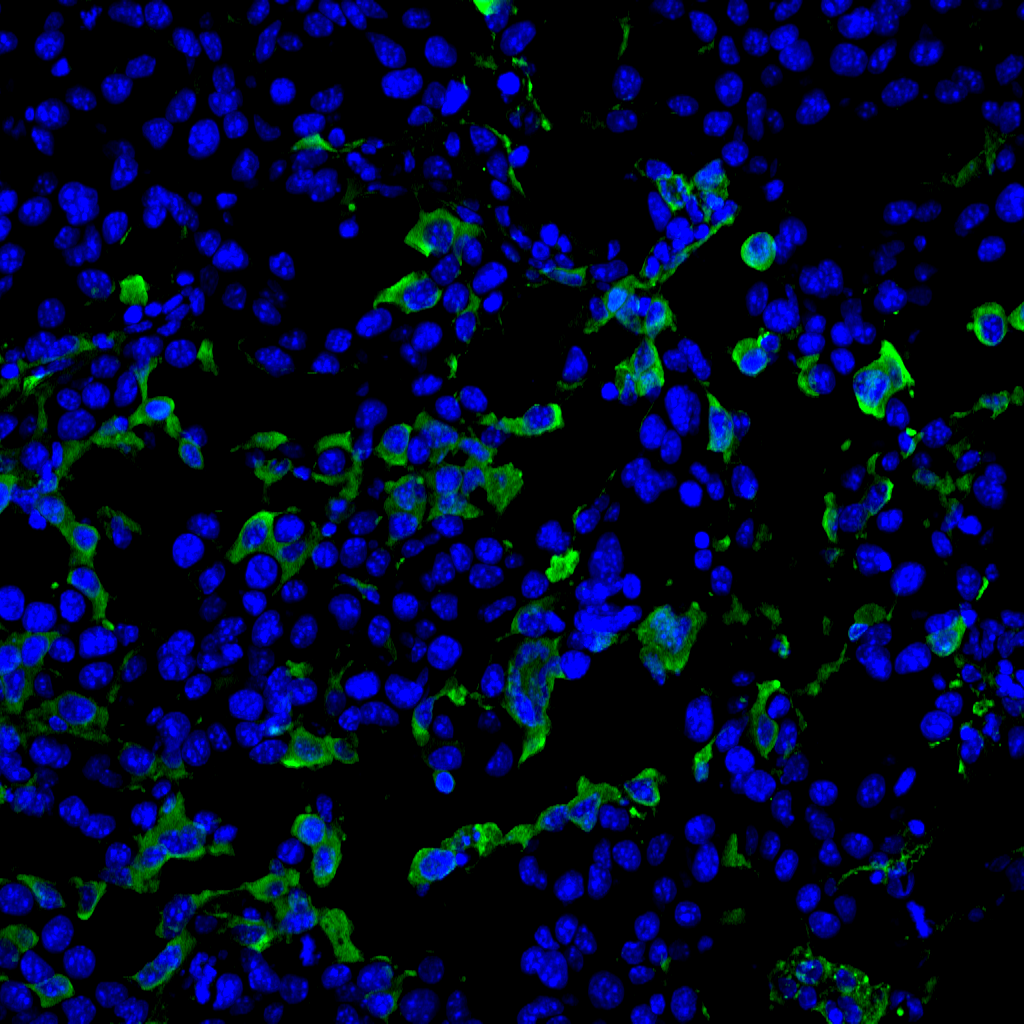

Supplement: Supplementary file 10 — Source data Fig. 6 [file 44318_2024_244_MOESM10_ESM.zip › Figure 6/6E/B16 Zyxin KO Ctrl CD8 Merge.tif]

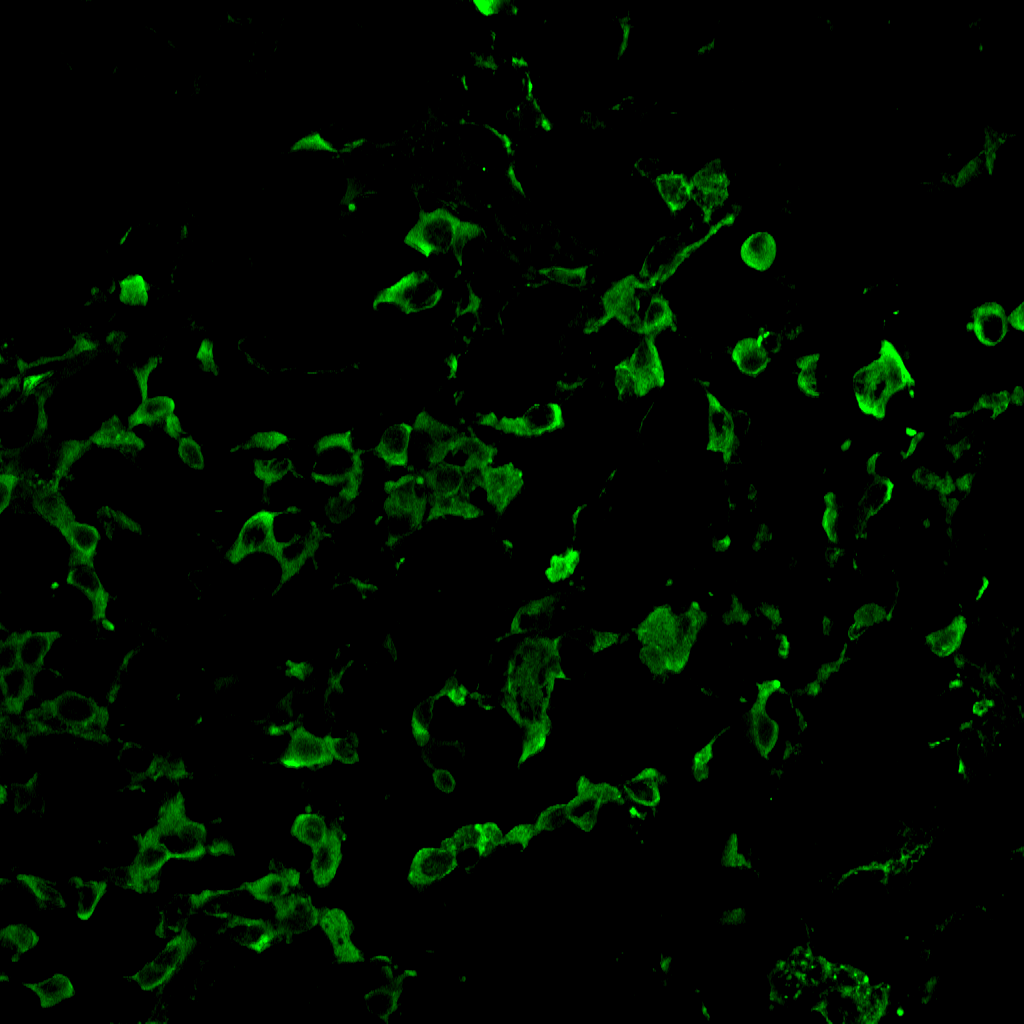

Supplement: Supplementary file 10 — Source data Fig. 6 [file 44318_2024_244_MOESM10_ESM.zip › Figure 6/6E/B16 Zyxin KO Ctrl CD8.tif]

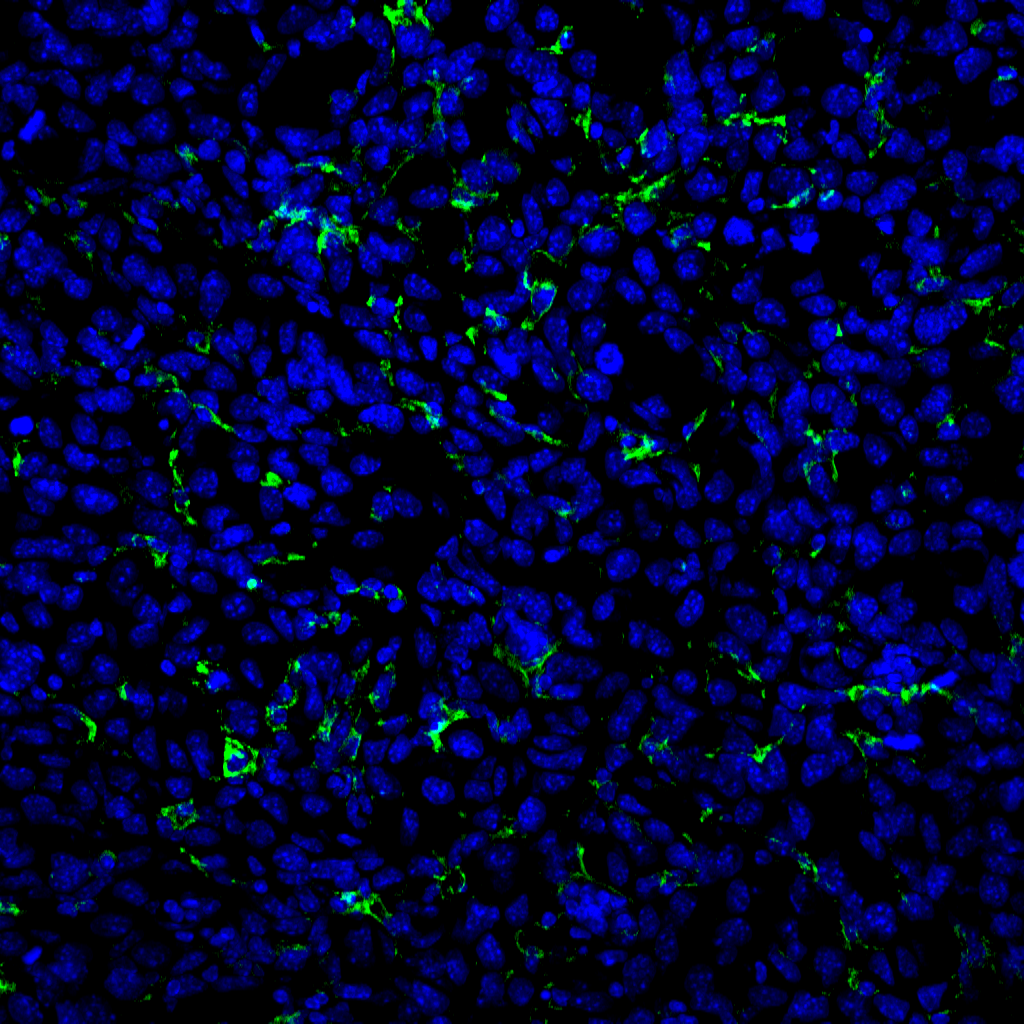

Supplement: Supplementary file 10 — Source data Fig. 6 [file 44318_2024_244_MOESM10_ESM.zip › Figure 6/6E/B16 Zyxin KO Ctrl F4 80 Merge.tif]

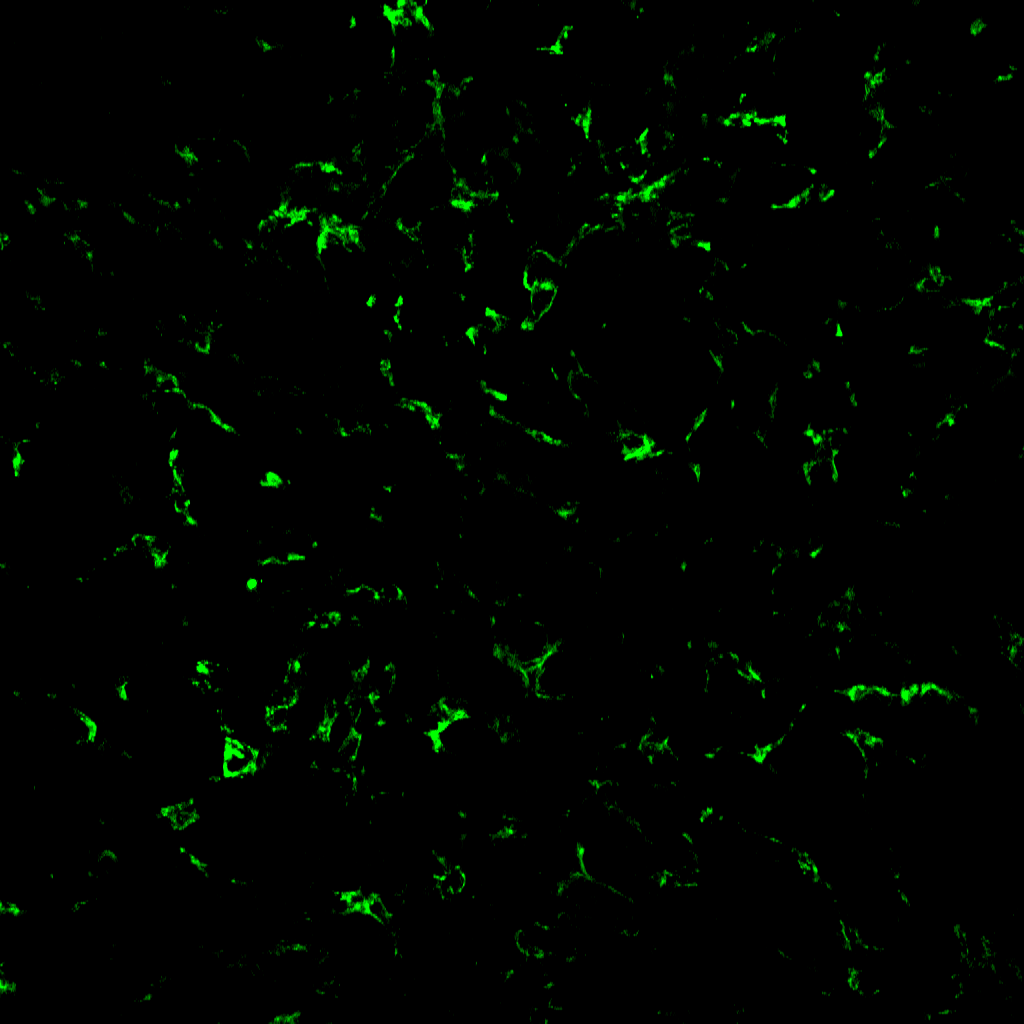

Supplement: Supplementary file 10 — Source data Fig. 6 [file 44318_2024_244_MOESM10_ESM.zip › Figure 6/6E/B16 Zyxin KO Ctrl F4 80.tif]

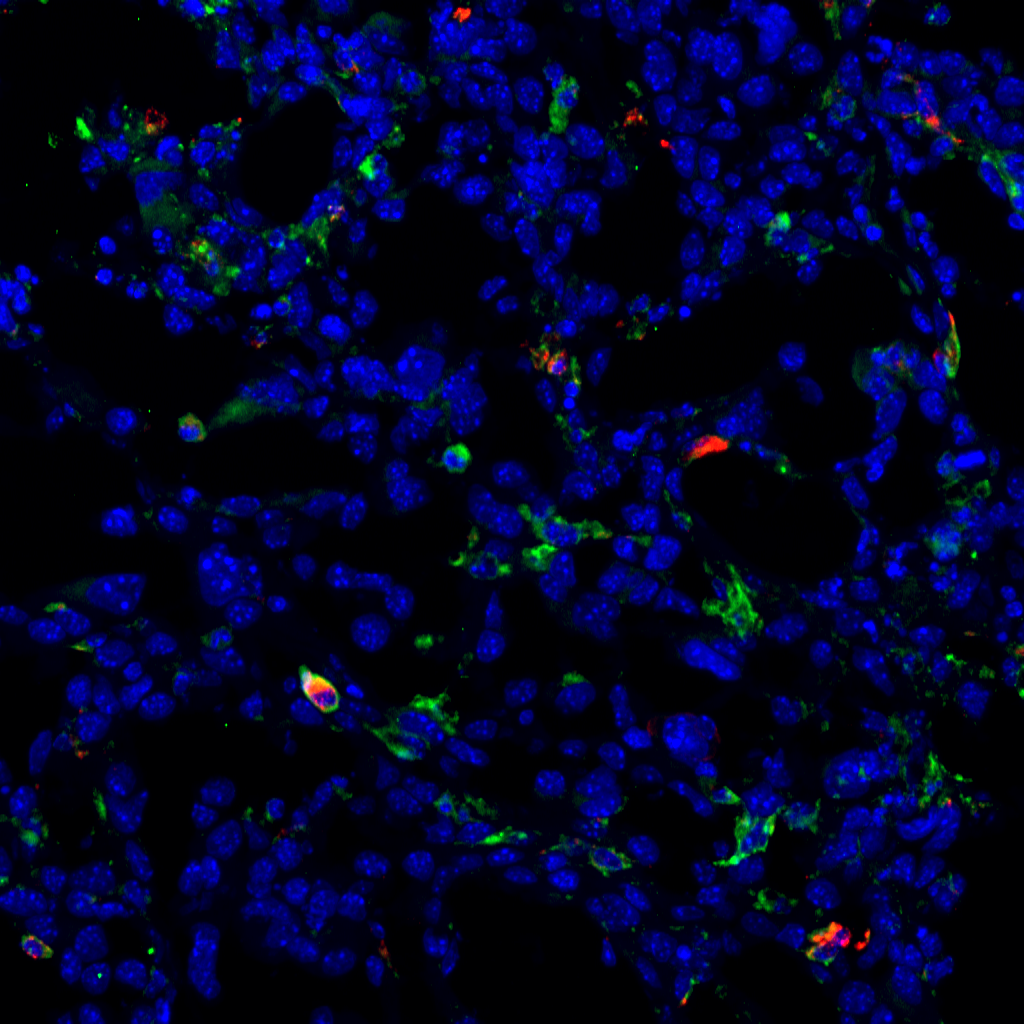

Supplement: Supplementary file 10 — Source data Fig. 6 [file 44318_2024_244_MOESM10_ESM.zip › Figure 6/6G/B16 WT cGAMP CD206 F4 80 Merge.tif]

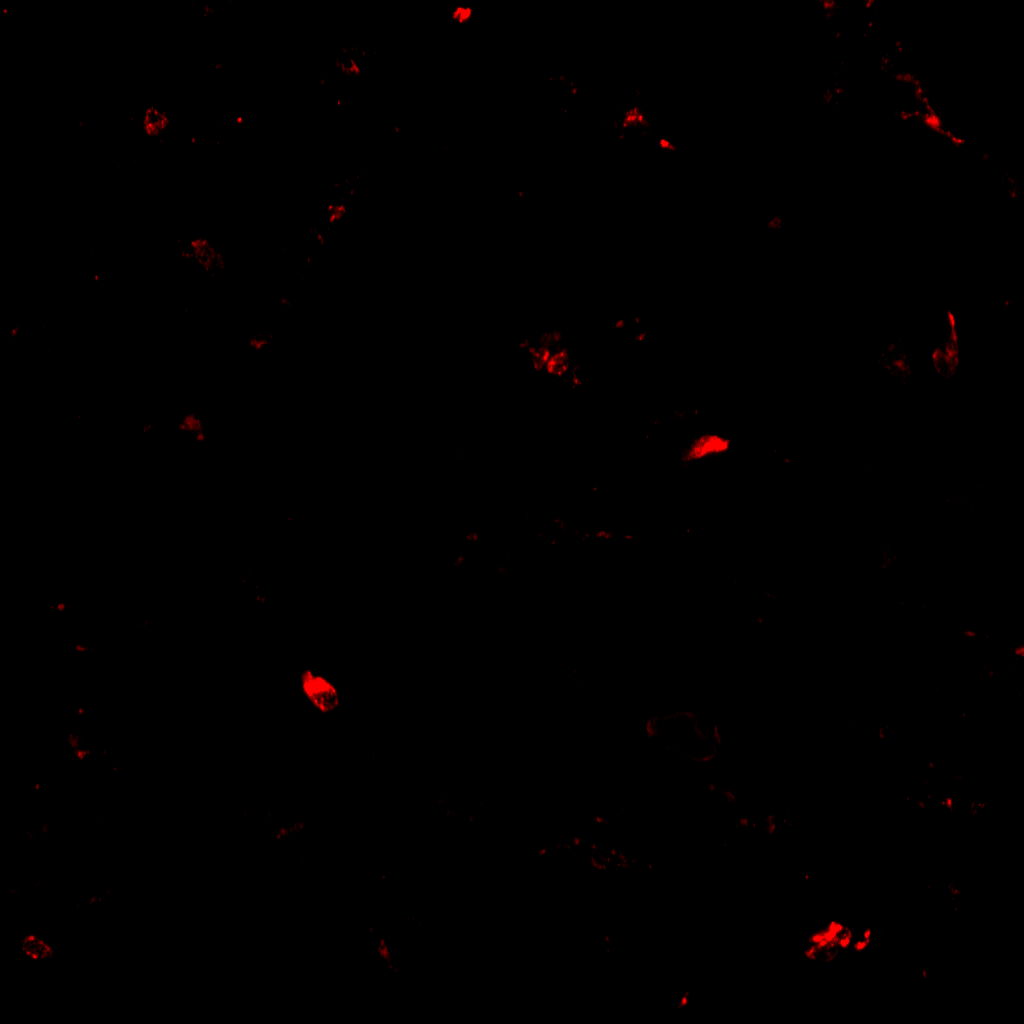

Supplement: Supplementary file 10 — Source data Fig. 6 [file 44318_2024_244_MOESM10_ESM.zip › Figure 6/6G/B16 WT cGAMP CD206.tif]

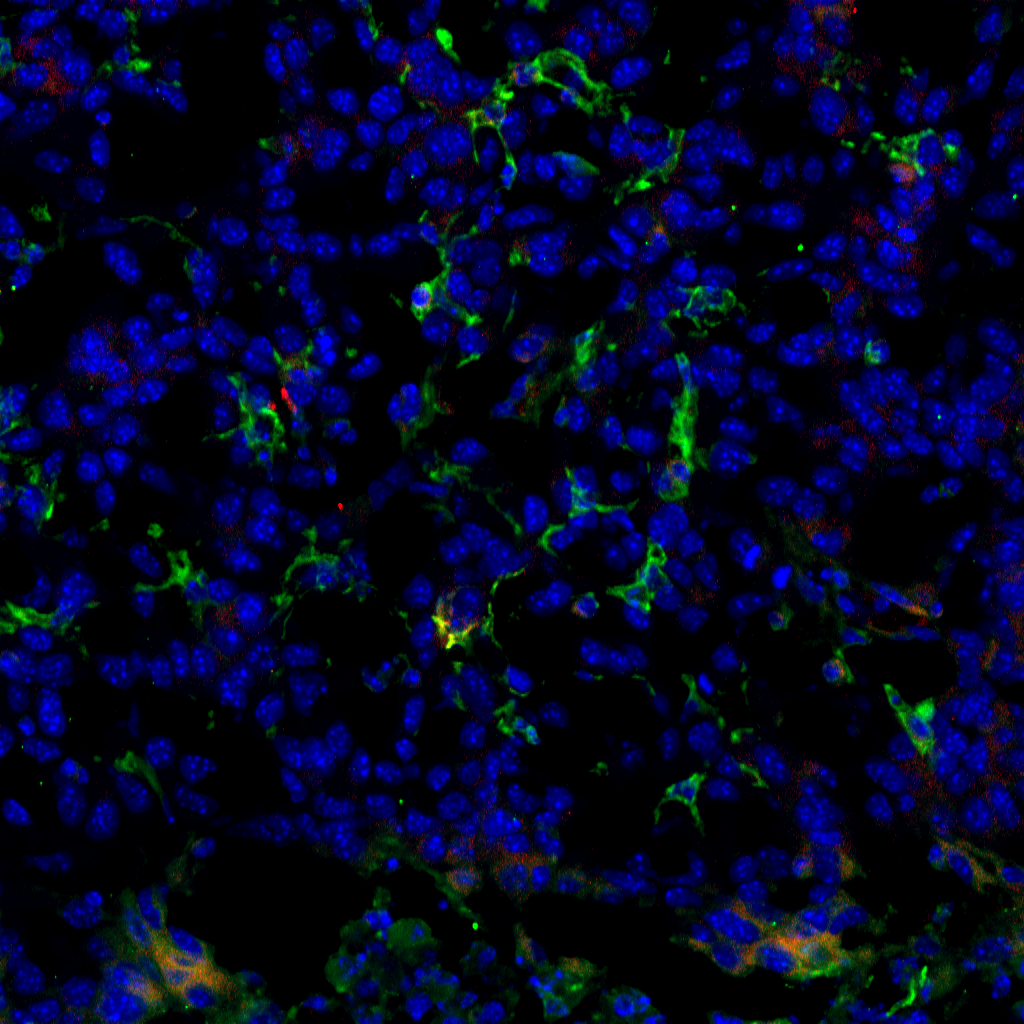

Supplement: Supplementary file 10 — Source data Fig. 6 [file 44318_2024_244_MOESM10_ESM.zip › Figure 6/6G/B16 WT cGAMP CD86 F4 80 Merge.tif]

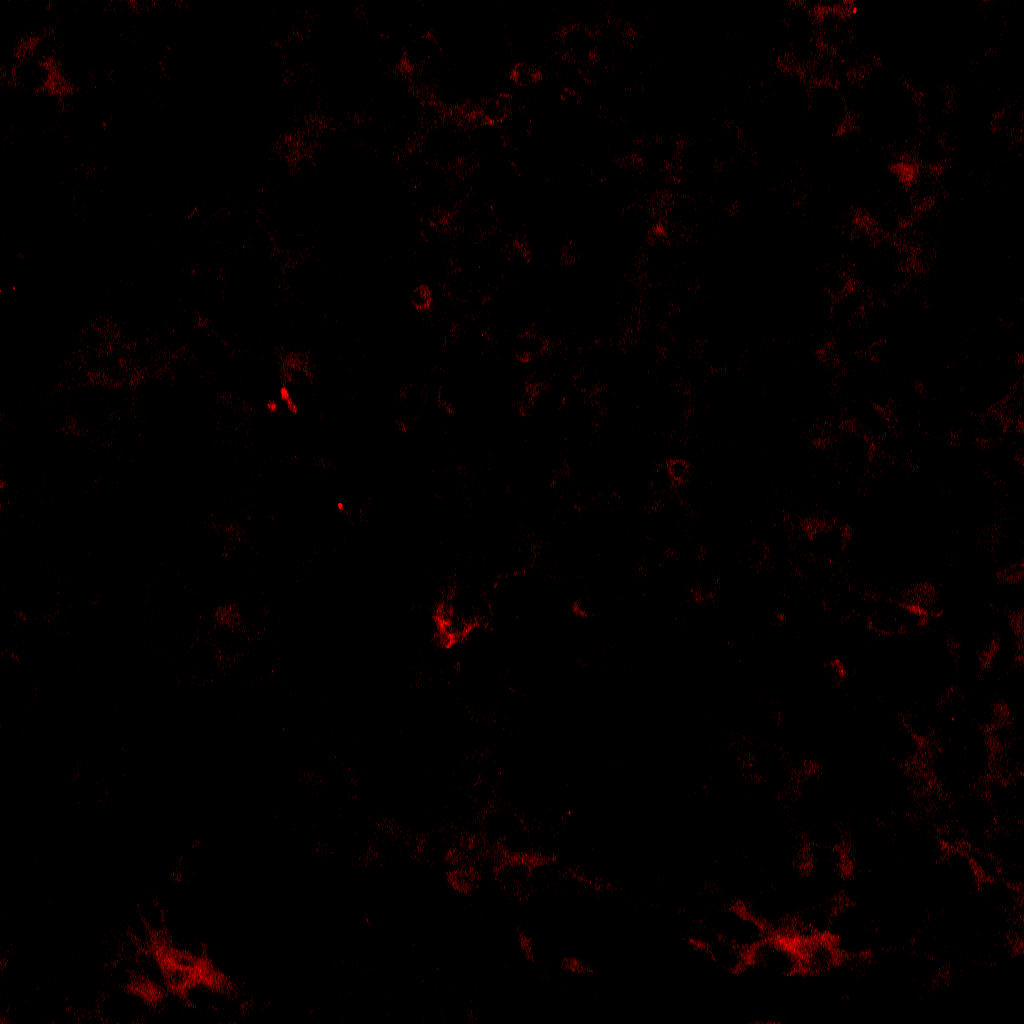

Supplement: Supplementary file 10 — Source data Fig. 6 [file 44318_2024_244_MOESM10_ESM.zip › Figure 6/6G/B16 WT cGAMP CD86.tif]

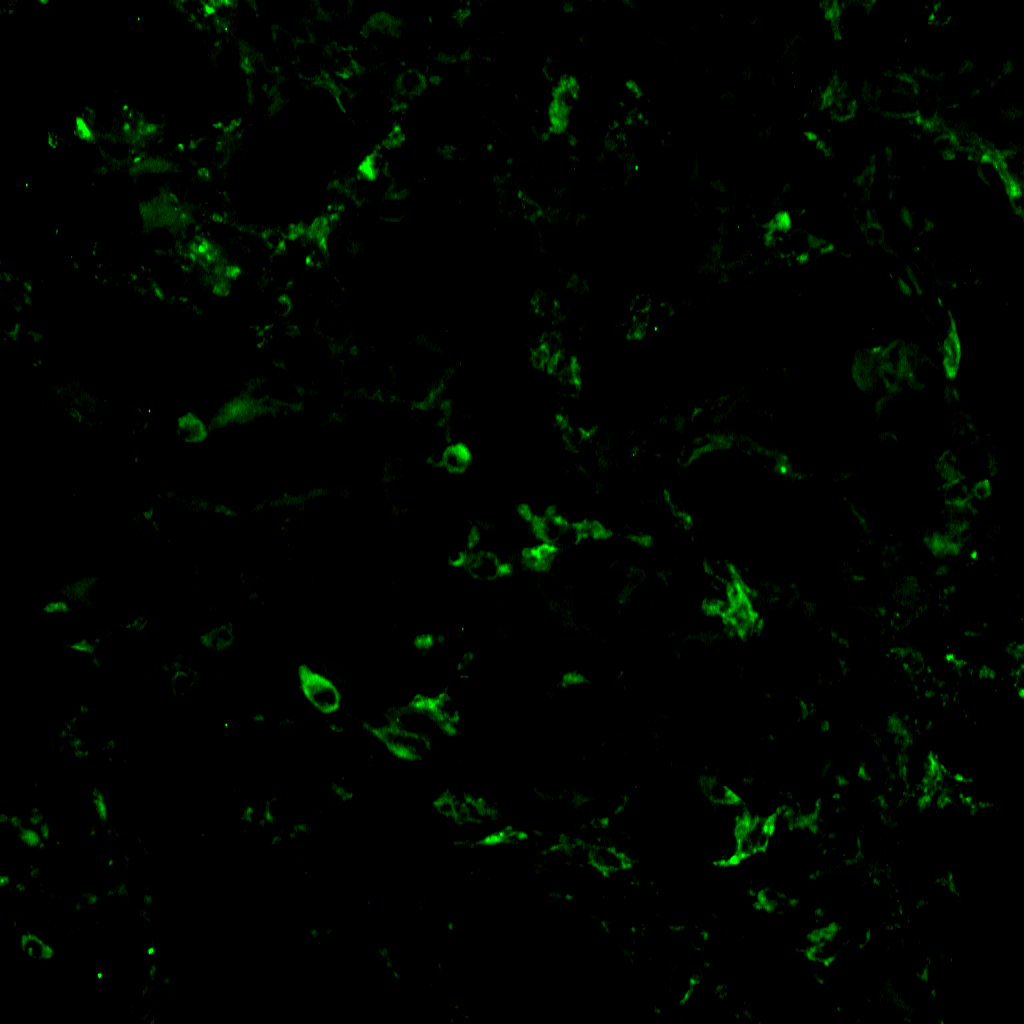

Supplement: Supplementary file 10 — Source data Fig. 6 [file 44318_2024_244_MOESM10_ESM.zip › Figure 6/6G/B16 WT cGAMP F4 80 with CD206.tif]

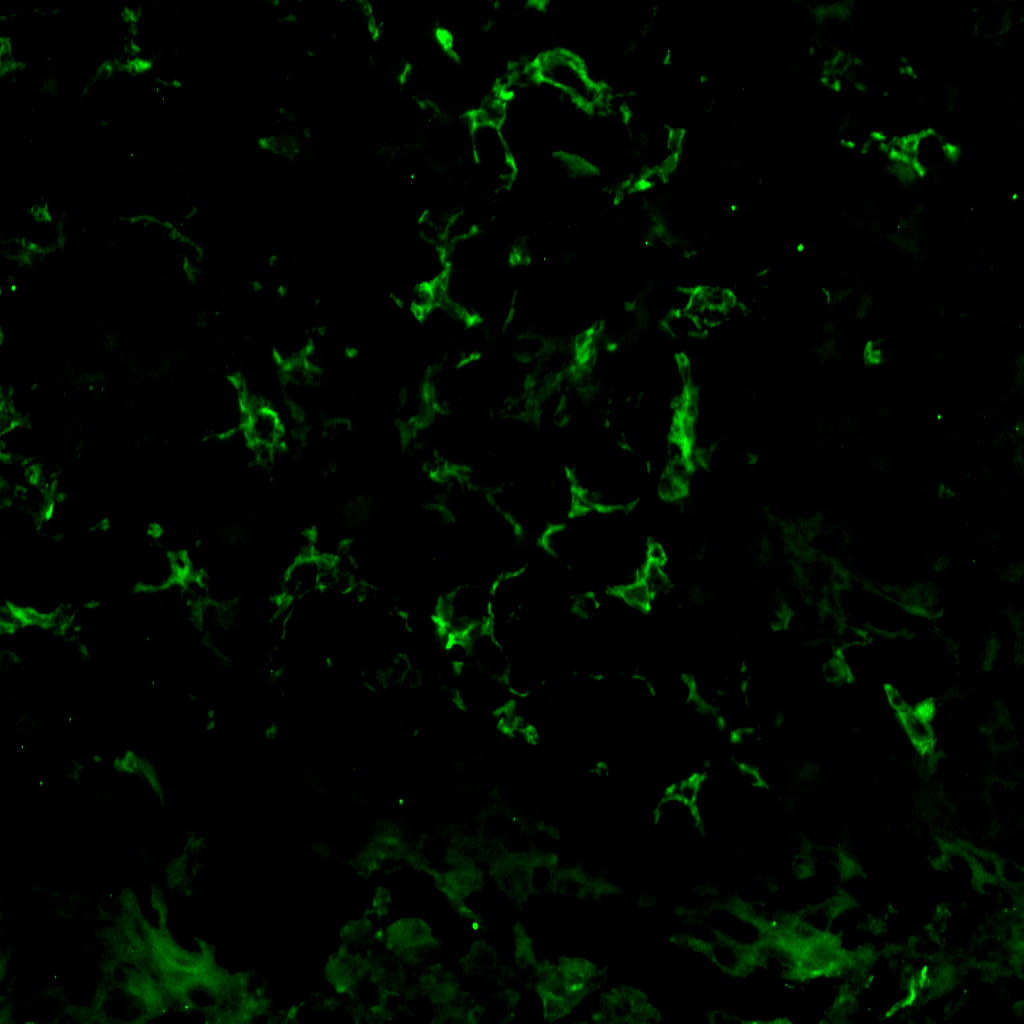

Supplement: Supplementary file 10 — Source data Fig. 6 [file 44318_2024_244_MOESM10_ESM.zip › Figure 6/6G/B16 WT cGAMP F4 80 with CD86.tif]

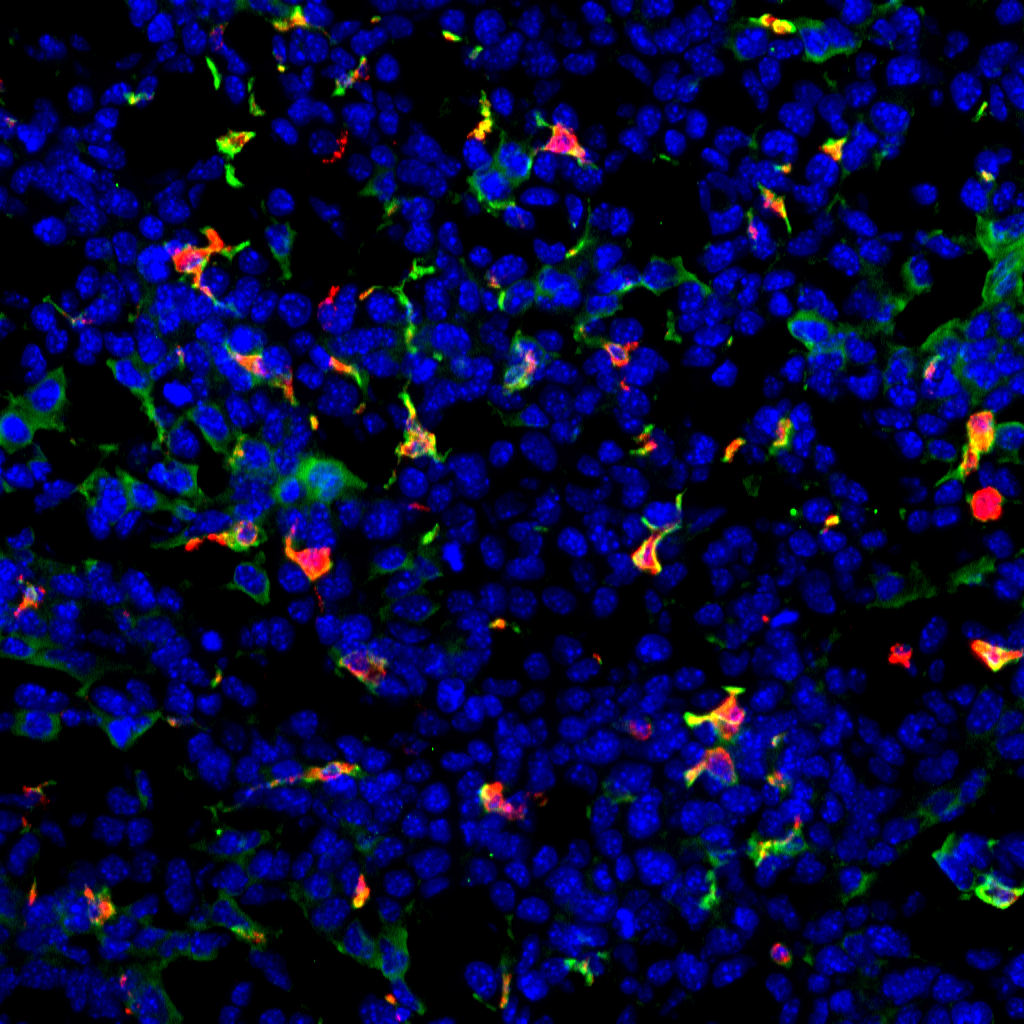

Supplement: Supplementary file 10 — Source data Fig. 6 [file 44318_2024_244_MOESM10_ESM.zip › Figure 6/6G/B16 WT Ctrl CD206 F4 80 Merge.tif]

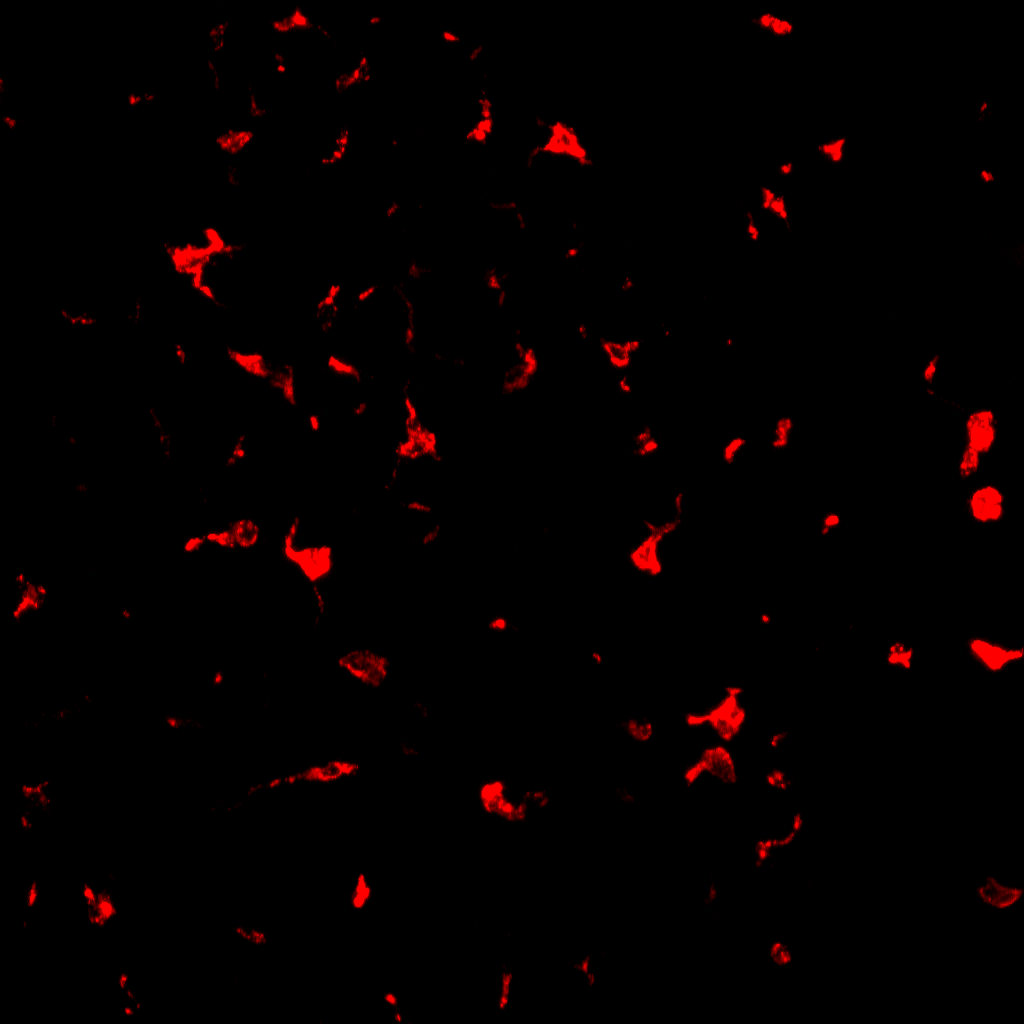

Supplement: Supplementary file 10 — Source data Fig. 6 [file 44318_2024_244_MOESM10_ESM.zip › Figure 6/6G/B16 WT Ctrl CD206.tif]

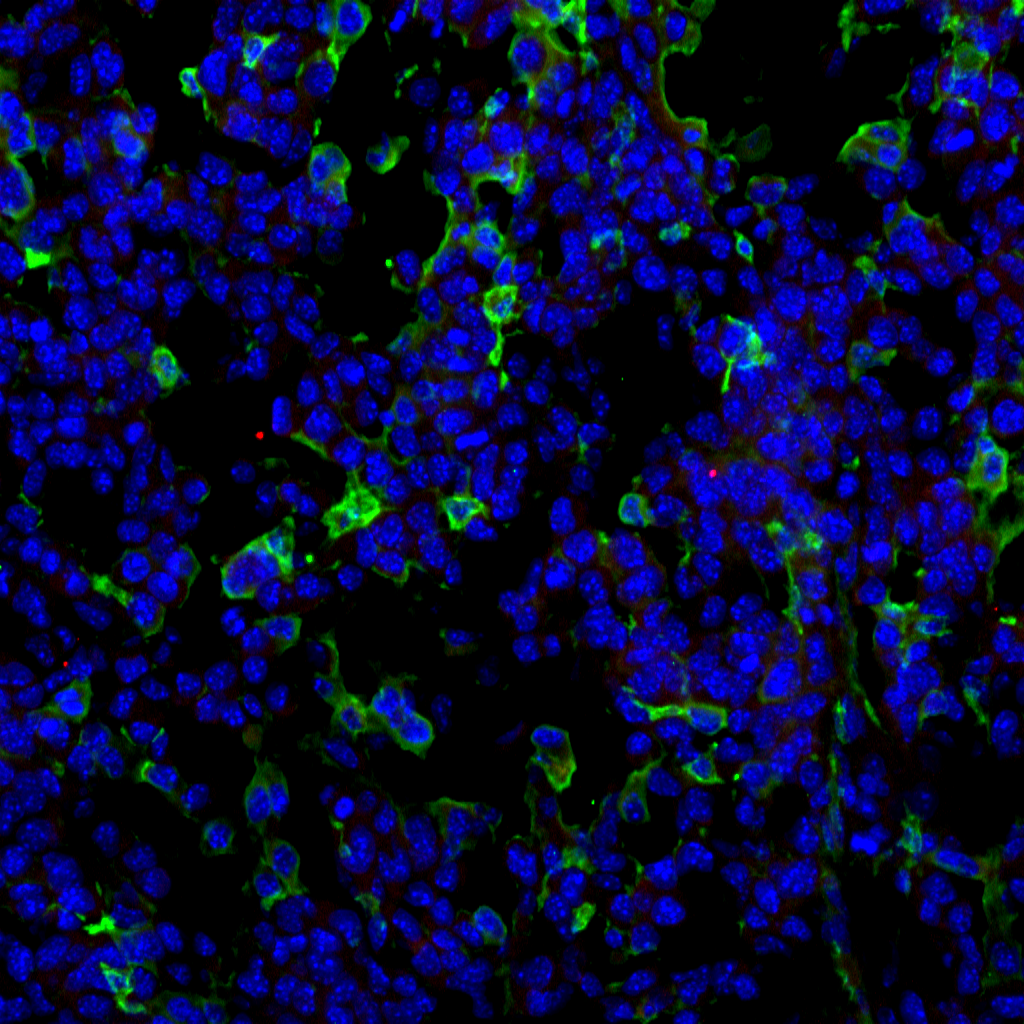

Supplement: Supplementary file 10 — Source data Fig. 6 [file 44318_2024_244_MOESM10_ESM.zip › Figure 6/6G/B16 WT Ctrl CD86 F4 80 Merge.tif]

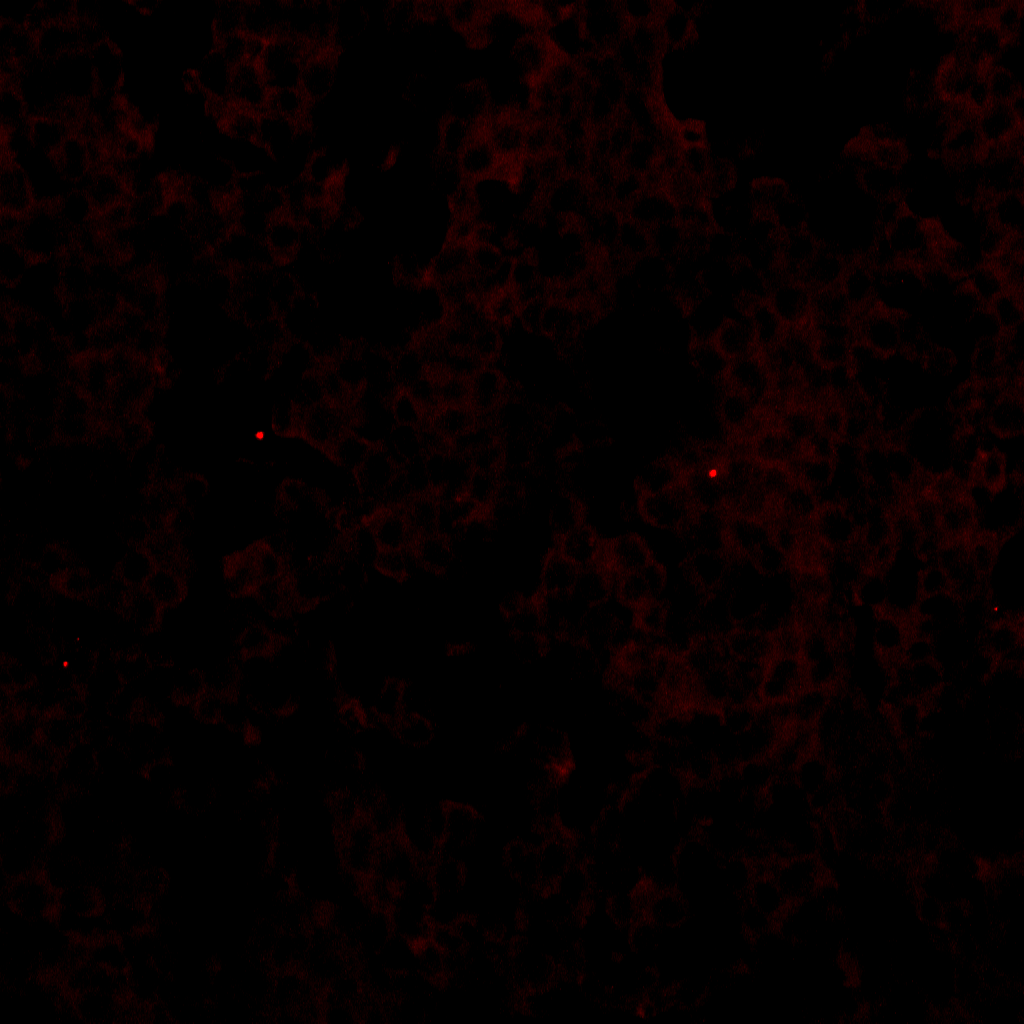

Supplement: Supplementary file 10 — Source data Fig. 6 [file 44318_2024_244_MOESM10_ESM.zip › Figure 6/6G/B16 WT Ctrl CD86.tif]

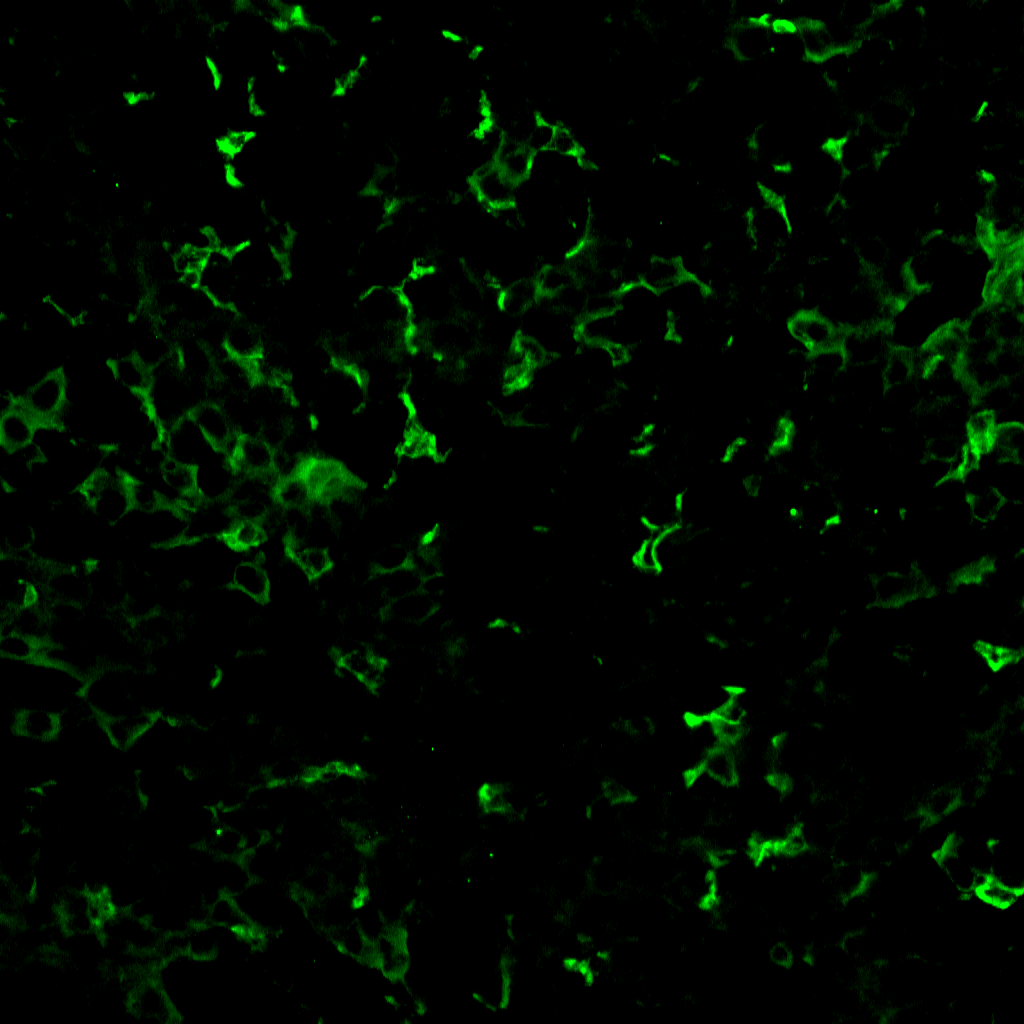

Supplement: Supplementary file 10 — Source data Fig. 6 [file 44318_2024_244_MOESM10_ESM.zip › Figure 6/6G/B16 WT Ctrl F4 80 with CD206.tif]

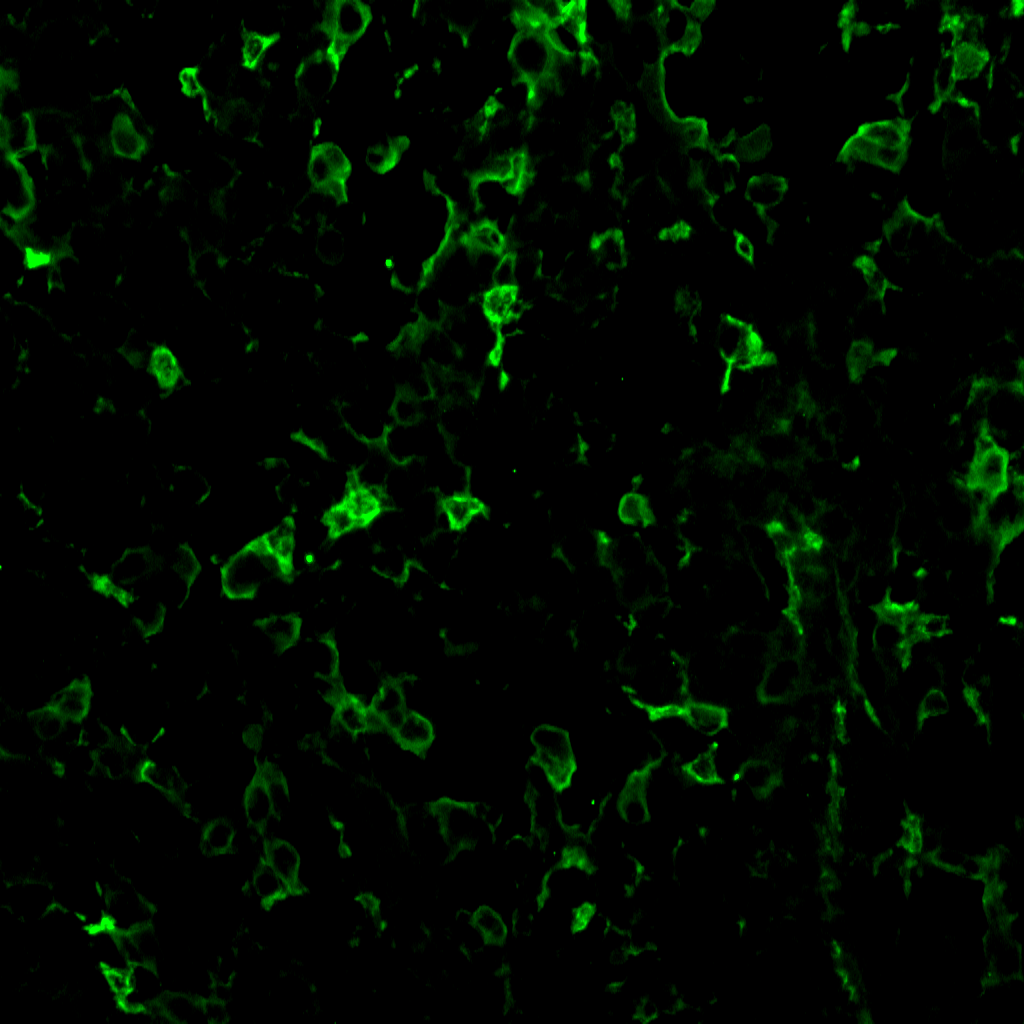

Supplement: Supplementary file 10 — Source data Fig. 6 [file 44318_2024_244_MOESM10_ESM.zip › Figure 6/6G/B16 WT Ctrl F4 80 with CD86.tif]

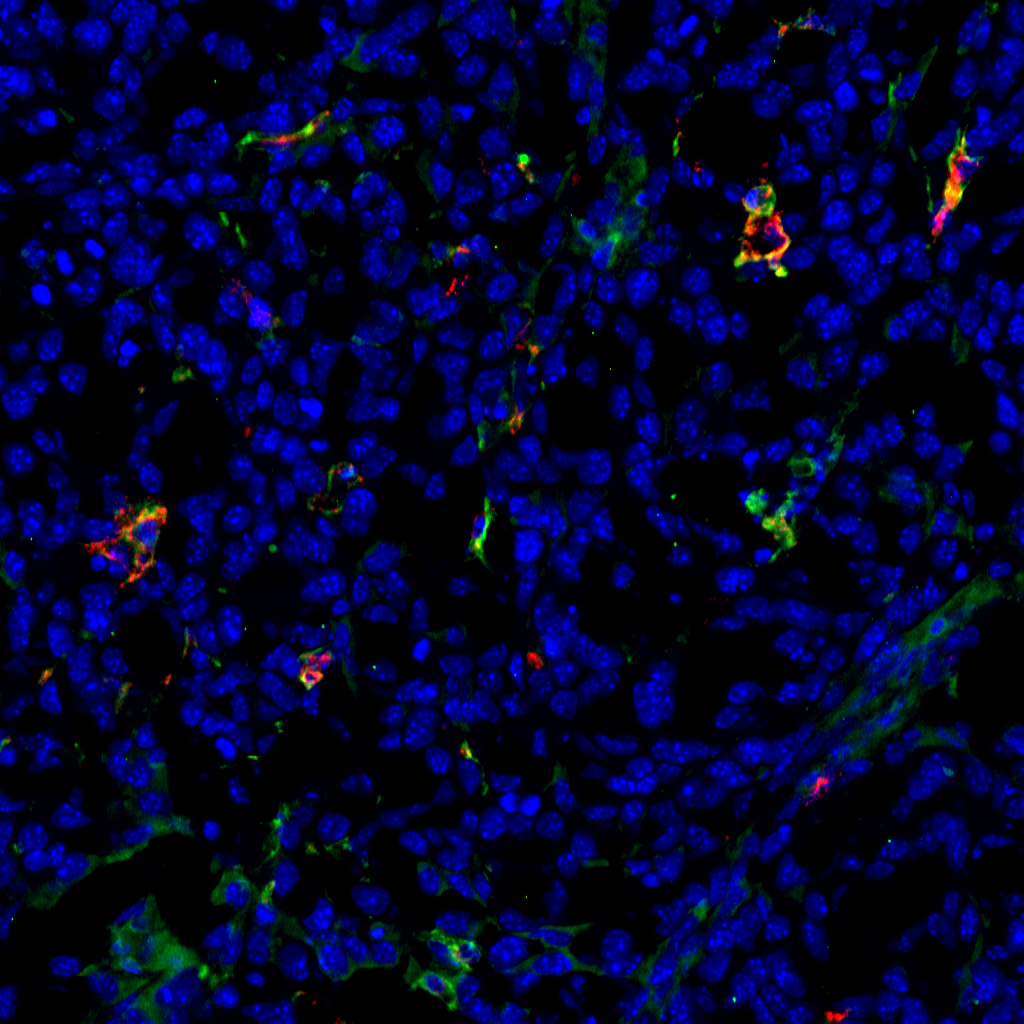

Supplement: Supplementary file 10 — Source data Fig. 6 [file 44318_2024_244_MOESM10_ESM.zip › Figure 6/6G/B16 Zyxin KO cGAMP CD206 F4 80 Merge.tif]

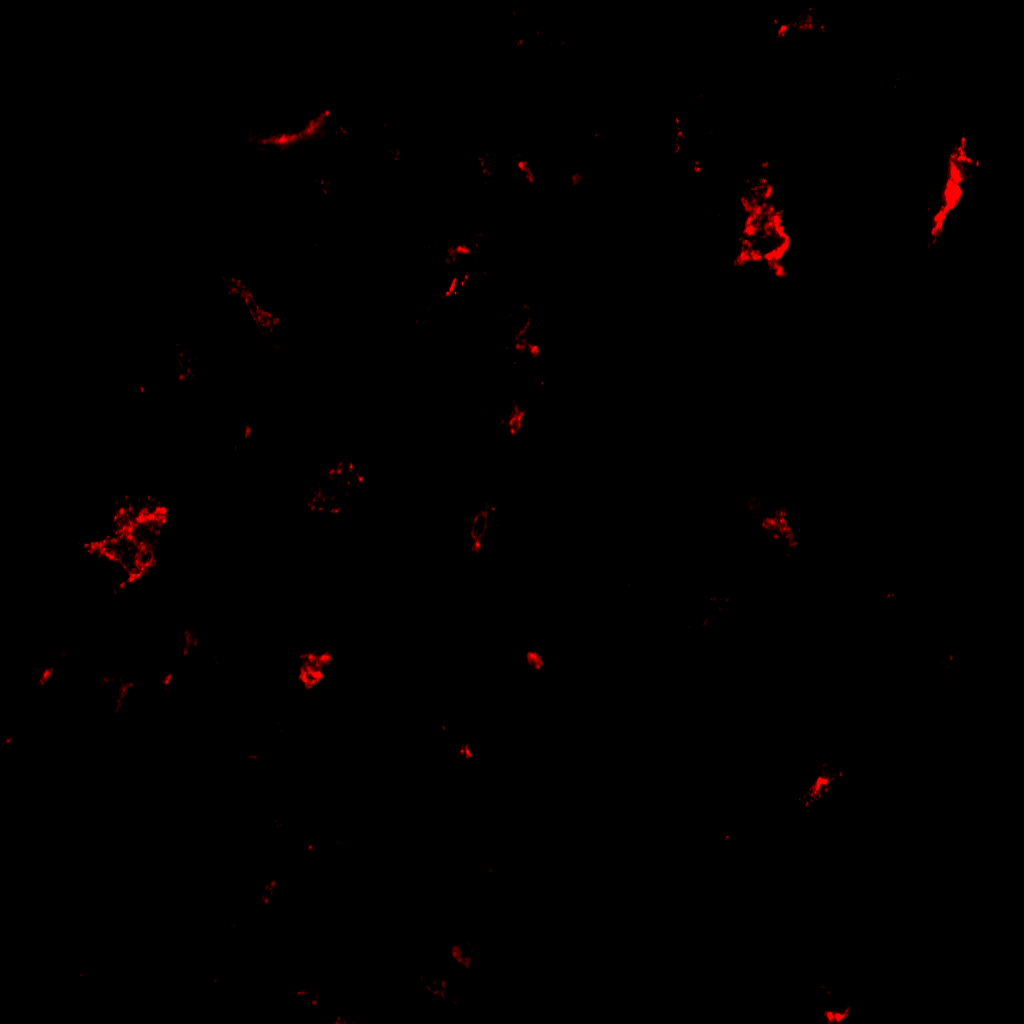

Supplement: Supplementary file 10 — Source data Fig. 6 [file 44318_2024_244_MOESM10_ESM.zip › Figure 6/6G/B16 Zyxin KO cGAMP CD206.tif]

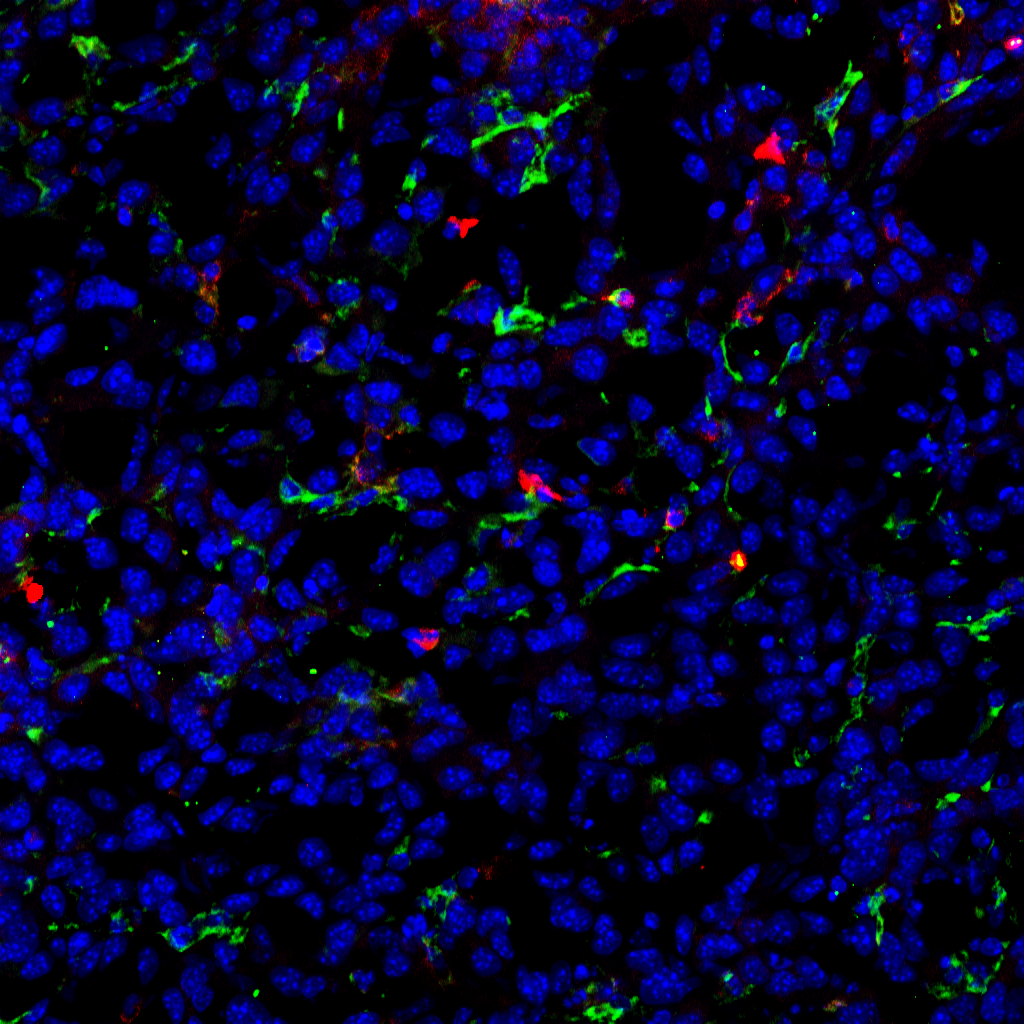

Supplement: Supplementary file 10 — Source data Fig. 6 [file 44318_2024_244_MOESM10_ESM.zip › Figure 6/6G/B16 Zyxin KO cGAMP CD86 F4 80 Merge.tif]

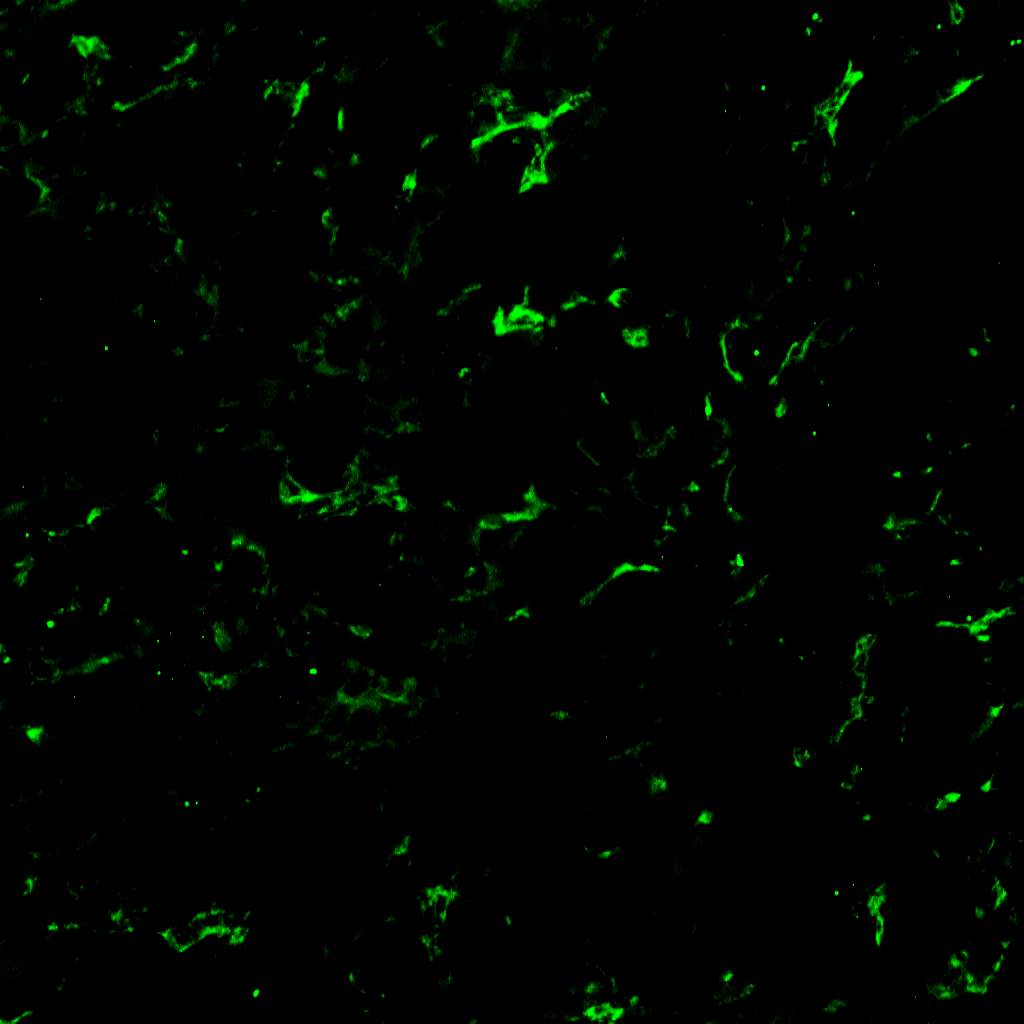

Supplement: Supplementary file 10 — Source data Fig. 6 [file 44318_2024_244_MOESM10_ESM.zip › Figure 6/6G/B16 Zyxin KO cGAMP CD86 with F4 80.tif]

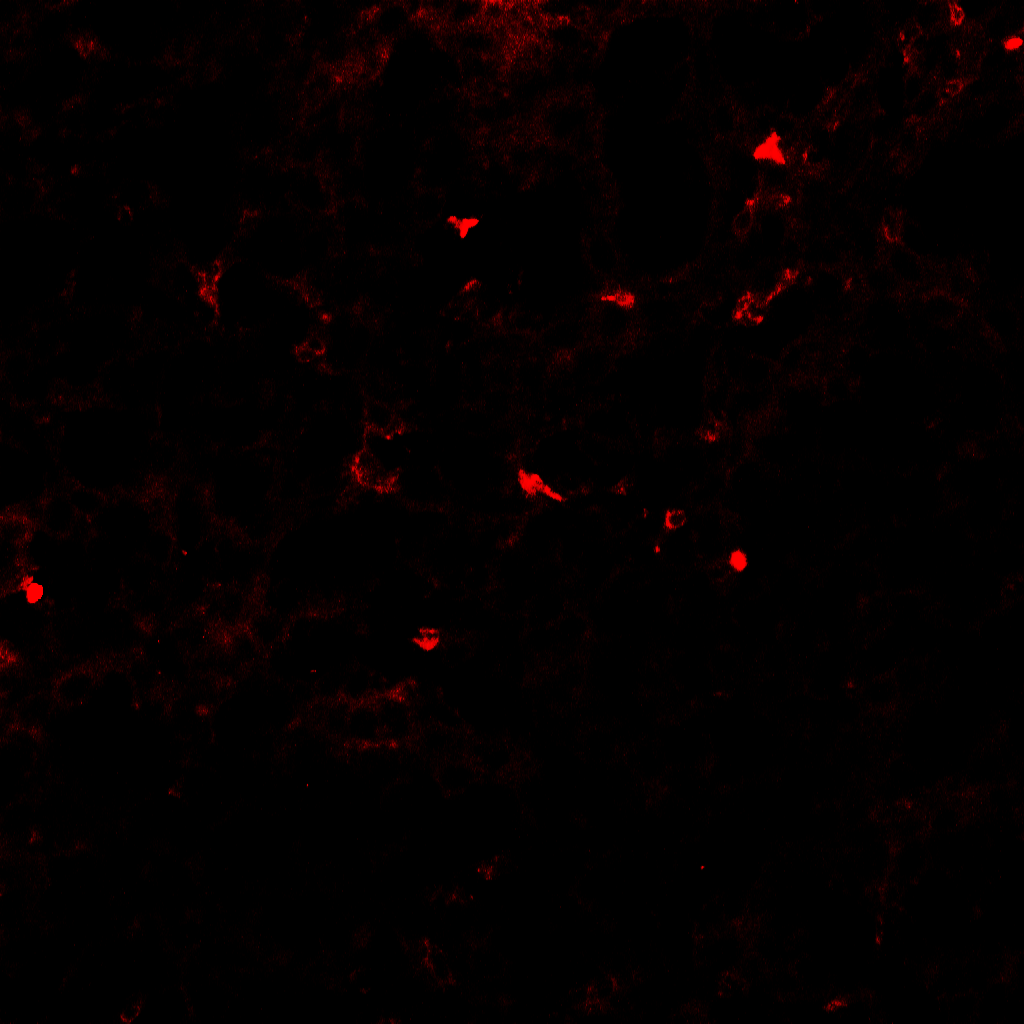

Supplement: Supplementary file 10 — Source data Fig. 6 [file 44318_2024_244_MOESM10_ESM.zip › Figure 6/6G/B16 Zyxin KO cGAMP CD86.tif]

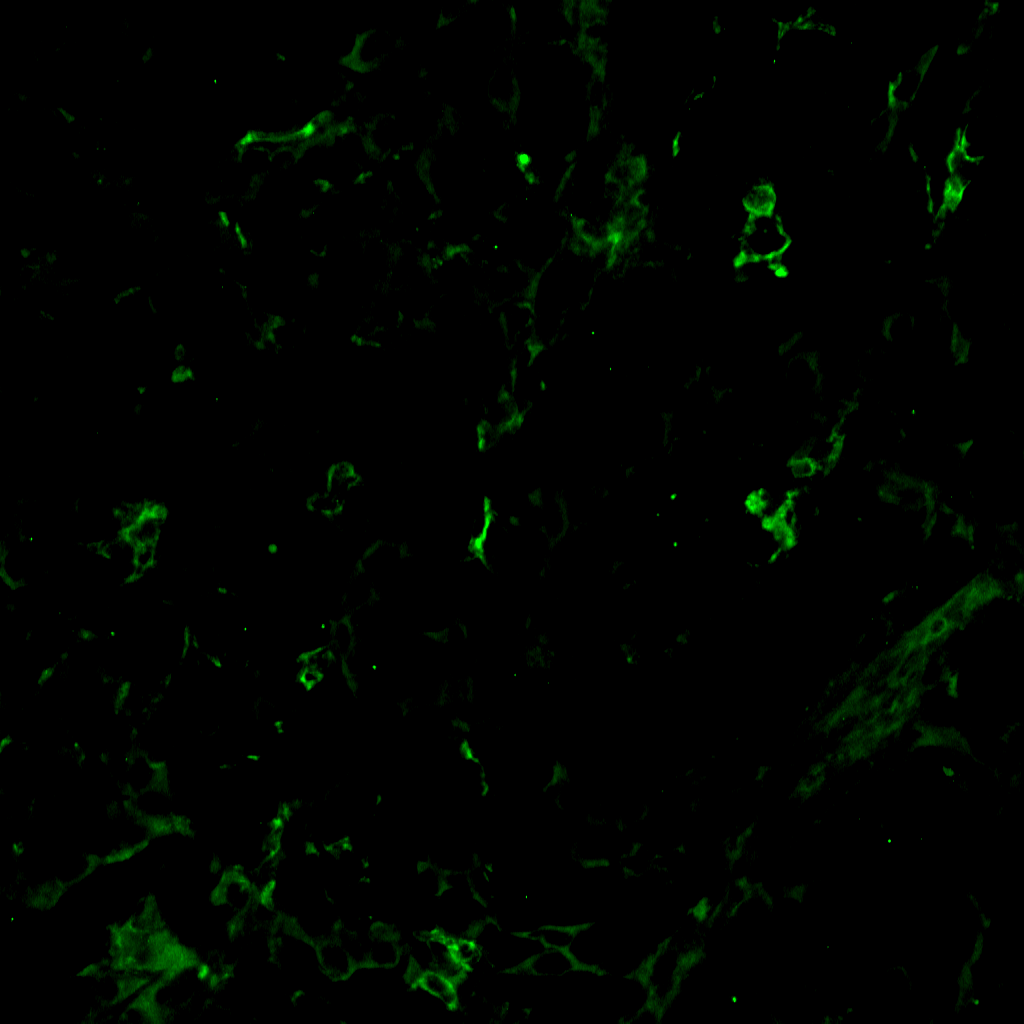

Supplement: Supplementary file 10 — Source data Fig. 6 [file 44318_2024_244_MOESM10_ESM.zip › Figure 6/6G/B16 Zyxin KO cGAMP F4 80 eith CD206.tif]

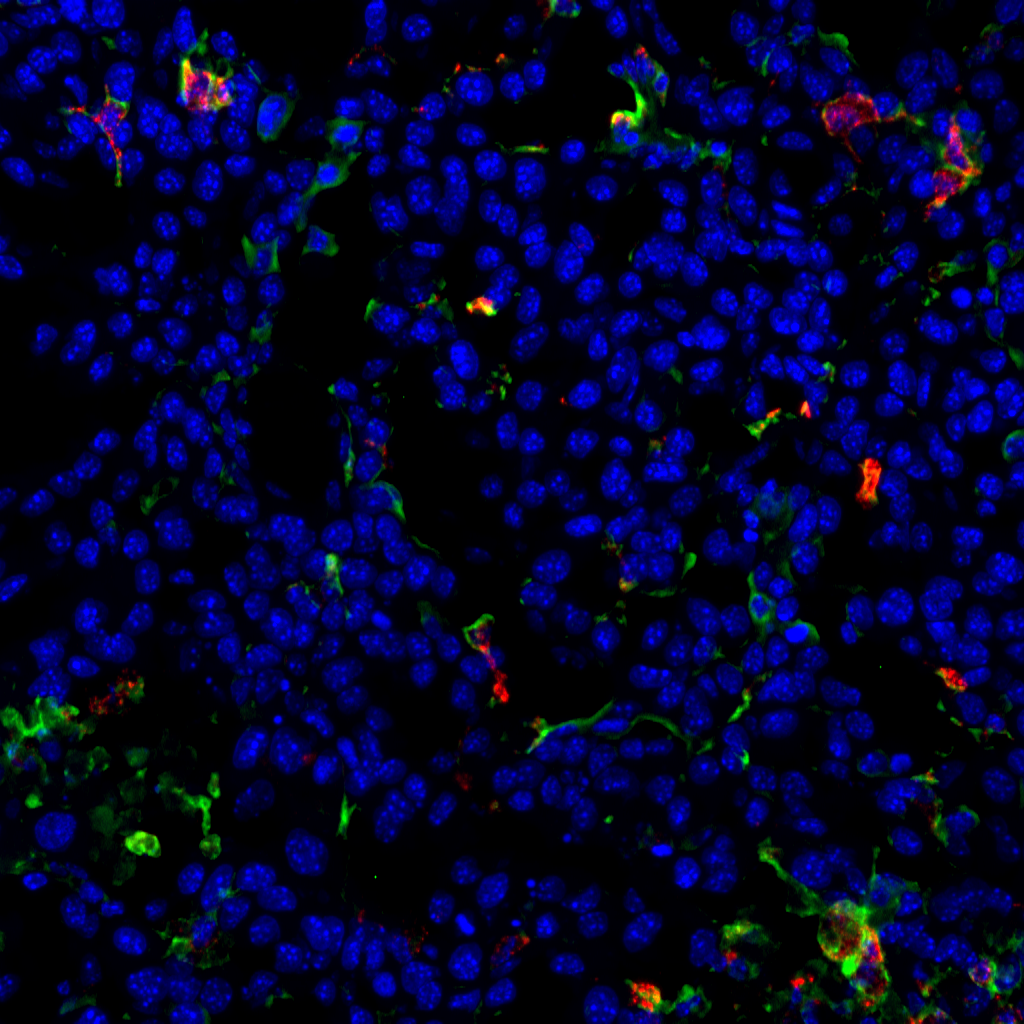

Supplement: Supplementary file 10 — Source data Fig. 6 [file 44318_2024_244_MOESM10_ESM.zip › Figure 6/6G/B16 Zyxin KO Ctrl CD206 F4 80 Merge.tif]

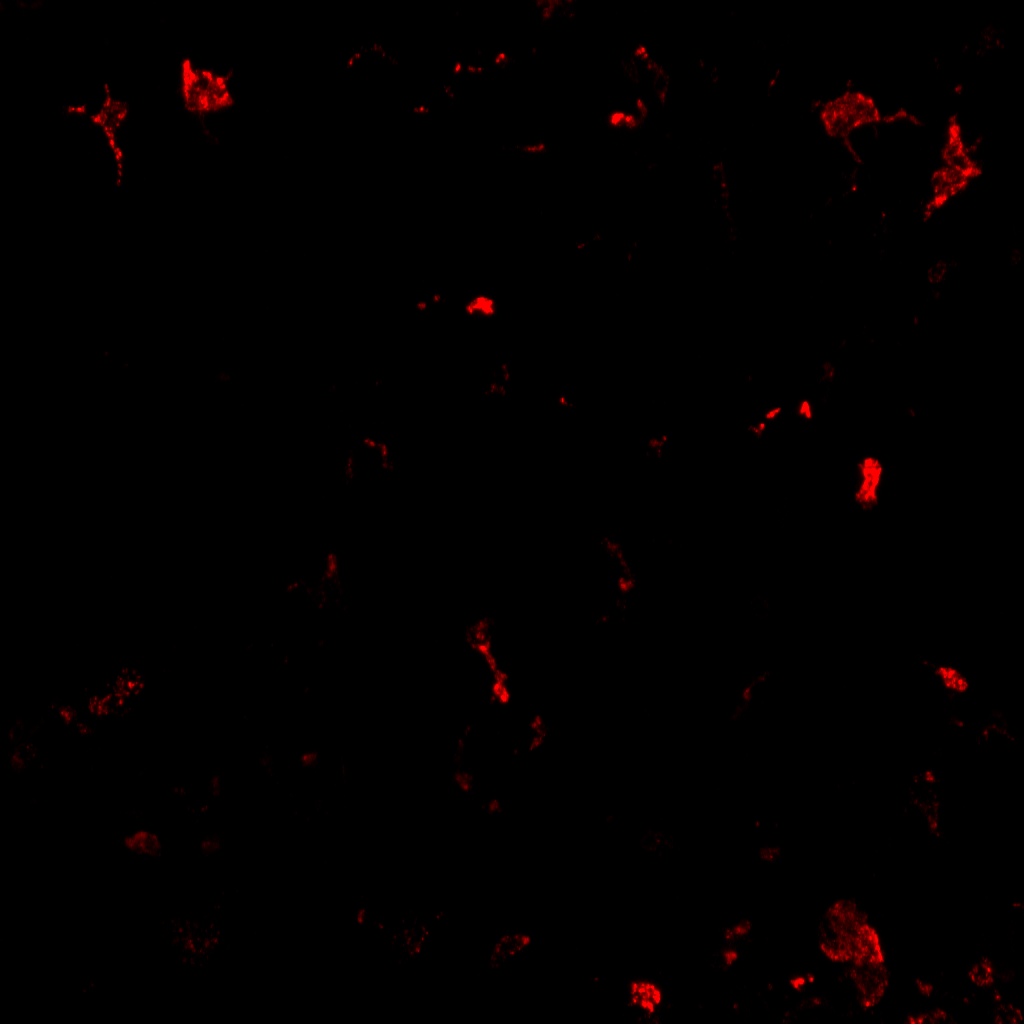

Supplement: Supplementary file 10 — Source data Fig. 6 [file 44318_2024_244_MOESM10_ESM.zip › Figure 6/6G/B16 Zyxin KO Ctrl CD206.tif]

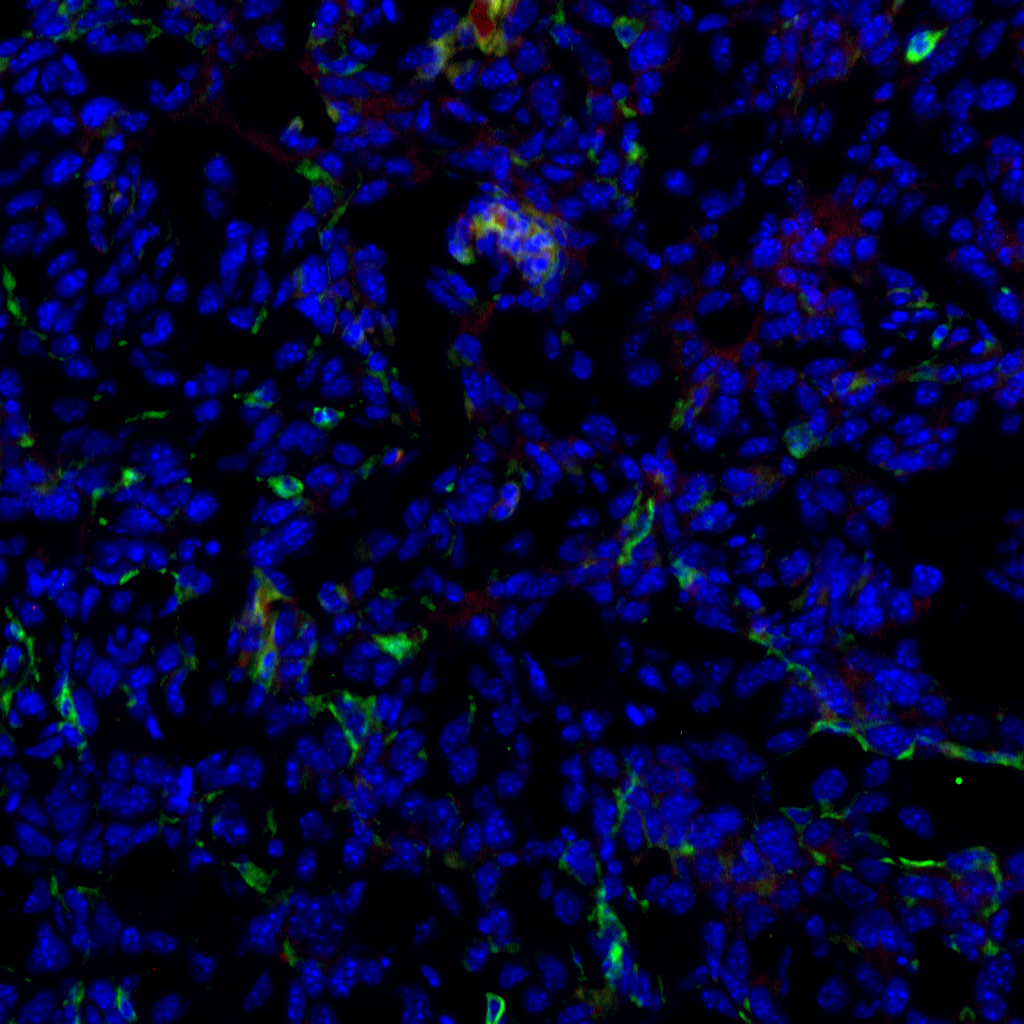

Supplement: Supplementary file 10 — Source data Fig. 6 [file 44318_2024_244_MOESM10_ESM.zip › Figure 6/6G/B16 Zyxin KO Ctrl CD86 F4 80 Merge.tif]

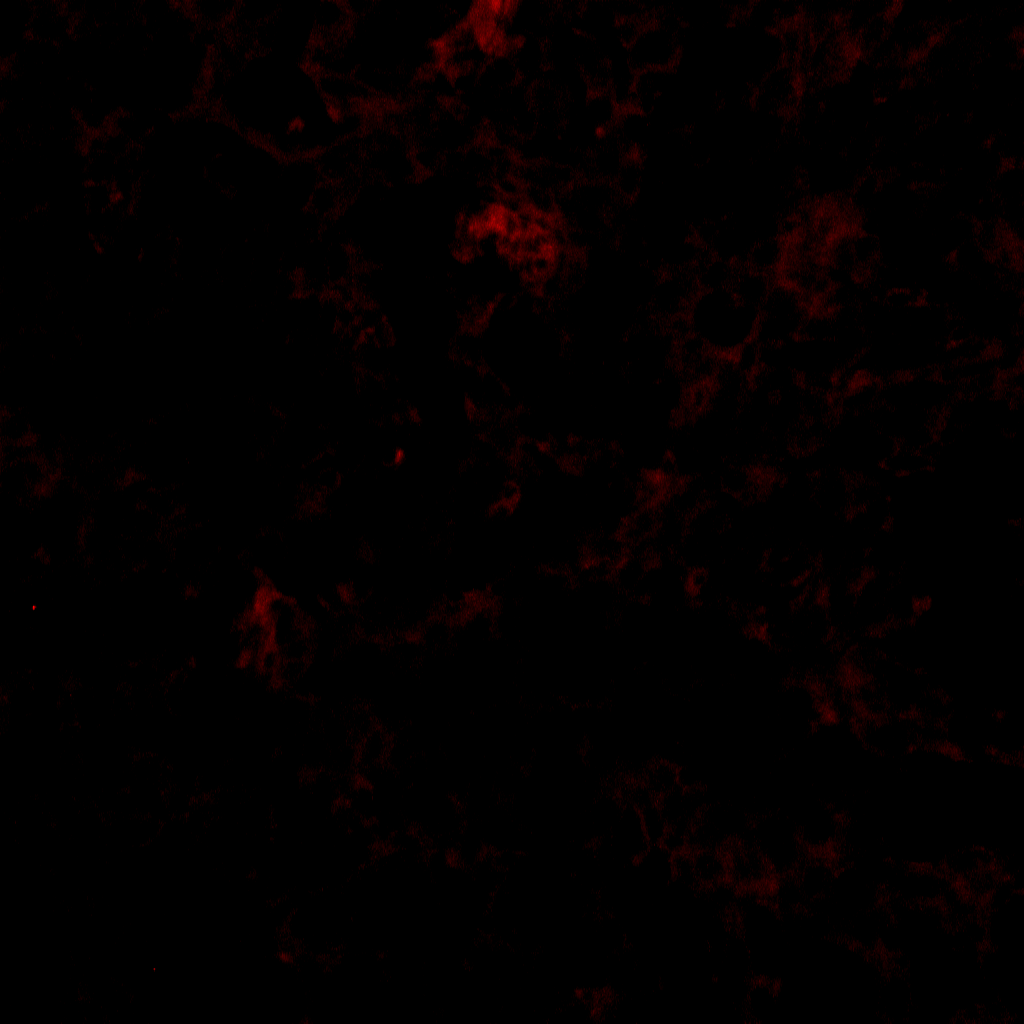

Supplement: Supplementary file 10 — Source data Fig. 6 [file 44318_2024_244_MOESM10_ESM.zip › Figure 6/6G/B16 Zyxin KO Ctrl CD86.tif]

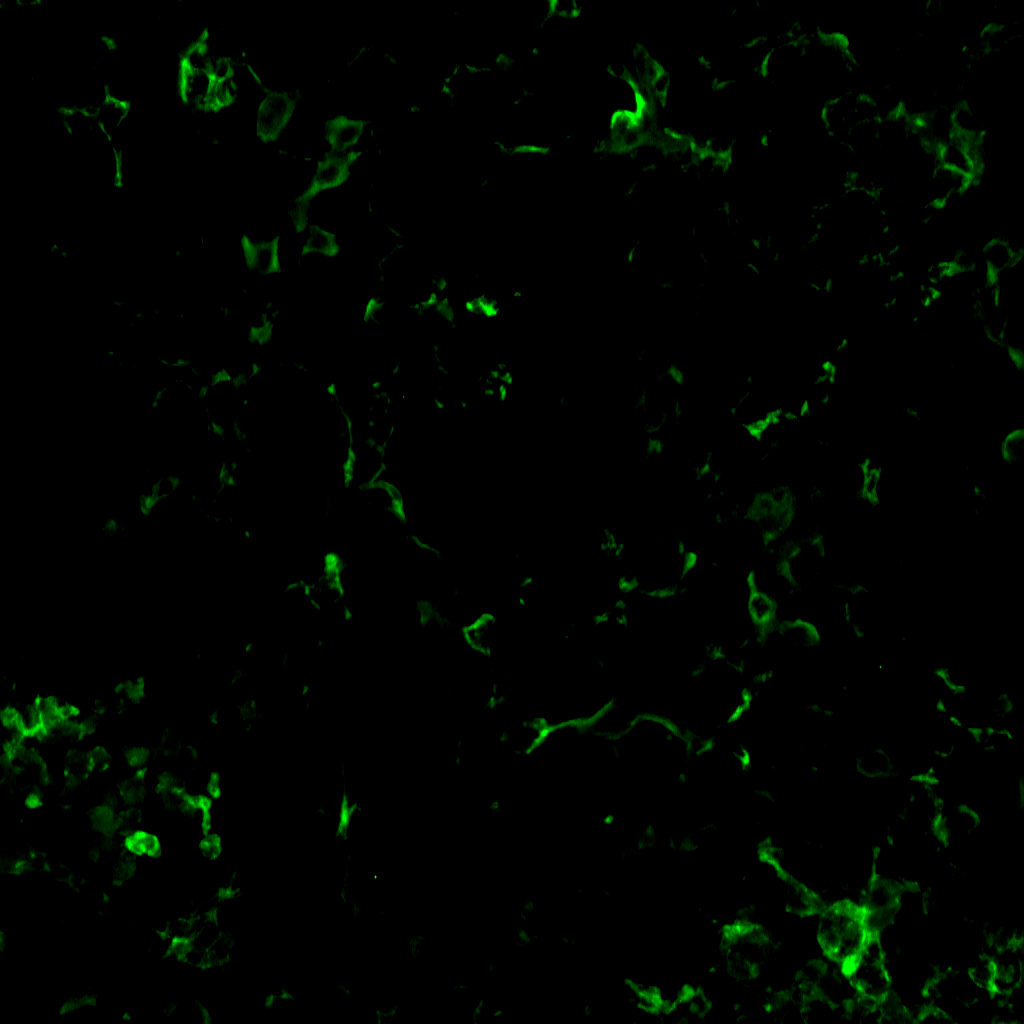

Supplement: Supplementary file 10 — Source data Fig. 6 [file 44318_2024_244_MOESM10_ESM.zip › Figure 6/6G/B16 Zyxin KO Ctrl F4 80 with CD206.tif]

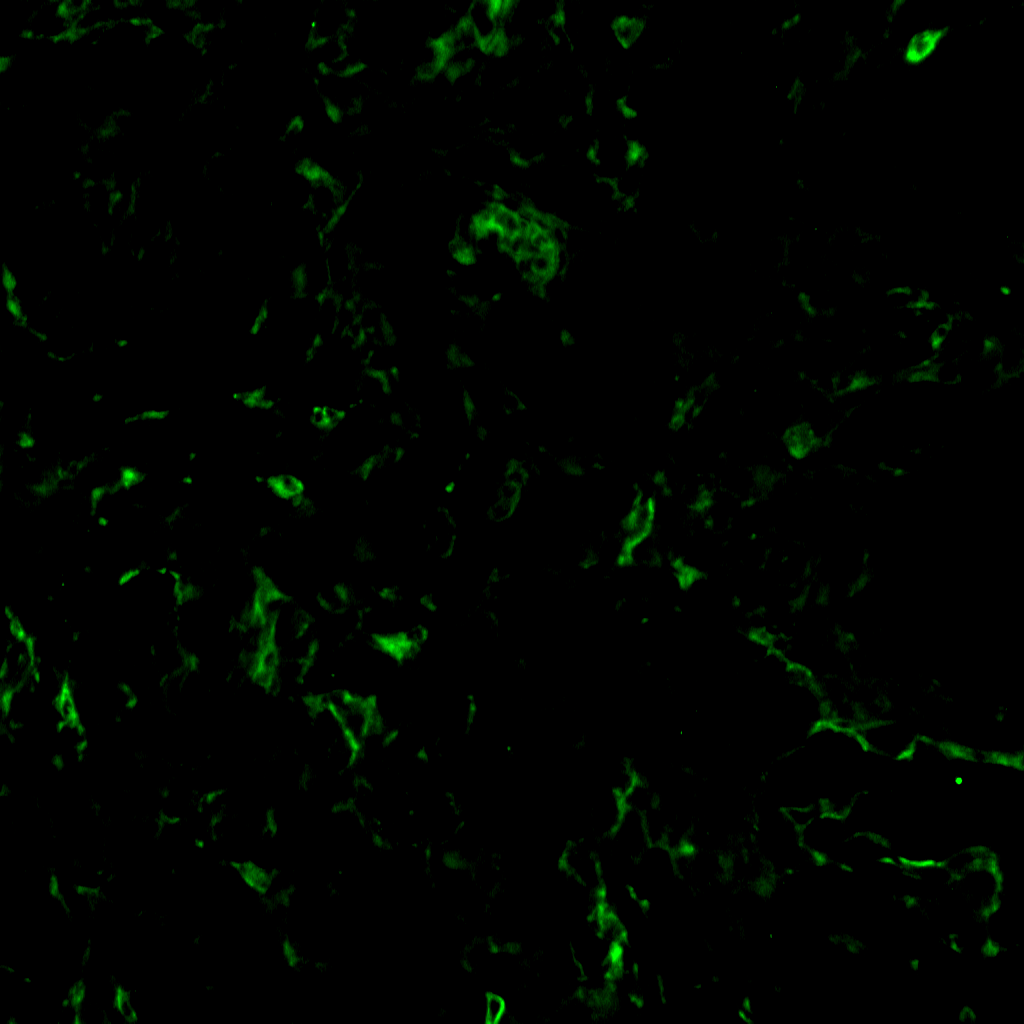

Supplement: Supplementary file 10 — Source data Fig. 6 [file 44318_2024_244_MOESM10_ESM.zip › Figure 6/6G/B16 Zyxin KO Ctrl F4 80 with CD86.tif]

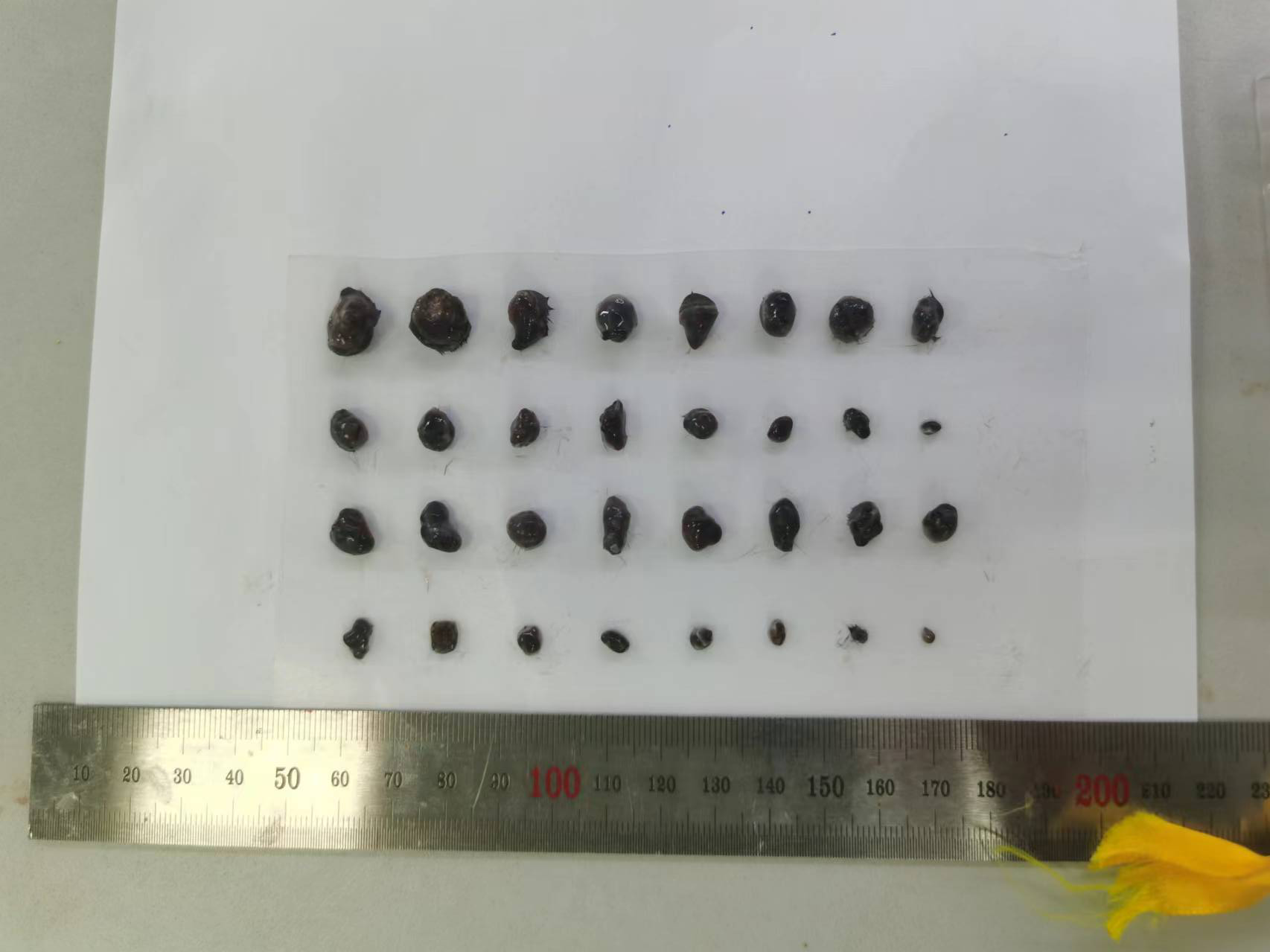

Supplement: Supplementary file 10 — Source data Fig. 6 [file 44318_2024_244_MOESM10_ESM.zip › Figure 6/6I/6I B16-F10 Inoculation.tif]

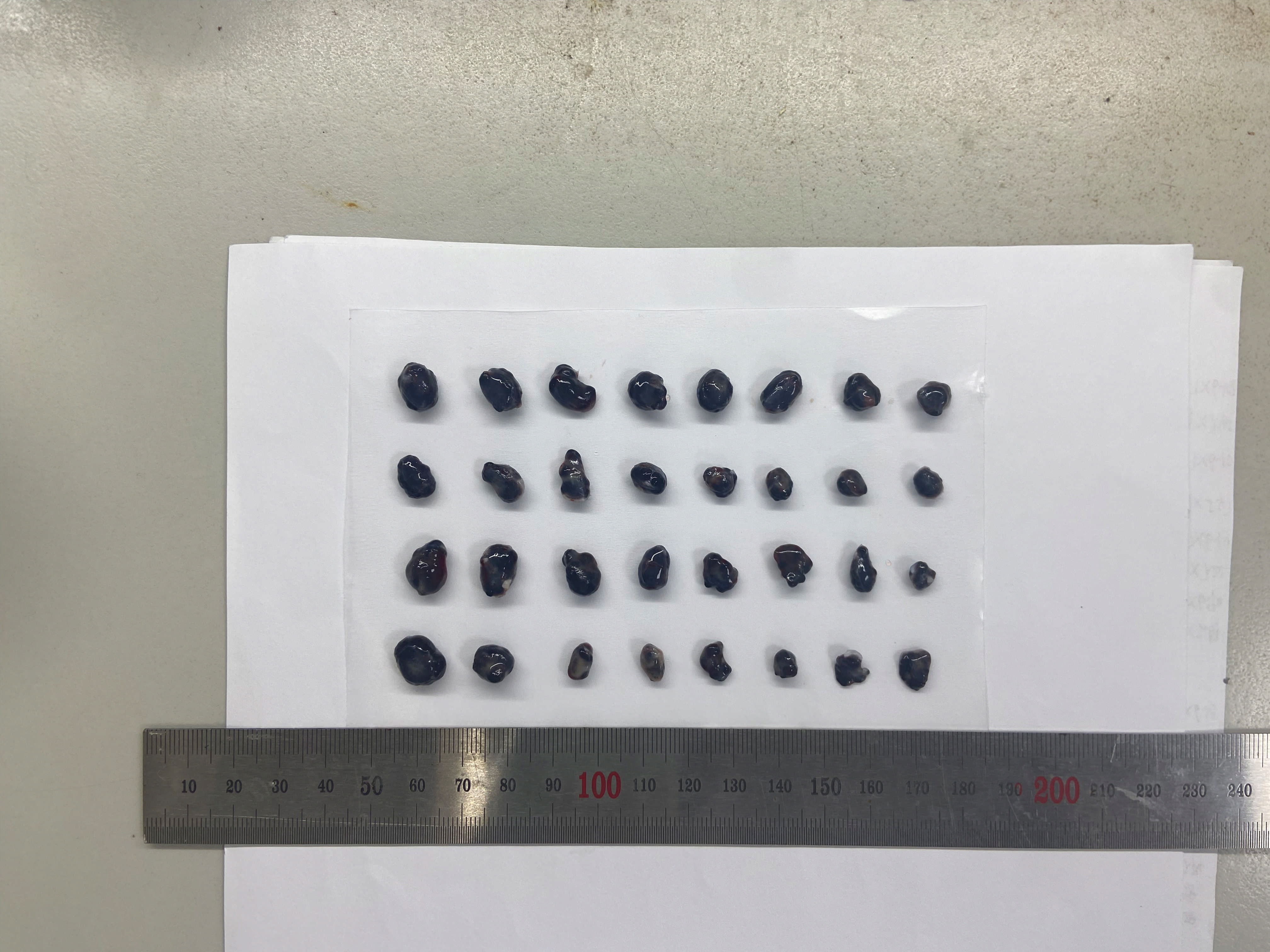

Supplement: Supplementary file 11 — Source data Fig. 7 [file 44318_2024_244_MOESM11_ESM.zip › Figure 7/7D/7D NSG-B16-F10-tumor.tiff]

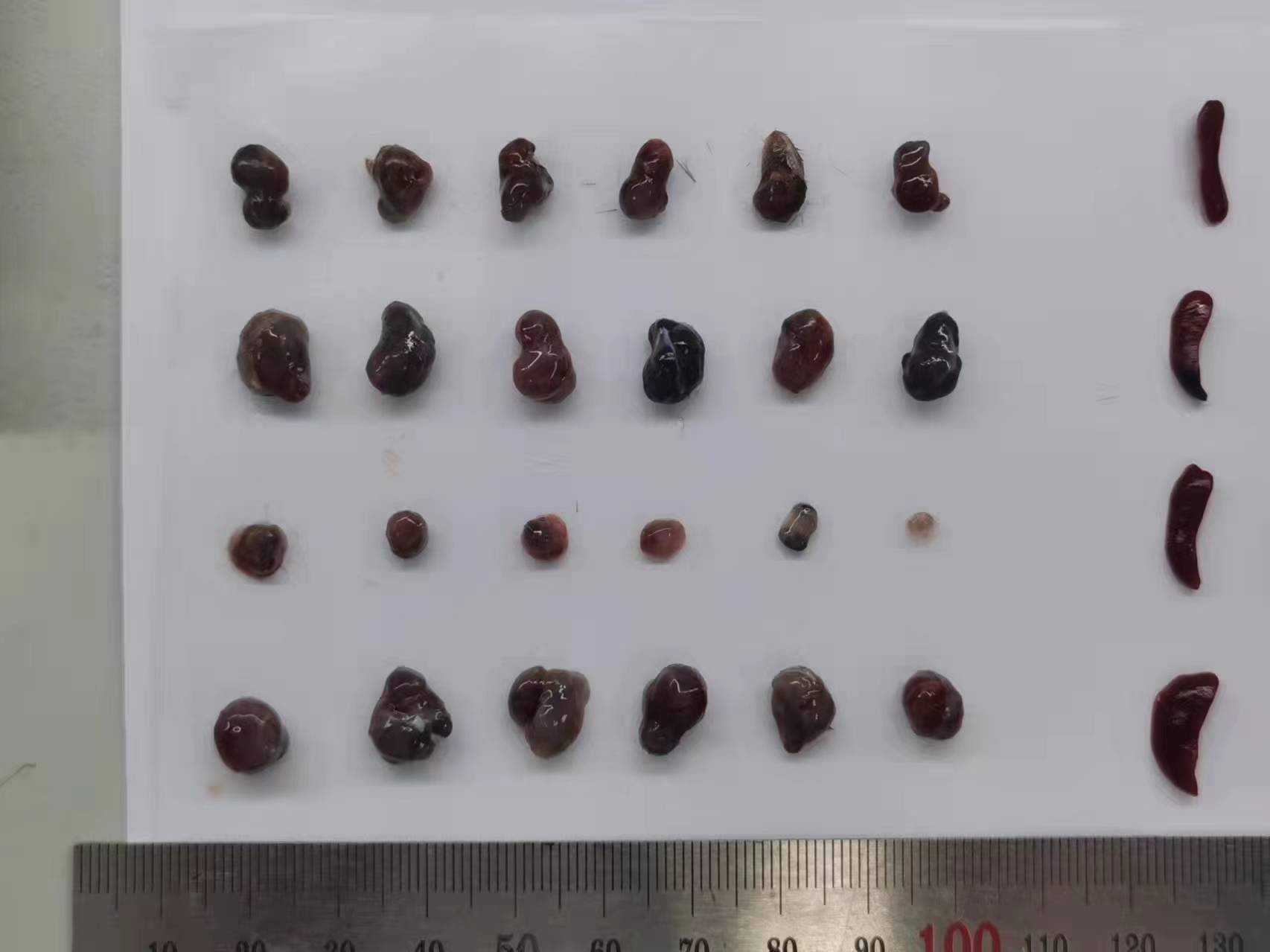

Supplement: Supplementary file 11 — Source data Fig. 7 [file 44318_2024_244_MOESM11_ESM.zip › Figure 7/7G/7G B16-F10 Inoculation.tif]

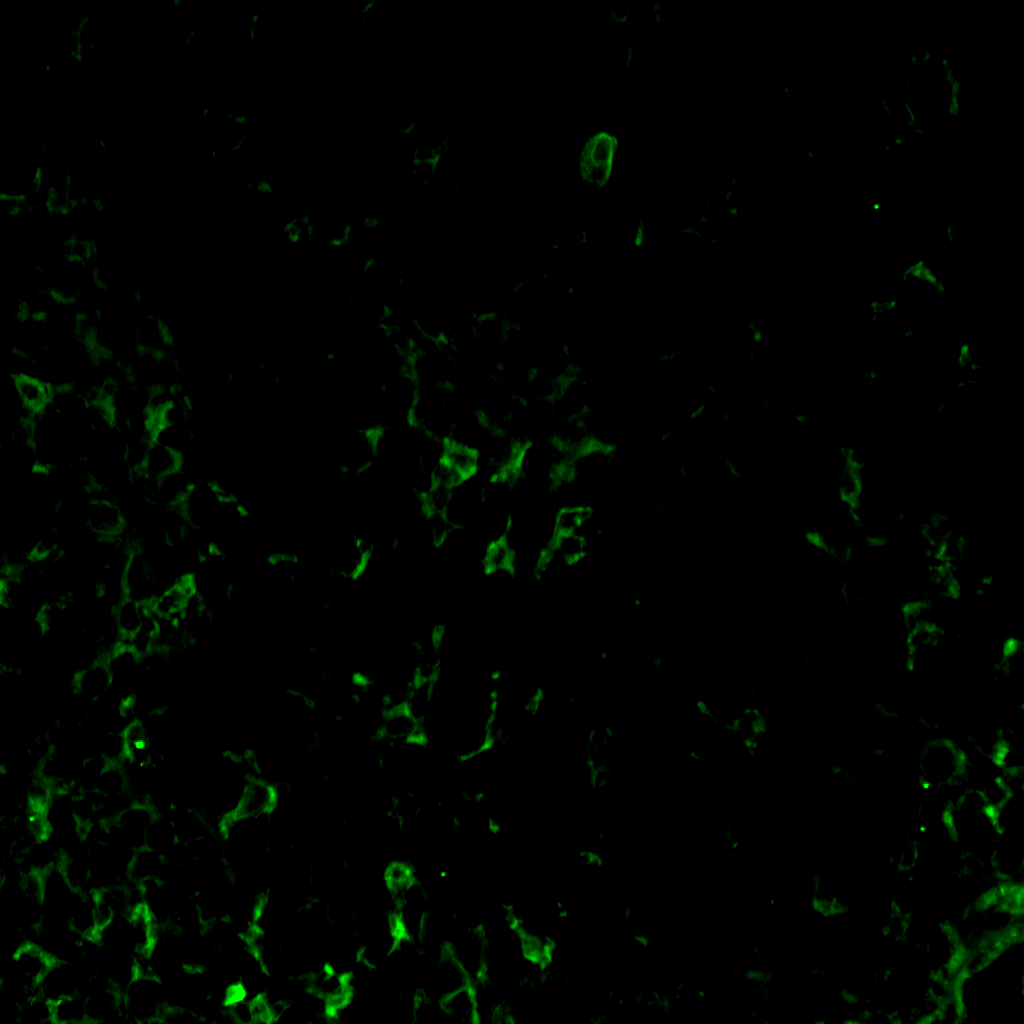

Supplement: Supplementary file 11 — Source data Fig. 7 [file 44318_2024_244_MOESM11_ESM.zip › Figure 7/7K/GSK8612 F4 80.tif]

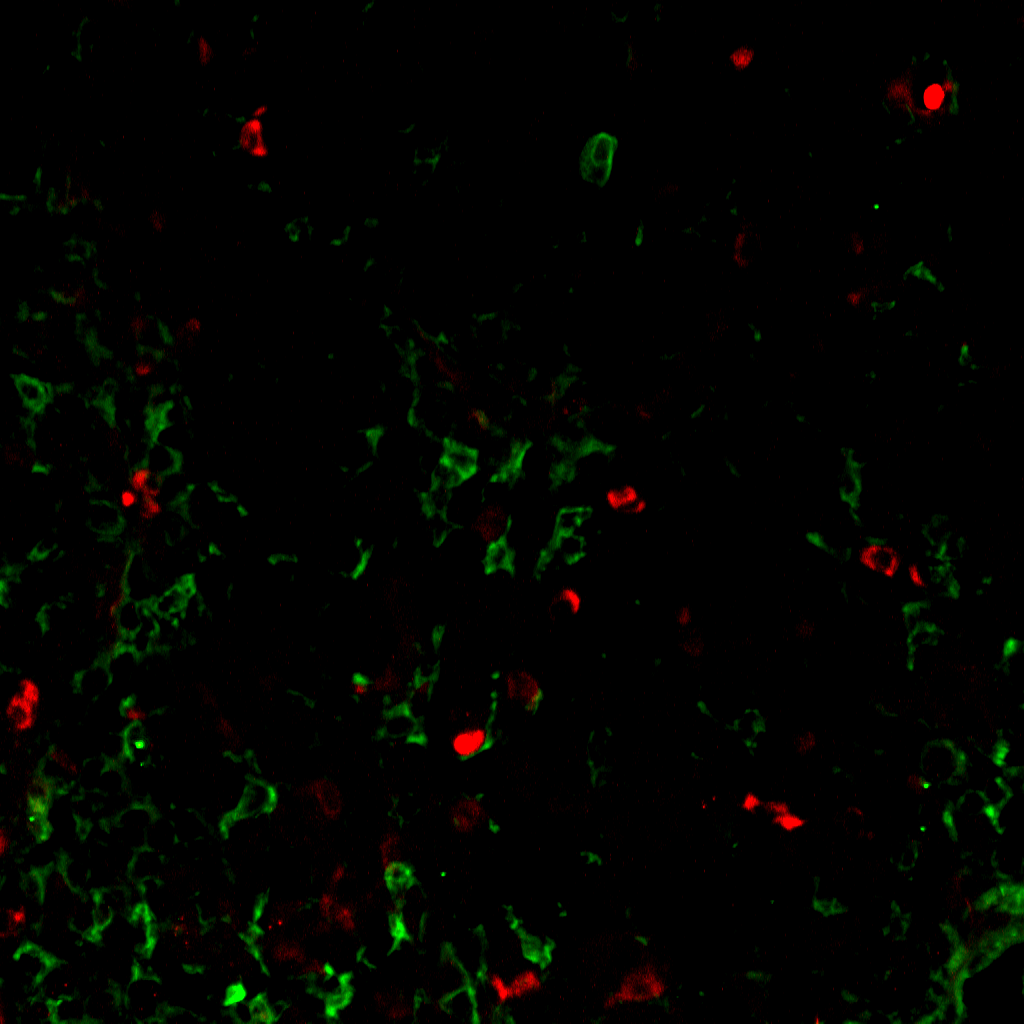

Supplement: Supplementary file 11 — Source data Fig. 7 [file 44318_2024_244_MOESM11_ESM.zip › Figure 7/7K/GSK8612 Merge.tif]

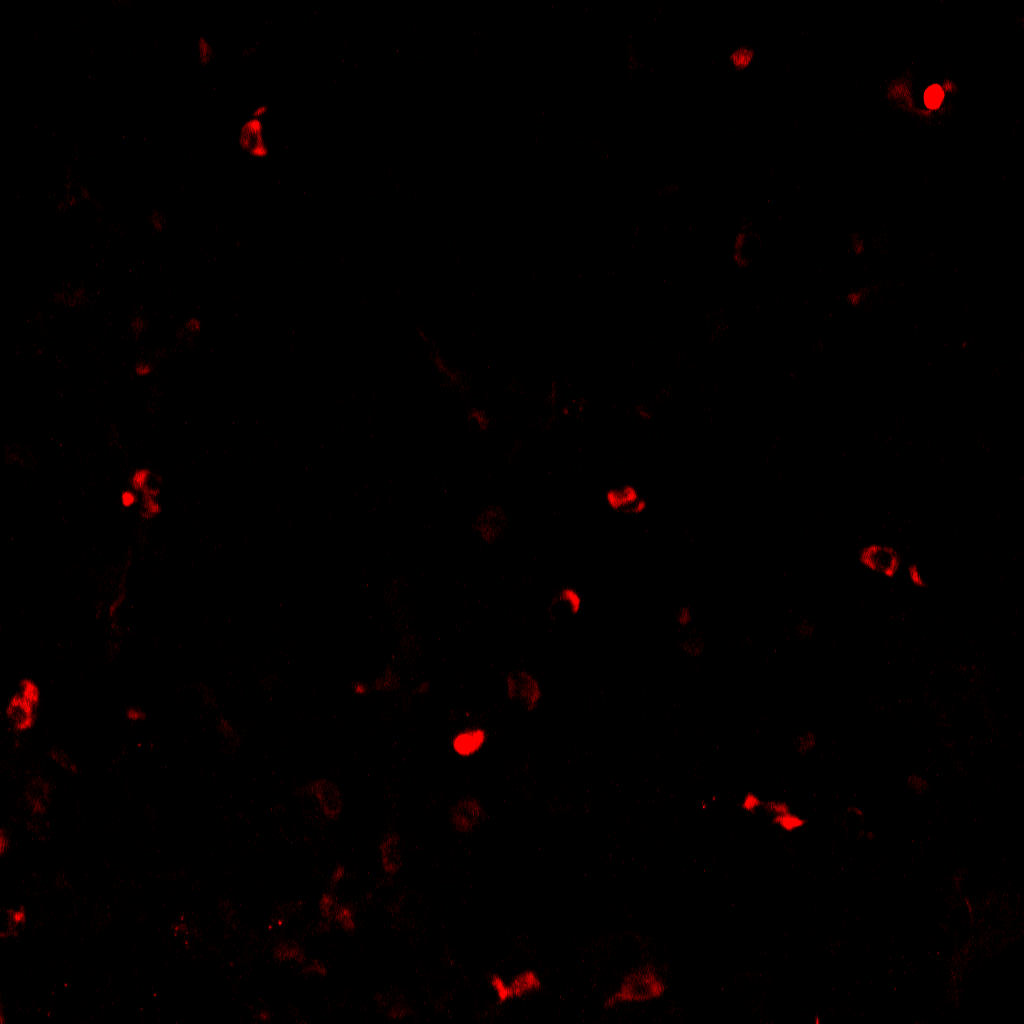

Supplement: Supplementary file 11 — Source data Fig. 7 [file 44318_2024_244_MOESM11_ESM.zip › Figure 7/7K/GSK8612 pZyxin.tif]

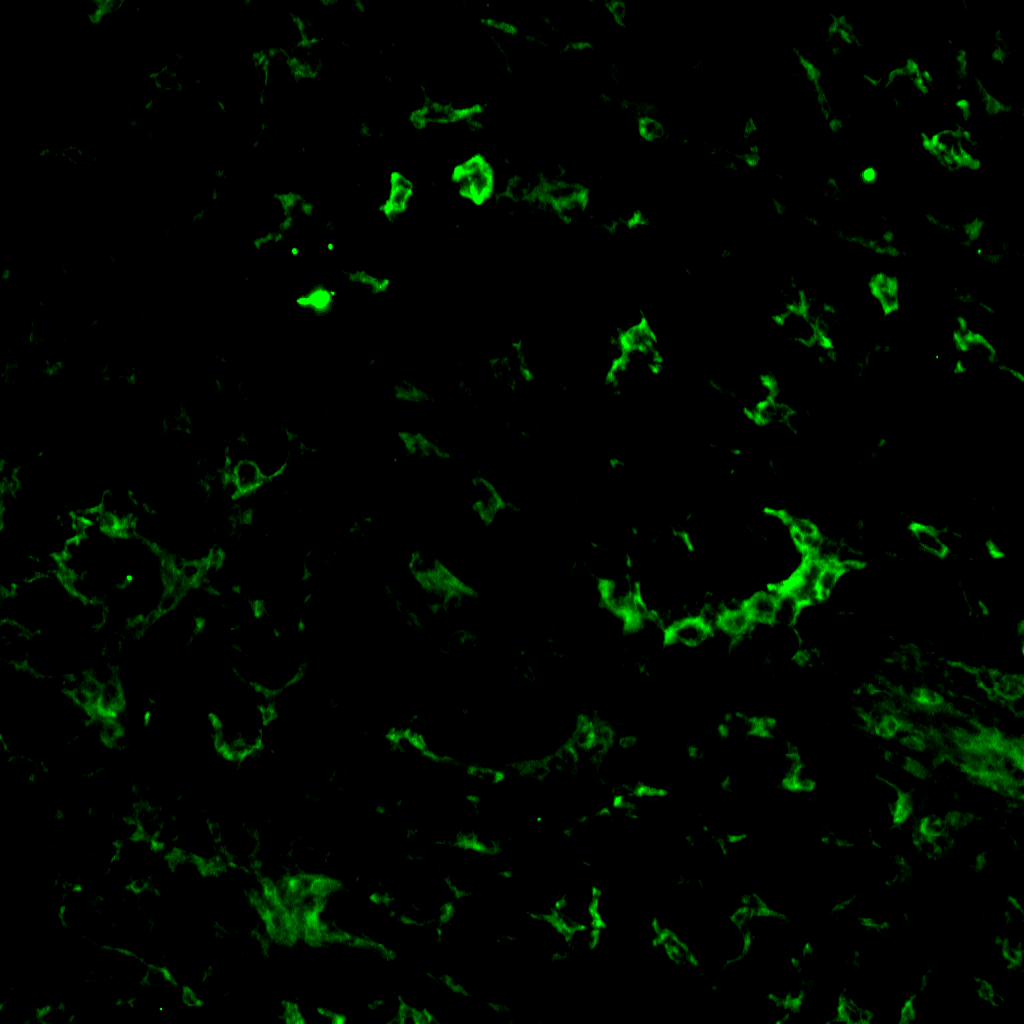

Supplement: Supplementary file 11 — Source data Fig. 7 [file 44318_2024_244_MOESM11_ESM.zip › Figure 7/7K/Vehicle F4 80.tif]

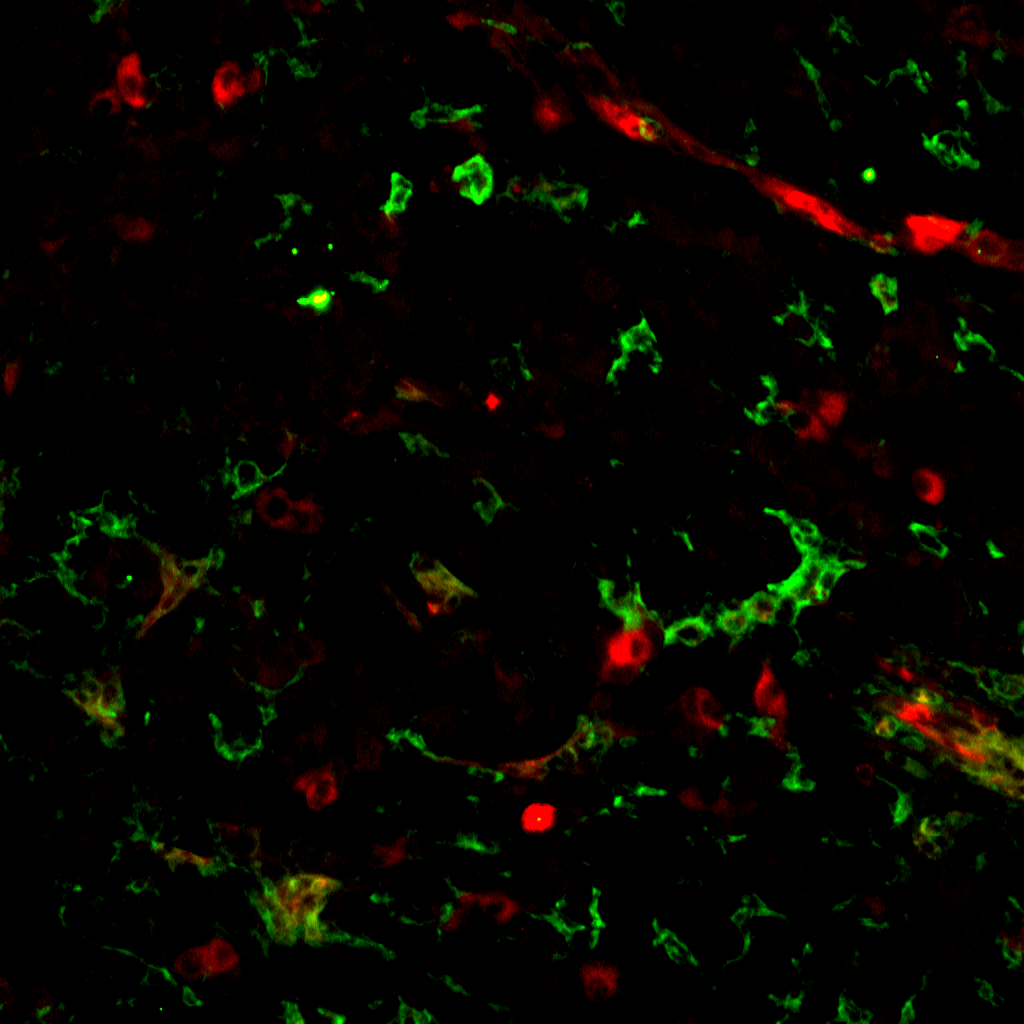

Supplement: Supplementary file 11 — Source data Fig. 7 [file 44318_2024_244_MOESM11_ESM.zip › Figure 7/7K/Vehicle Merge.tif]

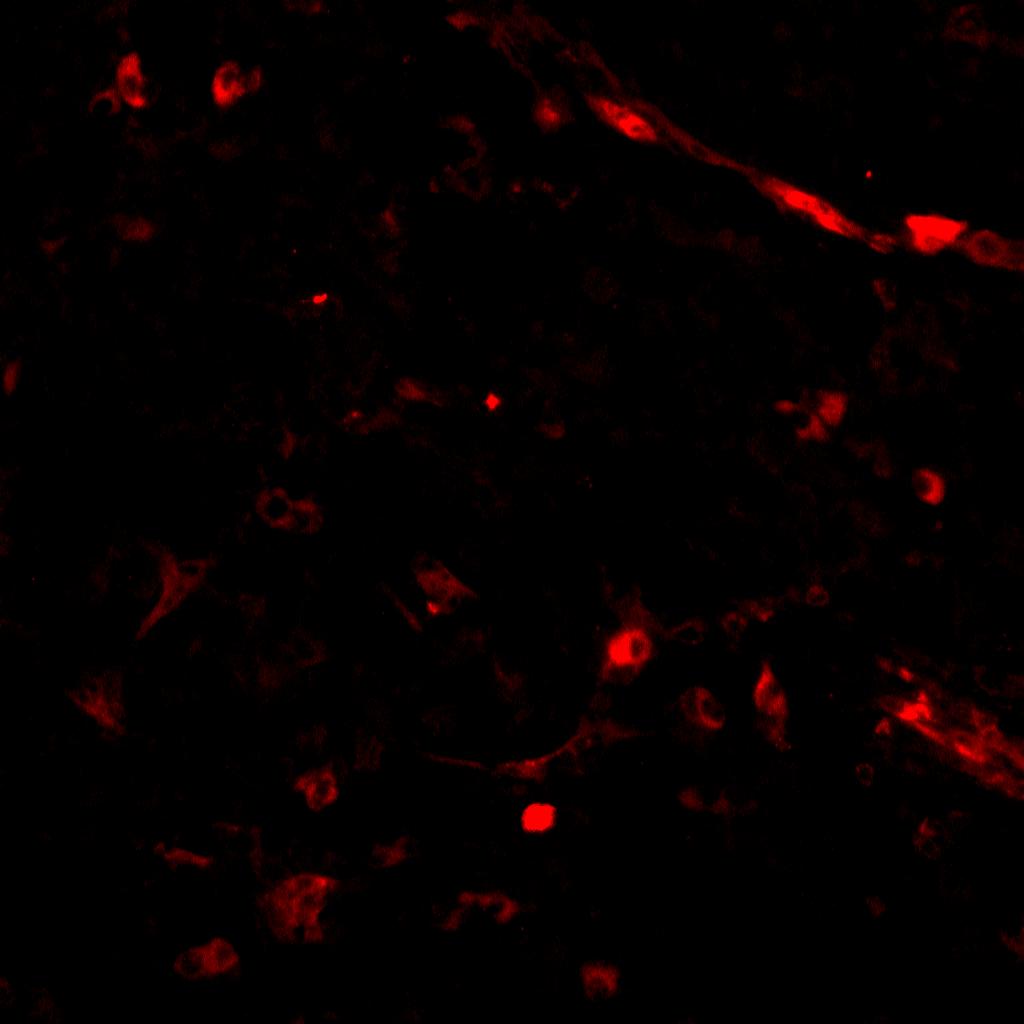

Supplement: Supplementary file 11 — Source data Fig. 7 [file 44318_2024_244_MOESM11_ESM.zip › Figure 7/7K/Vehicle pZyxin.tif]

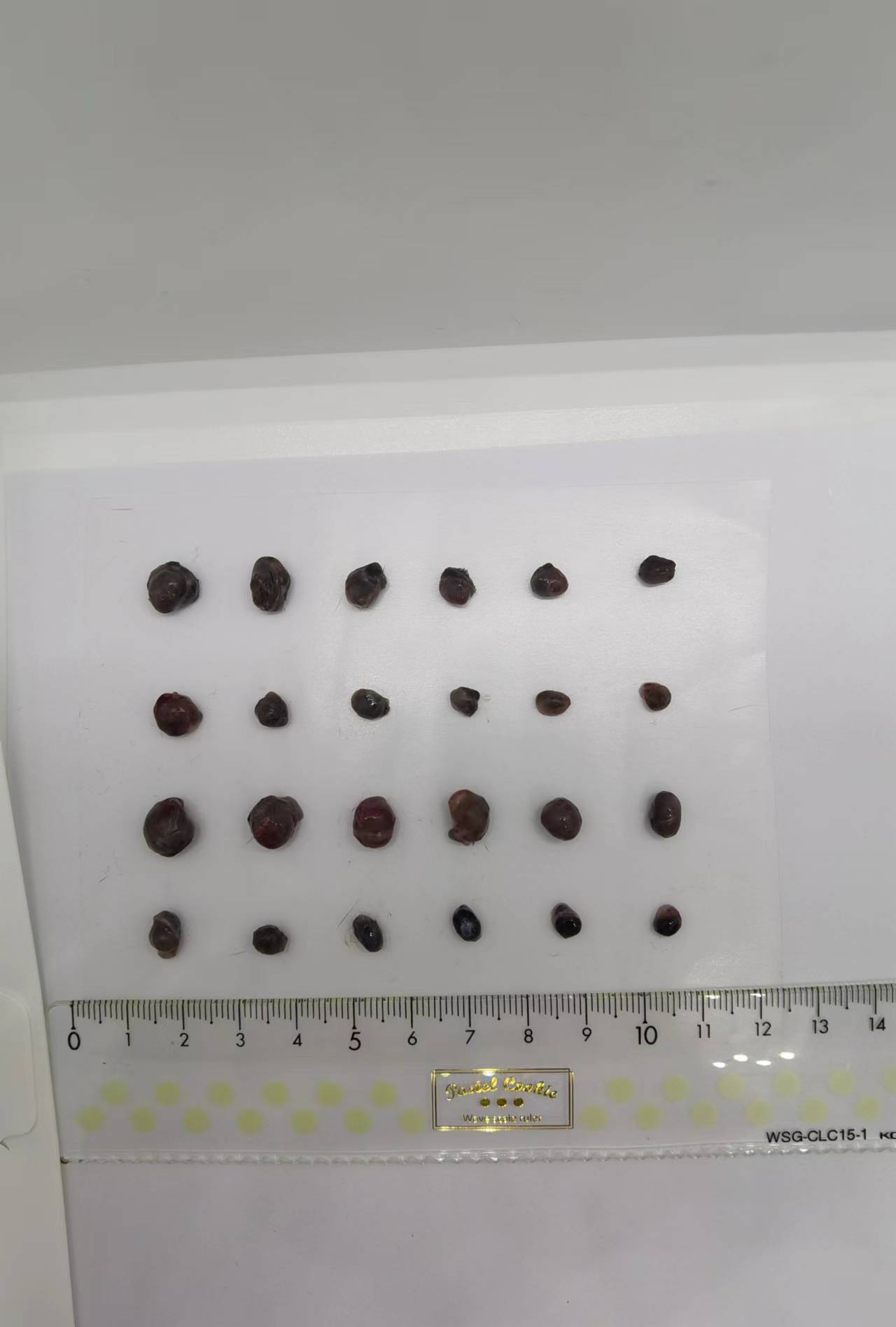

Supplement: Supplementary file 11 — Source data Fig. 7 [file 44318_2024_244_MOESM11_ESM.zip › Figure 7/7L/7L B16-F10 Inoculation.jpg]

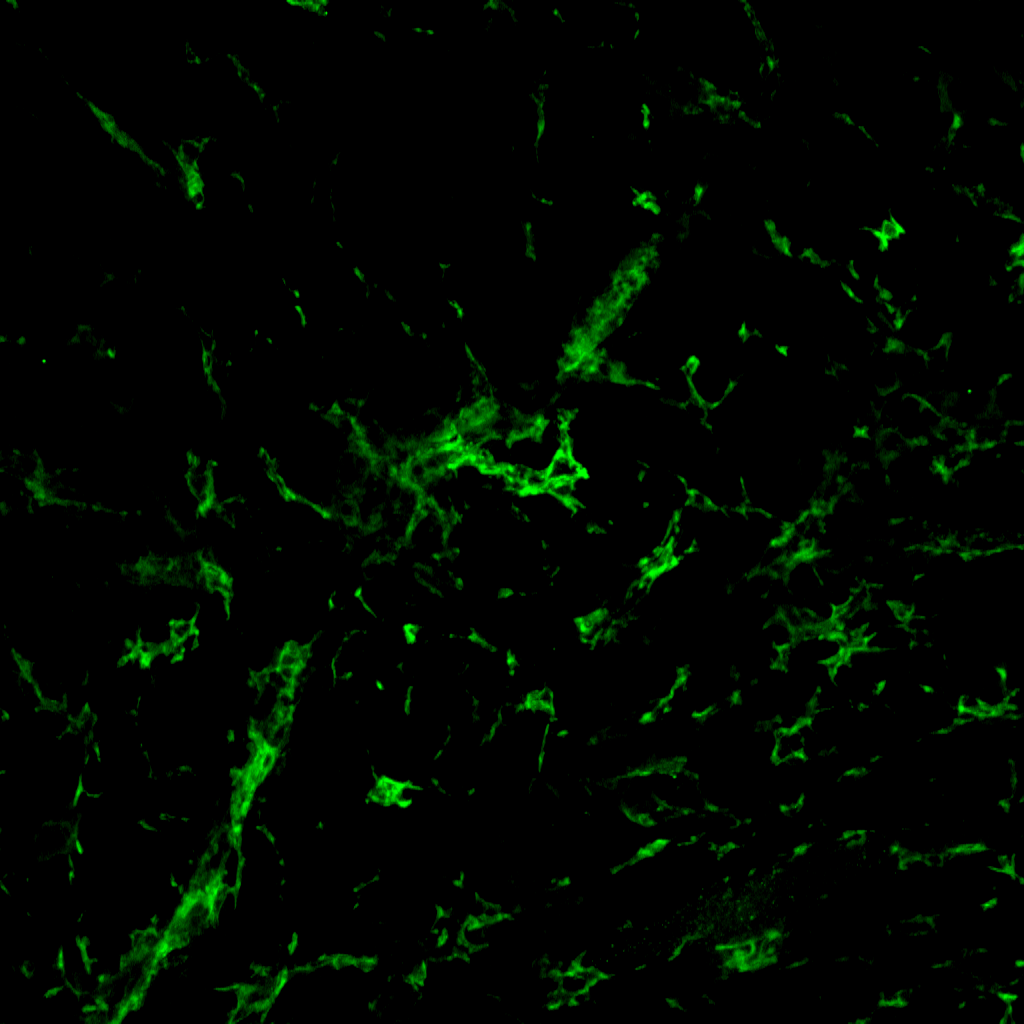

Supplement: Supplementary file 11 — Source data Fig. 7 [file 44318_2024_244_MOESM11_ESM.zip › Figure 7/7N/Tumor IRF3+ + Zyxin + + F4 80.tif]

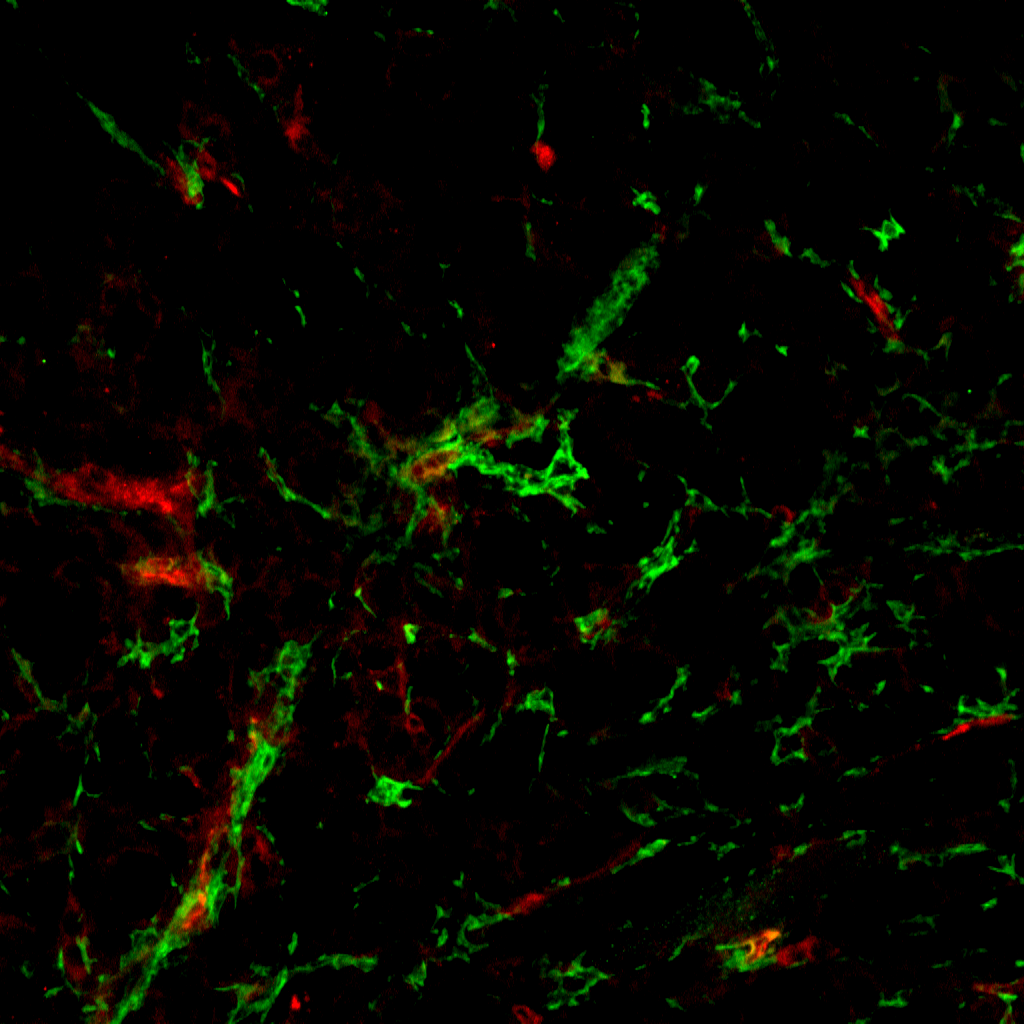

Supplement: Supplementary file 11 — Source data Fig. 7 [file 44318_2024_244_MOESM11_ESM.zip › Figure 7/7N/Tumor IRF3+ + Zyxin + + Merge.tif]

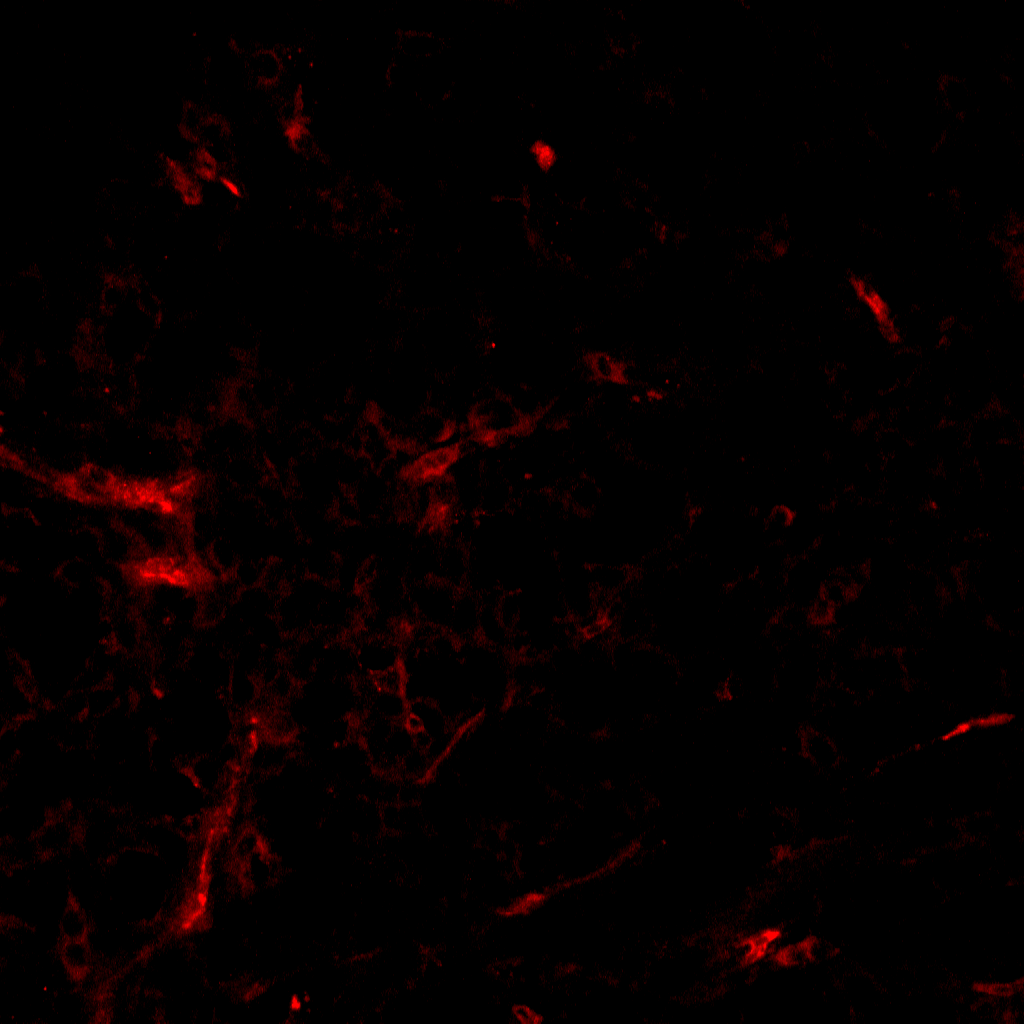

Supplement: Supplementary file 11 — Source data Fig. 7 [file 44318_2024_244_MOESM11_ESM.zip › Figure 7/7N/Tumor IRF3+ + Zyxin + + pZyxin.tif]

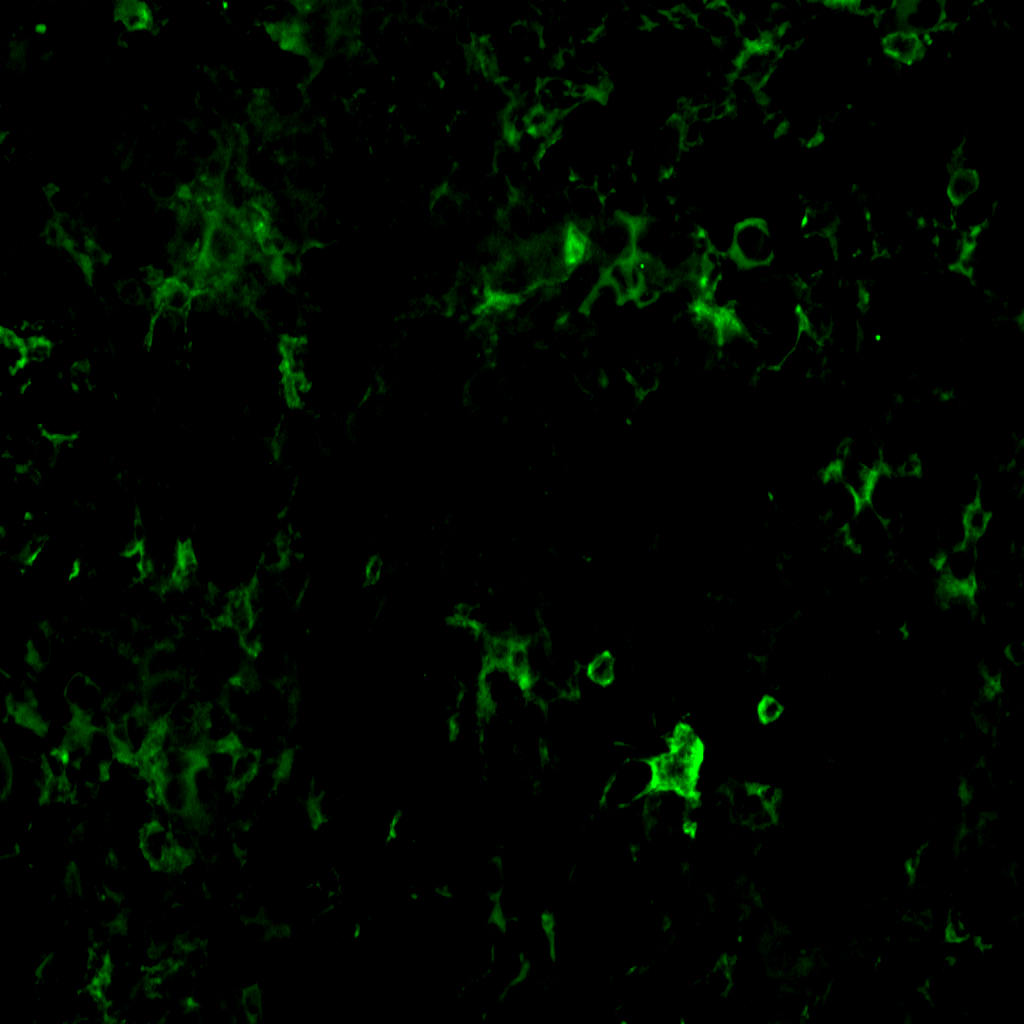

Supplement: Supplementary file 11 — Source data Fig. 7 [file 44318_2024_244_MOESM11_ESM.zip › Figure 7/7N/Tumor IRF3+ + Zyxin - - F4 80.tif]

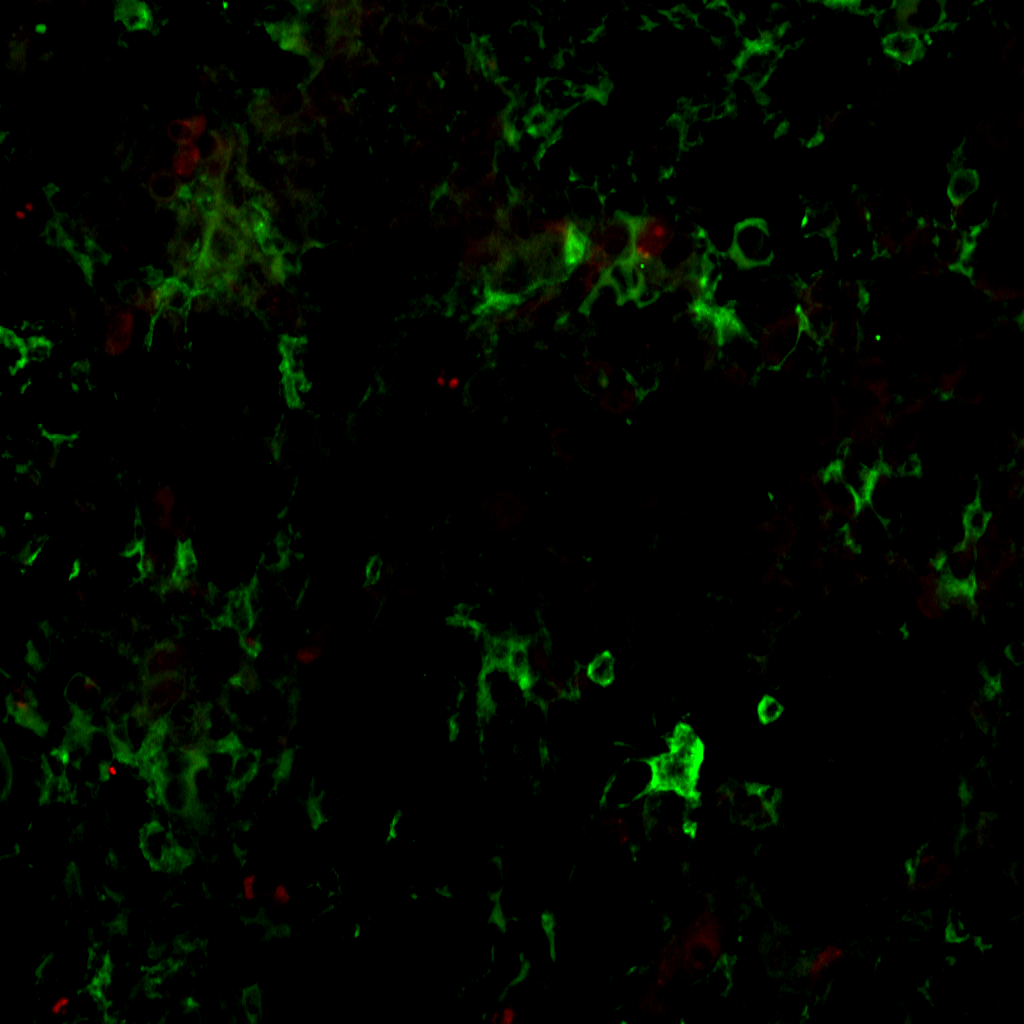

Supplement: Supplementary file 11 — Source data Fig. 7 [file 44318_2024_244_MOESM11_ESM.zip › Figure 7/7N/Tumor IRF3+ + Zyxin - - Merge.tif]

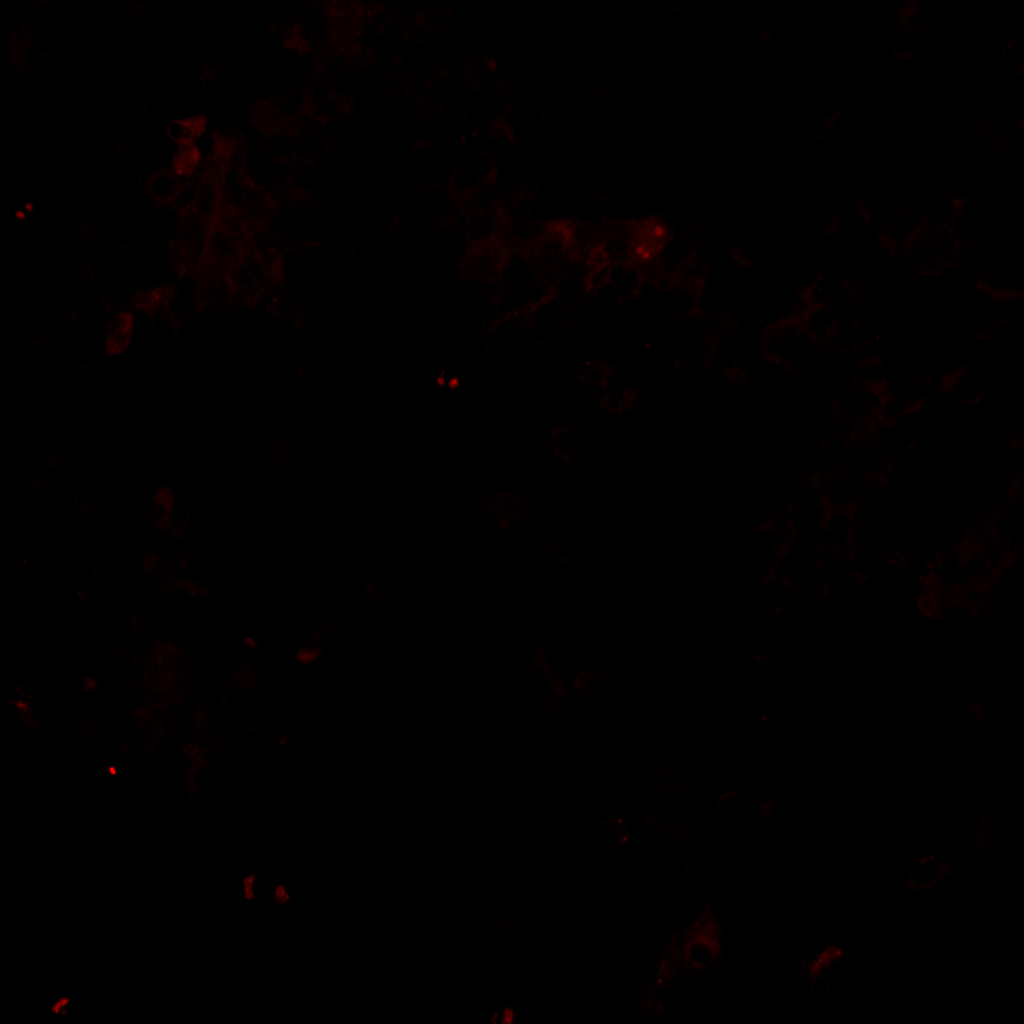

Supplement: Supplementary file 11 — Source data Fig. 7 [file 44318_2024_244_MOESM11_ESM.zip › Figure 7/7N/Tumor IRF3+ + Zyxin - - pZyxin.tif]

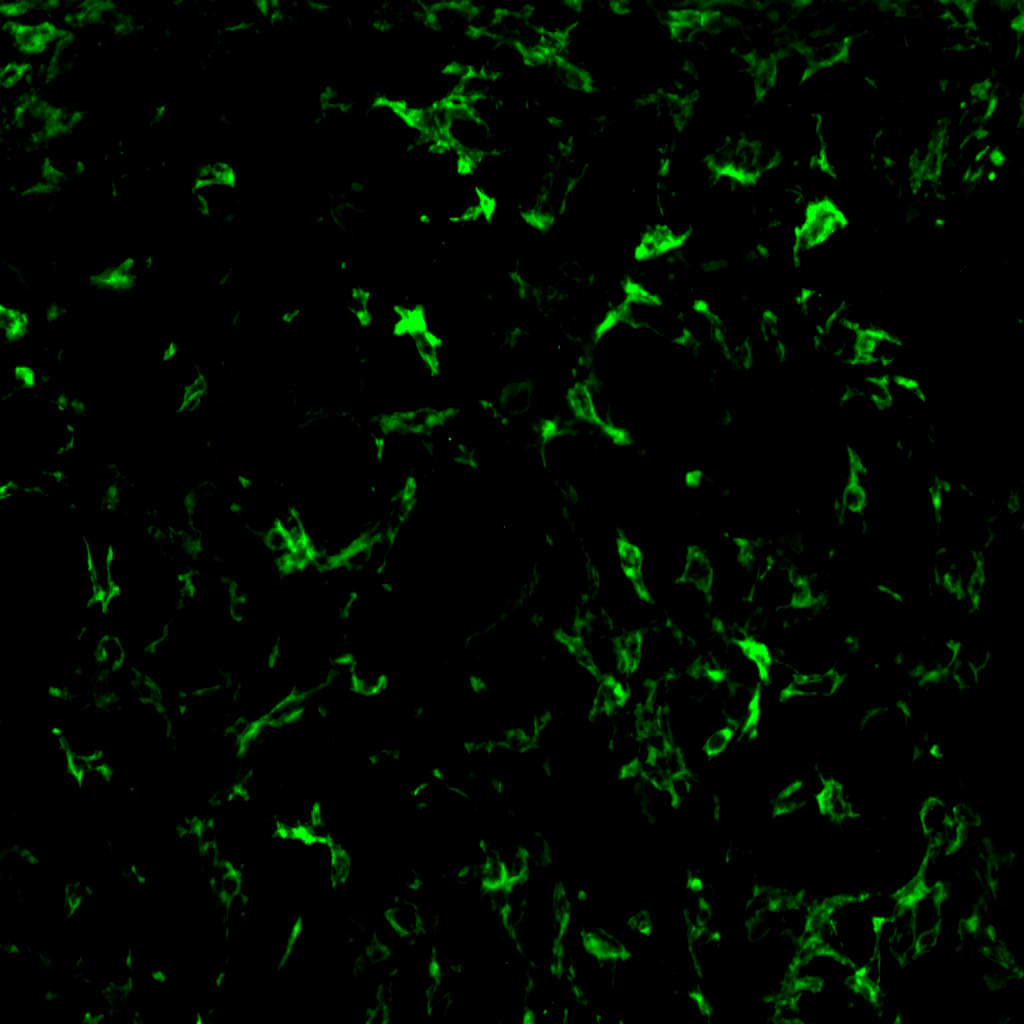

Supplement: Supplementary file 11 — Source data Fig. 7 [file 44318_2024_244_MOESM11_ESM.zip › Figure 7/7N/Tumor IRF3- - Zyxin + + F4 80.tif]

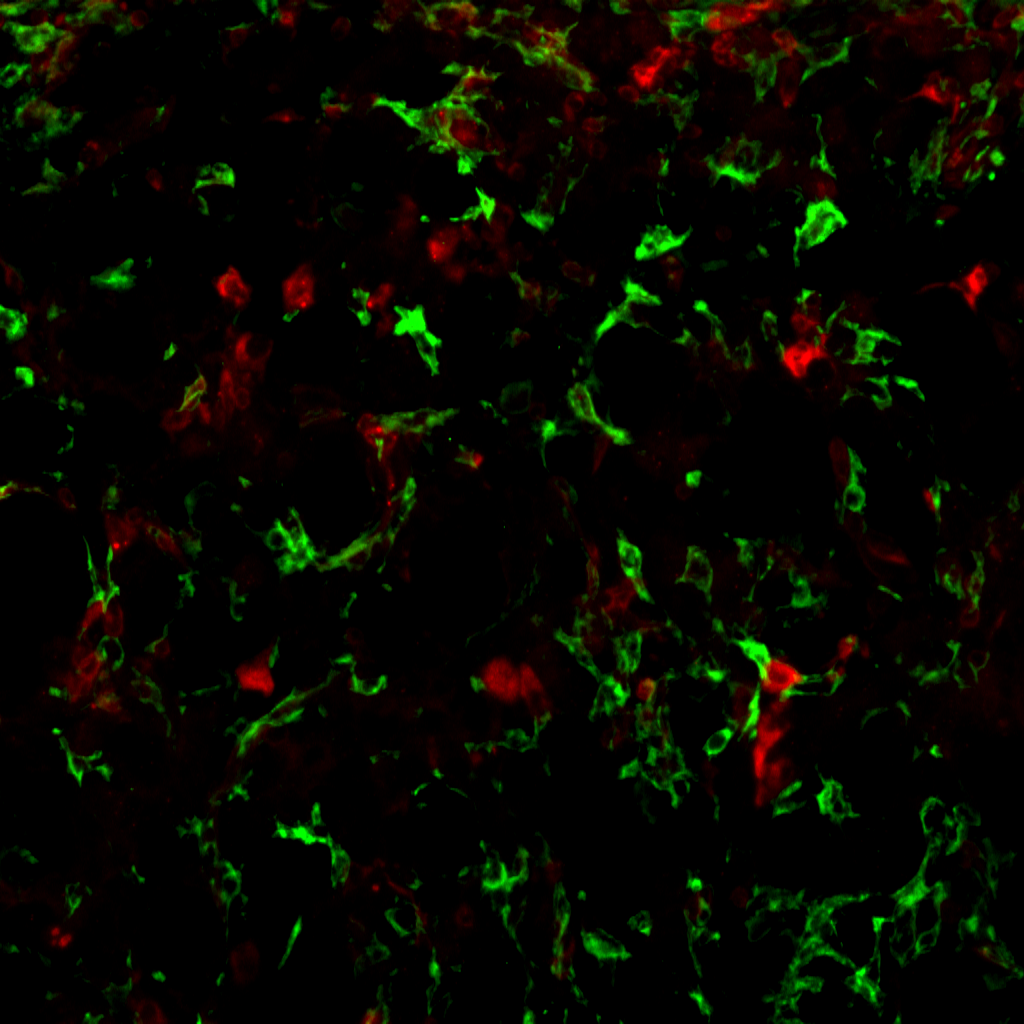

Supplement: Supplementary file 11 — Source data Fig. 7 [file 44318_2024_244_MOESM11_ESM.zip › Figure 7/7N/Tumor IRF3- - Zyxin + + Merge.tif]

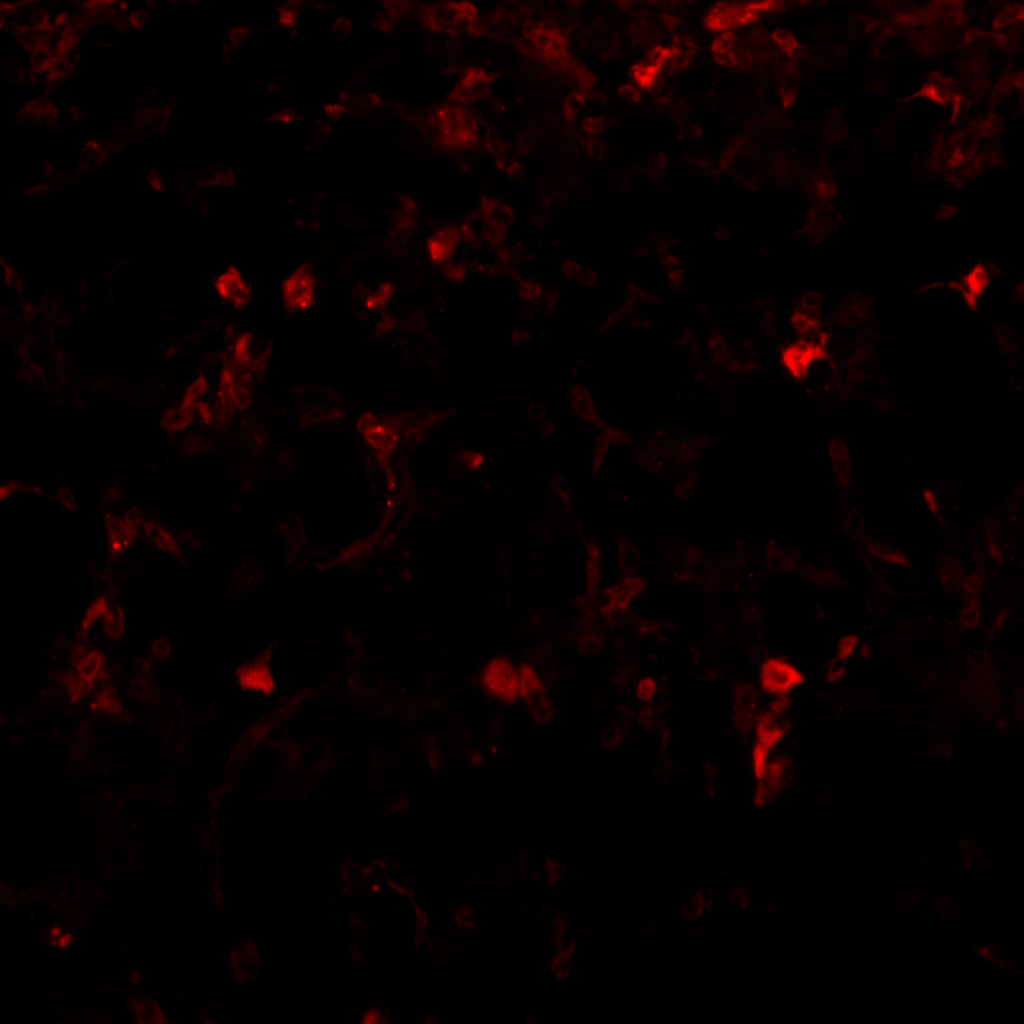

Supplement: Supplementary file 11 — Source data Fig. 7 [file 44318_2024_244_MOESM11_ESM.zip › Figure 7/7N/Tumor IRF3- - Zyxin + + pZyxin.tif]

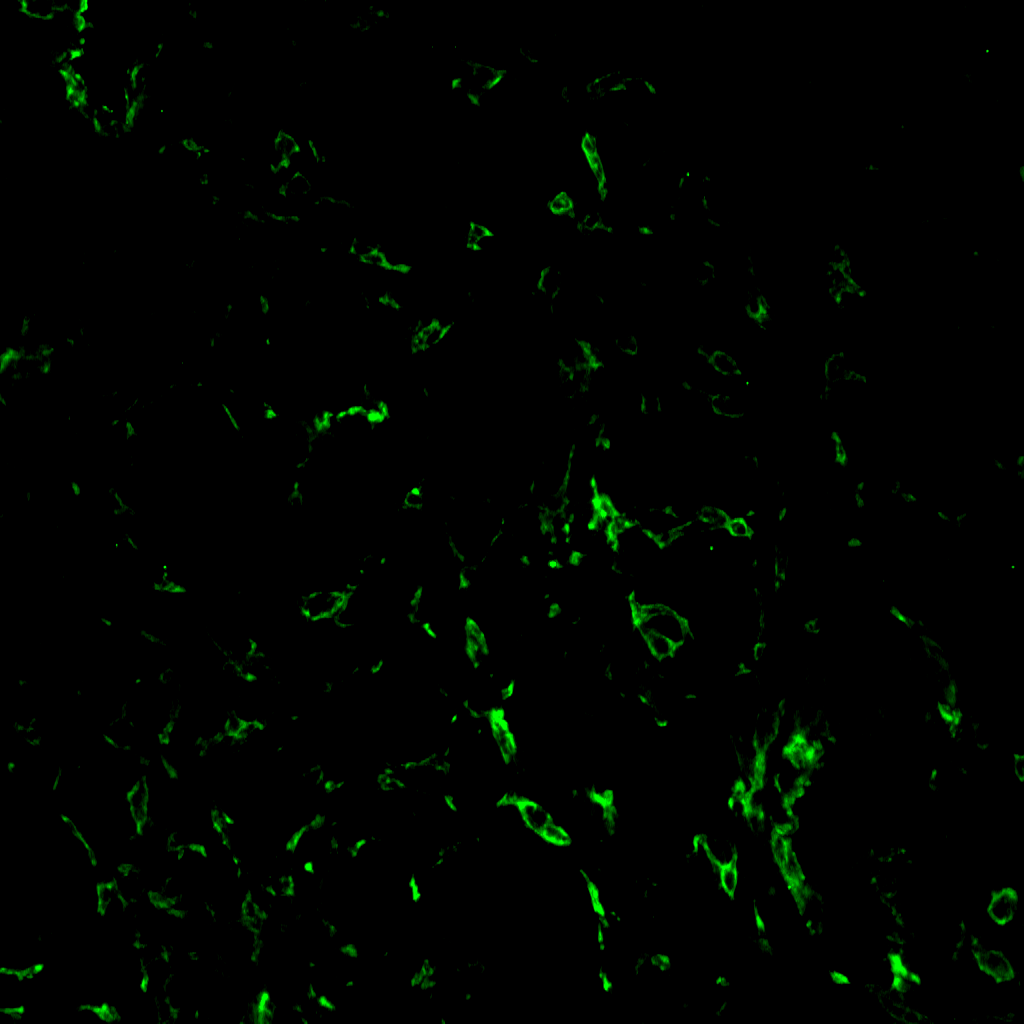

Supplement: Supplementary file 11 — Source data Fig. 7 [file 44318_2024_244_MOESM11_ESM.zip › Figure 7/7N/Tumor IRF3- - Zyxin- - F4 80.tif]

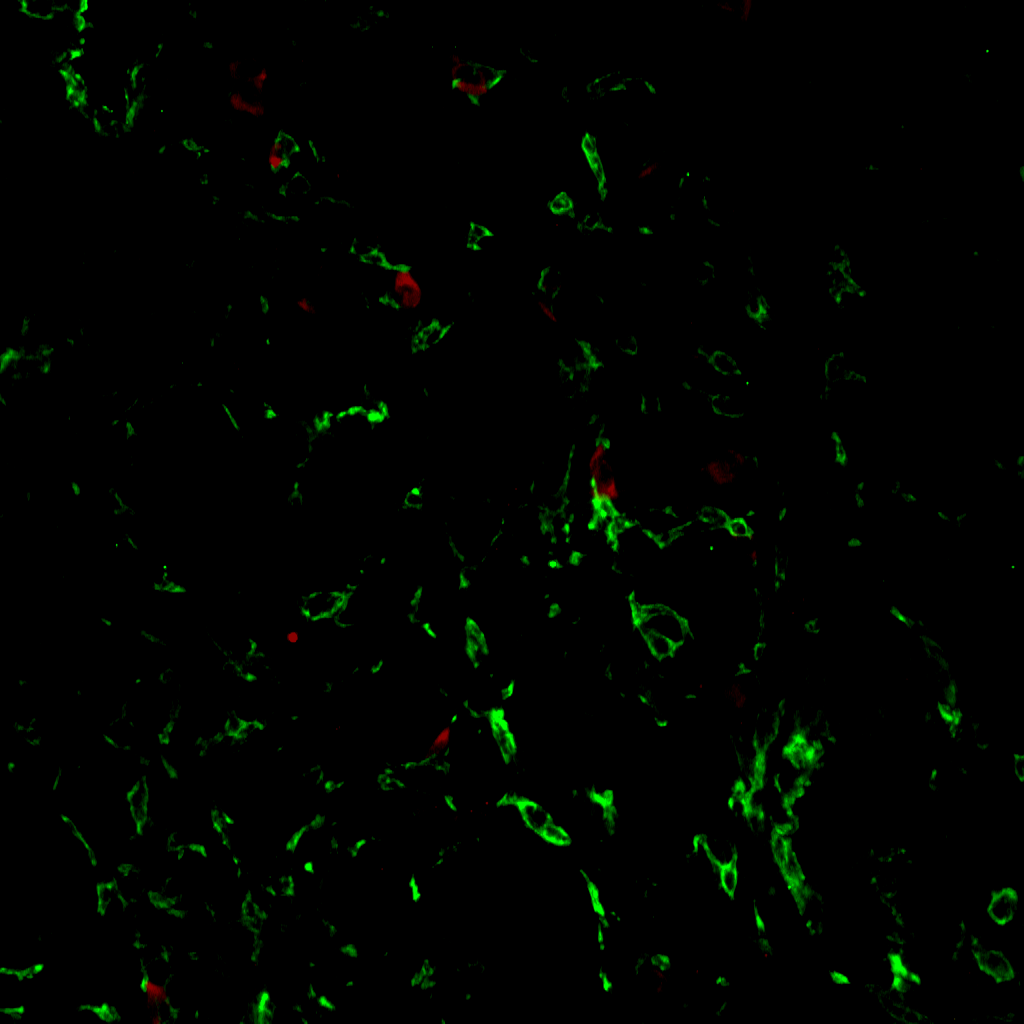

Supplement: Supplementary file 11 — Source data Fig. 7 [file 44318_2024_244_MOESM11_ESM.zip › Figure 7/7N/Tumor IRF3- - Zyxin- - Merge.tif]

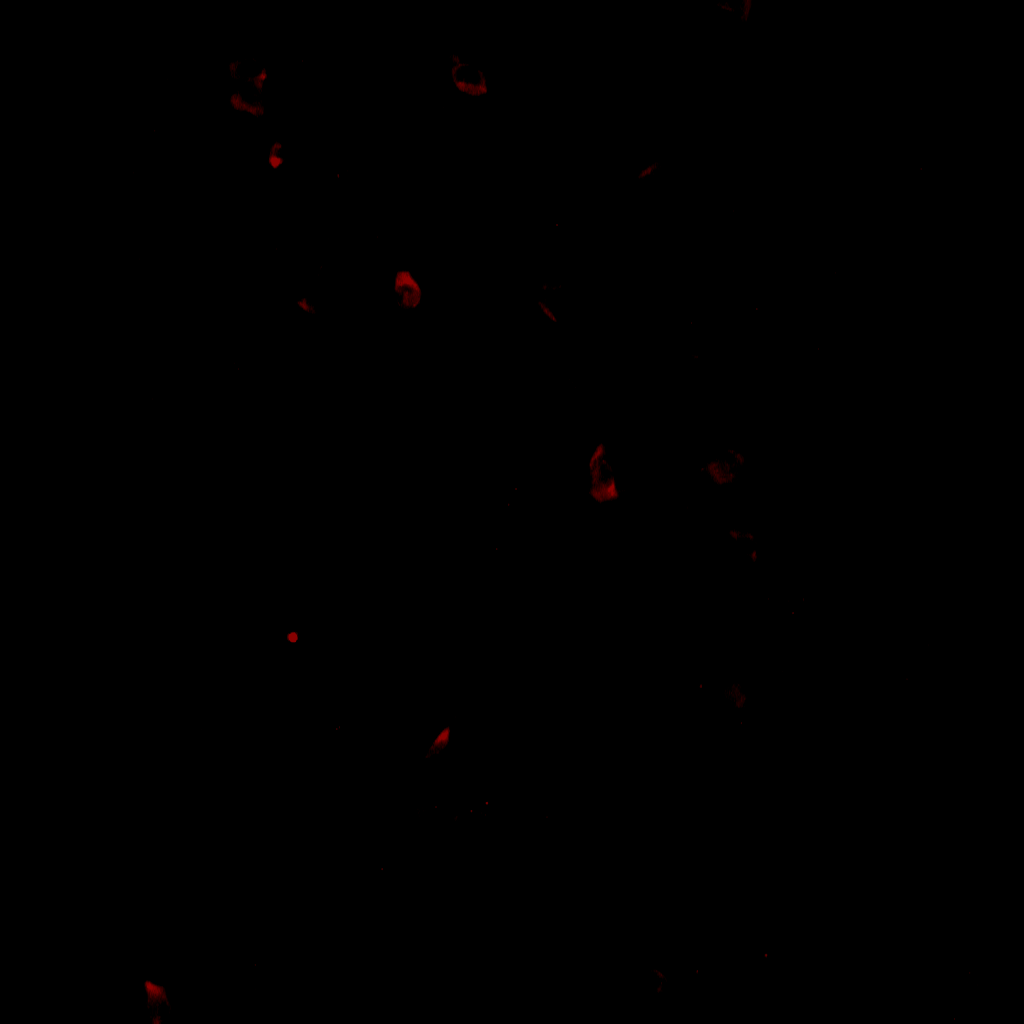

Supplement: Supplementary file 11 — Source data Fig. 7 [file 44318_2024_244_MOESM11_ESM.zip › Figure 7/7N/Tumor IRF3- - Zyxin- - pZyxin.tif]
