# Supplementary material for: TBK1-Zyxin signaling controls tumor-associated macrophage recruitment to mitigate antitumor immunity
Source: EMBO J. 2024 Sep 20;43(21):4984–5017. doi: 10.1038/s44318-024-00244-9 (PMC11535546; doi:10.1038/s44318-024-00244-9)
Supplement: Supplementary file 12 — Expanded View Figures [file 44318_2024_244_MOESM12_ESM.pdf]

Expanded View Figures

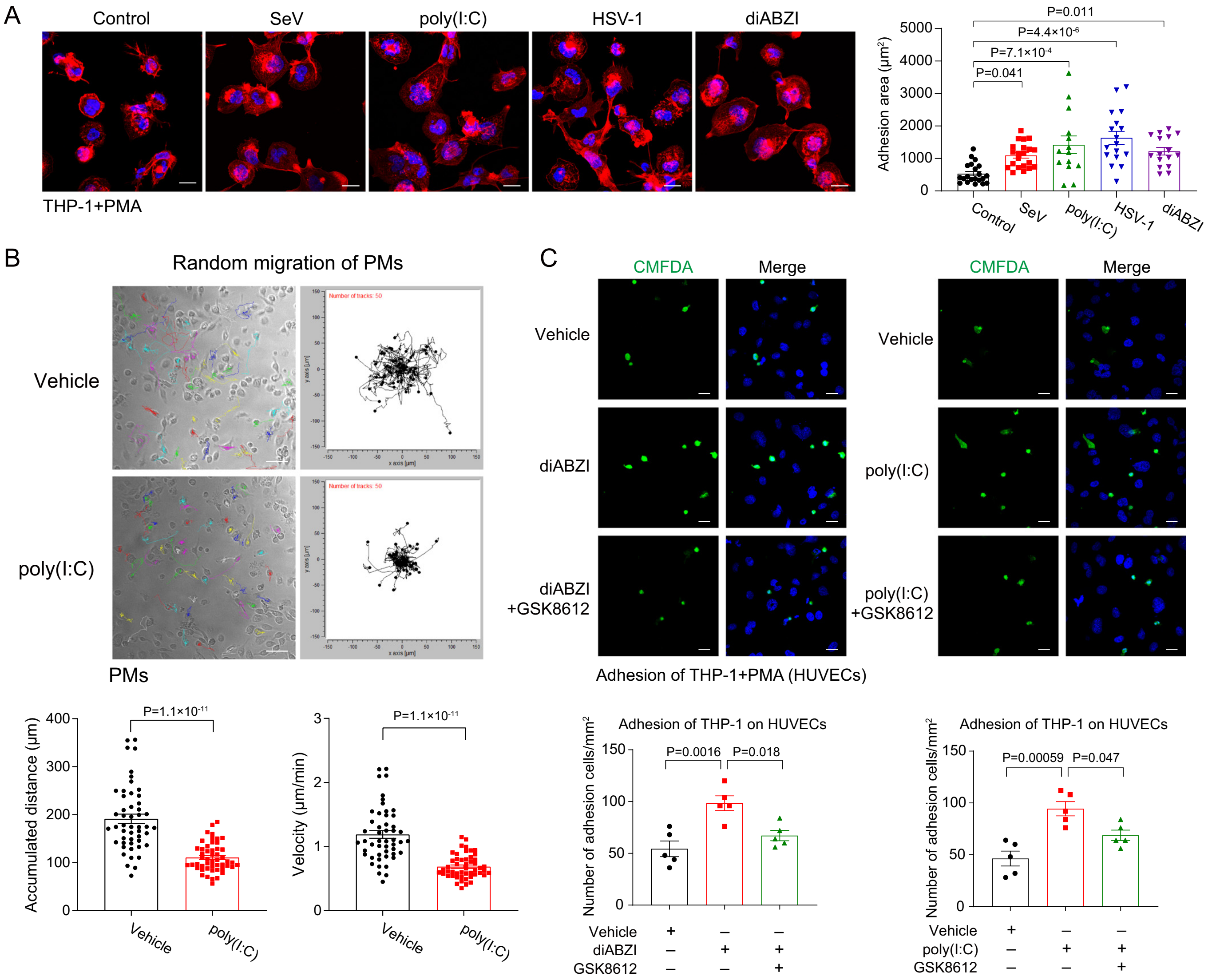

Figure EV1. Nucleic acid sensing regulates the adhesion and motility of macrophages.

(**A**) THP-1 cells, human monocytes, were differentiated into macrophages with PMA treatment, and their adhesion and migration were analyzed under the effects of SeV (RNA virus), poly(I:C) (RNA analog), HSV-1 (DNA virus), or diABZI (STING agonist). Scale bar, 20 μm. Control group, $n = 21$; SeV group, $n = 21$; poly(I:C) group, $n = 14$; HSV-1 group, $n = 17$; diABZI group, $n = 16$. (**B**) Representative migration plots indicated the movement of PMs upon the treatment of vehicle or poly(I:C). Statistics of the accumulated distance and velocity of cell movement were revealed. $n = 50$ cells per group. Scale bar, 100 μm. (**C**) diABZI or poly(I:C) promoted THP-1 adhesion to HUVECs, which was compromised by GSK8612, a TBK1 inhibitor. THP-1 cells were labeled by 5-chloromethyl fluorescein diacetate (CMFDA, green). Scale bar, 20 μm. $n = 5$ per group. Data information: unless otherwise indicated, $n = 3$ independent biological experiments (mean ± SEM). *$P < 0.05$, **$P < 0.01$, and ***$P < 0.001$, compared with the control condition (One-way ANOVA test and Bonferroni correction).

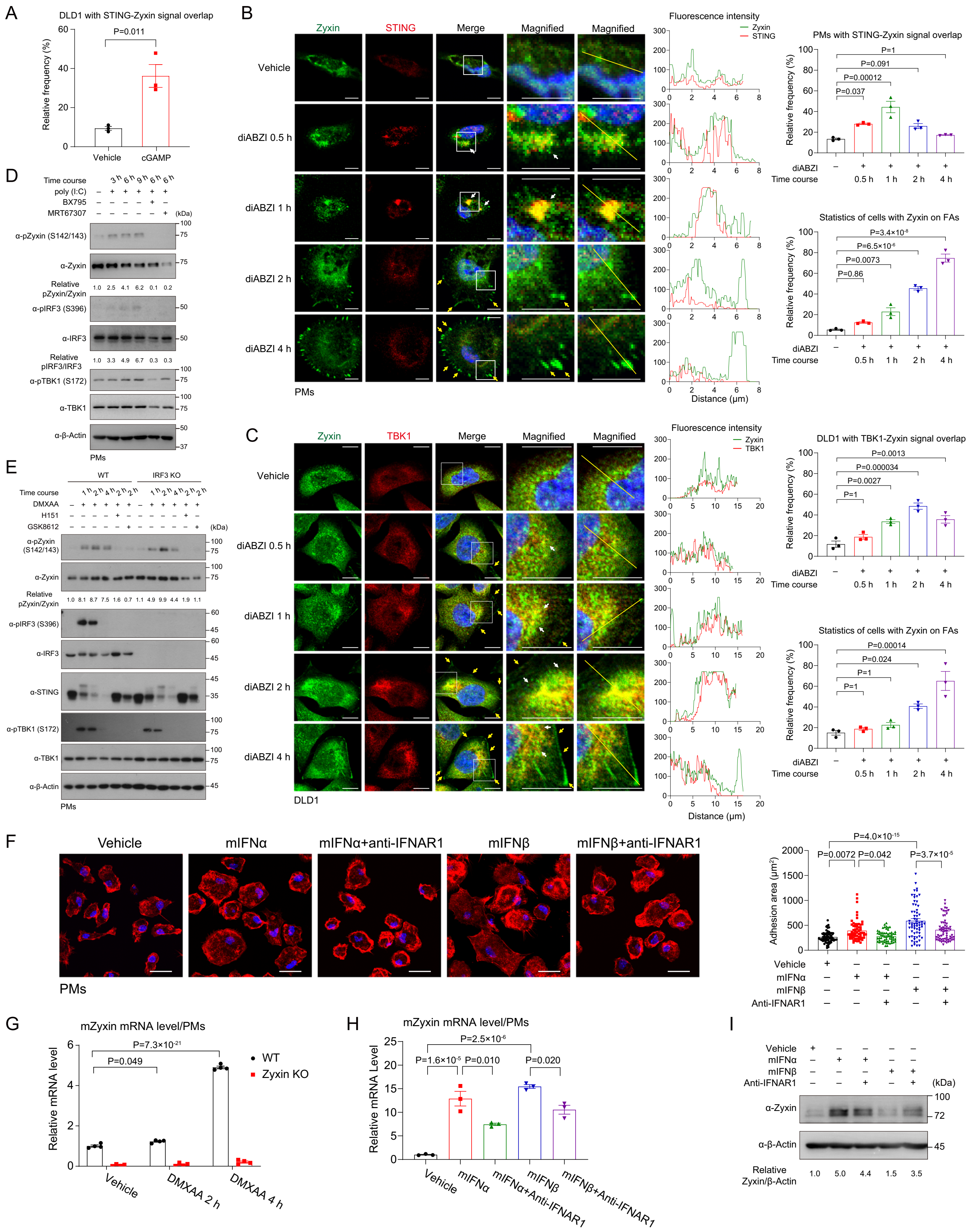
A
DLD1 with STING-Zyxin signal overlap
Relative frequency (%)
P=0.011
Vehicle
cGAMP
B
Zyxin
STING
Merge
Magnified
Magnified
Fluorescence intensity
Vehicle
diABZI 0.5 h
diABZI 1 h
diABZI 2 h
diABZI 4 h
PMs
Distance (μm)
PMs with STING-Zyxin signal overlap
P=1
P=0.091
P=0.00012
P=0.037
diABZI
Time course
Statistics of cells with Zyxin on FAs
P=3.4×10-8
P=6.5×10-6
P=0.0073
P=0.86
D
Time course
poly (I:C)
BX795
MRT67307
α-pZyxin (S142/143)
α-Zyxin
Relative pZyxin/Zyxin
1.0 2.5 4.1 6.2 0.1 0.2
α-pIRF3 (S396)
α-IRF3
Relative pIRF3/IRF3
1.0 3.3 4.9 6.7 0.3 0.3
α-pTBK1 (S172)
α-TBK1
α-β-Actin
PMs
E
WT
IRF3 KO
Time course
DMXAA
H151
GSK8612
α-pZyxin (S142/143)
α-Zyxin
Relative pZyxin/Zyxin
1.0 8.1 8.7 7.5 1.6 0.7 1.1 4.9 9.9 4.4 1.9 1.1
α-pIRF3 (S396)
α-IRF3
α-STING
α-pTBK1 (S172)
α-TBK1
α-β-Actin
PMs
C
Zyxin
TBK1
Merge
DLD1
DLD1 with TBK1-Zyxin signal overlap
P=0.0013
P=0.000034
P=0.0027
P=1
Statistics of cells with Zyxin on FAs
P=0.00014
P=0.024
P=1
P=1
F
Vehicle
mIFNα
mIFNα+anti-IFNAR1
mIFNβ
mIFNβ+anti-IFNAR1
PMs
Adhesion area (μm²)
P=4.0×10-15
P=0.0072 P=0.042
P=3.7×10-5
Vehicle
mIFNα
mIFNβ
Anti-IFNAR1
G
mZyxin mRNA level/PMs
Relative mRNA level
P=7.3×10-21
P=0.049
WT
Zyxin KO
Vehicle
DMXAA 2 h
DMXAA 4 h
H
mZyxin mRNA level/PMs
Relative mRNA Level
P=2.5×10-6
P=1.6×10-5 P=0.010
P=0.020
Vehicle
mIFNα
mIFNα+Anti-IFNAR1
mIFNβ
mIFNβ+Anti-IFNAR1
I
Vehicle
mIFNα
mIFNβ
Anti-IFNAR1
(kDa)
α-Zyxin
α-β-Actin
Relative Zyxin/β-Actin
1.0 5.0 4.4 1.5 3.5

 © The Author(s)

Figure EV2. STING or MAVS signalosomes recruit and phosphorylate Zyxin.

(**A**) Statistics of cGAMP-induced Zyxin-STING signal overlap in Fig. 2B were shown. $n = 3$ per group. (**B**) Immunofluorescence imaging and statistics indicated an association of endogenous Zyxin proteins with STING signalosomes (white arrowed) in response to stimulation and its sequential distribution on focal adhesions (yellow arrowed) in primary macrophages. Scale bar, 5 μm. $n = 3$ per group. (**C**) Immunofluorescence and statistics showed diABZI induced an association of endogenous Zyxin with TBK1 signalosomes (white arrowed) and its sequential distribution on focal adhesions (yellow arrowed) in DLD1 cells. Scale bar, 5 μm. $n = 3$ per group. (**D**) Poly(I:C)-induced RNA sensing triggered Zyxin phosphorylation at S142/S143 residues in PMs. (**E**) Genetic ablation of IRF3 in primary macrophages failed to eliminate the DMXAA-induced phosphorylation of TBK1, STING, and Zyxin. (**F**) mIFNα (100 U, 6 h) or mIFNβ (100 U, 6 h) increased PM adhesion area, which was reversed upon treating anti-INFAR1 neutralizing. Scale bar, 20 μm. Vehicle group, $n = 68$; mIFNα group, $n = 63$; mIFNα+Anti-IFNAR1 group, $n = 59$; mIFNβ group, $n = 64$; mIFNβ+Anti-IFNAR1 group, $n = 65$. (**G**) An elevated level of Zyxin mRNA was detected upon the activation of cGAS-STING signaling at 4 h. $n = 4$ per group. (**H, I**) mIFNα (100 U, 6 h) and mIFNβ (100 U, 6 h) induced an increase in mRNA (**H**) and protein levels of Zyxin (**I**), which was reversed by an anti-IFNAR1 neutralizing antibody. (**H**) $n = 3$ per group. Data information: unless otherwise indicated, $n = 3$ independent biological experiments (mean ± SEM). $*P < 0.05$, $**P < 0.01$, and $***P < 0.001$, compared with the control condition (One-way ANOVA test and Bonferroni correction).

© The Author(s)

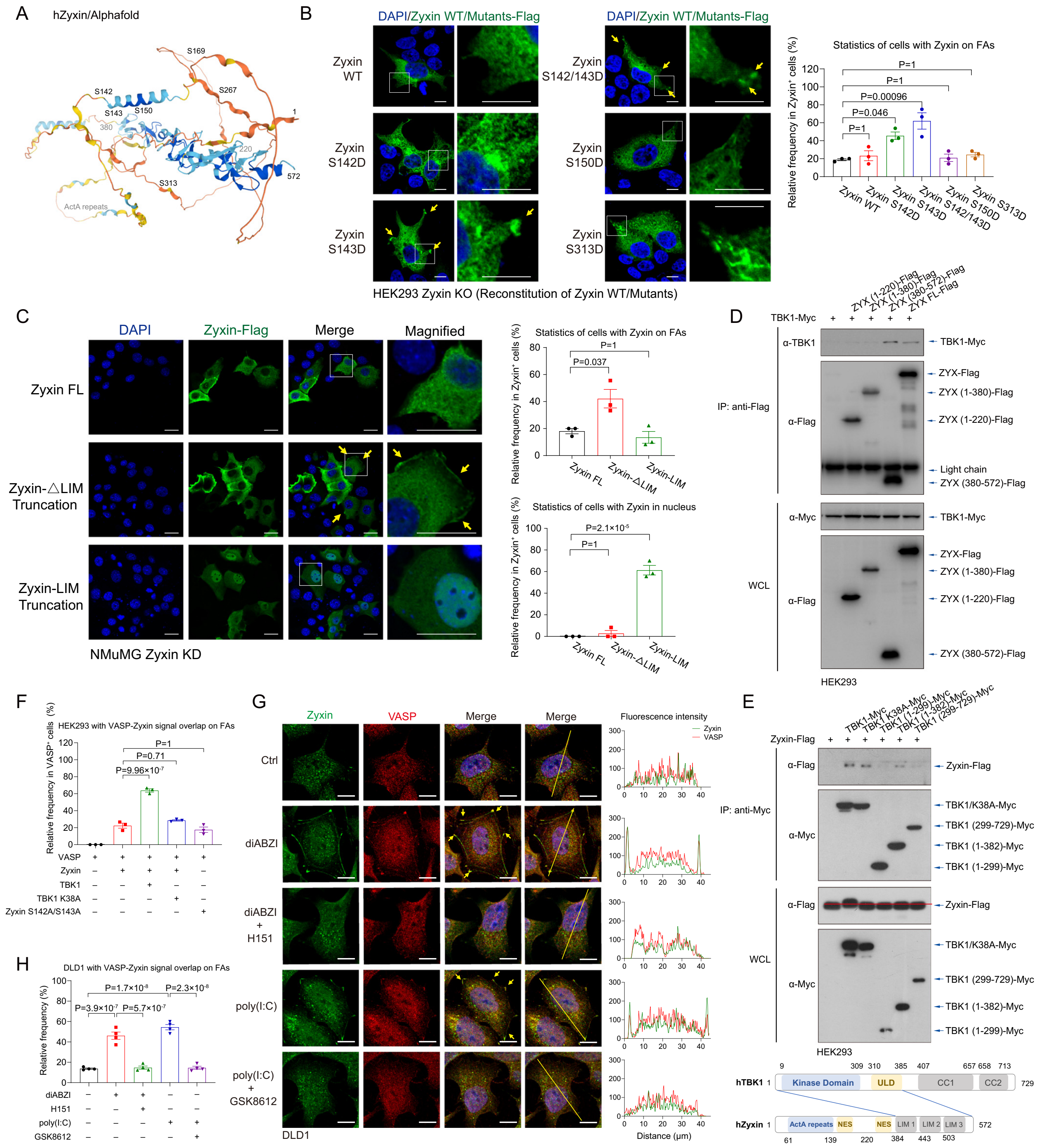

A
hZyxin/Alphafold
B
DAPI/Zyxin WT/Mutants-Flag
Statistics of cells with Zyxin on FAs
HEK293 Zyxin KO (Reconstitution of Zyxin WT/Mutants)
C
DAPI
Zyxin-Flag
Merge
Magnified
Zyxin FL
Zyxin-△LIM Truncation
Zyxin-LIM Truncation
NMuMG Zyxin KD
Statistics of cells with Zyxin in nucleus
D
IP: anti-Flag
WCL
HEK293
E
F
HEK293 with VASP-Zyxin signal overlap on FAs
G
Zyxin
VASP
Fluorescence intensity
Distance (μm)
DLD1
H
DLD1 with VASP-Zyxin signal overlap on FAs
hTBK1
Kinase Domain
ULD
CC1
CC2
hZyxin
ActA repeats
NES
LIM 1
LIM 2
LIM 3

 © The Author(s)

Figure EV3. TBK1 directly phosphorylates S143 residue to facilitate Zyxin focal adhesion localization.

(**A**) Alphafold predicted the protein structure of human Zyxin proteins, implying an interaction between a long α-helix containing S143 and the C-terminal LIM domain. (**B**) Zyxin phosphomimetic mutants mimicking TBK1-mediated phosphorylation were individually reconstituted in Zyxin KO HEK293 cells; immunofluorescence imaging and statistics revealed a substantial subset of Zyxin S143D or S142D/S143D mutant localized on focal adhesions (yellow arrowed), in contradiction to wild-type Zyxin. Scale bar, 10 µm. $n = 3$ per group. (**C**) Immunofluorescence imaging and statistics indicated the cellular localization of Zyxin truncations in NMuMG cells. $n = 3$ per group. (**D**, **E**) Domain mapping by coimmunoprecipitations between TBK1 or Zyxin truncations showed that the kinase domain of TBK1 (a.a. 1–382) and the LIM domain of Zyxin (a.a. 380–572) were responsible for their mutual interaction. (**F**) Statistics of Zyxin-VASP signal overlap on focal adhesions in HEK293 cells are shown in Fig. 3K. $n = 3$ per group. (**G**, **H**) The signal overlap of endogenous Zyxin and VASP on focal adhesions (yellow arrowed) in DLD1 cells under TBK1 activation (diABZI or poly(I:C), 4 h) or inhibition (STING inhibitor H151 or TBK1 inhibitor GSK8612), respectively. Scale bar, 10 µm. (**H**) $n = 4$ per group. Data information: unless otherwise indicated, $n = 3$ independent biological experiments (mean ± SEM). *$P < 0.05$, **$P < 0.01$, and ***$P < 0.001$, compared with the control condition (One-way ANOVA test and Bonferroni correction).

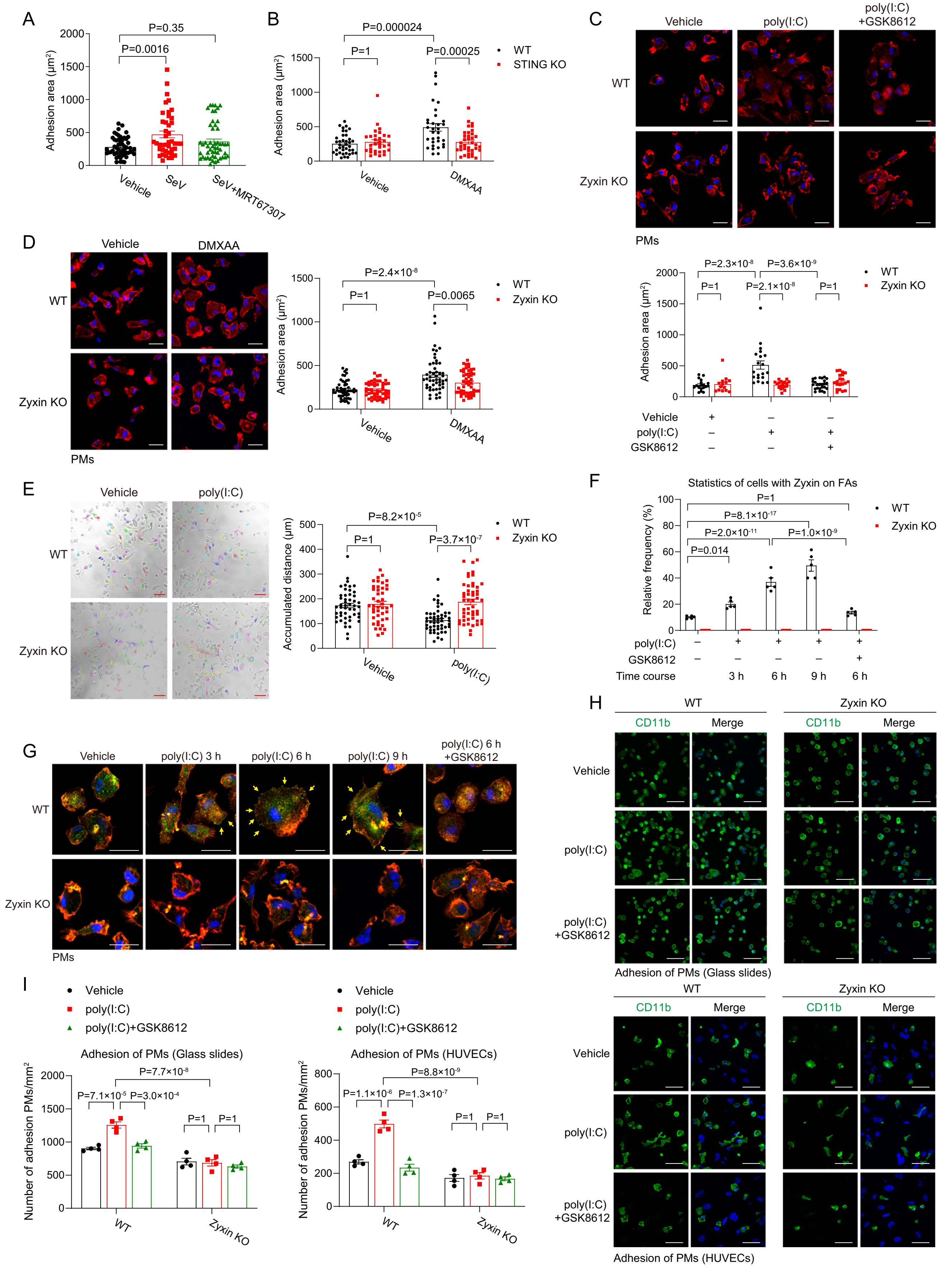

 © The Author(s)

Figure EV4. The TBK1-Zyxin cascade restricts macrophage motility.

(**A**, **B**) Statistics revealed that SeV infection upregulated the adhesion area of PMs, blocked by TBK1 inhibitor MRT67307 (**A**) and dependent on STING (**B**). (**A**) Vehicle group, $n = 52$; SeV group, $n = 42$; SeV+MRT67307 group, $n = 51$; (**B**) WT Vehicle group, $n = 38$; WT DMXAA group, $n = 32$; STING KO Vehicle group, $n = 31$; STING KO DMXAA group, $n = 36$. (**C**) Immunofluorescence and statistics revealed the adhesion of Zyxin KO macrophages upon the activation of innate RNA sensing by poly(I:C) transfection. The enhanced cellular adhesion induced by poly(I:C) was attenuated upon Zyxin deletion. Scale bar, 20 µm. WT Vehicle group, $n = 18$; WT poly(I:C) group, $n = 20$; WT poly(I:C) + GSK8612 group, $n = 25$; Zyxin KO Vehicle group, $n = 14$; Zyxin KO poly(I:C) group, $n = 19$; Zyxin KO poly(I:C) + GSK8612 group, $n = 20$. (**D**) Genetic ablation of Zyxin attenuated STING signaling-enhanced cellular adhesion, as revealed by immunofluorescence imaging and statistics of PMs from *Zyxin*$^{+/+}$ or *Zyxin*$^{-/-}$ mice treated with DMXAA (10 µg/mL, 4 h). Scale bar, 20 µm. $n = 50$ per group. (**E**) Macrophage motility inhibited by poly(I:C) transfection was relieved upon Zyxin deletion. Scale bar, 100 µm. WT Vehicle group, $n = 49$; WT poly(I:C) group, $n = 50$; Zyxin KO Vehicle group, $n = 44$; Zyxin KO poly(I:C) group, $n = 50$. (**F**, **G**) Immunofluorescence and statistics indicated Zyxin distribution at focal adhesions (indicated by the yellow arrows), induced by poly(I:C) transfection, was blocked by either TBK1 inhibitor GSK8612 or Zyxin deletion. Scale bar, 20 µm. (**F**) $n = 5$ per group. (**H**, **I**) Poly(I:C) transfection promoted the adhesion of murine PMs on glass slides and HUVEC, a process restored upon TBK1 inhibition or Zyxin deletion. Scale bar, 50 µm. (**I**) $n = 4$ per group. Data information: unless otherwise indicated, $n = 3$ independent biological experiments (mean ± SEM). $^{*}P < 0.05$, $^{**}P < 0.01$, and $^{***}P < 0.001$, compared with the control condition (one-way ANOVA test and Bonferroni correction).

© The Author(s)

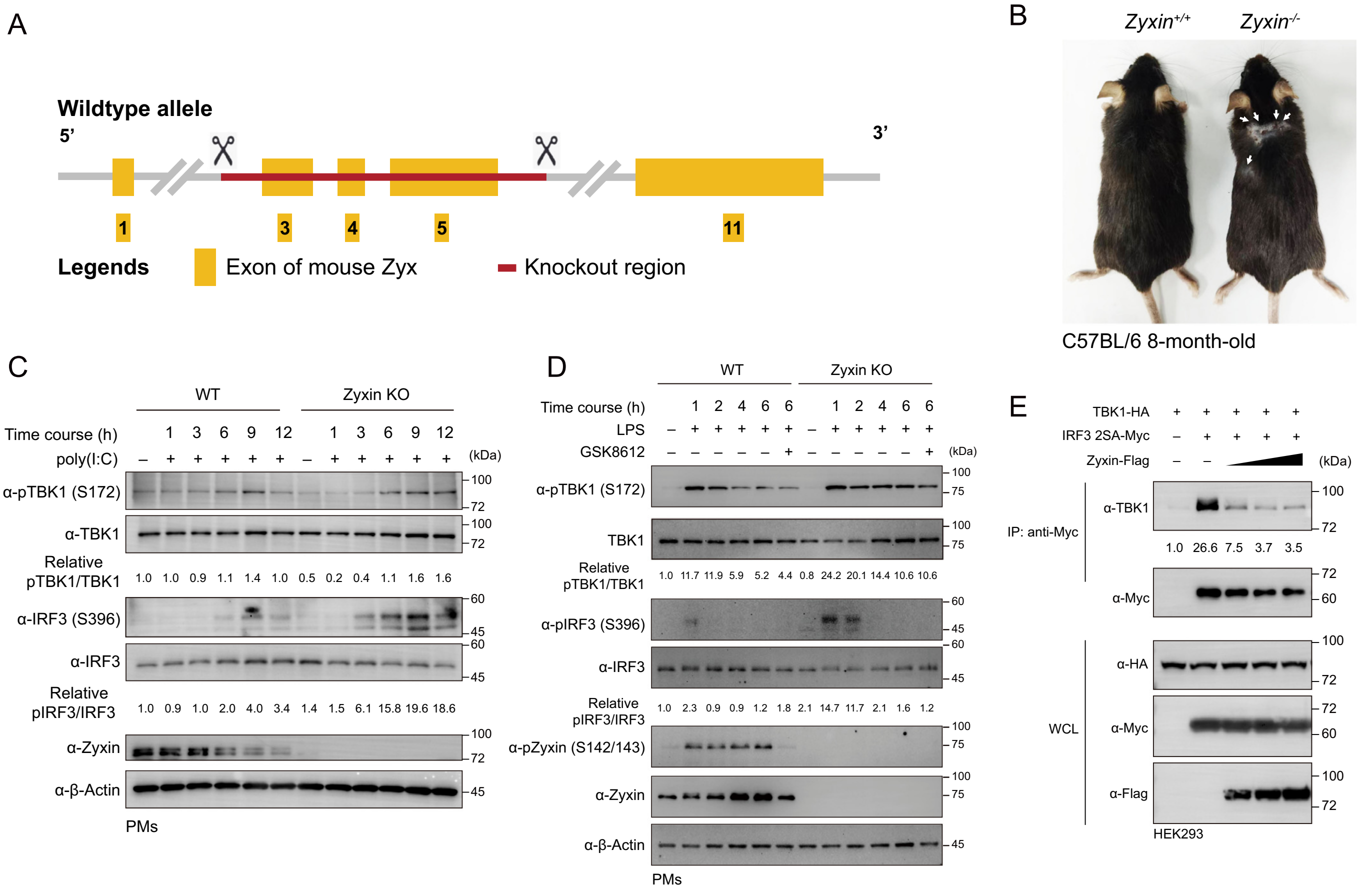

Figure EV5. Zyxin deficiency enhances inflammatory responses.

(**A**, **B**) The strategy of generating Zyxin KO mice by CRISPR-mediated genome editing was described (**A**), and spontaneous skin lesions in 8-month-old Zyxin knockout mice were found (**B**). (**C**, **D**) Poly(I:C)-induced RNA sensing and LPS-induced TLR4 signaling moderately increased pIRF3 (S396) levels in Zyxin-deficient cells. (**E**) Coimmunoprecipitation assays to detect the TBK1-IRF3 interaction in the absence or presence of Zyxin.

 © The Author(s)

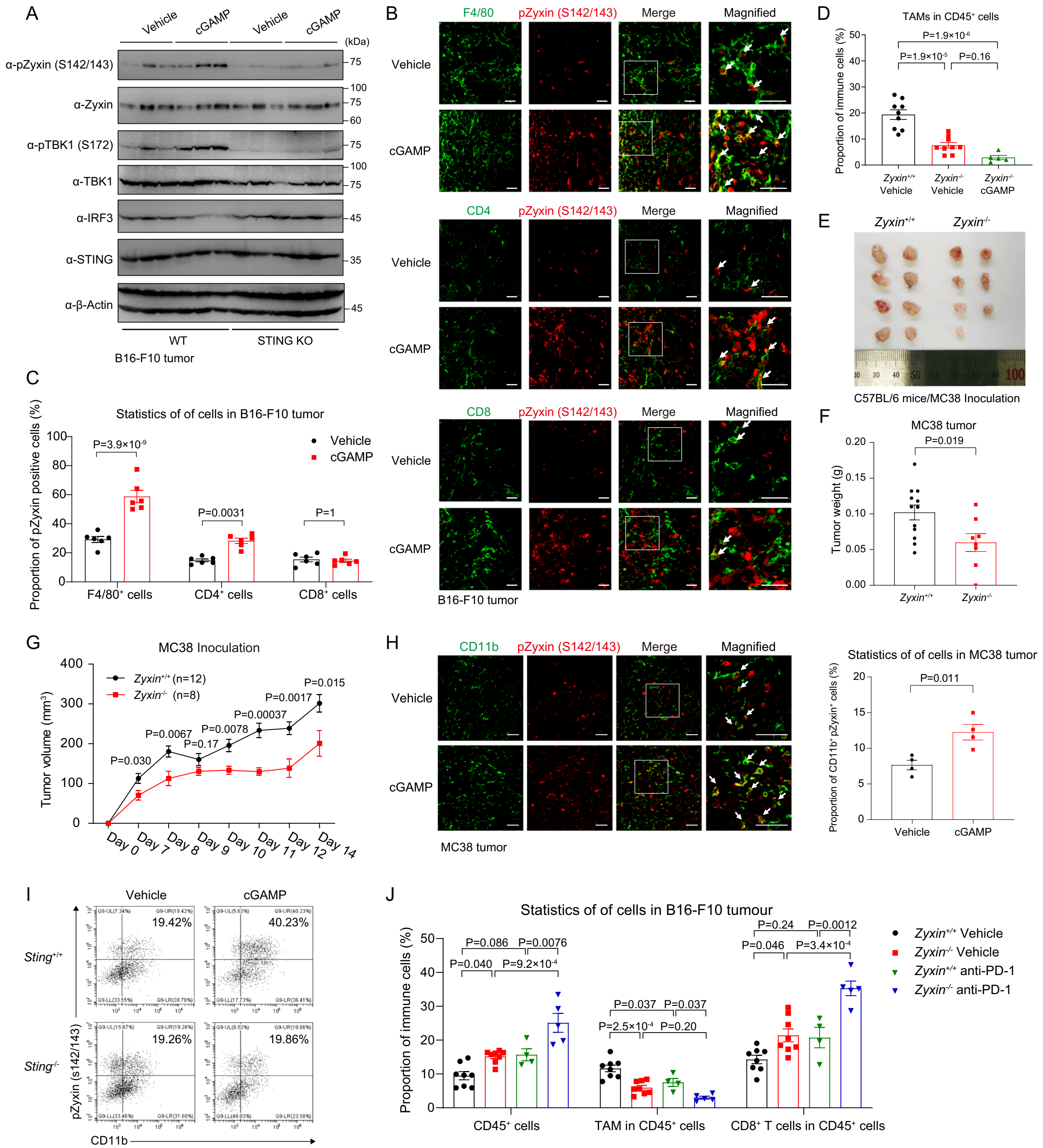
A
Vehicle
cGAMP
Vehicle
cGAMP
(kDa)
α-pZyxin (S142/143)
α-Zyxin
α-pTBK1 (S172)
α-TBK1
α-IRF3
α-STING
α-β-Actin
WT
STING KO
B16-F10 tumor
B
F4/80
pZyxin (S142/143)
Merge
Magnified
Vehicle
cGAMP
CD4
CD8
B16-F10 tumor
C
Statistics of of cells in B16-F10 tumor
Proportion of pZyxin positive cells (%)
F4/80+ cells
CD4+ cells
CD8+ cells
D
TAMs in CD45+ cells
Proportion of immune cells (%)
E
Zyxin+/+
Zyxin-/-
C57BL/6 mice/MC38 Inoculation
F
MC38 tumor
Tumor weight (g)
G
MC38 Inoculation
Tumor volume (mm3)
H
CD11b
MC38 tumor
Statistics of of cells in MC38 tumor
I
Sting+/+
Sting-/-
pZyxin (s142/143)
CD11b
J
Statistics of of cells in B16-F10 tumour
Zyxin+/+ Vehicle
Zyxin-/- Vehicle
Zyxin+/+ anti-PD-1
Zyxin-/- anti-PD-1
CD45+ cells
TAM in CD45+ cells
CD8+ T cells in CD45+ cells

© The Author(s)

Figure EV6. Interventing in STING-TBK1-Zyxin signaling improves antitumor immunity and synergizes with PD-1 immunotherapy.

(**A**) Intratumoral injection of cGAMP induced the signal of phospho-Zyxin (S142/143) in melanoma specimens from WT mice but not STING KO mice. (**B**, **C**) Images and statistics showed cGAMP administration in mice induced the phospho-Zyxin mainly in F4/80^{+} macrophages and, to a lesser extent, in CD4^{+} T cells (indicated by the white arrows). Scale bars, 50 µm. (**C**) $n = 6$ per group. (**D**) Wild-type or Zyxin KO B16-F10 melanoma specimens in the absence or presence of cGAMP were analyzed by flow cytometry, and statistical results of TAMs (CD11b^{+} F4/80^{+}) were displayed. *Zyxin*$^{+/+}$ Vehicle group, $n = 9$; *Zyxin*$^{-/-}$ Vehicle group, $n = 9$; *Zyxin*$^{-/-}$ cGAMP group, $n = 5$. (**E–G**) Knockout of Zyxin compromised the growth of MC38 colon adenocarcinoma in the syngeneic tumor model. (**F**, **G**) *Zyxin*$^{+/+}$ group, $n = 12$; *Zyxin*$^{-/-}$ group, $n = 8$. (**H**, **I**) Immunofluorescence and flow cytometry showed that cGAMP increased pZyxin (S142/143) levels, majorly in myeloid cells (CD11b^{+}) of tumor tissues. STING KO mice showed a decreased proportion of CD11b^{+}pZyxin^{+} cells. Scale bars, 50 µm. (**H**) $n = 4$ per group. (**J**) Statistics represented the individual abundance of TAMs (CD11b^{+} F4/80^{+}) and CD8^{+} T cells in B16-F10 tumors analyzed by flow cytometry, showing a substantially low proportion of TAMs and a high proportion of CD8^{+} T cells under the combinational condition of Zyxin KO and anti-PD-1 administration. *Zyxin*$^{+/+}$ Vehicle group, $n = 8$; *Zyxin*$^{-/-}$ Vehicle group, $n = 8$; *Zyxin*$^{+/+}$ anti-PD-1 group, $n = 4$; *Zyxin*$^{-/-}$ anti-PD-1 group, $n = 5$. Data information: unless otherwise indicated, $n = 3$ independent biological experiments (mean ± SEM). *$P < 0.05$, **$P < 0.01$, and ***$P < 0.001$, compared with the control condition (One-way ANOVA test and Bonferroni correction).

 © The Author(s)

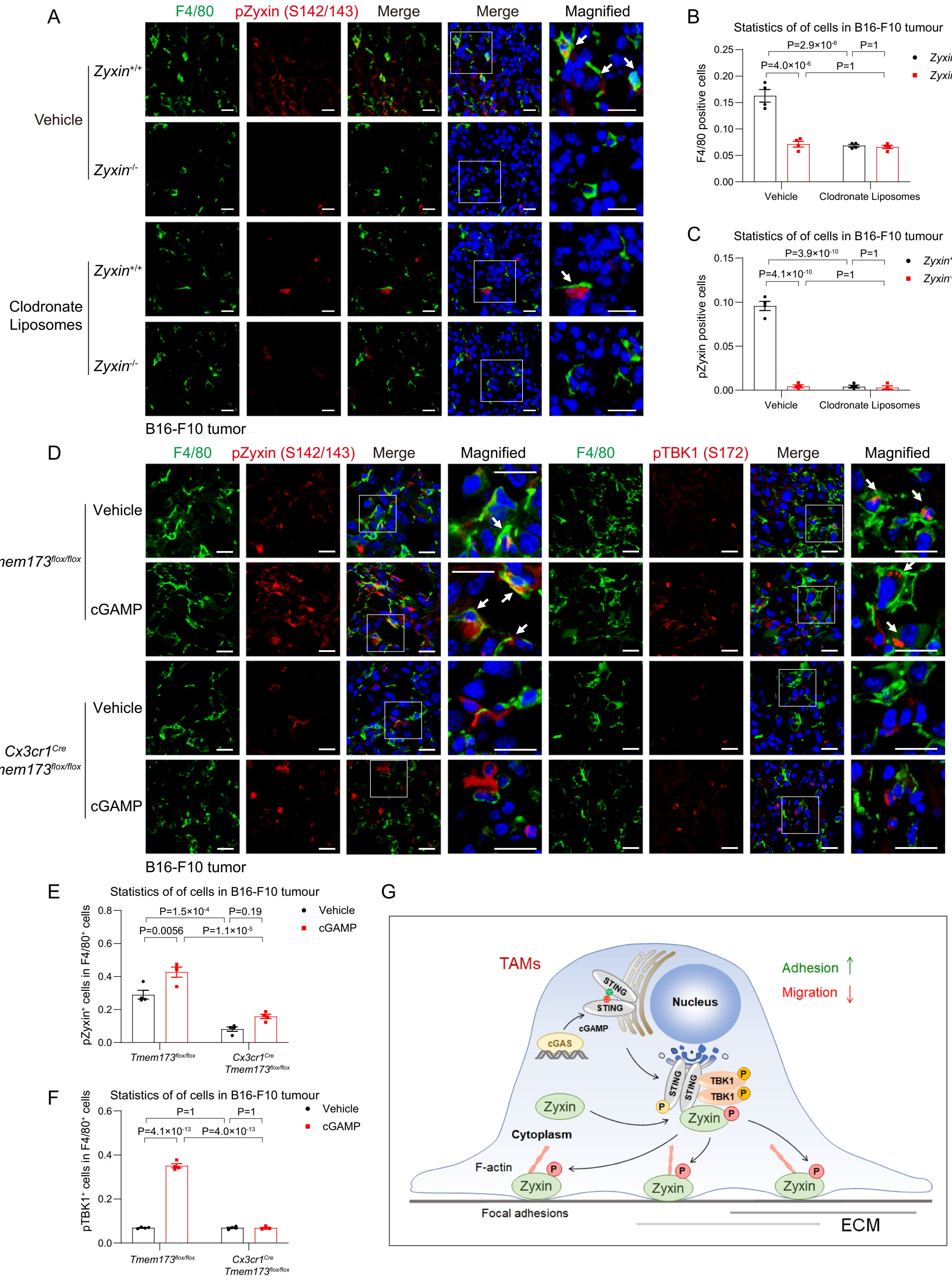

A
F4/80
pZyxin (S142/143)
Merge
Merge
Magnified
Vehicle
Zyxin+/+
Zyxin-/-
Clodronate Liposomes
B16-F10 tumor
B
Statistics of of cells in B16-F10 tumour
F4/80 positive cells
P=2.9×10-6
P=1
P=4.0×10-6
P=1
Vehicle
Clodronate Liposomes
C
Statistics of of cells in B16-F10 tumour
pZyxin positive cells
P=3.9×10-10
P=4.1×10-10
D
F4/80
pZyxin (S142/143)
pTBK1 (S172)
Tmem173flox/flox
Cx3cr1Cre Tmem173flox/flox
cGAMP
E
Statistics of of cells in B16-F10 tumour
pZyxin+ cells in F4/80+ cells
P=1.5×10-4
P=0.19
P=0.0056
P=1.1×10-5
F
pTBK1+ cells in F4/80+ cells
P=4.1×10-13
P=4.0×10-13
G
TAMs
Nucleus
STING
cGAMP
cGAS
Adhesion ↑
Migration ↓
TBK1
Zyxin
Cytoplasm
F-actin
Focal adhesions
ECM

Figure EV7. STING-TBK1-Zyxin signaling retains TAM residency to suppress antitumor immunity.

(**A–C**) Immunofluorescence imaging and statistics of F4/80$^+$ and pZyxin$^+$ cells in B16-F10 tumors. Administration of clodronate liposomes decreased F4/80$^+$ macrophages and pZyxin signals in B16-F10 tumors. Scale bars, 20 μm. White arrows indicated the pZyxin-positive TAMs. (**B**, **C**) $n = 4$ per group. (**D–F**) Immunofluorescence imaging and statistics of pZyxin (S142/143) and pTBK1 (S172) in B16-F10 tumors transplanted into *Sting1$^{flox/flox}$* or *Cx3cr1^{cre}Sting1$^{flox/flox}$* mice were shown, in the absence or presence of intratumoral injected cGAMP. cGAMP administration induced pZyxin and pTBK1 in TAMs, whereas tamoxifen-induced myeloid STING deficiency attenuated pZyxin and pTBK1 puncta in F4/80$^+$ macrophages. White arrows indicated the pTBK1-positive TAMs or pZyxin-positive TAMs. Scale bars, 20 μm. (**E**, **F**) $n = 4$ per group. (**G**) The diagram: cGAS-STING-Zyxin signaling converts biological signals into mechanical cues to reorganize the actin cytoskeleton that retains tumor-associated macrophages in the tumor microenvironment to mitigate antitumor immunity. Data information: unless otherwise indicated, $n = 3$ independent biological experiments (mean ± SEM). $^*P < 0.05$, $^{**}P < 0.01$, and $^{***}P < 0.001$, compared with the control condition (One-way ANOVA test and Bonferroni correction).

 © The Author(s)
